# Supplementary material for: Pregnancy-related interventions in mothers at risk for gestational diabetes in Asian India and low and middle-income countries (PRIMORDIAL study): protocol for a randomised controlled trial
Source: BMJ Open. 2021 Feb 17;11(2):e042069. doi: 10.1136/bmjopen-2020-042069 (PMC7893661; doi:10.1136/bmjopen-2020-042069)
Supplement: Supplementary data [file bmjopen-2020-042069supp002.pdf]

SPIRIT 2013 Checklist: PRIMORDIAL Study

Section/item	PageNo	Description
**Administrative information**		
Title	1	Descriptive title identifying the study design, population, interventions, and, if applicable, trial acronym
Trial registration	1	Trial identifier and registry name. If not yet registered, name of intended registry
Protocol version	1	Date and version identifier
Funding	1	Sources and types of financial, material, and other support
Roles and responsibilities	1	Names, affiliations, and roles of protocol contributors
	1	Name and contact information for the trial sponsor
	42	Role of study sponsor and funders, if any, in study design; collection, management, analysis, and interpretation of data; writing of the report; and the decision to submit the report for publication, including whether they will have ultimate authority over any of these activities
	39-41	Composition, roles, and responsibilities of the coordinating centre, steering committee, endpoint adjudication committee, data management team, and other individuals or groups overseeing the trial, if applicable (see Item 21a for data monitoring committee)
**Introduction**		
Background and rationale	9-16	Description of research question and justification for undertaking the trial, including summary of relevant studies (published and unpublished) examining benefits and harms for each intervention
Objectives	10,17	Specific objectives or hypotheses
Trial design	18	Description of trial design including type of trial (eg, parallel group, crossover, factorial, single group), allocation ratio, and framework (eg, superiority, equivalence, noninferiority, exploratory)
**Methods: Participants, interventions, and outcomes**		
Study setting	22-23	Description of study settings (eg, community clinic, academic hospital) and list of countries where data will be collected. Reference to where list of study sites can be obtained

Vasan SK, *et al. BMJ Open* 2021; 11:e042069. doi: 10.1136/bmjopen-2020-042069

Eligibility criteria	21	Inclusion and exclusion criteria for participants. If applicable, eligibility criteria for study centres and individuals who will perform the interventions (eg, surgeons, psychotherapists)
Interventions	19, 31	Interventions for each group with sufficient detail to allow replication, including how and when they will be administered
	29	Criteria for discontinuing or modifying allocated interventions for a given trial participant (eg, drug dose change in response to harms, participant request, or improving/worsening disease)
Outcomes	17, 25, 35	Primary, secondary, and other outcomes, including the specific measurement variable (eg, blood glucose, OGTT), analysis metric (eg, change from baseline, final value, time to event), method of aggregation (eg, median, proportion), and time point for each outcome. Explanation of the clinical relevance of chosen efficacy and harm outcomes is strongly recommended
Participant timeline	22-26	Time schedule of enrolment, interventions (including any run-ins and washouts), assessments, and visits for participants. A schematic diagram is highly recommended (see Figure)
Sample size	34,35	Estimated number of participants needed to achieve study objectives and how it was determined, including clinical and statistical assumptions supporting any sample size calculations
Recruitment	22,23	Strategies for achieving adequate participant enrolment to reach target sample size

Methods: Assignment of interventions (for controlled trials)

Allocation:

Sequence generation	24	Method of generating the allocation sequence (eg, computer-generated random numbers), and list of any factors for stratification. To reduce predictability of a random sequence, details of any planned restriction (eg, blocking) should be provided in a separate document that is unavailable to those who enrol participants or assign interventions
Implementation	24	Who will generate the allocation sequence, who will enrol participants, and who will assign participants to interventions

Methods: Data collection, management, and analysis

Data collection methods	37-38	Plans for assessment and collection of outcome, baseline, and other trial data, including any related processes to promote data quality (eg, duplicate measurements, training of assessors) and a description of study instruments (eg, questionnaires, laboratory tests) along with their reliability and validity, if known. Reference to where data collection forms can be found, if not in the protocol

Data management	36-38	Plans for data entry, coding, security, and storage, including any related processes to promote data quality (eg, double data entry; range checks for data values). Reference to where details of data management procedures can be found, if not in the protocol
Statistical methods	33-36	Statistical methods for analysing primary and secondary outcomes. Reference to where other details of the statistical analysis plan can be found, if not in the protocol
	35,36	Methods for any additional analyses (eg, subgroup and adjusted analyses)
	33,36	Definition of analysis population relating to protocol non-adherence (eg, as randomised analysis)
**Methods: Monitoring**		
Data monitoring	39	Composition of data safety monitoring committee (DSMC); summary of its role and reporting structure; statement of whether it is independent from the sponsor and competing interests; and reference to where further details about its charter can be found, if not in the protocol. Alternatively, an explanation of why a DMC is not needed
Harms	32	Plans for collecting, assessing, reporting, and managing solicited and spontaneously reported adverse events and other unintended effects of trial interventions or trial conduct
**Ethics and dissemination**		
Research ethics approval	41	Plans for seeking research ethics committee/institutional review board (REC/IRB) approval
Protocol amendments	40,54-60	Plans for communicating important protocol modifications (eg, changes to eligibility criteria, outcomes, analyses) to relevant parties (eg, investigators, REC/IRBs, trial participants, trial registries, journals, regulators)
Consent or assent	23	Who will obtain informed consent or assent from potential trial participants or authorised surrogates, and how
Confidentiality	38	How personal information about potential and enrolled participants will be collected, shared, and maintained in order to protect confidentiality before, during, and after the trial
Access to data	38	Statement of who will have access to the final trial dataset, and disclosure of contractual agreements that limit such access for investigators

Vasan SK, *et al. BMJ Open* 2021; 11:e042069. doi: 10.1136/bmjopen-2020-042069

Dissemination policy	43	Plans for investigators and sponsor to communicate trial results to participants, healthcare professionals, the public, and other relevant groups (eg, via publication, reporting in results databases, or other data sharing arrangements), including any publication restrictions
	43	Authorship eligibility guidelines and any intended use of professional writers
Biological specimens	28	Plans for collection, laboratory evaluation, and storage of biological specimens for genetic or molecular analysis in the current trial and for future use in ancillary studies, if applicable

*It is strongly recommended that this checklist be read in conjunction with the SPIRIT 2013 Explanation & Elaboration for important clarification on the items. Amendments to the protocol should be tracked and dated. The SPIRIT checklist is copyrighted by the SPIRIT Group under the Creative Commons "Attribution-NonCommercial-NoDerivs 3.0 Unported" license.

Vasan SK, *et al. BMJ Open* 2021; 11:e042069. doi: 10.1136/bmjopen-2020-042069
