# Supplementary material for: Pregnancy-related interventions in mothers at risk for gestational diabetes in Asian India and low and middle-income countries (PRIMORDIAL study): protocol for a randomised controlled trial
Source: BMJ Open. 2021 Feb 17;11(2):e042069. doi: 10.1136/bmjopen-2020-042069 (PMC7893661; doi:10.1136/bmjopen-2020-042069)
Supplement: Author's manuscript [file bmjopen-2020-042069.draft_revisions.pdf]

BMJ Open

BMJ Open is committed to open peer review. As part of this commitment we make the peer review history of every article we publish publicly available.

When an article is published we post the peer reviewers' comments and the authors' responses online. We also post the versions of the paper that were used during peer review. These are the versions that the peer review comments apply to.

The versions of the paper that follow are the versions that were submitted during the peer review process. They are not the versions of record or the final published versions. They should not be cited or distributed as the published version of this manuscript.

BMJ Open is an open access journal and the full, final, typeset and author-corrected version of record of the manuscript is available on our site with no access controls, subscription charges or pay-per-view fees (http://bmjopen.bmj.com).

If you have any questions on BMJ Open's open peer review process please email info.bmjopen@bmj.com

BMJ Open

Protocol for a randomised controlled trial: Pregnancy Related Interventions in Mothers at risk for gestational Diabetes in Asian Indian and Low and middle income countries (PRIMORDIAL Study)

Journal:	*BMJ Open*
Manuscript ID	bmjopen-2020-042069
Article Type:	Protocol
Date Submitted by the Author:	25-Jun-2020

Complete List of Authors:	Vasan, Senthil; University of Oxford, Radcliffe Department of Medicine; University of Southampton, MRC Lifecourse Epidemiology Unit<br>Jobe, Modou ; Medical Research Council The Gambia<br>Mathews, Jiji; Christian Medical College and Hospital Vellore, Obstetrics and Gynaecology<br>Cole , Fatoumata; MRC Unit The Gambia at London School of Hygiene & Tropical Medicine<br>Rathore, Swathi; Christian Medical College and Hospital Vellore, Obstetrics and Gynaecology<br>Jarjou, Ousman; MRC Unit The Gambia at London School of Hygiene & Tropical Medicine, Nutrition<br>Thompson, Dylan; University of Bath, Department for Health<br>Jarde, Alexander; MRC Unit The Gambia at London School of Hygiene & Tropical Medicine, , Statistics<br>Bittaye, Mustapha ; MRC Unit The Gambia at London School of Hygiene & Tropical Medicine<br>Ulijaszek, Stanley; University of Oxford, School of Anthropology<br>Fall, Caroline ; University of Southampton, MRC Lifecourse Epidemiology Unit<br>Osmond, Clive; Medical Research Council, Epidemiology Resource Centre<br>Prentice, Andrew; London School of Hygiene andTropical Medicine, MRC International Nutrition Group<br>Karpe, Fredrik; University of Oxford , Oxford center for Diabetes, Endocrinology and Metabolism

Keywords:	OBSTETRICS, PREVENTIVE MEDICINE, PUBLIC HEALTH

SCHOLARONE™ Manuscripts

I, the Submitting Author has the right to grant and does grant on behalf of all authors of the Work (as defined in the below author licence), an exclusive licence and/or a non-exclusive licence for contributions from authors who are: i) UK Crown employees; ii) where BMJ has agreed a CC-BY licence shall apply, and/or iii) in accordance with the terms applicable for US Federal Government officers or employees acting as part of their official duties; on a worldwide, perpetual, irrevocable, royalty-free basis to BMJ Publishing Group Ltd ("BMJ") its licensees and where the relevant Journal is co-owned by BMJ to the co-owners of the Journal, to publish the Work in this journal and any other BMJ products and to exploit all rights, as set out in our licence.

The Submitting Author accepts and understands that any supply made under these terms is made by BMJ to the Submitting Author unless you are acting as an employee on behalf of your employer or a postgraduate student of an affiliated institution which is paying any applicable article publishing charge ("APC") for Open Access articles. Where the Submitting Author wishes to make the Work available on an Open Access basis (and intends to pay the relevant APC), the terms of reuse of such Open Access shall be governed by a Creative Commons licence – details of these licences and which Creative Commons licence will apply to this Work are set out in our licence referred to above.

Other than as permitted in any relevant BMJ Author's Self Archiving Policies, I confirm this Work has not been accepted for publication elsewhere, is not being considered for publication elsewhere and does not duplicate material already published. I confirm all authors consent to publication of this Work and authorise the granting of this licence.

For peer review only

Protocol for a randomised controlled trial: Pregnancy Related Interventions in Mothers at risk for gestational Diabetes in Asian India and Low and middle income countries (PRIMORDIAL Study)

Senthil K Vasan[1,2], Modou Jobe[3], Jiji Mathews[4], Fatoumata Cole[3], Swathi Rathore[4], Ousman Jarjou[3], Dylan Thompson[5], Alexander Jarde[3], Mustapha Bittaye[3], Stanley J Ulijaszek[6], Caroline H D Fall[2], Clive Osmond[2], Andrew M Prentice[3], Fredrik Karpe[1,7]

[1]Oxford Centre for Diabetes, Endocrinology and Metabolism (OCDEM), University of Oxford Churchill Hospital, Oxford OX3 7LE, UK

[2]MRC Lifecourse Epidemiology Unit, Southampton General Hospital, University of Southampton, Tremona Road, Southampton, UK

[3]MRC Unit The Gambia at London School of Hygiene & Tropical Medicine, Fajara, The Gambia

[4]Department of Obstetrics & Gynaecology, Christian Medical College & Hospital, Vellore. Tamil Nadu, India

[5]Department for Health, Centre for Therapeutic Innovation, University of Bath, UK

[6]Institute of Social and Cultural Anthropology, University of Oxford, Oxford, UK

[7]NIHR Oxford Biomedical Research Centre, OUH Foundation Trust, Oxford, UK

Key words: gestational diabetes, yoghurt, physical activity, Africa, India, randomised controlled trial

Corresponding author:
Prof Fredrik Karpe
Oxford Centre for Diabetes, Endocrinology and Metabolism (OCDEM),
University of Oxford
Churchill Hospital
Oxford OX3 7LE
UK
Email: Fredrik.Karpe@ocdem.ox.ac.uk
Tel: +44-1865-857222

For peer review only - http://bmjopen.bmj.com/site/about/guidelines.xhtml

Abstract

Introduction: Lifestyle modification is the mainstay of gestational diabetes mellitus (GDM) prevention. However, clinical trials evaluating the safety and efficacy of diet or physical activity (PA) in low-and middle-income settings such as Africa and India are lacking. This trial aims to evaluate the efficacy of yoghurt consumption and increased PA (daily walking) in reducing GDM incidence in high-risk pregnant women.

Methods and analysis: The study is a 2x2 factorial, open-labelled, multi-centre randomized controlled trial to be conducted in Vellore, South India and The Gambia, West Africa. "High-risk" pregnant women (n=1,856) aged ≥18 years and ≤16 weeks of gestational age (GA), with at least one risk factor for developing GDM, will be randomized to either (i) yoghurt (ii) PA (iii) yoghurt + PA or (iv) standard antenatal care. Participants will be followed until 32 weeks of gestation with total active intervention lasting for a minimum of 16 weeks. The primary endpoint is GDM incidence at 26-28 weeks diagnosed using International Association of the Diabetes and Pregnancy Study Groups (IADPSG) criteria or elevated fasting glucose (≥5.1 mmol/l) at 32 weeks. Secondary endpoints include absolute values of fasting plasma glucose concentration at 32 weeks gestation, maternal blood pressure, gestational weight gain, intrapartum and neonatal outcomes. Analysis will be both by intention to treat and per-protocol. Continuous outcome measurements will be analysed using multiple linear regression and binary variables by logistic regression.

Ethics and dissemination: The study is approved by Oxford Tropical Research Ethics Committee (OxTREC 44-18), ethics committees of the Christian Medical College, Vellore (IRB 11367) and MRCG Scientific Coordinating Committee (SCC 1645) and The Gambia Government/MRCG joint ethics committee (L2020.E15). Findings of the study will be published in peer-reviewed scientific journals and presented in conferences.

Trial registration number: ISRCTN: 18467720 and Clinical Trials Registry, India (CTRI/2018/07/014947)

Word count:
Abstract: 294/300
Manuscript: 3914/4000

For peer review only

For peer review only - http://bmjopen.bmj.com/site/about/guidelines.xhtml

Strengths and limitations of the study

- This study will be the largest lifestyle intervention trial among high-risk GDM mothers in India and Africa to evaluate the efficacy of daily yoghurt consumption and physical activity in reducing GDM incidence.
- The selective screening and intervention in high-risk women using low-cost interventions is suitable for GDM prevention in low-resource settings such as India and Africa.
- The trial will help understand barriers to lifestyle interventions during pregnancy and will thus help tailor lifestyle interventions that are cost-effective as well as socially and culturally acceptable to pregnant women in these populations.

For peer review only

BACKGROUND

Gestational Diabetes Mellitus (GDM) accounts for 86% of hyperglycaemia during pregnancy[1] and is associated with significant intrapartum complications, perinatal morbidity, and long-term risk of developing type 2 diabetes (T2D) and cardiovascular disease in both the mother and the offspring.[2-4] Consequent to the global rise in obesity prevalence and the introduction of more stringent diagnostic criteria by the International Association of the Diabetes and Pregnancy Study Groups (IADPSG), the global prevalence of GDM has been estimated at 17.8% (range 9.3–25.5%).[5] In low- and middle-income countries (LMICs), especially in India and Africa, there are few nation-wide estimates of GDM burden. Cross-sectional studies using variable diagnostic criteria report GDM prevalence ranging between 10 to 16% in India[6 7] and 11 to 14% in Africa[8 9], which is higher than the reported prevalence from high income countries like the UK (2-3% using NICE guidelines) and USA (2-10% using various criteria including IADPSG).[10 11] Direct comparisons of these estimates may not be appropriate due to inconsistency in diagnostic criteria. Nevertheless, it seems clear that LMICs are facing a high burden of GDM.

In normal pregnancy, the physiological response to reduced insulin-mediated glucose removal is an increase in insulin secretion to maintain normoglycaemia.[12] In GDM, insufficient β-cell plasticity leads to an inability to secrete adequate quantities of insulin to counterbalance the insulin resistance (IR), and this process is accelerated in obesity. Women with chronic IR states such as pre-pregnancy excess body weight or obesity, impaired glucose tolerance prior to pregnancy, polycystic ovarian syndrome (PCOS), previous GDM, advanced maternal age and rapid gestational weight gain (GWG), have a higher risk for developing glucose intolerance during pregnancy.[13] The hyperglycaemia of GDM rapidly abates following delivery and normoglycaemia normally returns within 12 weeks. However, 50-70% of GDM mothers progress to T2D within 5-10 years postpartum[14] suggesting that GDM may be a prodrome of "common T2D" and may reflect the underlying T2D frequency in the community.[15]

Screening for GDM

Following the demonstration of a linear relationship between maternal glycaemia and obstetric and perinatal outcomes by the Hyperglycemia and Adverse Pregnancy Outcome (HAPO) study,[16 17] the IADPSG recommends universal screening of all women at first antenatal visit, without restriction to high-risk pregnant women, in order to identify all potential GDM cases.[18] However, some countries have adopted selective screening, despite the possibility of missing over 40% of GDM cases.[19] In low economic setting like the LMICs, this approach may be more cost effective and oral glucose tolerance test (OGTT) may be less beneficial to low risk women.[20]

GDM management

The main goal of lifestyle modification using diet and PA in pregnancy is to prevent excessive GWG, improve materno-foetal outcomes and prevent the onset/subsequent progression to GDM/T2D in both the mother and the new born.[21] In order to maximize the benefit of such interventions, the best strategy is likely to target high-risk populations by initiating early interventions during the "critical window", i.e. before insufficient β-cell plasticity sets in[22] (**Figure 1**).

Dietary interventions in Pregnancy

For peer review only - http://bmjopen.bmj.com/site/about/guidelines.xhtml

Meta-analysis of dietary intervention trials in pregnant women, have shown that diet modification in any form resulted in a 61% risk reduction in GDM incidence (relative risk (RR) 0.39, 95%CI 0.23-0.69).[23-26] Besides macronutrient modification, dairy products, particularly yoghurt has been shown to lower the risk of T2D, [27] probably because of their probiotic effect of reducing inflammatory signalling and gut permeability giving rise to a range of putative signals from the gut to the systemic circulation.[28-30] However, it should be noted that these studies were not in pregnant women. Differences in gut microbial composition between healthy pregnant and GDM women provide evidence of a potential role of altered gut microbiota in pregnancy-related metabolic dysfunction.[31 32]

Manipulation of the gut microbiome during pregnancy, using fermented dairy products or probiotics, has been shown to positively impact on maternal weight, blood pressure, insulin resistance and plasma lipid profile.[31 33-36] The "Probiotics and Pregnancy outcome" study showed that incident GDM was lower in women supplemented with probiotics compared to diet/placebo or control groups (16% vs 36% vs 34%).[37] The Nutrition, Allergy, Mucosal immunology and Intestinal microbiota (NAMI) study showed reductions in glucose and insulin concentration, GDM incidence and central adiposity, possibly mediated by anti-inflammatory cytokine production in probiotic supplemented pregnant women.[38 39] Multiple pieces of evidence also support a positive relationship between probiotic yoghurt supplementation during pregnancy and improved glycaemic profile in women with GDM or obesity.[40-42]

Physical activity in pregnancy

PA directly impacts non-insulin mediated glucose utilization through translocation of glucose transporter type 4 (GLUT 4) on skeletal muscle.[43 44] PA during pregnancy is safe and ACOG guidelines recommend ≥30 minutes of moderate PA performed at an intensity of 3-6 metabolic equivalents, which corresponds to brisk walking at 5-7km/h on most days of the week.[45]

Evidence of a beneficial effect from PA-intervention during pregnancy mostly come from high-income countries, and include a wide range of exercise programme that vary in their intensity, duration, frequency and assessment techniques. Results show that regular PA in any form improves maternal (hyperglycaemia, hypertension, weight gain and cardiovascular function)[46], obstetric (pre-eclampsia, shoulder dystocia, preterm births), and neonatal outcomes (low birth weight or small-for-gestational age, decreased fat mass, improved stress tolerance and advanced neurobehavioural maturation).[47]

Meta-analysis of RCTs of PA intervention (8 studies, n=1,441) in obese/overweight women has shown a 24% reduction in GDM incidence in the intervention groups (RR 0.76, 95% CI 0.56-1.03; p=0.07) with moderate heterogeneity between studies (I^2=50%, p=0.05).[48] Some studies have shown negative or neutral effects, including a Cochrane review (5 studies, n=1,115) of PA on GDM incidence,[49-51] and this is likely to be related to study heterogeneity, bias, type of exercise, timing, duration and intensity of exercise and also subjective assessments. Nevertheless, the potential beneficial effects of effective PA intervention in pregnancy should not be underestimated.

The knowledge gap on effectiveness of PA interventions in LMIC settings is particularly challenging in women from Asian Indian and African backgrounds where the baseline PA level

For peer review only - http://bmjopen.bmj.com/site/about/guidelines.xhtml

is generally considered low. The ability to engage pregnant women from diverse ethnic groups will also be determined by population-specific socioeconomic and cultural factors.

Combined approaches in Pregnancy

Targeting multiple risk factors simultaneously may have a synergistic effect to reduce GDM. Combined approaches have shown favourable improvements in maternal and foetal outcomes, particularly GWG, which is a potentially modifiable risk factor for GDM development.[52-56] Two RCTs using combined interventions in high-risk women (BMI ≥ 30kg/m^2), the UPBEAT[57] and RADIEL trial,[58] showed 0.55 kg and 0.58 kg reduction in GWG, respectively. The RADIEL trial additionally showed about 36% reduction in GDM incidence (crude RR 0.64; 95% CI 0.38–1.09) in the intervention group. A recent Cochrane review of combined interventions showed a tendency towards reduced GDM risk (RR 0.85, 95%CI 0.71-1.01; 6,633 women, 19 RCTs) and reduced weight gain (mean difference -0.89 kg, 95%CI -1.39 to -0.40; 5,052 women, 16RCTs).[59]

HYPOTHESIS

We hypothesize that daily yoghurt consumption and/or increased PA will reduce the risk of developing GDM in 'high-risk' pregnant women.

AIMS

To examine if daily yoghurt consumption and/or PA beginning from ≤16 weeks of gestation in 'high-risk' pregnant women can reduce the incidence of GDM in two LMIC settings.

OBJECTIVES

Primary objective

To establish the efficacy and safety of low-cost interventions (fermented yoghurt consumption/daily walking/both) to prevent GDM in two LMIC settings.

Secondary objectives

(i) To evaluate the effect of yoghurt/daily walking/combination of both on fasting glucose concentration at 32 weeks of gestation, GWG, blood pressure, intrapartum and neonatal outcomes.
(ii) To identify barriers that prevent participation/adherence to lifestyle changes during pregnancy and thus design culturally and socially acceptable, cost effective interventions that can be implemented successfully in LMICs.

CENTRES

Christian Medical College, Vellore, India
MRC Unit at LSHTM, Fajara, The Gambia

DESIGN

The study is a 2 x 2 factorial design and eligible women will be randomized to either one of the arms – (i) yoghurt (ii) PA (iii) yoghurt + PA (iv) standard care

ELIGIBILITY CRITERIA

For peer review only - http://bmjopen.bmj.com/site/about/guidelines.xhtml

The study will include pregnant women aged ≥18 years and GA ≤16 weeks, and meeting at least one risk-factor for GDM (booking BMI ≥25kg/m^2, age ≥25 years; a first-degree relative with diabetes; a previous pregnancy complicated by GDM, pre-eclampsia/eclampsia and/or a large baby (≥3.5kg); a history of PCOD/impaired fasting glucose) and not currently on any medication that is known to interfere with glucose metabolism.

Exclusion criteria

- GDM diagnosed prior to screening visit based on IADPSG criteria or documented raised HbA1C, i.e., either fasting glucose ≥5.1 mmol/L or 1h glucose ≥10.0 mmol/L or 2h glucose ≥8.5 mmol/L, or a documented HbA1c of ≥6.5% at first booking
- History of pre-gestational diabetes, severe hyperemesis in the first trimester
- History of recurrent (≥2) first trimester spontaneous abortions, stillbirth, significant ante- or post-partum haemorrhage in previous pregnancies
- Multiple gestation in the current pregnancy
- Uncontrolled pre-gestational or gestational hypertension (BP>150/100 mmHg) on treatment
- Previous child born with congenital anomalies
- Pregnancy following in-vitro fertilization or any assisted reproductive technology
- Previous or current psychiatric illness on medication, epileptic seizures or on anti-epileptic medication
- Women meeting absolute contraindications for physical activity during pregnancy as recommended by the ACOG (heart disease, restrictive lung disease, incompetent cervix/cerclage, pregnancies at risk for premature labour, gestational hypertension, severe anaemia)

METHODS

Women attending obstetric outpatient clinics of the Christian Medical College, Vellore, India and MRC Unit in The Gambia operating in four centres in The Gambia (Brikama Major Health Centre, Bundung Maternal and Child Hospital, Barfrow Medical Centre and Edward Francis Small Teaching Hospital) will be informed about the study and assessed for eligibility. Following informed consent, women enrolled in the study will be asked to attend five scheduled visits (screening, run-in phase, randomisation, visits 1, 2 and 3) as shown in **Figure 2** and detailed in the protocol **(online supplementary file 1)**.

RANDOMISATION

Participants will be randomly allocated to one of the following arms (i) yoghurt (ii) PA (iii) yoghurt + PA or (iv) standard care following an allocation schedule pre-stratified for centre, age and BMI. A variable block randomisation, stratified by centre, and then by age, (<25 and ≥25 years) and BMI (<25 and ≥25 kg/m^2) will be generated, using a password-protected access database developed by an independent statistician not involved with trial participants. In India, the allocation will be performed using a computer interface (Access database), while separate computer-generated randomisation lists for each strata combination (age X BMI) will be prepared for each health facility in the Gambia. Due to logistic limitations, this will be implemented using pre-issued opaque, sealed envelopes containing the allocation code.

For peer review only - http://bmjopen.bmj.com/site/about/guidelines.xhtml

INTERVENTIONS

Dietary intervention Yoghurt will be prepared locally at the dietary department in CMC, Vellore and at Kombo Dairy farm in The Gambia based on a common recipe and using the same starter culture containing *lactobacillus sp* and *Bifidobacterium sp* at 10^7 CFU. Yoghurt will be prepared in batches and an internal quality check will be performed to ensure that the bacterial species and colony count are maintained with each batch. Participants in the active yoghurt arm will consume 200 g/day of regular, unflavoured, fermented yoghurt throughout the active intervention period independent of the timing of their regular meal. Yoghurt will be dispensed either daily or weekly as feasible by the study centre and possibility of storage in refrigerator at participants' homes when not in use. Compliance with yoghurt will be assessed using pot count by field workers. All participants will receive dietary counselling, will not be advised to stop their regular dairy consumption, assuming that any effect on the outcome will be related to the trial yoghurt consumed. Information on background dairy consumption will be collected at each scheduled visit using a standardized questionnaire.

Physical activity intervention The PA activity intervention will include daily walking to increase the individual target step count, monitored using a wrist-mounted accelerometer device (Garmin Vivofit 4 fitness band).

The baseline step count will be recorded in all participants (blinding the step count on the device) for 7 days, with no emphasis on step count and/or activity during the run-in phase. At randomisation, baseline step count will be calculated as the average over 7 days including two weekend days. An individual target will be set, to a 40% or greater increase in daily step count compared with the baseline reading. The step count reading on the device will be unblinded after randomisation for women in the active PA arms, to provide visual feed back to the participants and to motivate them to achieve their daily target step count. Women in the non-PA arm will continue to use their fitness bands blinded throughout the study to measure their routine PA behaviour. Step count data will be periodically uploaded using Garmin application to provide visual feedback, compliance monitoring and counselling to participants in the PA arm.

STUDY ASSESSMENT

Clinical measurements

Detailed study-related procedures, as outlined in **Table 1** and routine antenatal examination will be carried out at all study visits. All measurements will be standardized between the two study centres and will be carried out as detailed in study specific standard operating procedures (SOPs).

Table 1. Study-related procedures at each study visit

For peer review only

Procedures	Visits						
	Visit -2	Visit -1	Visit 0	Visit 1	Visit 2	Visit 3	Delivery
	Screening ≤16 weeks	Run-in-phase ≤17 weeks	Randomization Week ≤18	Week 18-20	Week 26-28	Week 32	
Informed consent	X						
Eligibility assessment	X						
Demographics	X						
History	X						
Height	X						
Weight	X	X	X	X	X	X	X
Blood pressure	X	X	X	X	X	X	X
Antenatal examination	X	X	X	X	X	X	
Dairy consumption questionnaire		X	X	X	X	X	
Barriers questionnaire	X					X	
Baseline PA monitoring with Vivofit 4		X					
PA step-count monitoring			X	X	X	X	
Randomisation			X				
OGTT	X				X		
Dating scan	X						
Morphology scan				X			
Ultrasound scan (growth)						X	
Fasting plasma glucose						X	
Intrapartum assessment (mother)							X
New born assessment							X

Laboratory tests

Study participants will undergo a 75g OGTT at screening and visit 2 (week 26-28 GA) following an 8 hour overnight fast. Additional fasting plasma glucose will be measured at the end-of-study visit (week 32). Blood glucose measurements will be analysed using a Roche Cobas 800 autoanalyser at respective laboratories (Department of Clinical Biochemistry in Vellore, India

For peer review only - http://bmjopen.bmj.com/site/about/guidelines.xhtml

and the MRCG at LSHTM, The Gambia). Other routine ante-natal laboratory tests, including haemoglobin will be done as a part of routine antenatal care according to local guidelines.

GDM ascertainment

IADPSG criteria will be used to diagnose GDM using an OGTT at week 26-28. GDM diagnosis will be established if either fasting glucose ≥5.1 mmol/L or 1h glucose ≥10.0 mmol/L or 2h glucose ≥8.5 mmol/L.

Questionnaire based assessments

Standardized questionnaires will be used to obtain information on social demographics, background dairy consumption and to identify barriers to lifestyle intervention during pregnancy in all participating women. The barriers questionnaire is specifically designed to address ethnic-specific social and cultural barriers to lifestyle intervention during pregnancy in both populations and would be administered at the start and the end of the study.

Ultrasound scans

Ultrasound scans will be performed by a qualified obstetrician on Voluson GE S-8 (in India) and Aloka USI-145 (in Gambia) ultrasound machines on all participates at three visits, i) at screening (dating) ii) morphology scan at visit 1 (18-20 weeks GA) and iii) at visit 3 (week 32) for growth monitoring. Both centres will follow standard protocols as described in study standard operating procedures (SOPs). Evidence of foetal congenital anomaly, placental malformation or malposition or abnormal ultrasound features that can compromise pregnancy outcome will lead to termination of the participant's involvement in the study and referral for the normal care pathway at each participating centre.

OUTCOMES

Primary outcome

Incidence of GDM diagnosed using IADPSG criteria between 26-28 weeks of gestation or if the fasting plasma glucose concentration is ≥5.1 mmol/l at 32 weeks of gestation.

Secondary outcomes

Effect of the intervention on fasting blood glucose concentration at 32 weeks, gestational weight gain and blood pressure from randomisation to week 32, proportion of women undergoing instrumental/caesarean delivery, proportion of women developing pre-eclampsia and eclampsia, blood loss during delivery, post-partum haemorrhage, pre-term births (<37 weeks GA), foetal macrosomia defined as birth weight >2 SDs above the population-specific mean in each setting, birth weight and length, APGAR score at 1 and 5 minutes and responses to questionnaire based assessments of barriers to lifestyle interventions in pregnancy.

ADVERSE (AE)/SERIOUS ADVERSE EVENTS (SAEs) AND REPORTING

All AE and SAEs will be captured in respective electronic CRFs. Details of all events including onset date, relationship to the study intervention, treatment given, and stop date will be captured in the respective AE report form. The PI will report all SAEs without filtration, whether or not related to the intervention, within 24 hours of becoming aware of the event, to the sponsor. All SAEs will be notified to the respective Ethics Committee within seven calendar days if fatal or life-threatening, and all others within 15 calendar days. All AEs and

For peer review only - http://bmjopen.bmj.com/site/about/guidelines.xhtml

SAEs will also be notified to the Data Safety and Monitoring Committee (DSMC). Expected SAEs which are protocol-defined exceptions to SAE reporting will be captured and reported to respective ECs and DSMC as outlined in the protocol **(online supplementary file 1)**

STATISTICAL ANALYSIS PLAN INCLUDING SAMPLE SIZE AND POWER CALCULATION

Sample size

The sample size calculation was performed by simulation (in R version 3.5.2) using an algorithm as described in detail in the protocol (**online supplementary file 1**). After 10,000 repetitions, we estimated the sample size to be 1856 high-risk women (928 from each centre). The sample size is estimated to have 80% power to detect a 33% reduction in the incidence of GDM, for the main effects of diet and PA, with a family-wise significance level of 5% for a two-sided test (i.e. each effect was tested at a significance level of 2.5%). It was assumed that participants could be pooled across centres (no interaction between interventions and country at the 5% level). The sample size was inflated to allow for a 15% drop out rate in each of the four strata, with drop outs defined as non-compliant participants (i.e. no risk reduction in their incidence of GDM).

Data analysis

Data will be analysed using both (1) the intention-to-treat principle, including all enrolled participants regardless of their adherence to the proposed intervention, and (2) a per protocol approach, including only those participants adhering to at least 75% of the proposed intervention. In addition, we will run a sensitivity analysis to assess the effect of protocol deviation compared to 100% adherence.

Descriptive analyses at baseline

We will describe baseline characteristics of the four groups (Control, yoghurt, PA, and yoghurt + PA) using means and standard deviations (normally distributed quantitative variables), medians and interquartile ranges (non-normally distributed quantitative variables), or proportions (categorical variables).

Primary analysis

For our primary outcome (presence or absence of GDM), we will use multiple logistic regression models and results presented as OR and 95% CI. We will examine the assumption that there are no interaction effects between study centre and each of the interventions by fitting a multiple logistic regression model.

We will build two covariate adjusted models. First, we will assess the relative difference between centres in the effects of each intervention on GDM by adding a term for study centre. Second, we will fit a covariate (age, pre-pregnancy BMI, previous history of GDM and GA) adjusted model adding further terms for each, as well as relevant interaction terms, to remove bias from the estimate of the treatment effect on the primary outcome. Each covariate will be added to the unadjusted model separately first and then added one at a time and kept in the model if their significance level is <10%.

Secondary analyses

For peer review only - http://bmjopen.bmj.com/site/about/guidelines.xhtml

Continuous variables will be analysed using multiple linear regression and binary variables by multiple logistic regression. For each secondary outcome, we will fit a simple model with only terms for each intervention, and a covariate adjusted model including terms for study centre and the remaining pre-specified covariates and relevant interactions.

DATA COLLECTION

Research data will be collected using electronic CRFs (eCRFs) on tablets by designated study staff after training. Where there is internet connectivity, collected data will be stored directly onto the central server housed at MRC, Fajara and where there is no/unreliable connectivity, data will be stored locally on the tablet and later synchronized to the central servers. The accelerometer data used for PA monitoring will be directly synced with the Garmin app on the tablets on a weekly basis and transferred to eCRFs. To ensure standardisation of processes, SOPs with respect to trial management, quality assurance, data management, IT & security, and statistics will be adhered to.

COMMITTEE OVERSIGHTS

The study will be conducted in accordance with the approved protocol, Good Clinical Practice regulations and SOPs. The implementation of the study will be overseen by the Trial Steering committee (TSC). An independent DSMC, comprised of experts in clinical trials/epidemiology, biostatistics and obstetrics will meet 6-monthly to review the progress of the trial (recruitment, compliance, loss to follow-up), clinical study safety data, data quality and assess and/or adjudicate all events of deaths, the GDM event rate and key secondary/safety outcomes and provide feedback to the TSC. The DSMC will also meet if required, more frequently depending on the enrolment rate, safety issues and AEs. A copy of the full study protocol and all amendments would be submitted to DSMC prior to study initiation and subsequently. Thereafter, periodic reports including summary of enrolment, demographics, protocol compliance and safety will be compiled by the study statistician and submitted at least one week prior to the scheduled meeting. The board will advise on continuation or stopping the trial based upon safety and efficacy.

ETHICS AND DISSEMINATION

Ethical issues

The proposed interventions are considered generally safe both for maternal and foetal health. Participation in this trial is voluntary and subject to informed consent. A sample informed consent statement is available in the study protocol (**online supplementary file 1).** Participants who develop GDM at the visit 2 OGTT will be started on pharmacological therapy (as per local management guidelines) and results will be analysed as per intention to treat. Research participants can withdraw from the study at any time during the trial. The SPIRIT Checklist for the protocol is detailed in **online supplementary file 3.**

Dissemination

Results of the study will be presented at major national and international conferences, and publish in peer-reviewed journals, ensuring open access and the highest possible impact. The de-identified data will be openly available to the scientific community after publication of initial results. We will interact with national and international health agencies and governments to stimulate translation of our findings into policy and expect our research to influence policy for mothers and children within the next 5-10 years.

For peer review only - http://bmjopen.bmj.com/site/about/guidelines.xhtml

Patient and public involvement
The trial was informed by patients' need for an effective preventive therapies for GDM since randomised trial data are lacking in these populations. Patients were not involved in the design, conduct or reporting of the study. A summary of the main trial results will be disseminated to the general public and study participants after the completion of the study.

DISCUSSION
GDM poses increased risks to maternal and child health. LMICs face numerous challenges to GDM care at various levels. These include (i) late second trimester screening for GDM, ii) lack of standardized nation-wide guidelines for diagnosis, iii) interventions targeting only women who are diagnosed with GDM around 24-28 weeks, iv) lack of knowledge and awareness of GDM and its health implications and v) sociocultural barriers to early antenatal care and life-style interventions during pregnancy. It seems very likely that early screening and establishment of positive lifestyle changes beginning from conception, or even prior to conception, particularly in selected high-risk women, and especially in low-resource settings, will reduce the burden of GDM as well as improve both immediate obstetric outcomes and long term health of the mother and the new born. Primary prevention of GDM, rather than treatment initiated after GDM onset is likely to lead to both economic and individual health benefits.

The PRIMORDIAL study is expected to bridge a gap in knowledge of the potential effectiveness of GDM prevention in two LMIC settings and create opportunities for capacity building in both India and The Gambia. We believe the trial can provide information that will aid in policy guidelines relating to GDM screening and management and also suggest areas that may require further research.

Contributors SKV and FK conceived and designed the study. SKV, DT, CHDF, CO, SU, AP and FK drafted the original grant proposal and trial protocol. CO and AJ provided methodological and statistical expertise. JM, SR, MJ, and AP provided expertise in the pregnancy clinical outcomes. DT, SU, CHDF and AP provided expertise in study interventions. SKV drafted the original protocol. SKV and FK drafted the manuscript. SKV, MJ, JO, MB, JM, SR have responsibilities for day-to-day running of the trial including participant recruitment, data collection and liaising with other sites. All authors critically reviewed and approved the final version of the manuscript.

Funding This study is supported by grants jointly from Medical Research Council, UK (MR/R020345/1), and Newton Fund- Director of Biotechnology, India (BT/IN/DBT-MRC/DIFD/JM/12/2018-19) The study is partly supported by the Global Challenges Research fund, University of Oxford, UK (0006138)
.

Disclaimer The views expressed in this publication are those of the author(s) and not necessarily those of the MRC, UK, GCRF, UK, DBT, India, healthcare systems or competent authorities.

Competing interests None declared.

For peer review only - http://bmjopen.bmj.com/site/about/guidelines.xhtml

Ethics approval This study will be conducted in accordance with the principles of Good Clinical Practice. The study is approved by Oxford Tropical Research Ethics Committee (OxTREC 44-18), ethics committees of Christian Medical College, Vellore (IRB 11367), Scientific co-ordinating committee, The Gambia (SCC 1645) and The Gambia Government/MRCG joint ethics committee (L2020.E15).

Provenance and peer review Not commissioned; externally peer reviewed.

REFERENCES

1. Cho NH, Shaw JE, Karuranga S, et al. IDF Diabetes Atlas: Global estimates of diabetes prevalence for 2017 and projections for 2045. *Diabetes Res Clin Pract* 2018;138:271-81. doi: 10.1016/j.diabres.2018.02.023 [published Online First: 2018/03/03]
2. Buchanan TA, Xiang AH. Gestational diabetes mellitus. *J Clin Invest* 2005;115(3):485-91. doi: 10.1172/JCI24531 [published Online First: 2005/03/15]
3. Reece EA, Leguizamon G, Wiznitzer A. Gestational diabetes: the need for a common ground. *Lancet* 2009;373(9677):1789-97. doi: 10.1016/S0140-6736(09)60515-8 [published Online First: 2009/05/26]
4. Yu Y, Arah OA, Liew Z, et al. Maternal diabetes during pregnancy and early onset of cardiovascular disease in offspring: population based cohort study with 40 years of follow-up. *BMJ* 2019;367:l6398. doi: 10.1136/bmj.l6398 [published Online First: 2019/12/06]
5. Sacks DA, Hadden DR, Maresh M, et al. Frequency of gestational diabetes mellitus at collaborating centers based on IADPSG consensus panel-recommended criteria: the Hyperglycemia and Adverse Pregnancy Outcome (HAPO) Study. *Diabetes Care* 2012;35(3):526-8. doi: 10.2337/dc11-1641 [published Online First: 2012/02/23]
6. Wahi P, Dogra V, Jandial K, et al. Prevalence of gestational diabetes mellitus (GDM) and its outcomes in Jammu region. *J Assoc Physicians India* 2011;59:227-30. [published Online First: 2011/07/16]
7. Seshiah V, Balaji V, Balaji MS, et al. Pregnancy and diabetes scenario around the world: India. *Int J Gynaecol Obstet* 2009;104 Suppl 1:S35-8. doi: 10.1016/j.ijgo.2008.11.035 [published Online First: 2009/01/22]
8. Macaulay S, Dunger DB, Norris SA. Gestational diabetes mellitus in Africa: a systematic review. *PLoS One* 2014;9(6):e97871. doi: 10.1371/journal.pone.0097871 [published Online First: 2014/06/04]
9. Mwanri AW, Kinabo J, Ramaiya K, et al. Gestational diabetes mellitus in sub-Saharan Africa: systematic review and metaregression on prevalence and risk factors. *Trop Med Int Health* 2015;20(8):983-1002. doi: 10.1111/tmi.12521 [published Online First: 2015/04/17]
10. Jiwani A, Marseille E, Lohse N, et al. Gestational diabetes mellitus: results from a survey of country prevalence and practices. *J Matern Fetal Neonatal Med* 2012;25(6):600-10. doi: 10.3109/14767058.2011.587921 [published Online First: 2011/07/19]
11. Zhu Y, Zhang C. Prevalence of Gestational Diabetes and Risk of Progression to Type 2 Diabetes: a Global Perspective. *Curr Diab Rep* 2016;16(1):7. doi: 10.1007/s11892-015-0699-x [published Online First: 2016/01/09]

For peer review only

12. Catalano PM, Tyzbir ED, Roman NM, et al. Longitudinal changes in insulin release and insulin resistance in nonobese pregnant women. *Am J Obstet Gynecol* 1991;165(6 Pt 1):1667-72. doi: 10.1016/0002-9378(91)90012-g [published Online First: 1991/12/01]
13. Catalano PM. Obesity, insulin resistance, and pregnancy outcome. *Reproduction* 2010;140(3):365-71. doi: 10.1530/REP-10-0088 [published Online First: 2010/05/12]
14. Benhalima K, Lens K, Bosteels J, et al. The Risk for Glucose Intolerance after Gestational Diabetes Mellitus since the Introduction of the IADPSG Criteria: A Systematic Review and Meta-Analysis. *J Clin Med* 2019;8(9) doi: 10.3390/jcm8091431 [published Online First: 2019/09/13]
15. Ferrara A. Increasing prevalence of gestational diabetes mellitus: a public health perspective. *Diabetes Care* 2007;30 Suppl 2:S141-6. doi: 10.2337/dc07-s206 [published Online First: 2007/07/13]
16. Group HSCR, Metzger BE, Lowe LP, et al. Hyperglycemia and adverse pregnancy outcomes. *N Engl J Med* 2008;358(19):1991-2002. doi: 10.1056/NEJMoa0707943 [published Online First: 2008/05/09]
17. Lowe LP, Metzger BE, Dyer AR, et al. Hyperglycemia and Adverse Pregnancy Outcome (HAPO) Study: associations of maternal A1C and glucose with pregnancy outcomes. *Diabetes Care* 2012;35(3):574-80. doi: 10.2337/dc11-1687 [published Online First: 2012/02/04]
18. International Association of D, Pregnancy Study Groups Consensus P, Metzger BE, et al. International association of diabetes and pregnancy study groups recommendations on the diagnosis and classification of hyperglycemia in pregnancy. *Diabetes Care* 2010;33(3):676-82. doi: 10.2337/dc09-1848 [published Online First: 2010/03/02]
19. Berger H, Sermer M. Counterpoint: Selective screening for gestational diabetes mellitus. *Diabetes Care* 2009;32(7):1352-4. doi: 10.2337/dc09-0361 [published Online First: 2009/07/01]
20. Ostlund I, Hanson U. Occurrence of gestational diabetes mellitus and the value of different screening indicators for the oral glucose tolerance test. *Acta Obstet Gynecol Scand* 2003;82(2):103-8. doi: 10.1034/j.1600-0412.2003.00001.x [published Online First: 2003/03/22]
21. Guo XY, Shu J, Fu XH, et al. Improving the effectiveness of lifestyle interventions for gestational diabetes prevention: a meta-analysis and meta-regression. *BJOG* 2019;126(3):311-20. doi: 10.1111/1471-0528.15467 [published Online First: 2018/09/15]
22. Agha-Jaffar R, Oliver N, Johnston D, et al. Gestational diabetes mellitus: does an effective prevention strategy exist? *Nat Rev Endocrinol* 2016;12(9):533-46. doi: 10.1038/nrendo.2016.88 [published Online First: 2016/06/25]
23. Thangaratinam S, Rogozinska E, Jolly K, et al. Effects of interventions in pregnancy on maternal weight and obstetric outcomes: meta-analysis of randomised evidence. *BMJ* 2012;344:e2088. doi: 10.1136/bmj.e2088 [published Online First: 2012/05/19]
24. Aune D, Norat T, Romundstad P, et al. Dairy products and the risk of type 2 diabetes: a systematic review and dose-response meta-analysis of cohort studies. *Am J Clin Nutr* 2013;98(4):1066-83. doi: 10.3945/ajcn.113.059030 [published Online First: 2013/08/16]
25. Elwood PC, Pickering JE, Givens DI, et al. The consumption of milk and dairy foods and the incidence of vascular disease and diabetes: an overview of the evidence. *Lipids*

For Peer Review Only

2010;45(10):925-39. doi: 10.1007/s11745-010-3412-5 [published Online First: 2010/04/17]
26. Grantham NM, Magliano DJ, Hodge A, et al. The association between dairy food intake and the incidence of diabetes in Australia: the Australian Diabetes Obesity and Lifestyle Study (AusDiab). *Public Health Nutr* 2013;16(2):339-45. doi: 10.1017/S1368980012001310 [published Online First: 2012/06/08]
27. Tong X, Dong JY, Wu ZW, et al. Dairy consumption and risk of type 2 diabetes mellitus: a meta-analysis of cohort studies. *Eur J Clin Nutr* 2011;65(9):1027-31. doi: 10.1038/ejcn.2011.62
28. Veiga P, Pons N, Agrawal A, et al. Changes of the human gut microbiome induced by a fermented milk product. *Sci Rep* 2014;4:6328. doi: 10.1038/srep06328 [published Online First: 2014/09/12]
29. Hill C, Guarner F, Reid G, et al. Expert consensus document. The International Scientific Association for Probiotics and Prebiotics consensus statement on the scope and appropriate use of the term probiotic. *Nat Rev Gastroenterol Hepatol* 2014;11(8):506-14. doi: 10.1038/nrgastro.2014.66 [published Online First: 2014/06/11]
30. Wen L, Duffy A. Factors Influencing the Gut Microbiota, Inflammation, and Type 2 Diabetes. *J Nutr* 2017;147(7):1468S-75S. doi: 10.3945/jn.116.240754 [published Online First: 2017/06/16]
31. Gomez-Arango LF, Barrett HL, McIntyre HD, et al. Connections Between the Gut Microbiome and Metabolic Hormones in Early Pregnancy in Overweight and Obese Women. *Diabetes* 2016;65(8):2214-23. doi: 10.2337/db16-0278 [published Online First: 2016/05/25]
32. Crusell MKW, Hansen TH, Nielsen T, et al. Gestational diabetes is associated with change in the gut microbiota composition in third trimester of pregnancy and postpartum. *Microbiome* 2018;6(1):89. doi: 10.1186/s40168-018-0472-x [published Online First: 2018/05/17]
33. Fox C, Eichelberger K. Maternal microbiome and pregnancy outcomes. *Fertil Steril* 2015;104(6):1358-63. doi: 10.1016/j.fertnstert.2015.09.037 [published Online First: 2015/10/27]
34. Ruan Y, Sun J, He J, et al. Effect of Probiotics on Glycemic Control: A Systematic Review and Meta-Analysis of Randomized, Controlled Trials. *PLoS One* 2015;10(7):e0132121. doi: 10.1371/journal.pone.0132121 [published Online First: 2015/07/15]
35. Gohir W, Ratcliffe EM, Sloboda DM. Of the bugs that shape us: maternal obesity, the gut microbiome, and long-term disease risk. *Pediatr Res* 2015;77(1-2):196-204. doi: 10.1038/pr.2014.169 [published Online First: 2014/10/15]
36. Lindsay KL, Walsh CA, Brennan L, et al. Probiotics in pregnancy and maternal outcomes: a systematic review. *J Matern Fetal Neonatal Med* 2013;26(8):772-8. doi: 10.3109/14767058.2012.755166 [published Online First: 2012/12/05]
37. Luoto R, Laitinen K, Nermes M, et al. Impact of maternal probiotic-supplemented dietary counselling on pregnancy outcome and prenatal and postnatal growth: a double-blind, placebo-controlled study. *Br J Nutr* 2010;103(12):1792-9. doi: 10.1017/S0007114509993898
38. Laitinen K, Poussa T, Isolauri E, et al. Probiotics and dietary counselling contribute to glucose regulation during and after pregnancy: a randomised controlled trial. *Br J*

For peer review only

Nutr 2009;101(11):1679-87. doi: 10.1017/S0007114508111461 [published Online First: 2008/11/20]

39. Ilmonen J, Isolauri E, Poussa T, et al. Impact of dietary counselling and probiotic intervention on maternal anthropometric measurements during and after pregnancy: a randomized placebo-controlled trial. *Clin Nutr* 2011;30(2):156-64. doi: 10.1016/j.clnu.2010.09.009 [published Online First: 2010/10/26]
40. Sahhaf Ebrahimi F, Homayouni Rad A, Mosen M, et al. Effect of L. acidophilus and B. lactis on blood glucose in women with gestational diabetes mellitus: a randomized placebo-controlled trial. *Diabetol Metab Syndr* 2019;11:75. doi: 10.1186/s13098-019-0471-5 [published Online First: 2019/09/06]
41. Asgharian H, Homayouni-Rad A, Mirghafourvand M, et al. Effect of probiotic yoghurt on plasma glucose in overweight and obese pregnant women: a randomized controlled clinical trial. *Eur J Nutr* 2019 doi: 10.1007/s00394-019-01900-1
42. Asemi Z, Samimi M, Tabassi Z, et al. Effect of daily consumption of probiotic yoghurt on insulin resistance in pregnant women: a randomized controlled trial. *Eur J Clin Nutr* 2013;67(1):71-4. doi: 10.1038/ejcn.2012.189 [published Online First: 2012/11/29]
43. Kennedy JW, Hirshman MF, Gervino EV, et al. Acute exercise induces GLUT4 translocation in skeletal muscle of normal human subjects and subjects with type 2 diabetes. *Diabetes* 1999;48(5):1192-7. doi: 10.2337/diabetes.48.5.1192 [published Online First: 1999/05/20]
44. Perseghin G, Price TB, Petersen KF, et al. Increased glucose transport-phosphorylation and muscle glycogen synthesis after exercise training in insulin-resistant subjects. *N Engl J Med* 1996;335(18):1357-62. doi: 10.1056/NEJM199610313351804 [published Online First: 1996/10/31]
45. Artal R, O'Toole M. Guidelines of the American College of Obstetricians and Gynecologists for exercise during pregnancy and the postpartum period. *Br J Sports Med* 2003;37(1):6-12; discussion 12. doi: 10.1136/bjsm.37.1.6 [published Online First: 2003/01/28]
46. Aune D, Sen A, Henriksen T, et al. Physical activity and the risk of gestational diabetes mellitus: a systematic review and dose-response meta-analysis of epidemiological studies. *Eur J Epidemiol* 2016;31(10):967-97. doi: 10.1007/s10654-016-0176-0 [published Online First: 2016/08/04]
47. Clapp JF, 3rd, Lopez B, Harcar-Sevcik R. Neonatal behavioral profile of the offspring of women who continued to exercise regularly throughout pregnancy. *Am J Obstet Gynecol* 1999;180(1 Pt 1):91-4. doi: 10.1016/s0002-9378(99)70155-9 [published Online First: 1999/01/23]
48. Nasiri-Amiri F, Sepidarkish M, Shirvani MA, et al. The effect of exercise on the prevention of gestational diabetes in obese and overweight pregnant women: a systematic review and meta-analysis. *Diabetol Metab Syndr* 2019;11:72. doi: 10.1186/s13098-019-0470-6 [published Online First: 2019/08/31]
49. Oostdam N, van Poppel MN, Wouters MG, et al. No effect of the FitFor2 exercise programme on blood glucose, insulin sensitivity, and birthweight in pregnant women who were overweight and at risk for gestational diabetes: results of a randomised controlled trial. *BJOG* 2012;119(9):1098-107. doi: 10.1111/j.1471-0528.2012.03366.x [published Online First: 2012/05/24]

For peer review only - http://bmjopen.bmj.com/site/about/guidelines.xhtml

50. Han S, Middleton P, Crowther CA. Exercise for pregnant women for preventing gestational diabetes mellitus. *Cochrane Database Syst Rev* 2012(7):CD009021. doi: 10.1002/14651858.CD009021.pub2 [published Online First: 2012/07/13]
51. Stafne SN, Salvesen KA, Romundstad PR, et al. Regular exercise during pregnancy to prevent gestational diabetes: a randomized controlled trial. *Obstet Gynecol* 2012;119(1):29-36. doi: 10.1097/AOG.0b013e3182393f86 [published Online First: 2011/12/21]
52. Vinter CA, Tanvig MH, Christensen MH, et al. Lifestyle Intervention in Danish Obese Pregnant Women With Early Gestational Diabetes Mellitus According to WHO 2013 Criteria Does Not Change Pregnancy Outcomes: Results From the LiP (Lifestyle in Pregnancy) Study. *Diabetes Care* 2018;41(10):2079-85. doi: 10.2337/dc18-0808 [published Online First: 2018/08/01]
53. Simmons D, Jelsma JG, Galjaard S, et al. Results From a European Multicenter Randomized Trial of Physical Activity and/or Healthy Eating to Reduce the Risk of Gestational Diabetes Mellitus: The DALI Lifestyle Pilot. *Diabetes Care* 2015;38(9):1650-6. doi: 10.2337/dc15-0360 [published Online First: 2015/06/27]
54. Renault KM, Norgaard K, Nilas L, et al. The Treatment of Obese Pregnant Women (TOP) study: a randomized controlled trial of the effect of physical activity intervention assessed by pedometer with or without dietary intervention in obese pregnant women. *Am J Obstet Gynecol* 2014;210(2):134 e1-9. doi: 10.1016/j.ajog.2013.09.029 [published Online First: 2013/09/26]
55. Asbee SM, Jenkins TR, Butler JR, et al. Preventing excessive weight gain during pregnancy through dietary and lifestyle counseling: a randomized controlled trial. *Obstet Gynecol* 2009;113(2 Pt 1):305-12. doi: 10.1097/AOG.0b013e318195baef [published Online First: 2009/01/22]
56. International Weight Management in Pregnancy Collaborative G. Effect of diet and physical activity based interventions in pregnancy on gestational weight gain and pregnancy outcomes: meta-analysis of individual participant data from randomised trials. *BMJ* 2017;358:j3119. doi: 10.1136/bmj.j3119 [published Online First: 2017/07/21]
57. Poston L, Bell R, Croker H, et al. Effect of a behavioural intervention in obese pregnant women (the UPBEAT study): a multicentre, randomised controlled trial. *Lancet Diabetes Endocrinol* 2015;3(10):767-77. doi: 10.1016/S2213-8587(15)00227-2 [published Online First: 2015/07/15]
58. Koivusalo SB, Rono K, Klemetti MM, et al. Gestational Diabetes Mellitus Can Be Prevented by Lifestyle Intervention: The Finnish Gestational Diabetes Prevention Study (RADIEL): A Randomized Controlled Trial. *Diabetes Care* 2016;39(1):24-30. doi: 10.2337/dc15-0511 [published Online First: 2015/08/01]
59. Shepherd E, Gomersall JC, Tieu J, et al. Combined diet and exercise interventions for preventing gestational diabetes mellitus. *Cochrane Database Syst Rev* 2017;11:CD010443. doi: 10.1002/14651858.CD010443.pub3 [published Online First: 2017/11/13]

For peer review only - http://bmjopen.bmj.com/site/about/guidelines.xhtml

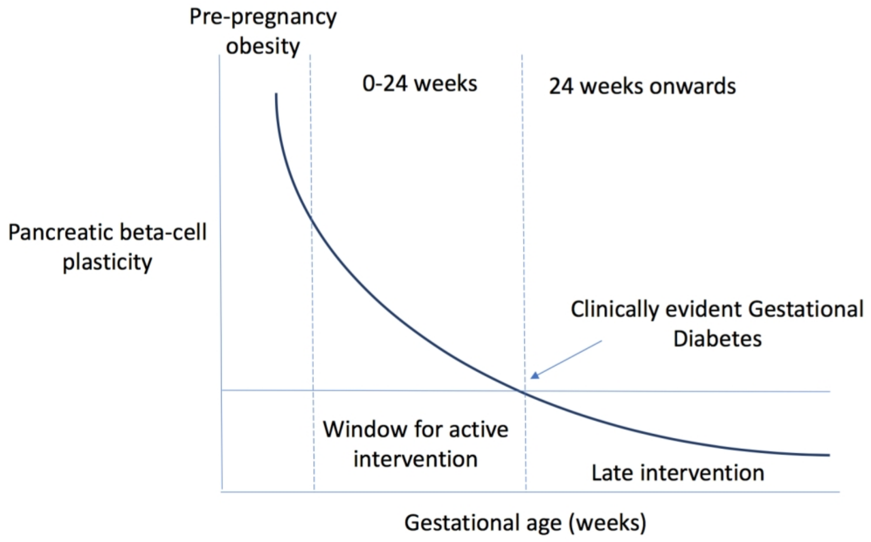

Figure 1. Relationship between pancreatic beta cell plasticity with advancement in gestational age

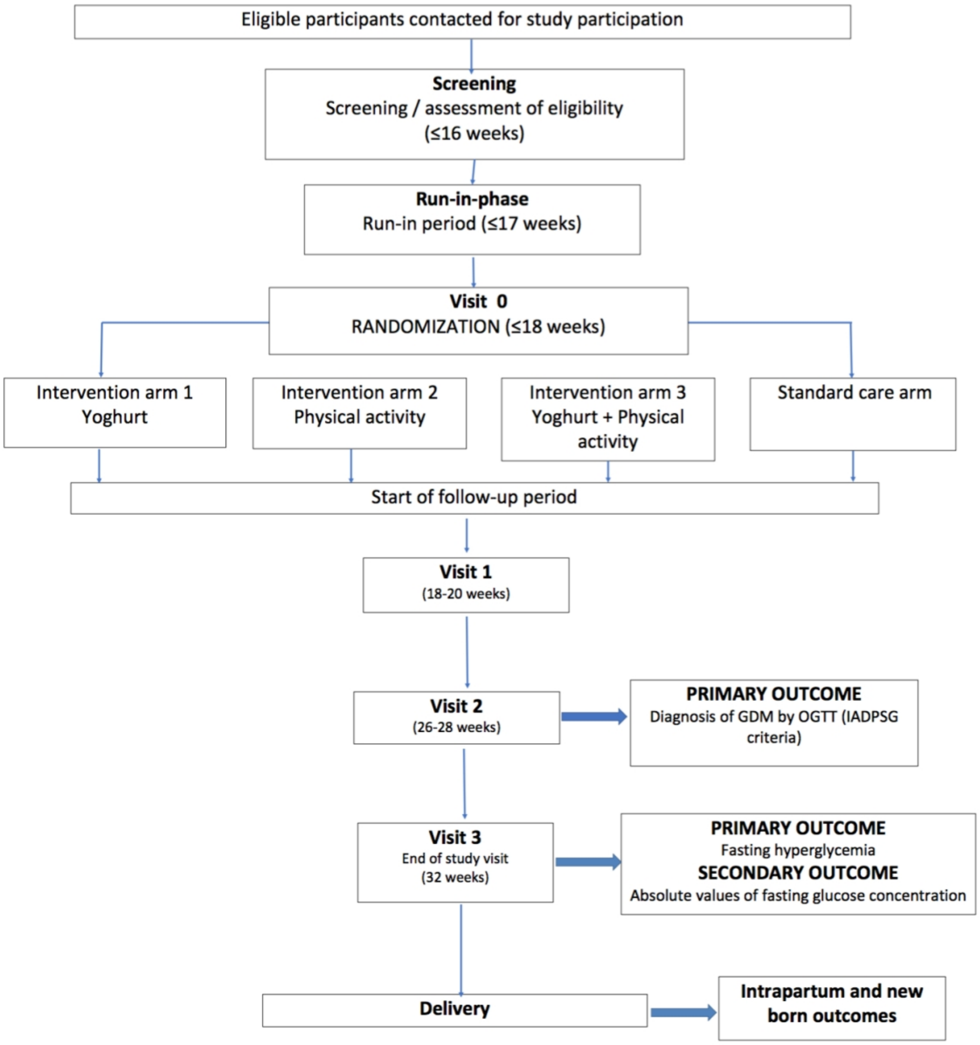

Figure 2. Flowchart of study recruitment and scheduled visits

Study Title: Pregnancy Related Interventions in Mothers at Risk for gestational Diabetes in Asian India and Low- and middle income countries

Internal Reference Number / Short title: PRIMORDIAL Study

OxTREC Ref: 44-18

Date and Version No: 18th July 2019. Version 4.2

Principal Investigator: Fredrik Karpe
Oxford Centre for Diabetes, Endocrinology and Metabolism (OCDEM)
University of Oxford
Churchill Hospital, Oxford OX3 7LE, UK
Phone: 0044 (0)1865 857222

Investigators: Andrew Prentice
MRC Unit The Gambia &
MRC International Nutrition Group,
London School of Hygiene & Tropical Medicine,
Keppel Street, London, WC1E 7HT, UK.

Jiji Elizabeth Matthews
Department of Obstetrics & Gynaecology – Unit 5
Christian Medical College & Hospital, Vellore -632004
Tamil Nadu, India

Sponsor: University of Oxford
Funder: Medical Research Council, UK

Principal Investigator Signature:

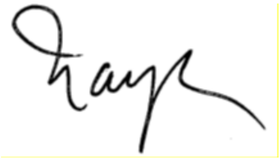

Trial Registration: ISRCTN: 18467720 and Clinical Trials Registry, India (CTRI/2018/07/014947)

Protocol written by: Senthil K Vasan and Fredrik Karpe, OCDEM, University of Oxford Churchill Hospital, Oxford OX3 7LE, UK

Conflicts of interest: None

Confidentiality Statement

This document contains confidential information that must not be disclosed to anyone other than the authorised individuals from the University of Oxford, the Investigator Team and members of the Oxford Tropical Research Ethics Committee (OxTREC), unless authorised to do so.

For peer review only

TABLE OF CONTENTS

1. SYNOPSIS 5
2. ABBREVIATIONS 7
3. BACKGROUND AND RATIONALE 9
4. OUTCOME MEASURES 17
5. STUDY DESIGN 18
6. PARTICIPANT IDENTIFICATION AND RECRUITMENT 20
6.1. Study Participants 20
6.2. Inclusion Criteria 21
6.3. Exclusion Criteria 21
7. STUDY PROCEDURES 21
7.1. Recruitment 22
7.2. Informed Consent 23
7.3. Screening and Eligibility Assessment: 23
7.4. Randomisation 24
7.5. Baseline Assessments 24
7.6. Subsequent Visits 26
7.6.1. Visit 1 (week 18-20) 26
7.6.2. Visit 2 (week 26-28) 26
7.6.3. Visit 3 (Week 32) - END OF STUDY VISIT 27
7.6.4. Post-intervention surveillance visit 27
7.7. Sample Handling 28
7.8. Discontinuation/Withdrawal 28
7.9. Lost to follow-up 30
7.10. Protocol deviation 30
7.11. Definition of End of Study 30
8. INTERVENTIONS 30
9. SAFETY REPORTING 32
9.1. Adverse event reporting 32
9.2. Definition of Serious Adverse Events 32
9.3. Reporting Procedures for Serious Adverse Events 33
10. STATISTICS AND ANALYSIS 33
10.1. Description of Statistical Methods 33
10.2. The Number of Participants 34

For peer review only

10.3. Analysis of Outcome Measures 35
11. DATA MANAGEMENT 36
11.1. Types of data 36
11.2. Format and scale of data 36
11.3. Data collection/generation 37
11.4. Data quality and standards 37
11.5. Managing, storing and curating data 37
11.6. Data preservation strategy and standards 38
11.7. Main risks to data security 38
11.8. Suitability for sharing 38
11.9. Access to Data 38
11.10. Data Handling and Record Keeping 39
12. COMMITTEES STRUCTURES 39
12.1. DATA SAFETY MONITORING COMMITTEE (DSMC) 39
12.2. Trial Steering committee (TSC) 40
12.3. Executive committee 41
13. ETHICAL AND REGULATORY CONSIDERATIONS 41
13.1. ICH-GCP 41
13.2. Approvals 42
13.3. Participant Confidentiality 42
13.4. Expenses and Benefits 42
13.5. Annual Progress Report 42
13.6. Other Ethical Considerations 42
14. FINANCE AND INSURANCE 42
14.1. Funding 42
14.2. Insurance 43
15. PUBLICATION POLICY 43
16. REFERENCES 44
18. APPENDIX A: STUDY FLOW CHART 47
19. APPENDIX B: STUDY TIMELINES (approximate) 48
20. APPENDIX C: SCHEDULE OF STUDY PROCEDURES 49
21. APPENDIX D: PATIENT INFORMATION SHEET 50
22. APPENDIX E: INFORMED CONSENT FORM 53
23. APPENDIX F: AMENDMENT HISTORY 54

For peer review only

For peer review only

1. SYNOPSIS

**Long Study Title**	**Pr**egnancy **I**nterventions **I**n **M**others **R**elating to **D**iabetes In **A**sian India and **L**ow-income countries	
**Short Study Title (to be used on participant-facing documents, if applicable)**	PRIMORDIAL Study	
**Nature of Study Participants**	Pregnant women with high-risk for developing gestational diabetes	
**Intended number of participants**	1,875 high-risk women (~937 from each centre)	
**Planned Study Period**	36 months	
	**Objectives**	**Outcome Measures**
**Primary**	To determine the efficacy of life-style interventions (yoghurt consumption and physical actvity), given to pregnant women at high risk of GDM , in reducing the incidence of GDM.	Women will be diagnosed to have GDM if one or more plasma glucose concentrations following a 75g OGTT equals or exceeds the following: fasting glucose ≥5.1 mmol/l (92 mg/dl) and/or 1h glucose ≥10.0 mmol/l (180 mg/dl) and/or 2h glucose ≥8.5 mmol/l (153 mg/dl) at 26-28 weeks gestation or if the fasting plasma glucose concentration is ≥5.1 mmol/l at 32 weeks of gestation
**Secondary**	To determine the effect of life-style intervention (yoghurt and PA), given to women at high risk of GDM , on fasting blood glucose concentrations..	Absolute concentration of fasting blood glucose at 26-28 weeks and/or at 32 weeks of gestation.
Trial and post-trial surveillance	(i) To determine the safety and efficacy of life-style intervention (yoghurt and PA), given to women at high risk of GDM, on maternal weight gain and blood pressure during pregnancy<br><br>(ii) To determine the safety and efficacy of life-style	serial weight and blood pressure measurements at all scheduled visits<br><br>**<u>Maternal outcomes</u>**<br>• Proportion of women undergoing instrumental/caesarean

For peer review only

	intervention (yoghurt and PA) on adverse maternal and new-born outcomes.<br><br>(iii) To determine social and cultural barriers to life-style changes during pregnancy	delivery (elective and emergency) captured on delivery records by treating obstetrician<br>• Post-partum haemorrhage (Primary) as captured on delivery records by treating obstetrician<br>• Pre-eclampsia and eclampsia based on blood pressure recording at each visit<br>• Blood loss at delivery as captured on delivery records by treating obstetrician<br><br>**New born outcomes**<br>• Pre-term births (<37 weeks of gestational age) calculated from the ultrasound derived gestational age captured at screening visit dating scan.<br>• Foetal macrosomia (defined as birth weight >2 SDs above the population-specific mean in each setting)<br>• Birth weight and length<br>• APGAR score at 1 and 5min of after birth<br><br>Questionnaire based assessment of social and cultural barriers.

For peer review only

2. ABBREVIATIONS

AC	Abdominal circumference
ACOG	American College of Obstetrics and Gynaecology
AE	Adverse event
AFVI	Amniotic Fluid Volume Index
BMI	Body Mass Index
BPD	Biparietal diameter
BW	Birth weight
CRF	Case Report Form
CRL	Crown-rump length
CTRG	Clinical Trials & Research Governance, University of Oxford, UK
CI	Confidence Interval
CCM	Cultural Consensus Modelling
CFU	Colony forming units
CMC	Christian Medical College, Vellore, India
CTRI	Clinical Trials Registry, India
CUREC	Central University Research Ethics Committee
CVD	Cardiovascular disease
DMC	Data Monitoring Committee
FFQ	Food Frequency Questionnaire
GA	Gestational age
GCP	Good Clinical Practice
GP	General Practitioner
GDM	Gestational Diabetes Mellitus
GWG	Gestational weight gain
HC	Head circumference
IADPSG	International Association of the Diabetes and Pregnancy Study Groups
ICF	Informed Consent Form
IDEA	Impact of Diet and Exercise Activity on Pregnancy Outcomes study
IR	Insulin Resistance
ISRCTN	International Standard Randomised Controlled Trial Number
IUGR	Intrauterine growth restriction
LGA	Large for gestational age

For peer review only

LIMIT	Antenatal lifestyle advices to improve pregnancy outcomes study
LiP	Lifestyle interventions in Pregnancy study
LMICs	Low and Middle Income Countries
LSHTM	London School of Hygiene and Tropical Medicine, UK
MRCG	Medical Research Council, The Gambia at LSHTM
MRC, UK	Medical Research Council, United Kingdom
MS IDREC	Medical Sciences Interdivisional Research Ethics Committee, Oxford, UK
OGTT	Oral Glucose Tolerance Test
PA	Physical Activity
PAMELA	Physical activity during pregnancy and maternal-child health study
PCOD	Polycystic ovarian disease
PI	Principal Investigator
PIS	Participant Information Sheet
PID	Personally identifiable data
PI_x	Pulsatility Index
PPAQ	Pregnancy-related Physical Activity Questionnaire
PRIMORDIAL	Pregnancy Interventions In Mothers Relating to Diabetes In Asian India and Low-income countries
RCT	Randomized Controlled Trial
REC	Research Ethics Committee
RR	Risk ratio
SD	Standard deviation
SOP	Standard Operating Procedure
SAE	Serious Adverse Event
SGA	Small for Gestational Age
S/D ratio	Systolic/diastolic ratio
TOP	Treatment of Obese Pregnant Women study
T2D	Type 2 Diabetes Mellitus
UPBEAT	UK Pregnancies Better Eating and Activity Trial
WHO	World Health Organization

For peer review only

3. BACKGROUND AND RATIONALE

Gestational diabetes mellitus (GDM) is associated with significant intrapartum complications, perinatal morbidity, and long-term risk of developing T2D, obesity and CVD in both the mother and the offspring (1, 2). Normally, hyperglycaemia rapidly abates following delivery. However, about 50-70% of GDM mothers develop T2D within 5-10 years postpartum (3) suggesting that GDM may be a prodrome of “common T2D”. The key underlying pathophysiology is the loss of β-cell plasticity to adapt to the progressive insulin resistance (IR). GDM risk can be reduced at two levels: i) by reducing IR secondary to obesity and ii) by improving glucose disposal. Recently, alterations in the gut microbiota have been linked to obesity, IR and T2D (4, 5). The gut microbiota composition changes over the course of gestation (6) and this is further exacerbated on the background of maternal IR and adiposity. Alteration of gut microbial composition through dietary changes and physical activity exert beneficial effects in terms of weight gain, blood glucose levels, reduced IR and T2D risk (7, 8). Thus, simple interventions that aim at altering the gut microbiota by life-style modification [diet and physical activity (PA)] and thus improving glucose disposal constitute potentially attractive options to reduce the risk of GDM.

Global burden of GDM GDM affects 1-14% of all pregnancies worldwide (9) and the risk of GDM reflects the underlying T2D frequency in the community (10). The prevalence is increased to 17.8% (range 9.3**–**25.5%) with the recent introduction of IADPSG criteria (11). The risk of GDM in specific ethnic groups particularly Asians and Africans is increased compared with Caucasians and thus contributes substantially to future T2D in these communities (12, 13). The incidence of GDM in LMICs is largely unknown and is mostly based on single-centre based statistics. In India, the reported prevalence varies between 17-19% (14). A recent systematic review of GDM prevalence in six African countries (mostly based on 2-step Carpenter Coustan’s or WHO criteria), report a prevalence equating to 11% (15). This may not be a true representation of the African continent and there is a relative absence of reliable prevalence estimates of GDM in West Africa.

Window for intervention ‘High-risk’ GDM mothers include pregnant women with chronic IR states such as overweight or obesity, rapid gestational weight gain (GWG), history of a polycystic ovarian disease (PCOD), impaired glucose tolerance prior to pregnancy, previous GDM and advanced maternal age. Other recognised risk factors include having a first-degree relative with T2D, previous history of stillbirth/miscarriage, a large baby or pre-eclampsia. Intense glycaemic management is usually reserved for mothers diagnosed with GDM, typically late in pregnancy (24-26 weeks), when glucose intolerance is apparent and almost 60% of β-cell plasticity is lost (16) (Figure 1). The “critical window” for any intervention should therefore ideally start earlier than 24-26 weeks when the pancreatic β-cells are still able to compensate with the maternal IR. The RADIEL study showed a 39% risk reduction in GDM incidence among high-risk pregnant women with diet and

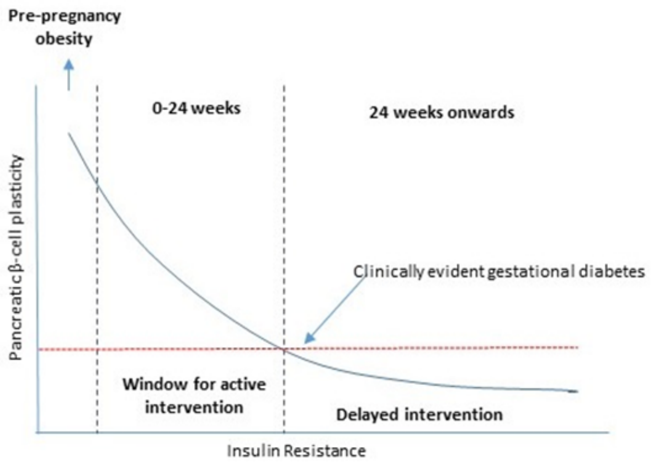

For peer review only - http://bmjopen.bmj.com/site/about/guidelines.xhtml

lifestyle intervention beginning from 20 weeks of gestation (17).

Dietary interventions Fermented dairy products (yoghurt and cultured sour milk) can alter the 'microbiotal-signature' and lower the gut-induced pro-inflammatory response (18). Recent meta-analyses have shown that daily yogurt consumption reduced risk of T2D by 14-18% compared to other dairy products (19, 20). Meta-analysis of probiotic use in late pregnancy has been shown to be safe (41). A summary of studies evaluating the beneficial effects of yoghurt in T2D prevention is shown in page 12.

Physical activity (PA) in pregnancy PA has well-documented health benefits and reduces peripheral insulin resistance in non-pregnant individuals. It is likely that the mechanisms responsible for alterations in insulin sensitivity and glucose uptake in response to exercise in non-pregnant populations may apply to pregnancy. PA is considered safe during pregnancy and substantially reduces the incidence of GDM by reducing IR (21, 22). RCTs and meta-analyses have shown benefits in terms of GWG, delivery type, preterm delivery rate and neonatal outcomes (23, 24). Interventional studies that used aerobic exercises or strength training initiated as early as 9-12 weeks and continued until 38 weeks have clearly shown benefits on glycaemic control, GWG and pregnancy outcomes (25, 26). The ACOG recommend 20-30 minutes of moderate-intensity aerobic activity or strength training at least 3-5 days/week during pregnancy (27). PA has also been shown to alter gut microbiota (28) and a differential microbiota pattern is observed between active vs. sedentary women, implying the role of PA in mediating glucose homeostasis by alteration of gut microbiota.

Hypothesis

Daily yoghurt and/or daily walking for at least 14 weeks will reduce the risk of developing gestational diabetes in 'high-risk' pregnant women

Benefits for the study population and the population whom the results of the project might be generalised to

Benefits to the participants: The current study will sensitise and expose women to specific healthy lifestyles during pregnancy. The opportunity to bring about behavioural changes in the early pregnant woman by changing maternal nutrition and increasing physical activity to prevent GDM can have immediate and lifelong health impact on the mother as well as the new born with possible positive consequences into adulthood. Lifestyle interventions during pregnancy are proven to have beneficial effects on GWG and improved neonatal outcomes. By demonstrating that maternal lifestyle changes beginning from early pregnancy using simple, realistic and culturally acceptable methods will have a beneficial impact in immediate and long term maternal and child health could potentially help reduce the overwhelming global burden of non-communicable diseases, particularly diabetes.

Benefits to the community: Our study aims at providing evidence to support political action and policy making at national and global level in low and middle-income countries (LMICs). This research aims at strengthening the existing antenatal services in The Gambia and the research facilities in India, where such resources are clearly lacking. In line with the research call, the

For peer review only

study will enable opportunities for capacity building in both India and the Gambia. The project will also create opportunities to specifically train young Gambian health providers (obstetricians, midwives, diabetes educators) to travel to India (which is a tertiary medical care facility and well known for its teaching excellence) to be trained in various aspects of antenatal care and high-risk management. In turn, the health researchers in India will clearly benefit from the support of MRC-Gambia in infrastructural set up of a data management facility.

For peer review only

Summary of findings from previous studies

Studies investigating the effect of consuming conventional or probiotic yoghurt during pregnancy

Study reference	Type of study	Target population	Intervention	Findings
Lindsay et al. 2013 (Ref 29)	Systematic review	7 studies including 3 clinical trials using probiotics in pregnancy	-	Impact of probiotic intervention on GDM is better among high-risk mothers
Laitinen et al. 2009 (Ref 30)	Double-blind RCT	Pregnant women <17 weeks (n=256)	Diet + Probiotic<br>Diet + Placebo<br>Control + placebo	Reduced risk of elevated glucose in probiotic group (OR 0.31, 95%CI 0.12-0.18; p=0.013)
Luoto et al. 2010 (Ref 31)	Double-blind RCT	Pregnant women <17 weeks (n=256)	Diet + Probiotic<br>Diet + Placebo<br>Control + placebo	Reduction in GDM risk by 13% in probiotic group
Zheng et al. 2018 (Ref 32)	Meta-analysis	Studies investigating probiotics in pregnancy		Probiotic supplementation during pregnancy is negatively associated with fasting insulin (OR -2.94, 95%CI [-5.69, -0.20], p = 0.04) and HOMA-IR (OR -0.65, 95%CI [-1.18, -0.11], p = 0.02)
Hajifaraji et al. 2018 (Ref 33)	Double-blind RCT	Pregnant women with GDM (n=64)	Probiotic vs placebo for 8 weeks	Improved inflammatory and oxidative stress biomarkers in probiotic group
Nilert et al. 2013 (SPRING Trial) (Ref 34)	Double-blind RCT	Pregnant women <16 weeks with BMI >25kg/m^2 (n=540)	Probiotic vs placebo capsules from 16 weeks to delivery	Ongoing

For peer review only - http://bmjopen.bmj.com/site/about/guidelines.xhtml

Studies investigating the effect of PA during pregnancy

Study reference	Type of study	Target population	Intervention	Findings
Russo et al. 2015 (Ref 21)	Meta-analysis	10/469 studies (Total 3,401 GDM/high risk pregnant mothers, control healthy pregnant women)	-	The PA intervention arm showed a 28% risk reduction with GDM incidence compared with standard care arm.
Barakat et al. 2013 (Ref 35)	RCT	510 healthy gravida to either an exercise intervention or a usual care (control) group (n=255 each)	Moderate-intensity resistance and aerobic exercises (three times/week, 50–55 min/session) beginning from 2nd trimester	No reduction in GDM risk between groups. Other outcomes: 58% reduction macrosomia, 34% reducti in elective caesarean delivery and 12% reductic in maternal weight gain.
Barakat et al. 2011 (Ref 36)	RCT	Exercise programme with instructor (three sessions per week, n=40) vs non – exercise group (n=43)	25 min core session + 7-8 min gradual warm up and cool down beginning from 6-9 weeks to 38-39 weeks + one set of aerobic dance every 2 weeks for the intervention arm	Moderate PA during pregnancy improved maternal glucose toleran
Poston et al. 2015 UK Pregnancies Better Eating and Activity (UPBEAT) trial. (Ref 37)	RCT	1,555 women with a BMI ≥ 30 kg/m2 between 15-18 weeks +	Behavioural intervention (n=772, healthy eating with low glycaemic load foods + 8 sessions with the health trainer on a weekly basis, each lasting 1 to 1.5 hours) vs standard care (n=783)	No difference in risk of GI or incidence of LGA babie

© Copyright: The University of Oxford 2019
For peer review only - http://bmjopen.bmj.com/site/about/guidelines.xhtml

Da Silva et al. 2017 (Ref 38) Physical activity during pregnancy and maternal-child health (PAMELA) Study.	RCT	Healthy pregnant women (n=639)	Exercise intervention thrice weekly for 16 weeks (from 16-20 to 32-36 GA)	No differences between t two groups in mean GWG GDM, BW, infant length, and head circumference.
Haakstad et al. 2011 (Ref 39)	RCT	Nulliparous, sedentary healthy pregnant women (n=105)	12-week supervised exercise programme (60 min supervised aerobic dance = strength training for 60min at least twice weekly)	Mean weight gain during pregnancy was lower in exercise compared to control group (11.0 +2.3 v 13.8 +3.8 kg, p 50.01)
Wang et al. 2017 (Ref 40)	RCT	Singleton pregnant women with BMI between 24 to 28 kg/m2 at 12+6 weeks of GA until 37 weeks	Exercise group vs controls Exercise 3 times per week (at least 30 min/session with a rating of perceived exertion between 12-14) via a cycling program at least 3 times/week	Lower GDM incidence in exercise vs control arm (22% vs 40.6%, p<0.001). Reduced GWG, IR, rates c caesarean delivery, fetal macrosomia and preterm births in exercise group

Studies investigating the effect of diet + PA during pregnancy

Study reference	Type of study	Target population	Intervention	Findings
Vinter et al. 2011 (Ref 41) Lifestyle in Pregnancy (LiP) study.	RCT	360 obese (pre-pregnant BMI 30-45 kg/m^2) pregnant women	Intervention program included dietary guidance + moderate PA for 30-60 min daily + closed training sessions with instructor for 1 h each week	Intervention group had a significantly lower median (range) GWG compared with the contro group of 7.0 (4.7–10.6) vs. 8.6 kg (5.7–11.5; P = 0.01)

For peer review only

© Copyright: The University of Oxford 2019
For peer review only - http://bmjopen.bmj.com/site/about/guidelines.xhtml

Renault et al. 2014 (Ref 42) Treatment of Obese Pregnant Women (TOP) study.	RCT	425 obese pregnant women comparing 3 groups	PA + Diet, (n = 142)<br>PA intervention (daily step count of 11,000 steps/day every day for 4 weeks (n = 142)<br>Control group receiving standard care (n = 141)	PA intervention decreasec GWG by a mean of 1.38 kg (P =0 .040).
Koivusalo et al 2016. (Ref 43)<br>The Finnish Gestational Diabetes Prevention Study (RADIEL study).	RCT	293 pregnant women with a history of GDM and/or a pre-pregnancy BMI of ≥30 kg/m2 at <20 weeks gestation	Intervention group received individualized counselling on diet, physical activity, and weight control. control group received standard antenatal care	GDM incidence of was 13.9% in the intervention group and 21.6% in the control group ([95% CI 0.4 0.98%]; P = 0.044.<br>GWG was significantly lower in the intervention group (p=0.037)
Dodd et al. 2014<br>(Ref 44, 45)<br>Limiting weight gain in overweight and obese women during pregnancy (LIMIT) trial	RCT	2,152 singleton pregnant mothers between 10-20 weeks GA and BMI ≥25kg/m^2	Diet and lifestyle advice group (n=1,075) vs standard care (n=1067)	Risk of LGA babies were similar in both groups Babies of life-style advice group had higher mid-thig fat mass measured by US and rate of adipose tissue deposition was slow in thi group compared with control group.

© Copyright: The University of Oxford 2019
For peer review only - http://bmjopen.bmj.com/site/about/guidelines.xhtml

Bennet et al. 2018 (Ref 46)	Systematic review and meta-analysis	45 RCTs	-	Diet and PA interventions aimed to reduce GWG wa[illegible] associated with reduced GDM risk by 44% and 38[illegible] respectively. Among Asia[illegible] the risk reduction with die[illegible] and life-style 62% and 32[illegible] respectively
Shepherd et al. 2017 (Ref 47)	Cochrane Database Systematic Review	23 RCTs and cluster-RCTs, comparing combined diet and exercise interventions with no intervention (i.e. standard care), that reported on GDM diagnosis as an outcome	-	Reduced GDM risk in intervention group compared with the standard care group (average risk ratio (RR) 0.[illegible] 95% CI 0.71 to 1.01. Reduced risk of caesarean section (RR 0.95, 95% CI 0.88 to 1.02) in interventi[illegible] arm
Petrella et al. 2014 (Ref 48)	Case-control study	Singleton pregnant women with BMI >25 kg/m^2	Therapeutic lifestyle changes (diet 1800kcal/day + mild PA 30min/d thrice weekly) (n=33) vs Controls (n=28)	Lower GWG, lower incidence of GDM, hypertensive disorders an[illegible] preterm births in the intervention group compared to controls.

For peer review only

For peer review only - http://bmjopen.bmj.com/site/about/guidelines.xhtml
© Copyright: The University of Oxford 2019

4. OUTCOME MEASURES

Outcomes	Measurements	Timepoint(s) of evaluation of this outcome measure (if applicable)
**Primary objective**		
Incidence of GDM	Fasting plasma glucose<br>Glucose at 1 hour and 2 hours' post 75g OGTT (according to IADPSG criteria) or Fasting hyperglycemia measured by fasting glucose	Between weeks 26-28<br><br>At 32 weeks
**Secondary objective**		
Effect of intervention on fasting blood glucose concentration	Fasting plasma glucose	Between weeks 26-28 and at<br>32 weeks of gestation
**Trial and post-trial surveillance measures**		
Gestational weight gain	Maternal weight	At screening, run-in-phase, randomization, visits 1,2,3 and<br>at delivery (before the birth of the child)
Gestational hypertension	Maternal blood pressure	At screening, run-in-phase, randomization, visits 1,2,3 and<br>at delivery (before the birth of the child)
Proportion of women undergoing Instrumental/caesarean delivery	Instrumental/caesarean delivery (elective and emergency)	At delivery
Post-partum haemorrhage (primary)	Amount of blood loss within 24 hours of child birth	From delivery to 24 hours
Blood loss at delivery	Amount of blood lost during delivery	At delivery
Pre-eclampsia and eclampsia	Clinical diagnosis (hypertension + proteinuria +/- seizures)	Clinical diagnosis after 28 weeks of GA
Pre-term births (<37 weeks of gestational age)	Gestational age calculated from dating scan	At birth of the new born
Foetal macrosomia	Birth weight (defined as birth weight >2 SDs above the population-specific mean in each setting)	At birth of the new born
Birth weight	Birth weight	At birth of the new born
APGAR score at 1 and 5 min of birth	Clinically recorded APGAR score	At birth of the new born

For peer review only - http://bmjopen.bmj.com/site/about/guidelines.xhtml

Length of the new born	Length measured from head to the heel	Within 48 hours of birth
Barriers to interventions in pregnancy 1 & 2	Questionnaire based assessment	At screening<br>At 32 weeks

5. STUDY DESIGN

Design: 2 x 2 factorial design, multi-centric, open labelled, randomized controlled trial
Total study duration: 36 months
Number of scheduled visits: 5

(i) **Screening visit (visit -2, ≤16 weeks GA):** Singleton pregnant women willing to participate in the study will be assessed for eligibility based on inclusion/exclusion criteria and eligible participants will be asked to sign an informed consent if willing to participate. Eligible women will undergo an oral glucose tolerance test (OGTT) to rule out impaired glucose intolerance or diabetes at screening and will also undergo dating ultrasound scan. Routine antenatal examination will be done. Barriers to life-style interventions-1 during pregnancy will be assessed using a questionnaire.

(ii) **Run-in visit (visit -1, ≤17 weeks GA)**: Baseline assessments of dairy product consumption will be done using standardized questionnaire . Additionally, objective measurements of baseline PA activity over 7 days will be assessed in all study participants (with the result blinded to participants) using a wearable accelerometer device (Garmin Vivofit 4 fitness band).

(iii) **Randomization (≤18 weeks GA):** Women will be randomized to either (i) diet (consumption of 200g of yoghurt per day), (ii) PA (a minimum of 40% increase above the recorded baseline PA) (iii) diet and PA or (iv) standard care. Active intervention will be from randomization to 32 weeks of GA.

(iv) **Visit 1 (18-20 weeks GA)**: maternal weight, blood pressure, routine antenatal examination and foetal morphology scan.

(v) **Visit 2 (26-28 weeks GA)**: maternal weight, blood pressure, routine antenatal examination and OGTT. Primary endpoint (diagnosis of GDM based on IADPSG critria) will be assessed at this visit. Women with GDM will continue on allocated study intervention until end of study (week 32) in addition to the clinical managemnt provided as per the local obstetric practice.

(vi) **Visit 3 (32 weeks GA, End of Study visit)**: maternal weight, blood pressure, routine antenatal examination, ultrasound foetal scan and fasting blood glucose and barriers to pregnancy intervention-2 assessment. (A repeat ultrasound scan at this stage is based on the evidence that growth restriction although starts in early pregnancy, is more marked between 22-34 weeks and also that foetal measurements do not have

For peer review only

exact equivalents to new born measurements. Therefore, a last trimester scan is essential as it reflects foetal growth more precisely).

(vii) **Delivery visit:** This is a post-trial surveillance visit primarily intended to assess the safety and efficacy of interventions on pregnancy and new-born outcomes.

Aside from the scheduled study visits, participants will be contacted on a weekly basis either over telephone or visited at home by research personnel. This will be made to ensure general well-being, compliance (wearing device, adhering to individually advised increment in step count, reminders of study visits, yoghurt consumption etc) and motivation.

Interventions

Dietary intervention Each participant in the diet and diet+PA arm will be advised to consume yoghurt 200 g/day once daily prepared locally at the dietary department in CMC, Vellore and from a local production in The Gambia. The yoghurt consumption is independent of the timing of their regular meal time (can be consumed anytime). Record of daily yoghurt and background dairy consumption will be maintained by every participant. The yoghurt will be prepared on a common recipe with the same starter culture at both centres. The yoghurt will be dispensed daily/weekly as feasible by the study centre. Adherence to yoghurt consumption will be monitored on a weekly basis by home visiting field staff. Dietary counselling will be reinforced in all participants and the non-diet arm will not be advised to stop their regular dairy consumption, assuming that any effect on outcome is related to the trial yoghurt consumed.

Physical activity intervention Women randomized to the PA and diet+PA arm will be provided PA targets to be followed throughout pregnancy based on their baseline PA assessment which was obtained at run-in-phase. The recommendation will be set based on a minimum of a 40% increase of the baseline step count measured during run-in-phase (refer to section 8.1.2 for details). The daily PA activity will be monitored using an accelerometer device (Garmin Vivofit 4 fitness band) which will provide visual feed back to the participants in the active PA arms in order to motivate participants and help them achieve their daily target step count. All other (yoghurt and standard arm) will also be provided with fitness bands to objectively measure their routine PA behavior throughout the study. However, step counts will be blinded for these participants. Data from these devices will be periodically uploaded using vivohub 2 for compliance monitoring and downloaded to provide visual feedback and counselling to participants.

Potential risks and benefits

Risks with study-related procedures

Risks with venepuncture may include bruising in the area of venipuncture, dizziness, nausea, vasovagal syncope. Consumption of oral glucose solution on an empty stomach may cause nausea, vomiting, headache. Ultrasound scans during pregnancy are not considered harmful. Utmost care would be taken to minimize such risks. A trained phlebotomist or research nurse will draw blood samples for the study. We will use clean, sterile phlebotomy equipment. Blood will be drawn using vacutainers with women sitting or lying down, whichever is comfortable. Local pressure will be applied for 20-30 seconds to avoid post-puncture bleeding.

For peer review only

Risks with interventions
There are no major serious adverse effects directly linked to the proposed interventions in the study. Both yoghurt and PA are generally considered safe during pregnancy. However, some potential adverse effects of the proposed interventions can be anticipated and are not necessarily caused by or related to the intervention. In the yoghurt consumption arm, anticipated risks may include nausea, vomiting and intolerance to yoghurt. In women undertaking physical activity, common adverse effects may include dizziness, headache, exertional dyspnoea, easy fatigability and muscle aches. The proposed PA in our study meet the safety guidelines for physical activity recommended by the ACOG (27). Safety will be ensured by excluding women who have absolute contraindications to aerobic physical activity during screening. Potential risks of PA may include persistent vaginal spotting/bleeding, calf pain/swelling (diagnosed to be related to thrombophlebitis or deep vein thrombosis), dyspnoea at rest, painful premature contractions, decreased foetal movements, preterm labour and preterm/premature rupture of membranes. A participant will be withdrawn from the study, in the event of persistent adverse events reported by the participant, and the PI feels that the event may be related to the intervention.

Data collection process

Data type	Method of collection	
	Gambia	Vellore
Weight (of the mother)	Digital Tanita scale	Micro weight electronics. Model: DS415 series
Height (of the mother)	Leicester height measure, seca 214	Stadiometer
Blood pressure	Electronic BP apparatus (Omron Corporation, Tokyo, Japan)	Electronic BP apparatus (Omron Corporation, Tokyo, Japan)
Baseline diet consumption	Dairy consumption questionnaire	Dairy consumption questionnaire
Barriers assessment	Questionnaire	Questionnaire
PA activity monitoring device	Garmin Vivofit 4 fitness band	Garmin Vivofit 4 fitness band
Height (of the baby)	Infantometer	Infantometer
Ultrasound	Aloka USI-145	Voluson GE S-8
Biochemistry		
Blood glucose	Roche Cobas 800<br>Enzymic (autoanalyser)	Roche Cobas 800<br>Enzymic (autoanalyser)

6. PARTICIPANT IDENTIFICATION AND RECRUITMENT

6.1. Study Participants
The study will include healthy pregnant women who have at least one “risk-factor” for developing gestational diabetes (see section 6.2 for high-risk definition).

For peer review only

6.2. Inclusion Criteria

- Pregnant women, aged ≥18 years
- Pregnant women ≤16 weeks of gestational age
- In addition to the above, pregnant women should meet at least one of the following criteria for high-risk GDM:
  - Booking BMI ≥25kg/m^2
  - Age ≥25 years
  - First-degree relative with diabetes
  - Previous pregnancy with GDM
  - Previous gestation with large baby (≥3.5kg)
  - Previous pregnancy with pre-eclampsia/eclampsia
  - History of PCOD/impaired fasting glucose
- Not currently on any medications (except iron, calcium or folic acid supplements, thyroxine supplement for hypothyroidism, low dose aspirin for pre-eclampsia)*
  *women conceived by ovulation induction using clomiphene citrate or metformin for PCOD will not be excluded from the study.

6.3. Exclusion Criteria

The participant may not enter the study if ANY of the following apply:

- GDM diagnosed prior to screening visit based on IADPSG criteria or documented raised HbA1C, i.e., either fasting glucose ≥5.1 mmol/L or 1h glucose ≥10.0 mmol/L or 2h glucose ≥8.5 mmol/L, or a documented HbA1c of ≥6.5% at first booking
- History of pre-gestational diabetes
- Multiple gestation in current pregnancy
- History of severe hyperemesis in the first trimester
- Uncontrolled pre-gestational or gestational hypertension (BP>150/100mm Hg) or on treatment
- History of recurrent (more than two) first trimester spontaneous abortions or stillbirths
- History of significant ante- or post-partum hemorrhage in the previous pregnancy
- Previous child born with congenital anomalies
- Pregnancy following in-vitro fertilization or any assisted reproductive technology and those unwilling to adhere to the study protocol will be excluded from the study
- Previous or current psychiatric illness on medication
- Previous or current neurological condition (e.g. epilepsy) or on medication
- Women meeting absolute contraindications for physical activity during pregnancy as recommended by the ACOG (heart disease, restrictive lung disease, incompetent cervix/cerclage, pregnancies at risk for premature labour, gestational hypertension, severe anaemia)
- Physical disability to PA and/or known lactose intolerance

7. STUDY PROCEDURES

The schedule of procedures at each visit is provided below

For peer review only

Visit	Study procedures
**Screening visit (≤16 weeks) (Visit -2)**	Height<br>Weight<br>Blood pressure (mother)<br>Antenatal examination (fundal height)<br>OGTT<br>Dating Ultrasound scan<br>Barriers to pregnancy intervention assessment-1
**Run-in-phase (≤17 weeks) (visit -1)**	Weight<br>Blood pressure<br>Antenatal examination (fundal height)<br>Questionnaire based assessment of background dairy consumption<br>Diet and PA counselling<br>Dispensing wearable activity monitors device for assessment of baseline PA for 7-days and compliance testing.
**Randomization (≤18 weeks) (visit 0)**	Weight<br>Blood pressure<br>Routine antenatal examination (fundal height)<br>Randomization
**Visit 1 (week 18-20)**	Weight<br>Blood pressure<br>Routine antenatal examination (fundal height)<br>Morphology scan
**Visit 2 (26-28 weeks)**	Weight<br>Blood pressure<br>Routine antenatal examination (fundal height)<br>OGTT
**Visit 3 (32 weeks) End of Study visit**	Weight<br>Blood pressure<br>Routine antenatal examination (fundal height)<br>Fasting plasma glucose<br>Ultrasound scan (to monitor foetal growth)<br>Barriers to pregnancy interventions assessment-2 (for future studies)
**Delivery (post-trial surveillance)**	Weight (mother)<br>Blood pressure (mother)<br>Intrapartum outcomes<br>Neonatal outcomes (Birth length, Birth weight, APGAR score)
**Telephonic contacts / Home visits**	General well-being<br>Compliance with interventions (step count from accelerometer, yoghurt pot-count)

7.1. Recruitment

In both India and The Gambia, women will be identified through home-visits by field workers and in the out-patient clinics of recruiting hospitals operated through the MRC Unit. In The

For peer review only

Gambia, the investigators will use additional strategies such as (i) community sensitization through village meetings that includes the community leaders, ward heads and their development committees, traditional birth attendants (TBAs) and community health workers (ii) house-to-house campaigns led by field workers and TBAs to encourage newly pregnant women to attend antenatal clinics (iii) Frequent use of the local radio stations for general community sensitisation and (iv) referrals from maternal and child health staff and obstetric radiologists to enhance participant identification and recruitment. In India, CMC, Vellore, recruitment will also be made from referrals from several community hospitals and peripheral health centres such as Community Health and Development Hospital (CHAD), Low-cost Effective Care Unit (LCECU), Rural Unit for Health and Social Affairs (RUSHA) and Community Health Nursing (CONCH) by engaging the rural community in grass root health and social programmes. Participants will be recruited by word of mouth.

7.2. Informed Consent

Written and verbal versions of the Participant Information sheet (PIS) and Informed Consent form (ICF) will be presented to the participants detailing no less than: the exact nature of the study; what it will involve for the participant; the implications and constraints of the protocol; the known side effects and any risks involved in taking part. It will be clearly stated that the participant is free to withdraw from the study at any time for any reason, and with no obligation to give the reason for withdrawal. As a part of consenting women will be informed that withdrawing consent from the study will not prejudice their medical care in any way.
The participant would be allowed as much time as wished to consider the information, and the opportunity to question the personnel administering the ICF (physician, medical research officer/midwife/field worker who are authorised to do so by the PI). The participant would provide his willingness to take part in the study by signing the ICF. In the event that the participant in uneducated, her left thumb impression would be obtained along with the signature of the neutral person who will serve as an impartial witness. A copy of the signed ICF would be given to the participant. The original signed form will be retained at the study site.

7.3. Screening and Eligibility Assessment:

Pregnant women aged ≥18 years and ≤16 weeks of gestation who are willing to participate in the study will be screened and assessed for eligibility.

In addition to above, participants should meet at least one of the below mentioned criteria to be termed as “high-risk” for inclusion into the study

- Booking BMI ≥25kg/m^2
- Age ≥25 years
- First-degree relative with diabetes
- Previous pregnancy with gestational diabetes mellitus
- Previous pregnancy with large baby (≥3.5kg)
- Previous pregnancy with pre-eclampsia/eclampsia
- History of PCOD/impaired fasting glucose

Women will then undergo a dating scan to confirm the GA and an OGTT (as described in section 7. Study procedures). Women with blood glucose values at fasting, 1 hour and 2 hours within

For peer review only

normal limits will move forward to the run-in-phase (visit -1) and others will be treated as "screen failure"

7.4. Randomisation

Eligible women will be randomly allocated to one of the following arms (i) Yoghurt (ii) PA (iii) Yoghurt + PA or to the (iv) control group (neither intervention, standard care) following an allocation schedule prestratified for study centre, age and BMI generated by an independent statistician not involved in the study. A variable block randomisation, stratified by centre, and then by age, (<25 and ≥25 years) and BMI (<25 and ≥25 kg/m2) will be generated, using a password-protected Access database developed by an independent statistician who is not involved with trial participants or data analysis. A unique study ID will be assigned to each participant at screening by the research officer.

In India, the random allocation of each participant to one of the four intervention groups, stratified by age*BMI will be performed using a computer interface (Access database with forms and data separated into a frontend and password backend, respectively).

In Gambia, due to logistical limitations, separate lists will be generated for each strata combination (age x BMI) and randomisation assigned using opaque, sealed envelopes. As this is an open-labelled study, study participants, and investigators will not be blinded to the treatment arm. Laboratory personnel and the trial statistician will be blinded.

7.5. Baseline Assessments

Baseline assessments are divided over first three visits (screening, run-in-visit and randomization)

7.5.1. Baseline assessments at screening visit (Visit: -2; ≤16 weeks)

I. Eligibility check
II. **Height:** Height of the pregnant mother will be measured using a portable or fixed stadiometer.
III. **Weight:** Weight of the pregnant mother will be measured using a calibrated electronic weighing scale to the nearest 0.1kg.
IV. **Blood pressure**: Systolic and diastolic blood pressure will be measured after 5 minutes seated at rest. It will be measured three times using an automated validated device (Omron M3, Tokyo, Japan), with an appropriately sized cuff. Three measurements will be recorded in a seated position by a trained research staff. The average of three values will be taken for the final analysis.
V. **Antenatal examination (fundal height):** Routine antenatal examination will be carried out according to local obstetric practice.
VI. **Dating ultrasound scan:** The dating scan will be done at screening visit in all participants meeting the eligibility criteria. The following variables will be measured and recorded: Type of gestation –single/twins/triplets, gestational sac, crown-rump length, yolk sac, cardiac activity, gestational age by scan, scan expected date of confinement. Women diagnosed with multiple gestation on scan, gestational week corresponding to more than

For peer review only

1 week greater than the scheduled visit date and absent in-utero gestational sac will be screen failed.

VII. **Barriers to pregnancy intervention assessment-1:** This will be a questionnaire based assessment that includes questions relating to assessment of overall perceived barriers to life-style interventions in pregnancy, perceived difficulties to frequency of study visits, perceived willingness to interventions and study-related procedures, 9-point willingness to participate score, willingness and unwillingness to participate.

VIII. **OGTT:** A fasting glucose venous blood glucose sample taken will be following a minimum 8-hour fast. Participants will be then asked to drink a calibrated dose (75 grams) of anhydrous glucose solution within 10 minutes as soon as possible. Subsequent blood samples will be collected at 60 and 120 minutes following the drink. Based on IADPSG criteria, a diagnosis of GDM will be made if one or more values equals or exceeds the following:

	Values in mmol/l	Values in mg/dl
Fasting glucose	≥5.1 mmol/l	92 mg/dl
1-hour glucose	≥10.0 mmol/l	180 mg/dl
2-hour glucose	≥8.5 mmol/l	153 mg/dl

Women qualifying the criteria for diabetes (based on IADPSG cut-offs) at this stage will be screen failed.

7.5.2. Baseline assessment at Run-in visit (Visit: -1; week ≤17)

I. **Weight:** Weight of the mother will be recorded as described in 7.5.1 (II)

II. **Blood pressure:** Systolic and diastolic BP of the mother will be recorded as described in 7.5.1 (III)

III. **Antenatal examination (fundal height):** Routine antenatal examination including fundal height will be recorded as described in 7.5.1 (IV)

IV. **Dairy consumption assessment:** Background dairy product consumption of the study participant will be assessed using standardized questionnaires.

V. **Safety assessments** to interventions (e.g. adverse events and/or SAE assessment)

VI. **Concomitant medication** assessment

VII. **Baseline PA monitoring:** All participants will be advised to wear an accelerometer device to objectively measure baseline PA for next 7 days. The step counts will be blinded (not made visible on the device) in order to avoid influences on routine activity.

7.5.3. Baseline assessment at Randomization (Visit: 0; week ≤18)

I. **Weight:** Weight of the mother will be recorded as described in 7.5.1 (II)

II. **Blood pressure:** Systolic and diastolic BP of the mother will be recorded as described in 7.5.1 (III)

For peer review only

III. **Antenatal examination (fundal height):** Routine antenatal examination including fundal height will be recorded as described in 7.5.1 (IV)
IV. **Intervention allocation**
V. **Compliance assessment:** to interventions (step count, yoghurt diary)
VI. **Safety assessments** to interventions (e.g. adverse events and/or SAE assessment)
VII. **Concomitant medication** assessment.

7.6. Subsequent Visits

7.6.1. Visit 1 (week 18-20)

I. **Weight:** Weight of the mother will be recorded as described in 7.5.1 (II)
II. **Blood pressure:** Systolic and diastolic BP of the mother will be recorded as described in 7.5.1 (III)
III. **Antenatal examination (fundal height):** Routine antenatal examination including fundal height will be recorded as described in 7.5.1 (IV)
IV. **Safety assessments** to interventions (e.g. adverse events and/or SAE assessment).
V. **Concomitant medication** assessment
VI. **Compliance assessment:** to interventions (step count, yoghurt diary)
VII. **Morphology scan:** The following variables would be measured and recorded: Gestational age, bi-parietal diameter, head circumference, femoral length, Abdominal circumference, estimated foetal weight, liquor amount, placenta: anterior/posterior and upper segment/lower segment, amniotic Fluid Volume Index, Uterine artery Doppler: Pulsatility Index, Systolic/diastolic ratio, gross anomalies in region of head and neck (transcerebellar plane, transventricular plane, biparietal plane, nose/lips), thorax (lungs, 4 chamber view, outflow tract, diaphragm), Spine, Abdomen (stomach bubble, kidneys, cord insertion, number of vessels in cord, genitalia, limbs). Participants with any foetal congenital anomaly, placental malformation or malposition or abnormal ultrasound features that can compromise pregnancy outcome according to the investigator will be withdrawn from the study at this stage.

7.6.2. Visit 2 (week 26-28)

I. **Weight:** Weight of the mother will be recorded as described in 7.5.1 (II)
II. **Blood pressure:** Systolic and diastolic BP of the mother will be recorded as described in 7.5.1 (III)
III. **Antenatal examination (fundal height):** Routine antenatal examination including fundal height will be recorded as described in 7.5.1 (IV)
IV. **OGTT** (as described in in 7.5.1 (VIII))
V. **Safety assessments** to interventions (e.g. adverse events and/or SAE assessment) and review of concomitant medications.
VI. **Compliance assessment:** to interventions (step count, yoghurt diary)

For peer review only

7.6.3. Visit 3 (Week 32) - END OF STUDY VISIT

I. **Weight:** Weight of the mother will be recorded as described in 7.5.1 (II)
II. **Blood pressure:** Systolic and diastolic BP of the mother will be recorded as described in 7.5.1 (III)
III. **Antenatal examination (fundal height):** Routine antenatal examination including fundal height will be recorded as described in 7.5.1 (IV)
IV. **Fasting plasma glucose**
V. **Barriers** to pregnancy intervention-2 assessment
VI. **Ultrasound scan:** The ultrasound scan would be done during visit 3 (week 32) in all participants which would include measurement of bi-parietal diameter (BPD), head circumference, femoral length, abdominal circumference, estimated foetal weight, liquor amount, placenta: anterior/posterior and upper segment/lower segment, amniotic Fluid Volume Index, Uterine artery Doppler: Pulsatility Index, Systolic/diastolic ratio.
VII. **Safety assessments** to interventions (e.g. adverse events and/or SAE assessment) and review of concomitant medications.
VIII. **Compliance assessment:** to interventions (step count, yoghurt diary) & close of interventions.

In the event the participant withdraws from the study at any time prior to week 32 visit, then the date of voluntary withdrawal will be marked as "END OF STUDY VISIT". The participant will undergo all assessments as listed in 7.6.3 except morphology scan.

7.6.4. Post-intervention surveillance visit

All study-related intervention will be stopped at 32 weeks and mother and new born data will be collected during delivery as a part of post- trial surveillance visit. Women who consent will have weekly telephonic contacts/home visits by field staff to ensure general wellbeing. All women will be delivered in the hospital and inter-partum and neonatal measurements will be recorded as below

I. **Weight of the mother** before delivery of the baby
II. **Intrapartum maternal outcome assessment**
III. **Birth length (new born)** The measurement from the crown to the soles of the feet in centimetre scale would be recorded as birth length by an experienced mid-wife.
IV. **Birth weight (new born)** Weight at birth would be measured within 48 hours of delivery using a calibrated scale for measuring neonatal birth weight by an experienced mid-wife.
V. **APGAR score (new born)** The APGAR Score should be used as the accepted and convenient method for reporting the status of the new born immediately after birth and the response to resuscitation if needed. Local neonatal practices should be followed for APGAR score reporting in cases of maternal sedation or anaesthesia, congenital malformations and birth trauma. The APGAR assessment would be made as below by a trained medical neonatologist/paediatrician or midwife at 1 and 5 minutes.

For peer review only

Sign	0	1	2
Colour	Blue or Pale	Acrocyanotic	Pink
Heart rate	Absent	<100/minute	>100/minute
Reflex irritability	No response	Grimace	Cry or Active withdrawal
Muscle tone	Limp	Some flexion	Active motion
Respiration	Absent	Weak Cry	Good, Crying

All women recruited in the study will be regularly contacted over telephone or by home visit to monitor general well-being, diet and PA adherence and early detection of serious adverse events and safety to interventions. The additional weekly phone review is also expected to assist with adherence and retention.

7.7. Sample Handling

Blood sample

Following a minimum of 8 hour overnight fast, women will provide a fasting sample followed by blood samples at 1 hour and 2 hours of consuming oral glucose solution (see Section 7. Oral Glucose Tolerance Test). 2ml venous blood will be collected for each measurement in an EDTA fluoride vacutainer tube (Grey Capped) and placed on ice until centrifuge. Samples will be centrifuged (2,750 rpm for 10 minutes), aliquoted and transported to the analysis lab on ice. OGTT glucose samples and fasting venous glucose during visit 3 will be analysed on Roche Cobas 800 Autoanalyser either on the same day or will be stored in freezer if analysed on the subsequent date. A total of 12 ml blood will be collected during both OGTTs. General timelines for sample handling will be followed (venepuncture to ice no more than 10 minutes, ice to centrifuge no longer than 30 minutes, centrifuge to ice no more than 10 minutes and ice to freezer no more than 6 hours). No samples will be stored for future use. The current study does not involve any genetic or molecular analysis.

7.8. Discontinuation/Withdrawal

Discontinuation/withdrawal has to be distinguished from i) discontinuation from the study (interventions, follow-up visits, study-related procedures) and ii) discontinuation from interventions only and could occur under following circumstances

7.8.1. Discontinuation/Withdrawal from the study

Discontinuation from the study occurs

- When a participant voluntary withdraws consent to further participation at any time during the trial.

For peer review only

- Significant protocol deviation such as Ineligibility (either arising during the study or retrospectively having been overlooked at screening)
- Loss to follow-up

Details of withdrawing/discontinuation from the study prior to completion of all scheduled visits should entered in the "study termination form"

Participants who are diagnosed to have GDM (by laboratory evidence within the protocol or outside diagnosis confirmed by an OGTT at study centre) should continue interventions till week 32 (end of study visit), unless there is a safety concern.

Temporary or permanent discontinuation of interventions (as detailed in sections 7.8.1 and 7.8.2) for safety reasons will not lead to withdrawal of the participant from the trial. Withdrawal of the participants will not be replaced by recruitment of new participants. The primary reason for withdrawal will be specified in the Case Report Form.

7.8.2. Discontinuation/Withdrawal from interventions

7.8.2.1. Temporary discontinuation/withdrawal from interventions

Temporary discontinuation from interventions may occur when a participant does not tolerate the intervention or due to a ***suspected AE*** by the investigator. Best efforts should be made to resume the intervention as early as possible.

7.8.2.2. Permanent discontinuation/withdrawal from interventions

Permanent discontinuation from intervention is any intervention discontinuation associated with the definitive decision from the Investigator not to re-expose the participant to the either yoghurt or PA or both at any time during the study. This may occur during situations of ***unexpected SAE***, where, in the investigator's opinion, continuation with intervention would be detrimental to the participant's well-being

Examples of permanent discontinuation could be
- development of blood pressure 150/100 mmHg anytime during the trial
- Hospitalisation due to medical event (only if further continuation of interventions will affect maternal or new born health)
- Anaemia diagnosed based on haemoglobin <10g/dl
- Ultrasound evidence of major degree placenta previa

Participants will be followed-up according to the study procedures specified in this protocol up to the scheduled date of study completion. It is important to collect data on all participants, regardless of intervention, during the entire duration of the study as all data until the scheduled date of study completion will be used in statistical analyses.

In case the participant decides to withdraw participation or consent during the study, we will not work on participant's samples without permission, but any information already generated from the samples until the time of withdrawal will be used and samples already collected, for which they have given consent, will also be analysed and data used.

For peer review only

7.9. Lost to follow-up

A subject will be considered potentially lost to follow-up if she completed all protocol specific procedures up to the administration of the intervention, but was then lost during the follow-up period, with no safety information and no efficacy endpoint data ever became available. This could occur in situations where a participant repeatedly fails to return for scheduled visits and is unable to be contacted by the trial site.

- Before a subject is deemed lost to follow-up, the investigator should make every effort to regain contact with the subject.
- A subject will not be declared lost to follow-up before all the attempts have been repeated and the trial has come to an end.

The attempts must be documented in the CRF.

7.10. Protocol deviation

A protocol deviation is any noncompliance with the clinical trial protocol, good clinical practice (GCP), or other applicable regulatory requirements. The noncompliance may be either on the part of the participant or the investigator including the study team members, and may result in significant added risk to the study participant.

Deviations from the protocol should be avoided. If deviations do occur, they must be documented and explained in a protocol deviation CRF by stating the reason, date and the action(s) taken. As a result of a deviation, corrective action should be developed and implemented promptly. The investigator must inform the trial monitor and the implications of the deviation must be reviewed and discussed. All protocol deviations must be notified to the regulatory authorities according to local requirements

Deviation from the protocol can occur at following circumstances

- When the investigators miss to schedule a visit
- If an ineligible subject is randomised in error/randomisation stratification error

7.11. Definition of End of Study

End of study will be the last scheduled visit as per protocol i.e., visit 3 (week 32). However, if the participant chooses to discontinue form the study due to any reasons as stated above in 7.8, the "study termination form" should be completed.

8. INTERVENTIONS

The study will be a 2 x 2 factorial design and eligible women will be randomized to either one of the arms – (i) Yoghurt (ii) Physical activity (PA) (iii) Yoghurt + PA (iv) Standard care

8.1.1. Yoghurt intervention

Women randomized to 'Yoghurt' and 'Yoghurt + PA' arms will consume 1 pot (200g) of yoghurt daily from the day of randomization visit to 32 weeks of gestation (more details provided under section 5 Study design – interventions).

8.1.2. PA intervention

For peer review only

Women randomized to 'PA' and 'Yoghurt + PA' arms will follow PA intervention (daily walking and weekly group activity sessions) from randomization to Visit 3 (week 32).

8.1.2.1. Daily walking

"Absolute target" will be calculated as a minimum of a 40% increase from the baseline. This will be calculated as the average of the weekly step count at run-in-phase. Step counts will be monitored using Garmin vivofit 4 band from randomization. Participants will be advised to use the "absolute target" as the minimum reference step count, but try to increase the step count beyond this. Women will be encouraged to perform walking at as speed faster than usual but within the individual's comfort.

Compliance will be calculated based on average step count achieved over a 7-day period including two weekend days. Women achieving at least 80% of the absolute target on a 7-day average will be considered as compliant. Non-compliant participants will be continually motivated and encouraged to achieve their absolute targets either telephonically or by home visits by field workers.

Baseline a	40% increase b	Minimum absolute target (a+b)	80% of absolute target (d=a+b) increase (expected average 1 week step count to be achieved) Used to calculate compliance
2500	1000	3500	3300
3000	1200	4200	3960
3500	1400	4900	4620
4000	1600	5600	5280
4500	1800	6300	5940
5000	2000	7000	6600
5500	2200	7700	7260
6000	2400	8400	7920
6500	2600	9100	8580
7000	2800	9800	9240
7500	3000	10500	9900
8000	3200	11200	10560
8500	3400	11900	11220
9000	3600	12600	11880

For example, if baseline activity is 6,000 steps, then an increase of 40% would be an increase of 2,400 steps a day and absolute target to be achieved/day would be a minimum of 8,400 steps.

For peer review only

9. SAFETY REPORTING

Safety evaluations will be performed on all randomized participants at each follow-up visit (Visit 1-3). All AE and SAE, including expected SAEs will be captured in the AE report form. The participants will be instructed to contact the study team if there are any concerns regarding the interventions.

9.1. Adverse event reporting

Any adverse event reported by the participant will be captured in the 'adverse event report' form. Information on AE will be collected by the treating physician at each scheduled visit and by the field worker during home visits. Besides scheduled study visits, AEs will be collected through weekly phone reviews. Any participant reporting an AE will be seen by the research clinician at the earliest appointment (depending on the severity). Details of all events including onset date, causality to the study intervention will be reported, treatment given, stop date will be captured in the respective AE report form.

9.2. Definition of Serious Adverse Events

An untoward medical event leading to /resulting in any of the following will be considered as "serious adverse event"

- Death
- is life-threatening
- requires inpatient hospitalisation or prolongation of existing hospitalisation
- results in persistent or significant disability/incapacity
- loss of foetal movements or ultrasonographically confirmed foetal death
- 'Important medical events' if they jeopardise the participant or require an intervention to prevent one of the above consequences.

9.2.1. Expected SAE

Expected SAEs include potential medical events (as listed below) will be considered as they are likely to be related to the interventions. These events are therefore **protocol-defined exceptions to SAE reporting**. These events should be captured in the SAE form, but does not require reporting to DMC within scheduled timelines. However, local ethics committees should be informed of such events if the principal investigator believes the event is related to PRIMORDIAL interventions.

- persistent vaginal spotting/bleeding after randomisation
- calf pain/swelling (diagnosed to be related to thrombophlebitis or deep vein thrombosis)
- Dyspnoea at rest
- painful premature contractions
- decreased foetal movements
- preterm labour
- preterm/premature rupture of membranes
- Diagnosis of placenta praevia after 26 weeks of pregnancy
- Pre-eclampsia / Eclampsia

For peer review only

9.3. Reporting Procedures for Serious Adverse Events

All research staff will undergo formal GCP training as well as on reporting procedures for AEs and SAEs.

The PI shall report all SAEs without filtration, whether or not related to the intervention, within 24 hours of becoming aware of the event to the Sponsor. If the SAE is related to the intervention, the respective Ethics Committee should be notified within seven calendar days if fatal or life-threatening, and all others within 15 calendar days.

The minimum information required for this initial SAE report is:

- Trial number and (short) title
- Participant's ID
- Date and time of onset
- Description of the event (clinical history, associated signs and symptoms)
- Intervention administered
- Reporter's name

The PI/study coordinator will not wait for additional information to fully document the event before notifying. The report is then to be followed by submission of a completed SAE Report Form as soon as possible, detailing relevant aspects of the SAE in question. All actions taken by the PI and the outcome of the event must also be reported immediately. A decision whether to continue/discontinue the intervention (yoghurt+/- PA) will be made by the treating obstetrician depending on the causality of the event.

For documentation of the SAE, any actions taken, outcome and follow-up, a SAE Report Forms will be used. All follow-up activities have to be reported, if necessary on one or more consecutive SAE report forms in a timely manner. All fields with additional or changed information must be completed and the report form should be forwarded to the respective Ethics Committee within seven calendar days after receipt of the new information. Hospital case records and autopsy reports, including verbal autopsy, will be obtained where applicable.

All SAEs would be reported to the Data Monitoring Committee (DMC) and Oxford Tropical Research Ethics Committee (OxTREC) where in the opinion of the PI the event was 'related' (resulted from administration of any of the research procedures) and 'unexpected' (the type of event is not listed in the protocol as an expected SAE). Reports of related and unexpected SAEs should be submitted within 15 days of the PI becoming aware of the event to DMC and OxTREC.

10. STATISTICS AND ANALYSIS

10.1. Description of Statistical Methods

We will analyse the study using both (1) the intention-to-treat principle and (2) per protocol, including only those participants adhering to at least 75% of the proposed intervention. In addition, we will run a sensitivity analysis to assess the effect of protocol deviation compared to 100% adherence. Analyses will be performed blinded to the intervention group allocation.

For peer review only

Descriptive analysis of the first 40 participants who complete visit 2 (26-28 weeks) will be performed as advised by the funders (MRC-UK). The analysis is intended to assess feasibility, retention and adherence to all study-related procedures.

10.2. The Number of Participants

The sample size is 1856 high-risk women (928 to be recruited in The Gambia and India). The study was powered to have 80% to detect a 33% reduction in the incidence of GDM, for the main effects of diet and PA, with a family-wise significance level of 5% for a two-sided test (i.e. each effect was tested at a significance level of 2.5%). It was assumed that participants could be pooled across centres (no interaction between interventions and country at the 5% level). The sample size was inflated to allow for a 15% drop out rate in each of the four strata, with drop outs defined as non-compliant participants (i.e. no risk reduction in their incidence of GDM).

The effect sizes for the sample size calculation were:

Prevalence of GDM in high-risk pregnant women: The largest study of GDM women (The HAPO study, 25,505 pregnant women unselected for GDM risk at 15 centres in nine countries) reported a GDM prevalence rate, based on IADPSG criteria, of 17.8% among the general population. In our 'high-risk' population, we assume that the prevalence will be higher than in the HAPO study. Therefore, we have estimated the prevalence of 25% in our study.

Estimated risk reduction: A recent meta-analysis (Song et al. Obesity reviews 2016) has shown that either diet or PA results in 18% (95% CI 5-30%) risk reduction in GDM in general pregnant population and interventions as early as 15 weeks of gestational age was also associated with reduced GDM risk (Relative risk: 0.80, 95%CI 0.66-0.97). Putting all these together, we estimate the risk reduction would be higher (about 1/3, 33%) in this high-risk population. We modelled the GDM percentages in four equal-sized combinations of control (c) and treatment (t) arms as 25% (c-c), 16.75% (c-t and t-c) and 11.2 in t-t group. Therefore, in simple comparisons of one exposure, the percentages will be 20.9% vs 13.0%.

*Absence of intervention*country interaction:* In order to pool the participants of both countries, we will be assuming that there are no differences between the participants of both countries. This initial test for a non-significant interaction term (at the 5% level) between each intervention and country was also taken into account in the sample size simulation.

The sample size calculation was performed by simulation (in R version 3.5.2) according to the following algorithim:

1. For a given sample size, we generated 8 subgroups, two (one per country) for each intervention combination (control-control, treatment1-control, control-treatment2, treatment1-treatment2).

2. For each subgroup we generated the expected GDM incidence, except for a random 15% (drop-outs), whose expected GDM incidence was considered to be 25% (corresponding to the control group) regardless of their originally allocated subgroup.

For peer review only

3. We first tested if either the interaction term site*PA or site*Yoghurt was statistically significant (at the 5% level).

4. If the assumption of no intervention*country interaction was met, we recorded for each intervention if it was statistically significant (at the 2.5%, as we were doing two tests, one for each intervention), assuming a two-sided test

5. This was repeated 10,000 times.

6. The % of repetitions with statistically significant intervention effects represents the power associated with the sample size.

7. Steps 1-6 were repeated for increasing sample sizes.

8. A smoothed (lowess) line was fitted.

9. The point where this line crossed the 80% power mark gave us the final sample size required. (Figure 1)

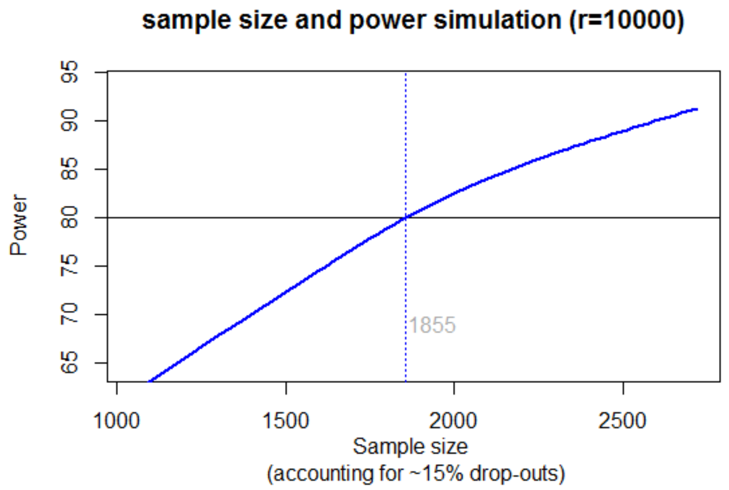

Figure 1: Estimated power for increasing sample size, with an estimated risk reduction of 33%, alpha = 0.05 and estimated drop-out rate of 15% (based on 10,000 iterations).

10.3. Analysis of Outcome Measures

All outcomes will be presented using descriptive statistics; normally distributed data by the mean and standard deviation and skewed distributions by the median and interquartile range. Binary variables will be presented using counts and percentages. We will summarise descriptive data using CONSORT reporting standards for RCTs. STATA will be used for statistical analysis.

10.3.1. Primary outcome

Gestational diabetes incidence will be analysed as risk ratios and risk differences will be estimated by binary regression and results presented as odds ratio and 95% confidence interval.

A covariate adjusted analysis of the effect of treatment on the primary outcome will be undertaken to remove bias from the estimate of the treatment effect on the primary outcome.

We will also test for the difference in effects on GDM incidence between the centres. If the odds ratio (OR) for GDM associated with the intervention in The Gambia is ORg, and in India is ORi, then the ratio of these two terms measures the difference in effect.

10.3.2. Secondary outcomes

The secondary outcomes will compare the difference in fasting plasma glucose at week 32, gestational weight gain, intrapartum and neonatal outcomes between the intervention and control groups. Continuous variables will be analysed using multiple linear regression and binary variables by logistic regression and adjusted for pre-specified covariates.

10.3.3. Safety

The incidence rates of AEs and SAEs and their relationship to trial interventions will be summarised by intervention group. The proportion of women discontinuing treatment will be summarised by reason and by intervention group.

11. DATA MANAGEMENT

All protocol required data will be captured in electronic Case Report forms (eCRF) that will be completed for each included participant by trial personnel. Data will be regularly checked by the Data Manager. On the eCRF, a reference to the source document will be provided. Instructions for completing all forms, including the CRF, used in the study will be developed. All data will be entered onto a secured, password-protected database that will only be accessed by the data manager and designated investigators.

11.1. Types of data

The study will include collection of both research (quantitative and qualitative) and personal data. Data minimisation has been considered and thus personal data will only include name, addresses and telephone number collected for the purpose of telephonic follow-ups/reminders and home visits by the field staff for the current study. These data will however be also used if the participant needs to be contacted for future approved studies (follow-up of babies born to high-risk GDM mothers). Personal information will be available only to the study PI and designated staff involved in contacting the participants either telephonically or by home visits. As this study has the potential to be extended in the future for other follow-up studies, personal data will pseudonymised and stored locally at their study centres. There will be no transfer/sharing of personal data outside the study centre. This personal data will be not be linked to research data and will be maintained in separate files at each study centre and will be password protected and accessible only to authorized staff of the study team.

11.2. Format and scale of data

REDCap software will be used in the trial. It is a secure web application for building and managing databases. The final data format will be downloaded to Excel and common statistical packages (SPSS, SAS, Stata, R). REDCap is specifically geared to support online or offline data

For peer review only

capture for research studies and operations. This data management system is a fully supported, externally validated (GCP compliant to 21 CFR parts 11) clinical database. The database will be hosted and managed by the MRCG and the final tested production database will be shared with the partner site in India who will enter/synchronize data into the central database.

11.3. Data collection/generation

Research data will be collected using electronic CRFs (eCRFs). Data collection will be undertaken by designated study staff involved in the study. Only those individuals who have been trained on the CRF/eCRFs and/or other document completion and have been authorized by the Site PI may complete eCRFs and other study specific documentation. The research data collected on the eCRFs will be captured using devices i.e. tablets and/or laptops used by study staff. Where there's internet connectivity, collected data will be stored directly onto the central server and where there is no/unreliable connectivity, data will be stored locally on the tablet and later synchronized to the central servers at MRCG Fajara. Field staff will be trained to use of the REDCap mobile application and clinicians will be trained to use the REDCap web portal. The data from the accelerometer used for monitoring PA will be directly downloaded on the tablet by the trained research staff on a weekly basis.

11.4. Data quality and standards

Review of data collection tools against the protocol will occur prior to database build by the Data Developer to ensure all data is being captured, at the correct time points, and in a format conducive to the proposed statistical analysis. Site based staff will be fully trained in conducting any study assessments and subsequent completion of the CRF and a Site delegation log will be maintained. Study staff will be trained in the use of Redcap. Principles of GCP will be adhered to and all training will be documented. To ensure standardisation of processes, standard operating procedures with respect to trial management, quality assurance, data management, IT & security, and statistics will be adhered to. The entry screens will be designed with range checks, skip patterns and validations to assist/guide data collectors and to ensure high level accuracy. Multiple choice options will be employed when and where applicable. Mandatory field checks and other computable data will be pre-populated to eliminate errors. The data manager will conduct periodical data cleaning routines to flag data queries that were not picked up in the earlier stages. All data queries raised by the data manager will be answered by the local PI or a delegated senior member of the study team involved in the generation of the data in question normally within one week. All changes to any CRFs or databases will be governed by a change control SOP, ensuring that all sites are using the same version of both CRFs and databases.

11.5. Managing, storing and curating data

Paper records will be stored securely in locked rooms or cabinets, according to standard filing and archiving procedures by both centres. Electronic data collected at both sites (India and The Gambia) will be centrally monitored and stored by the MRC Unit, The Gambia. The database will be housed on a secure network server with no direct physical access to it by project team members. Access to the system is via a web interface that will be hosted on the Fajara production server (https://redcap.mrc.gm/redcap/redcap) managed by the IT department. All data stored in REDCap will be formatted in XML when exported using Clinical Data. Interchange Standards Consortium Open Data Model (CDISC ODM) export format which is vendor neutral,

For peer review only

platform independent format for interchange and archive of clinical trials data. The database will be backed up as part of the IT department's backup routine and disaster recovery process. ICF will be maintained locally in the respective study centres in secure cabinets will limited access to authorized study staff only. The ICF will be retained for a period of 5 years after study completion and publication of primary results. Other PID (personally identifiable data) will be stored indefinitely for the purpose of future follow-up studies.

11.6. Data preservation strategy and standards

At the end of the study (when the last participant enrolled in the study completes the delivery visit) the database will be locked and all the electronic study data will be housed in the REDCap Database server until the first publication is made following which the data will be sent to the electronic data archives for long-term preservation. The locked database will have very highly controlled access as detailed in the Archives Research Records Management Policy (POL-ARM-001 - Available on request). All electronic records will be kept indefinitely and all paper records and lab books will be stored in the on-site temperature and pest controlled MRC as dictated by the MRC Unit The Gambia's SOPs on Data Retention and Destruction (SOP-ARM-005) and formal information/data security standards The study will be run in compliance with the MRC Corporate Information security Policy and the MRC Unit The Gambia's INFORMATION AND COMMUNICATION TECHNOLOGY SECURITY POL-INT-001 available on the MRC Gambia intranet (Available on request).

11.7. Main risks to data security

Main risk to confidentiality is the personal information on subjects kept by the local-PI for study follow up verification purposes. These will be kept separate from the actual research data and not available to the study staff so that no linkages of personal data to study records would be easily made. The research data will be stored in a limited access, password protected database so that only staff who have the required permissions can view the study records. Personal information will be available only to study PI and designated staff involved in contacting the participants either telephonically or by home visits.

11.8. Suitability for sharing

The data generated within the study will be suitable for sharing in an anonymised format. The data will be in a format that is suitable to share with other interested researchers. All data meant for sharing will be anonymised/de-identified by removing all individual-level participant data (IPD) and will be archived on the servers of the Archives department of MRCG. Data sharing will be in agreement with the MRCG as outlined and governed by the Data Sharing Policies i.e. Data Sharing Policy v2.2 and MRC Policy and Guidance on Sharing of Research Data from Population & Patient Studies. The regulations will also adhere to the University of Oxford data policy guidelines. PID will not be shared across organisations.

11.9. Access to Data

Direct access will be granted to authorised representatives from the University of Oxford and any host institution for monitoring and/or audit of the study to ensure compliance with regulations.

For peer review only

11.10. Data Handling and Record Keeping

All study data will be downloaded from RedCap into an Excel spreadsheet. The participants will be identified by a unique study specific number in any database. The name, addresses and any other identifying details will NOT be included in any study data electronic file. All data will be fully described in the Excel data dictionary which will include the variable name, variable description, label (if different from description), database table name, data grouping information, type of data (sting, integer, real etc.), data codes and decodes and any validation rules used. All data stored in REDCap will be formatted. Person data and research data will not be linked. After completion of the study, research data will be stored indefinitely at central servers in MRC, Fajara, The Gambia. PID including ICF will be stored for a minimal period of 3 years after study completion and destroyed subsequently as per local data management policies. All data management processes and procedures will be governed by the library of MRCG's data management standard operating procedures.

12. COMMITTEES STRUCTURES

The study will be conducted in accordance with the current approved protocol, Good Clinical Practice (GCP) regulations and standard operating procedures (SOPs).

12.1. DATA SAFETY MONITORING COMMITTEE (DSMC)

An independent Data Safety Monitoring Committee (DSMC) has been set up to review the progress of the trial and provide feedback to the Trial Steering Committee (TSC).

The DMC will be responsible for reviewing

(i) general study progress (recruitment, compliance, loss to follow-up)
(ii) clinical study safety data and assess and/or adjudicate all events of deaths
(iii) the GDM event rate and key secondary/safety outcomes
(iv) results of the interim analysis. The DSMC includes the following members (To address: chair of DSMC, Report preparation, confirm meeting timelines).

The DMC will review and analyze, on a regular basis, unblinded safety data throughout the study.

An independent DSMC with members who are independent from the Sponsor and the investigators for the PRIMORDIAL study comprises of experts in clinical trials/epidemiology, biostatistics and obstetrics and include the following members

Name (Designation)	Address	Email
Dr. Udak Okoma (Paediatrician & Postdoctoral Research Fellow)	MRC Unit The Gambia at the London School of Hygiene & Tropical Medicine, Fajara, Banjul, The Gambia	uokomo@mrc.gm
Dr. Matthew Johnson (Biostatistician)	MRC-LEU, University of Southampton, UK	mj2@mrc.soton.ac.uk

For peer review only

Dr. Lucy McKillop (Obstetrician)	Nuffield Department of Obstetrics and Gynaecology, Oxford, UK.	lucy.mackillop@ouh.nhs.uk

Meetings will be held by skype prior to start of the recruitment and then 6-monthly thereafter. The study protocol will be approved by the DSMC prior to enrolment. Any subsequent updates and implications will be reviewed periodically.

12.2. Trial Steering committee (TSC)

The TSC is responsible for supporting the Sponsor in designing a scientifically sound study. In this capacity, the members of the TSC shall address and resolve scientific issues encountered during the study. The TSC would take decisions on protocol amendments (along with DSMC, if required) and communicate with the research teams. All changes would be implemented following ethics approval from all participating bodies.

Name	Address	Email / Phone
Prof Lisa K Micklesfield (Independent chair)	University of Witwatersrand, Johannesburg, South Africa	Lisa.Micklesfield@wits.ac.za
Dr. G. Krishnaveni (Independent member)	CSI Holdsworth Memorial Hospital, Mysore, India	gv.krishnaveni@gmail.com
Prof Louise Bowman (Independent member)	CTSU, Nuffield Department of Population Health, Oxford	louise.bowman@ndph.ox.ac.uk
Prof Fredrik Karpe (Principal investigator)	OCDEM, Radcliffe department of Medicine, Oxford	fredrik.karpe@ocdem.ox.ac.uk
Prof Caroline Fall (Co-investigator)	MRC-LEU, University of Southampton, Southampton, UK	chdf@mrc.soton.ac.uk
Dr. Senthil Vasan (Trial Manager, Non-voting members)	OCDEM, Radcliffe department of Medicine, Oxford	senthil.vasan@ocdem.ox.ac.uk
Dr. Melissa Lennartz-Walker (Newton Fund Program Manager, MRC)	MRC	Melissa.Lennartz-Walker2@mrc.ukri.org

For peer review only

12.3. Executive committee

The executive committee includes active investigators who will be directly involved in the day-to-day running of the trial

Name	Address	Email / Phone
Prof Fredrik Karpe (Principal investigator)	OCDEM, Radcliffe department of Medicine, Oxford	fredrik.karpe@ocdem.ox.ac.uk
Dr. Senthil Vasan	OCDEM, Radcliffe department of Medicine, Oxford	senthil.vasan@ocdem.ox.ac.uk
Prof Andrew Prentice	MRC Unit The Gambia at LSHTM, Atlantic Boulevard, Fajara, P.O. Box 273, Banjul, The Gambia	Andrew.Prentice@lshtm.ac.uk
Dr. Modou Jobe	MRC Unit The Gambia at LSHTM, Atlantic Boulevard, Fajara, P.O. Box 273, Banjul, The Gambia	mojobe@mrc.gm
Dr. Mustapha Bittaye	MRC Unit The Gambia at LSHTM, Atlantic Boulevard, Fajara, P.O. Box 273, Banjul, The Gambia	mubittaye@mrc.gm
Prof Jiji Matthews	Department of Obstetrics and Gynaecology, Christian Medical College & Hospital, Vellore, India	coronistrial@yahoo.co.in
Dr Swathi Rathore	Department of Obstetrics and Gynaecology, Christian Medical College & Hospital, Vellore, India	swatixrathore@gmail.com

13. ETHICAL AND REGULATORY CONSIDERATIONS

13.1. ICH-GCP

The study will be conducted in accordance with the protocol and applicable International Conference on Harmonisation (ICH) GCP guidelines.

For peer review only

13.2. Approvals

The protocol, protocol amendments, ICF and the PIS will be submitted to the OxTREC, and host institution (MRCG and Ethics committee, CMC, Vellore) for written approval. Additionally, approvals will also be sought from Indian Council of Medical Research (ICMR) and the Medical control agency, The Gambia. The Investigator will submit and, where necessary, obtain approval from the above parties for all substantial amendments to the original approved documents.

13.3. Participant Confidentiality

The study staff will ensure that the participants' anonymity is maintained. The participants will be identified only by a participant ID number on all study documents and any electronic database. All documents will be stored securely and only accessible by study staff and authorised personnel. The study will comply with the Data Protection Act, which requires data to be anonymised as soon as it is practical to do so. Personal data will be not be linked to research data and maintained in separate files which are password protected and accessible only to authorized staff of the study team. More details are provided under Section 11.

13.4. Expenses and Benefits

Participants will be paid a sum equivalent to their day wage for their study visit as agreed by the respective ethics committees. Non-working mothers will be provided a compensation as agreeable by the respective ethics committees of Vellore and The Gambia. Reasonable travel expenses for any study-related visits will be reimbursed on production of receipts, or a mileage allowance provided as appropriate. The study participants will not have to pay for any study-related procedures (OGTT, ultrasound scans and study consultations).

13.5. Annual Progress Report

The PI would submit annually, a Progress Report to the OxTREC with a copy to CTRG. Annual report will also be submitted to the funding body – The MRC, UK.

13.6. Other Ethical Considerations

The study involves non-invasive measurements of the new born. Prior informed consent will be obtained from either the father or the mother to record new-born measurements. The parents of the new born will be informed that they can withdraw the consent from obtaining their child's measurements at any time point. No personal data of the new born will be collected. New-born's will be identified by a unique study identification number and linked to the mothers' research data of the mother and father's study identification number.

14. FINANCE AND INSURANCE

14.1. Funding

The study is funded by the Medical Research Council, UK and Global Challenges Research Fund, UK for the UK and the Gambian costs and by the Department of Biotechnology (DBT), India for the Indian costs. The Indian costs will be directly dealt between DBT and CMC, Vellore. The

For peer review only

sponsor of the study is Oxford University. The funders have no role in study design; collection, management, analysis, and interpretation of data; writing of the report; and the decision to submit the report for publication.

14.2. Insurance

The University of Oxford has a specialist insurance policy in place which would operate in the event of any participant suffering harm as a result of their involvement in the research (Newline Underwriting Management Ltd, at Lloyd's of London).

15. PUBLICATION POLICY

The Investigators will be involved in reviewing drafts of the manuscripts, abstracts, press releases and any other publications arising from the study. The study will be registered and the study protocol will be made available in publically accessible websites such as ISRCTN and some national registries (Clinical Trials Registry, India). The final results of the study will be published in peer-reviewed scientific journals. Authorship will be determined in accordance with the ICMJE guidelines and other contributors will be acknowledged. For those journals that request sharing of the analyzable data sets, requests should be made to the PI-Fredrik Karpe. All publications will acknowledge that the study was funded by MRC, UK and DBT, India.

In order to disseminate our research findings with general public and study participants, we will engage with our communities, the wider public, the media, the broad scientific community and national and international agencies. We will explain the relevance of our research to our research participants and provide feedback of the results to them. We will use websites, the written press, TV and radio, and presentations to lay and school groups in order to ensure that our research findings reach the widest possible public audience. We will hold annual retention meetings of the study participants where results of the interim analysis and final study will be conveyed.

For peer review only

16. REFERENCES

1. Buchanan TA, Xiang AH. Gestational diabetes mellitus. J Clin Invest 2005;115(3):485-91.
2. Reece EA, Leguizamón G, Wiznitzer A. Gestational diabetes: the need for a common ground. Lancet 2009;373(9677):1789-97.
3. Bellamy L, Casas JP, Hingorani AD, Williams D. Type 2 diabetes mellitus after gestational diabetes: a systematic review and meta-analysis. Lancet 2009;373(9677):1773-9.
4. Qin J, Li Y, Cai Z, Li S, Zhu J, Zhang F at al. A metagenome-wide association study of gut microbiota in type 2 diabetes. Nature 2012;490(7418):55-60.
5. Sanz Y, Santacruz A, Gauffin P. Gut microbiota in obesity and metabolic disorders. Proc Nutr Soc 2010;69(3):434-41.
6. Collado MC, Isolauri E, Laitinen K, Salminen S. Distinct composition of gut microbiota during pregnancy in overweight and normal-weight women. Am J Clin Nutr 2008;88(4):894-9.
7. Monda V, Villano I, Messina A, Valenzano A, Esposito T, Moscatelli F et al. Exercise Modifies the Gut Microbiota with Positive Health Effects. Oxid Med Cell Longev 2017;2017:3831972.
8. Le Chatelier E, Nielsen T, Qin J, Prifti E, Hildebrand F, Falony G et al. Richness of human gut microbiome correlates with metabolic markers. Nature 2013;500(7464):541-6.
9. Metzger BE, Buchanan TA, Coustan DR, de Leiva A, Dunger DB, Hadden DR et al. Summary and recommendations of the Fifth International Workshop-Conference on Gestational Diabetes Mellitus. Diabetes Care 2007;30 Suppl 2:S251-60.
10. Hunt KJ, Schuller KL. The increasing prevalence of diabetes in pregnancy. Obstet Gynecol Clin North Am 2007;34(2):173-99
11. Sacks DA, Hadden DR, Maresh M, Deerochanawong C, Dyer AR, Metzger BE at al. Frequency of gestational diabetes mellitus at collaborating centers based on IADPSG consensus panel recommended criteria: the Hyperglycemia and Adverse Pregnancy Outcome (HAPO) Study. Diabetes Care 2012;35(3):526-8.
12. Hunsberger M, Rosenberg KD, Donatelle RJ. Racial/ethnic disparities in gestational diabetes mellitus: findings from a population-based survey. Womens Health Issues 2010;20(5):323-8.
13. Savitz DA, Janevic TM, Engel SM, Kaufman JS, Herring AH. Ethnicity and gestational diabetes in New York City, 1995-2003. BJOG 2008;115(8):969-78.
14. Seshiah V, Balaji V, Balaji MS, Sanjeevi CB, Green A. Gestational diabetes mellitus in India. J Assoc Physicians India 2004;52:707-11.
15. Macaulay S, Dunger DB, Norris SA. Gestational diabetes mellitus in Africa: a systematic review. PLoS One 2014;9(6):e97871.
16. Harlev A, Wiznitzer A. New insights on glucose pathophysiology in gestational diabetes and insulin resistance. Curr Diab Rep 2010;10(3):242-7.
17. Koivusalo SB, Rönö K, Klemetti MM, Roine RP, Lindström J, Erkkola M et al. Gestational Diabetes Mellitus Can Be Prevented by Lifestyle Intervention: The Finnish Gestational Diabetes Prevention Study (RADIEL): A Randomized Controlled Trial. Diabetes Care 2016;39(1):24-30.
18. Cani PD, Delzenne NM. The role of the gut microbiota in energy metabolism and metabolic disease. Curr Pharm Des 2009;15(13):1546-58.
19. Gijsbers L, Ding EL, Malik VS, de Goede J, Geleijnse JM, Soedamah-Muthu SS. Consumption of dairy foods and diabetes incidence: a dose-response meta-analysis of observational studies. Am J Clin Nutr 2016;103(4):1111-24.
20. Chen M, Sun Q, Giovannucci E, Mozaffarian D, Manson JE, Willett WC et al. Dairy consumption and risk of type 2 diabetes: 3 cohorts of US adults and an updated meta-analysis. BMC Med 2014;12:215.

For peer review only

21. Dugoua JJ, Machado M, Zhu X, Chen X, Koren G, Einarson TR. Probiotic safety in pregnancy: a systematic review and meta-analysis of randomized controlled trials of Lactobacillus, Bifidobacterium, and Saccharomyces spp. J Obstet Gynaecol Can 2009;31(6):542-52.
21. Russo LM, Nobles C, Ertel KA, Chasan-Taber L, Whitcomb BW. Physical activity interventions in pregnancy and risk of gestational diabetes mellitus: a systematic review and meta-analysis. Obstet Gynecol 2015;125(3):576-82.
22. Sanabria-Martínez G, García-Hermoso A, Poyatos-León R, Álvarez-Bueno C, Sánchez-López M, Martínez-Vizcaíno V. Effectiveness of physical activity interventions on preventing gestational diabetes mellitus and excessive maternal weight gain: a meta-analysis. BJOG 2015;122(9):1167-74.
23. Barakat R, Pelaez M, Cordero Y, Perales M, Lopez C, Coteron J et al. Exercise during pregnancy protects against hypertension and macrosomia: randomized clinical trial. Am J Obstet Gynecol 2016;214(5):649 e1-8.
24. Wiebe HW, Boulé NG, Chari R, Davenport MH. The effect of supervised prenatal exercise on fetal growth: a meta-analysis. Obstet Gynecol 2015;125(5):1185-94.
25. Barakat R, Cordero Y, Coteron J, Luaces M, Montejo R. Exercise during pregnancy improves maternal glucose screen at 24-28 weeks: a randomised controlled trial. Br J Sports Med 2012;46(9):656-61.
26. de Barros MC, Lopes MA, Francisco RP, Sapienza AD, Zugaib M. Resistance exercise and glycemic control in women with gestational diabetes mellitus. Am J Obstet Gynecol 2010;203(6):556 e1-6.
27. ACOG Committee Opinion No. 650. Physical Activity and Exercise During Pregnancy and the Postpartum Period. Obstet Gynecol 2015;126(6):e135-42.
28. Campbell SC, Wisniewski PJ. Exercise is a Novel Promoter of Intestinal Health and Microbial Diversity. Exerc Sport Sci Rev 2017;45(1):41-47.
29. Lindsay KL, Walsh CA, Brennan L, McAuliffe FM at al. Probiotics in pregnancy and maternal outcomes: a systematic review. J Matern Fetal Neonatal Med 2013;26(8):772-8.
30. Laitinen K, Poussa T, Isolauri E; Nutrition, Allergy, Mucosal Immunology and Intestinal Microbiota Group. Probiotics and dietary counselling contribute to glucose regulation during and after pregnancy: a randomised controlled trial. Br J Nutr 2009;101(11):1679-87.
31. Luoto R, Laitinen K, Nermes M, Isolauri E. Impact of maternal probiotic-supplemented dietary counselling on pregnancy outcome and prenatal and postnatal growth: a double-blind, placebo-controlled study. Br J Nutr 2010;103(12):1792-9.
32. Zheng J, Feng Q, Zheng S, Xiao X. The effects of probiotics supplementation on metabolic health in pregnant women: An evidence based meta-analysis. PLoS One. 2018 May 21;13(5):e019777
33. Hajifaraji M, Jahanjou F, Abbasalizadeh F, Aghamohammadzadeh N, Abbasi MM, Dolatkhah N. Effect of probiotic supplements in women with gestational diabetes mellitus on inflammation and oxidative stress biomarkers: a randomized clinical trial. Asia Pac J Clin Nutr. 2018;27(3):581-591.
34. Nitert MD, Barrett HL, Foxcroft K, Tremellen A, Wilkinson S, Lingwood B et al. SPRING: an RCT study of probiotics in the prevention of gestational diabetes mellitus in overweight and obese women. BMC Pregnancy Childbirth. 2013 Feb 25;13:50.
35. Barakat R, Pelaez M, Cordero Y, Perales M, Lopez C, Coteron J et al. Exercise during pregnancy protects against hypertension and macrosomia: randomized clinical trial. Am J Obstet Gynecol 2016;214(5):649 e1-8.

For peer review only

36. Barakat R, Cordero Y, Coteron J, Luaces M, Montejo R. Exercise during pregnancy improves maternal glucose screen at 24-28 weeks: a randomised controlled trial. Br J Sports Med 2012;46(9):656-61.
37. Poston L, Bell R, Croker H, Flynn AC, Godfrey KM, Goff L at al. Effect of a behavioural intervention in obese pregnant women (the UPBEAT study): a multicentre, randomised controlled trial. Lancet Diabetes Endocrinol 2015;3(10):767-77
38. da Silva SG, Hallal PC, Domingues MR, Bertoldi AD, Silveira MFD, Bassani D et al. A randomized controlled trial of exercise during pregnancy on maternal and neonatal outcomes: results from the PAMELA study. Int J Behav Nutr Phys Act. 2017 Dec 22;14(1):175.
39. Haakstad LA, Bø K. Effect of regular exercise on prevention of excessive weight gain in pregnancy: a randomised controlled trial. Eur J Contracept Reprod Health Care. 2011 Apr;16(2):116-25.
40. Wang C, Wei Y, Zhang X, Zhang Y, Xu Q, Sun Y et al. A randomized clinical trial of exercise during pregnancy to prevent gestational diabetes mellitus and improve pregnancy outcome in overweight and obese pregnant women. Am J Obstet Gynecol. 2017 Apr;216(4):340-351.
41. Vinter CA, Jensen DM, Ovesen P, Beck-Nielsen H, Jørgensen JS. The LiP (Lifestyle in Pregnancy) study: a randomized controlled trial of lifestyle intervention in 360 obese pregnant women. Diabetes Care 2011;34(12):2502-7.
42. Renault KM, Nørgaard K, Nilas L, Carlsen EM, Cortes D, Pryds O at al. The Treatment of Obese Pregnant Women (TOP) study: a randomized controlled trial of the effect of physical activity intervention assessed by pedometer with or without dietary intervention in obese pregnant women. Am J Obstet Gynecol 2014;210(2):134 e1-9.
43. Koivusalo SB, Rönö K, Klemetti MM, Roine RP, Lindström J, Erkkola M et al. Gestational Diabetes Mellitus Can Be Prevented by Lifestyle Intervention: The Finnish Gestational Diabetes Prevention Study (RADIEL): A Randomized Controlled Trial. Diabetes Care 2016;39(1):24-30.
44. Dodd JM, Turnbull D, McPhee AJ, Deussen AR, Grivell RM, Yelland LN at al. Antenatal lifestyle advice for women who are overweight or obese: LIMIT randomised trial. BMJ 2014;348:g1285.
45. Grivell RM, Yelland LN, Deussen A, Crowther CA, Dodd JM. Antenatal dietary and lifestyle advice for women who are overweight or obese and the effect on fetal growth and adiposity: the LIMIT randomised trial. BJOG. 2016 Jan;123(2):233-43.
46. Bennett CJ, Walker RE, Blumfield ML, Gwini SM, Ma J, Wang F. Interventions designed to reduce excessive gestational weight gain can reduce the incidence of gestational diabetes mellitus: A systematic review and meta-analysis of randomised controlled trials. Diabetes Res Clin Pract. 2018 Jul;141:69-79.
47. Shepherd E, Gomersall JC, Tieu J, Han S, Crowther CA, Middleton P. Combined diet and exercise interventions for preventing gestational diabetes mellitus. Cochrane Database Syst Rev. 2017 Nov 13;11:CD010443
48. Petrella E, Malavolti M, Bertarini V, Pignatti L, Neri I, Battistini NC et al. Gestational weight gain in overweight and obese women enrolled in a healthy lifestyle and eating habits program. J Matern Fetal Neonatal Med. 2014 Sep;27(13):1348-52.

For peer review only

18. APPENDIX A: STUDY FLOW CHART

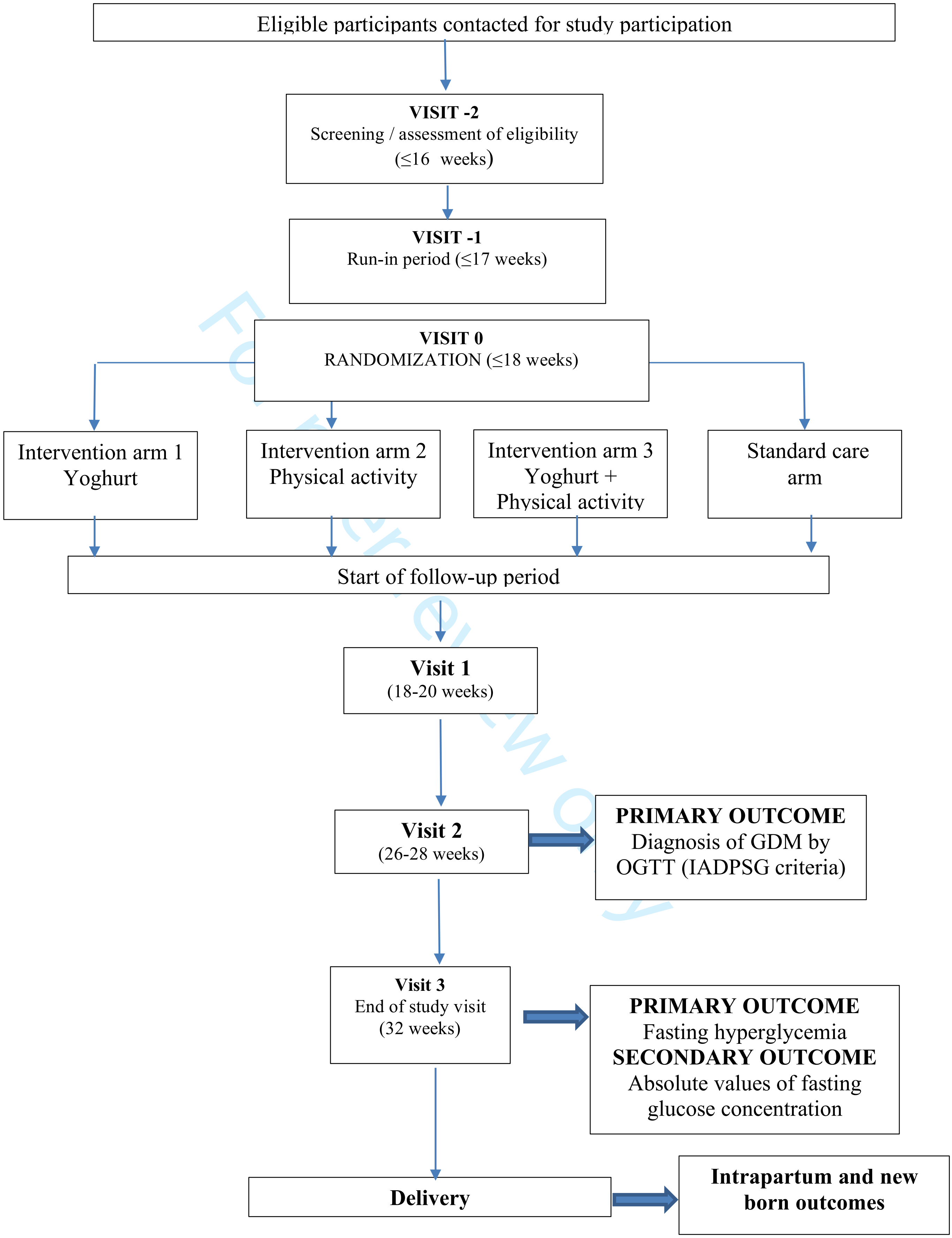

For peer review only

19. APPENDIX B: STUDY TIMELINES (approximate)

	2018-2019		2019		2020		2021	
	July - Nov	Dec 2018 - Jan 2019	Feb - June	Jul - Dec	Jan-Jun	Jul - Dec	Jan-Jun	Jul - Dec
Protocol preparation								
Regulatory approvals								
TSC meeting								
Investigators meeting								
Staff training								
Study initiation								
Recruitment & follow up								
Study close out								
Interim analysis								
Monitoring visits								
Data quality checks								
DMC meeting								
Annual report preparation								
Final data analysis								
Preparation of manuscripts								
Publication of manuscripts								
Final report submission								

For peer review only

20. APPENDIX C: SCHEDULE OF STUDY PROCEDURES

Procedures	Visits						
	Visit -2	Visit -1	Visit 0	Visit 1	Visit 2	Visit 3	Delivery
	Screening ≤16 weeks	Run-in-phase ≤17 weeks	Randomization Week ≤18	Week 18-20	Week 26-28	Week 32	
Informed consent	X						
Eligibility assessment	X						
Demographics	X						
History	X						
Height	X						
Weight	X	X	X	X	X	X	X
Blood pressure	X	X	X	X	X	X	X
Antenatal examination	X	X	X	X	X	X	
Dairy consumption questionnaire		X					
Barriers questionnaire	X					X	
Baseline PA monitoring with Vivofit 4		X					
PA step-count monitoring			X	X	X	X	
PA assessment (Baseline)		X					
Randomisation			X				
OGTT	X				X		
Dating scan	X						
Morphology scan				X			
Ultrasound scan						X	
Fasting plasma glucose						X	
Intrapartum assessment (mother)							X
New born assessment							X

For peer review only

21. APPENDIX D: PATIENT INFORMATION SHEET

PATIENT INFORMATION SHEET
Lifestyle interventions during pregnancy to prevent diabetes (PRIMORDIAL Study)

OxTREC reference number 44-18
Version 2.1 dated 19th September 2019

1. What's the purpose of this research?

Around 3 in 10 pregnant women are at risk for developing diabetes during pregnancy, and the risk is higher in African and Asian women. Diabetes during pregnancy imposes serious health effects to both the mother and the baby. For some women, this may be a sign of developing diabetes permanently in the future. In this study, we would like to find out, if simple lifestyle changes in diet and physical activity during pregnancy might help in reducing the risk of developing diabetes in pregnant women.

2. Why have I been invited to take part?

You are invited to take part in this study because you have been identified to have one (or more than one) condition that places you at an increased risk for developing diabetes during pregnancy. Risk factors can be being overweight or obese, previous pregnancy with diabetes, Age ≥25 years, first-degree relative with diabetes, previous pregnancy with large baby (≥3.5kg), previous pregnancy with hypertension/seizures, and history of impaired fasting glucose.

3. What's involved for me?

After study explained to you, if you agree to take part you will have to provide your consent. There are 6 visits in this study. Y our doctor may request you to attend the clinic for additional care if required. Information on what you will be asked to do at each visit are as below

Screening visit (Visit -2): At this first visit, you will be asked to do a timed blood test called the oral glucose tolerance test (OGTT), an ultrasound scan, answer a barriers questionnaire and routine clinical examination by your doctor.

A sample of your blood will be taken to measure fasting glucose (sugar). You should have not consumed any food or drink (except water) for a minimum 8 hours from previous night. You will then be given a sugary drink and subsequently blood samples will be taken at one hour and two hours from the time you have taken the drink. You cannot eat anything during the test but you can drink water. The test will take about 2 hours 15 minutes to be completed. The doctor will then perform an ultrasound scan to find out how many weeks of pregnancy are you currently in and also will be able to tell you the expected date of delivery. In addition, the doctor will take some basic measurements of the growing baby. The results of the scan will be informed to you immediately. The scans will take about 30 minutes. You will also to fill a questionnaire relating to difficulties you anticipate, that will prevent you from being physically active during pregnancy and eating healthy food including yoghurt during pregnancy. This will take about 30 minutes to complete.

You will be contacted within one week regarding your blood test and your eligibility to further continue in the study.

Run-in-phase (Visit -1): At the second visit, you will be asked questions relating to your regular milk-products and yoghurt consumption. This will take about 15 minutes for each of the questionnaire. You will also be asked to wear a watch-like device around your wrist for the next 7 days and do your routine day-to-day work. You will also be seen in the clinic by the doctor for general examination

Randomization (Visit 0): To be eligible to participate further in this study, you should have worn the wrist band on all 7 days as advised.

You will be allotted to one of the study arms – the yoghurt arm or the physical activity arm or the yoghurt and the physical activity arm or standard care arm. If you are allocated to the diet arm, you will have to consume a pot of yoghurt (200g) till 32 weeks of pregnancy. In the physical activity arm, you will be asked to increase your walking everyday till 32 weeks of pregnancy. In the yoghurt and physical activity arm, you will have to do both of the above. In the standard care arm, you will receive regular care from your physician.

Visit 1: In the fourth visit, the physician will perform an ultrasound scan to see the growth of the baby. Routine antenatal examination will be done by your doctor.

Visit 2: In the fifth visit, you will be asked to do blood tests similar to your first visit seen in the clinic by the doctor for routine antenatal examination.

Visit 3: A fasting blood sample (minimum 8-10 hours fasting from previous night) for blood glucose measurement will be taken and another scan to see if your baby is growing well. You will also be asked to complete a barriers questionnaire and provide feedback about your participation in the trial. All other routine antenatal care will be provided by your doctor.

This is the last visit and all interventions stop here. You will be contacted during delivery for measurements of yourself and new-born baby during delivery.

In the event that you develop diabetes at any point during the study, your physician will advise you on additional medications.

During the entire period you will be home visited by a field worker or telephonically contacted by a research staff to check if everything is ok.

4. What will happen to my data?

Research data You will be given a study number so that any information that you provide to us will be made anonymous. All data will be securely stored in the research office, kept in locked cupboards/secure servers in locked rooms with restricted access. All data will be owned by your local research investigator, kept in locked cupboards/secure servers in locked rooms with restricted access. Anonymised data will be shared between researchers in the Gambia, India, and Oxford, UK for research purposes. All research data collected as electronic records will be kept indefinitely in electronic data archives in The Gambia and may be used for future research studies with your consent. Responsible members of the research team may be given access to data for monitoring and/or audit of the study to ensure that the research is complying with applicable regulations.
Personal data Your name, date of birth, address and telephone number will be stored separately in order to invite you to follow-up visits and for the field staff to visit you at home. This information will not be shared with other researchers.

5. What will happen to my samples?

The blood samples will be used for study purpose only and analysed locally. Samples will not be shipped/stored overseas and will not be shared with other researchers. No genetic tests are carried out in this study.

6. Are there any risks in taking part?

There may be mild bruising/swelling in the area where blood is drawn. Consumption of oral glucose solution in empty stomach may cause nausea, vomiting, headache. Ultrasound scans during pregnancy are considered safe to the mother or the baby as the radiation exposure is minimal.

By consuming yoghurt, you may feel nauseated, experience vomiting, bowel upset or diarrhoea. Physical activity during pregnancy is considered generally safe. However, you may experience some common adverse effects like dizziness, headache, and breathlessness on exertion, easy fatigability and muscle aches. If either of the interventions is not tolerable, you can contact your doctor and he may stop the intervention if necessary.

For peer review only

7. What are the benefits of taking part?

The direct benefits of participating in this study is that your blood sugar levels, blood pressure and weight will be closely monitored. By achieving strict control of your sugar levels, blood pressure and weight, we hope to see immediate benefits and lifelong health impact on yourself and your baby.
You will be sensitised to healthy lifestyle during pregnancy.

8. Do I have to take part?

Participation in this study is voluntary. You can ask questions/concerns about the study to one of the members of the research team before deciding whether to take part. Your decision will not affect your routine clinical care. You can also withdraw from the study without penalty at any time.

10.Has the study been reviewed by an ethics committee?

The study has been reviewed and approved by the ethics committees of University of Oxford, UK, Christian Medical College & Hospital, Vellore, India and the MRC Unit, The Gambia.

11.What if I have any questions or want to raise a concern?

If you have any concerns about the study at any point or wish to complain about any aspect of the way in which you have been approached or treated in this study, you may contact one of the below mentioned persons

Research team:
Name:
Address:
Phone number:

Member independent of the study team
Name
Address:
Phone number:

12. Data protection
The University of Oxford is responsible for ensuring the safe and proper use of any personal information you provide, solely for research purposes.

For peer review only

22. APPENDIX E: INFORMED CONSENT FORM

INFORMED CONSENT FORM

Lifestyle interventions during pregnancy to prevent diabetes (PRIMORDIAL Study)

OxTREC reference number 44-18

1. I confirm that I have read the information sheet for the above study. I have had the opportunity to consider the information and ask questions, and have had these answered satisfactorily.	Yes / No
2. I give consent for my research data to be stored locally and shared with other investigators overseas	Yes / No
3. I agree to take part in this study.	Yes / No

Name of participant ____________ Date ____________ Signature ____________

Name of person taking consent ____________ Date ____________ Signature ____________

- For illiterate participants:

Name of participant ____________ Thumbprint of participant

Name of witness ____________ Relationship to participant ____________ Date ____________ Signature ____________

Name of person taking consent ____________ Date ____________ Signature ____________

For peer review

23. APPENDIX F: AMENDMENT HISTORY

All protocol amendments whenever a new version of the protocol is produced will be updated here.

Amendment No.	Protocol Version No.	Date issued	Author(s) of changes	Details of Changes made
1	2.1	19th October 2018	Senthil K Vasan<br>Fredrik Karpe<br>Caroline Fall<br>Clive Osmond	1. Stratification for risk factors during randomization (Section 7.2)<br>2. Explanations to sample size calculation (Section 10.2)<br>3. Study analysis details included unde section 10.3.1 and 10.3.2<br>4. Details of DSMB included under section 12.<br>5. SAE reporting to DSMB and OxTREC is included under section 9.3.<br>6. Skin fold thickness measurement of the new born is removed from the protocol<br>7. Under Section 13,4, the following line is changed “Participants will be paid a sum equivalent to their day wage for their study visit as agreed by the respective ethics committees”.
2	3.0	09th January 2019	Senthil K Vasan<br>Fredrik Karpe<br>Caroline Fall<br>Clive Osmond<br>Dylan Thompson	1. Additional change to primary outcome measure (eelevated fasting plasma glucose ≥5.1 mmol/l at 32 weeks GA) in Page 4<br>2. Secondary objective changed tc “To determine the effect of life style intervention (yoghurt and PA), given to high-risk women from 18 weeks of gestation, on fasting blood glucose at 32 weeks (Pages 4 and 16)<br>3. Low birth weight changed to ‘birth weight’ under section other outcome measures in Page 5

For peer review only

				4. Ponderal index and neonatal anthropometry deleted under neonatal outcomes in Pages 5, 16, 19, 27)<br>5. Run-in visit - Baseline PA will be assessed (blinded to participants) using a wearable accelerometer device (Garmin Vivofit 4 fitness band) for 7 days to objectively measure baseline step count in all participants (Page 17)<br>6. Visit 3 (32 weeks GA, End of Study visit): morphology scan changed to ultrasound scan (Pages 17, 26)<br>7. Physical actvity intervention The recommendation changed to 80% of the absolute target increase from the average target step count measured during run-in-phase (Pages 18 and 29)<br>8. Group session will use a patient participant initiative (PPI) approach where participants wi be involved in designing the type of group activity, which may include activities such as group walking, dancing, stretching exercises etc. (Page 18)<br>9. Reassessment of PA activity: All participants will be asked to objectively measure step count for next 7 days using Garmin Vivofit4 at visit 2 (26-28 weeks) Women not randomized to the diet and standard care arm will have blinded assessments, whil women on PA arm (PA arm or Diet+PA) will continue to achieve target step counts without blinded assessment (Page 18).

For peer review only

					10. Data collection process – height and weight of father newly added (Pages 19, 21, 23-24)<br>11. Inclusion criteria changed to ≥1 weeks and ≤ 16 weeks of gestational age (Pages 20,23)<br>12. Inclusion criteria stamen revise as: In addition to the above, pregnant women should meet at least one of the following criteria for high-risk GDM (Page 20).<br>13. Exclusion criteria related to hypertension changed as "Currently on treatment for hypertension (pre-gestational o gestational)" (Page 20).<br>14. New exclusion criteria added: Physical disability to PA and/or known lactose intolerance (pag 21).<br>15. Details of stratification at randomisation provided: Random allocation to intervention will be prestratifie for age, BMI and risk factor for GDM within each study centre. We will create two age bands (<25 y and ≥ 25 years), two BMI categories (<25, ≥26) and presence of any risk factor (No 0/ Yes 1) (Page 23).<br>16. PA intervention details and 80% absolute target step count char included in Page 29.<br>17. PA compliance definition included in Page 29 as "compliance" will be defined as will be defined as at least 80% c absolute target increase achieved on an average over 7-days period.<br>18. Statistical methods changed as "The primary analysis will be intention-to-treat. We will also

For peer review only

				do a secondary analysis using the per protocol principle" in page 31.<br>19. Members of the executive committee newly included in Pages 36-37.<br>20. Appendix A – study flow chart Primary and secondary outcomes changed in Page 42.<br>21. Timelines of investigators meeting, study initiation, staff training in Gnat chart changed i Page 43
3	4.0	09th February 2019	Senthil K Vasan<br>Fredrik Karpe<br>(Changes as suggested from TSC)	1. Primary objective – outcome measure: 26-28 weeks included newly<br>2. 'Other outcome measures' rename Trial and post-trial surveillance' (Sections 1, 4).<br>3. maternal blood pressure added as objective and serial blood pressure measurement at all visits included a outcome measurement (Sections 1, 4).<br>4. For maternal outcome measurements under 'Trial and post-trial surveillance' data on intra and postpartum outcomes will be captured on eCRF from delivery records (Section 1).<br>5. New born outcomes measure preterm births will be based on ultrasound based gestational age recorded at screening (Section 1).<br>6. DSMB changed to DMC (data monitoring committee) in abbreviations. New abbreviations added – LSHTM, PID (Section 2).<br>7. Protocol change: Under Section 5 – Run-in-visit – consumption of yoghurt by all participants for 7 day removed.

For peer review only

				8. Protocol change: Section 5 – Run-in visit and in data collection process, Section 7: Baseline dietary assessment using FFQ changed to background dairy consumption questionnaire.<br>9. Under Section 5 and Section 7– Visits 1-3: 'routine' added to antenatal examination.<br>10. Under Section 5 – Visit 3: GDM women will continue on allocatec study intervention till week 32 in addition to the treatment provided as per local obstetric practice. This i changed from previous version that stated 'terminating women who develop GDM from further intervention'.<br>11. Protocol change: Under Section 5 - Interventions: Newly added: All other (yoghurt and standard arm) will also be provided with fitness bands to objectively measure their routine PA behaviour throughout the study. However, step counts wil be blinded for these participants.<br>12. Under Section 6.2 – Medications revised to include women who are hypothyroid on thyroxine supplementation, low dose aspirin for pre-eclampsia) and women conceived by ovulation induction using clomiphene citrate or metformin for PCOD will not be excluded from the study.<br>13. Section 6.3 – Exclusion criteria – GDM diagnosed prior to screening visit based on IADPSG criteria or documented raised HbA1C.<br>14. Section 7 – Post-trial surveillance – Capillary blood glucose (mother) deleted.<br>15. Section 7.3 – Screening and Eligibility assessment: Newly added 'In addition to above, participants

For peer review only

				should meet at least one of the below mentioned criteria to be termed as "high-risk" for inclusion into the study'<br>16. Section 7.4 – Randomisation: Newly added: After qualifying for eligibility and completion of the run-in-visit, details of age, BMI and GDM risk wi be provided to the independent statistician, who will then assign the intervention arm. Randomisation allocation will be communicated to the study sites through emails.<br>17. Section 7.6.1 – Newly added under morphology scan - Participants with any foetal congenital anomaly, placental malformation or malposition or abnormal ultrasound features that can compromise pregnancy outcome according to th investigator will be withdrawn from the study at this stage.<br>18. Section 7.6.1, 7.6.2, 7.6.3: Change made - Safety assessments to interventions (e.g. adverse events and/or SAE assessment).<br>19. Section 7.8.1 – more details provided for "Discontinuation/withdrawal"<br>20. Section 7.8.2 – Newly added "discontinuation from interventions".<br>21. Section 7.9 - more details provided for "lost to follow-up".<br>22. Section 7.11 – last scheduled visit changed from Visit 2 (26-28 weeks) to visit 3 (week 32).<br>23. Section 8.1.2 – Details of PA intervention provided under sections 8.1.2.1 – daily walking and 8.1.2.2 – Group activity sessions.<br>24. Section 9.1 – Details of AE reporting in eCRF added as 'Details of all events including onset date, causality to the study intervention

For peer review only

				will be reported, treatment given, stop date will be captured in the respective AE report form'.<br>25. Section 9.2.1 – Expected SAE – newly added.<br>26. Section 12 title changed as "Committee structures"<br>27. Section 12.1., 12.2., 12.3 – Roles an responsibilities of DMC, TSC and executive committees are included.<br>28. Section 13.1 renamed as ICH-GCP.<br>29. Section 15 – Publication Policy: Registration with ISRCTN and Clinica Trials Registry, India newly added.<br>30. Section 15 - Publication Policy: Fredrik Karpe will be the contact person for requests for sharing analysable data.<br>31. PPAQ removed at run-in-visit.
4	4.1	09th April 2019	Senthil K Vasan<br>Alexander Jarde<br>Fredrik Karpe	1. Gestational age at screening, run-in and randomisation visit are changed from 16, 17 and 18 weeks to ≤16, ≤17 and ≤18 weeks (Pages 17, 20-25)<br>2. Detailed randomisation procedure outlined in Page 23. Risk factors for GDM is removed from pre-stratification criteria.<br>3. Section 10 on Statistics and analysis (incl. 10.1 and 10.2) are expanded to provide more details (Pages 33-35)
5	4.2	18th July 2019	Senthil K Vasan<br>Fredrik Karpe	1. Scheduled visit 1 is changec form week 22 to week 18-2 weeks. This is in line with ACOG and NICE antenatal guidelines for morphology scan at week 18-20 (Pages 17,21,25,46)

For peer review only

SPIRIT

STANDARD PROTOCOL ITEMS: RECOMMENDATIONS FOR INTERVENTIONAL TRIALS

SPIRIT 2013 Checklist: PRIMORDIAL Study

Section/item	PageNo	Description
**Administrative information**		
Title	1	Descriptive title identifying the study design, population, interventions, and, if applicable, trial acronym
Trial registration	1	Trial identifier and registry name. If not yet registered, name of intended registry
Protocol version	1	Date and version identifier
Funding	1	Sources and types of financial, material, and other support
Roles and responsibilities	1	Names, affiliations, and roles of protocol contributors
	1	Name and contact information for the trial sponsor
	42	Role of study sponsor and funders, if any, in study design; collection, management, analysis, and interpretation of data; writing of the report; and the decision to submit the report for publication, including whether they will have ultimate authority over any of these activities
	39-41	Composition, roles, and responsibilities of the coordinating centre, steering committee, endpoint adjudication committee, data management team, and other individuals or groups overseeing the trial, if applicable (see Item 21a for data monitoring committee)
**Introduction**		
Background and rationale	9-16	Description of research question and justification for undertaking the trial, including summary of relevant studies (published and unpublished) examining benefits and harms for each intervention
Objectives	10,17	Specific objectives or hypotheses
Trial design	18	Description of trial design including type of trial (eg, parallel group, crossover, factorial, single group), allocation ratio, and framework (eg, superiority, equivalence, noninferiority, exploratory)
**Methods: Participants, interventions, and outcomes**		
Study setting	22-23	Description of study settings (eg, community clinic, academic hospital) and list of countries where data will be collected. Reference to where list of study sites can be obtained

For peer review only

For peer review only - http://bmjopen.bmj.com/site/about/guidelines.xhtml

Eligibility criteria	21	Inclusion and exclusion criteria for participants. If applicable, eligibility criteria for study centres and individuals who will perform the interventions (eg, surgeons, psychotherapists)
Interventions	19, 31	Interventions for each group with sufficient detail to allow replication, including how and when they will be administered
	29	Criteria for discontinuing or modifying allocated interventions for a given trial participant (eg, drug dose change in response to harms, participant request, or improving/worsening disease)
Outcomes	17, 25, 35	Primary, secondary, and other outcomes, including the specific measurement variable (eg, blood glucose, OGTT), analysis metric (eg, change from baseline, final value, time to event), method of aggregation (eg, median, proportion), and time point for each outcome. Explanation of the clinical relevance of chosen efficacy and harm outcomes is strongly recommended
Participant timeline	22-26	Time schedule of enrolment, interventions (including any run-ins and washouts), assessments, and visits for participants. A schematic diagram is highly recommended (see Figure)
Sample size	34,35	Estimated number of participants needed to achieve study objectives and how it was determined, including clinical and statistical assumptions supporting any sample size calculations
Recruitment	22,23	Strategies for achieving adequate participant enrolment to reach target sample size

Methods: Assignment of interventions (for controlled trials)

Allocation:

Sequence generation	24	Method of generating the allocation sequence (eg, computer-generated random numbers), and list of any factors for stratification. To reduce predictability of a random sequence, details of any planned restriction (eg, blocking) should be provided in a separate document that is unavailable to those who enrol participants or assign interventions
Implementation	24	Who will generate the allocation sequence, who will enrol participants, and who will assign participants to interventions

Methods: Data collection, management, and analysis

Data collection methods	37-38	Plans for assessment and collection of outcome, baseline, and other trial data, including any related processes to promote data quality (eg, duplicate measurements, training of assessors) and a description of study instruments (eg, questionnaires, laboratory tests) along with their reliability and validity, if known. Reference to where data collection forms can be found, if not in the protocol

For peer review only - http://bmjopen.bmj.com/site/about/guidelines.xhtml

Data management	36-38	Plans for data entry, coding, security, and storage, including any related processes to promote data quality (eg, double data entry; range checks for data values). Reference to where details of data management procedures can be found, if not in the protocol
Statistical methods	33-36	Statistical methods for analysing primary and secondary outcomes. Reference to where other details of the statistical analysis plan can be found, if not in the protocol
	35,36	Methods for any additional analyses (eg, subgroup and adjusted analyses)
	33,36	Definition of analysis population relating to protocol non-adherence (eg, as randomised analysis)
**Methods: Monitoring**		
Data monitoring	39	Composition of data safety monitoring committee (DSMC); summary of its role and reporting structure; statement of whether it is independent from the sponsor and competing interests; and reference to where further details about its charter can be found, if not in the protocol. Alternatively, an explanation of why a DMC is not needed
Harms	32	Plans for collecting, assessing, reporting, and managing solicited and spontaneously reported adverse events and other unintended effects of trial interventions or trial conduct
**Ethics and dissemination**		
Research ethics approval	41	Plans for seeking research ethics committee/institutional review board (REC/IRB) approval
Protocol amendments	40,54-60	Plans for communicating important protocol modifications (eg, changes to eligibility criteria, outcomes, analyses) to relevant parties (eg, investigators, REC/IRBs, trial participants, trial registries, journals, regulators)
Consent or assent	23	Who will obtain informed consent or assent from potential trial participants or authorised surrogates, and how
Confidentiality	38	How personal information about potential and enrolled participants will be collected, shared, and maintained in order to protect confidentiality before, during, and after the trial
Access to data	38	Statement of who will have access to the final trial dataset, and disclosure of contractual agreements that limit such access for investigators

For peer review only - http://bmjopen.bmj.com/site/about/guidelines.xhtml

Dissemination policy	43	Plans for investigators and sponsor to communicate trial results to participants, healthcare professionals, the public, and other relevant groups (eg, via publication, reporting in results databases, or other data sharing arrangements), including any publication restrictions
	43	Authorship eligibility guidelines and any intended use of professional writers
Biological specimens	28	Plans for collection, laboratory evaluation, and storage of biological specimens for genetic or molecular analysis in the current trial and for future use in ancillary studies, if applicable

*It is strongly recommended that this checklist be read in conjunction with the SPIRIT 2013 Explanation & Elaboration for important clarification on the items. Amendments to the protocol should be tracked and dated. The SPIRIT checklist is copyrighted by the SPIRIT Group under the Creative Commons "Attribution-NonCommercial-NoDerivs 3.0 Unported" license.

For peer review only

BMJ Open

Protocol for a randomised controlled trial: Pregnancy Related Interventions in Mothers at risk for gestational Diabetes in Asian India and Low and middle income countries (PRIMORDIAL Study)

Journal:	*BMJ Open*
Manuscript ID	bmjopen-2020-042069.R1
Article Type:	Protocol
Date Submitted by the Author:	13-Jan-2021
Complete List of Authors:	Vasan, Senthil; University of Oxford, Radcliffe Department of Medicine; University of Southampton, MRC Lifecourse Epidemiology Unit<br>Jobe, Modou ; Medical Research Council The Gambia<br>Mathews, Jiji; Christian Medical College and Hospital Vellore, Obstetrics and Gynaecology<br>Cole , Fatoumata; MRC Unit The Gambia at London School of Hygiene & Tropical Medicine<br>Rathore, Swathi; Christian Medical College and Hospital Vellore, Obstetrics and Gynaecology<br>Jarjou, Ousman; MRC Unit The Gambia at London School of Hygiene & Tropical Medicine, Nutrition<br>Thompson, Dylan; University of Bath, Department for Health<br>Jarde, Alexander; MRC Unit The Gambia at London School of Hygiene & Tropical Medicine, , Statistics<br>Bittaye, Mustapha ; MRC Unit The Gambia at London School of Hygiene & Tropical Medicine<br>Ulijaszek, Stanley; University of Oxford, School of Anthropology<br>Fall, Caroline ; University of Southampton, MRC Lifecourse Epidemiology Unit<br>Osmond, Clive; Medical Research Council, Epidemiology Resource Centre<br>Prentice, Andrew; London School of Hygiene andTropical Medicine, MRC International Nutrition Group<br>Karpe, Fredrik; University of Oxford , Oxford center for Diabetes, Endocrinology and Metabolism
**Primary Subject Heading**:	Diabetes and endocrinology
Secondary Subject Heading:	Epidemiology, Obstetrics and gynaecology, Nutrition and metabolism, Diabetes and endocrinology
Keywords:	OBSTETRICS, PREVENTIVE MEDICINE, PUBLIC HEALTH, Diabetes in pregnancy < DIABETES & ENDOCRINOLOGY, Maternal medicine < OBSTETRICS

SCHOLARONE™
Manuscripts

I, the Submitting Author has the right to grant and does grant on behalf of all authors of the Work (as defined in the below author licence), an exclusive licence and/or a non-exclusive licence for contributions from authors who are: i) UK Crown employees; ii) where BMJ has agreed a CC-BY licence shall apply, and/or iii) in accordance with the terms applicable for US Federal Government officers or employees acting as part of their official duties; on a worldwide, perpetual, irrevocable, royalty-free basis to BMJ Publishing Group Ltd ("BMJ") its licensees and where the relevant Journal is co-owned by BMJ to the co-owners of the Journal, to publish the Work in this journal and any other BMJ products and to exploit all rights, as set out in our licence.

The Submitting Author accepts and understands that any supply made under these terms is made by BMJ to the Submitting Author unless you are acting as an employee on behalf of your employer or a postgraduate student of an affiliated institution which is paying any applicable article publishing charge ("APC") for Open Access articles. Where the Submitting Author wishes to make the Work available on an Open Access basis (and intends to pay the relevant APC), the terms of reuse of such Open Access shall be governed by a Creative Commons licence – details of these licences and which Creative Commons licence will apply to this Work are set out in our licence referred to above.

Other than as permitted in any relevant BMJ Author's Self Archiving Policies, I confirm this Work has not been accepted for publication elsewhere, is not being considered for publication elsewhere and does not duplicate material already published. I confirm all authors consent to publication of this Work and authorise the granting of this licence.

For peer review only

Protocol for a randomised controlled trial: Pregnancy Related Interventions in Mothers at risk for gestational Diabetes in Asian India and Low and middle income countries (PRIMORDIAL Study)

Senthil K Vasan[1,2], Modou Jobe[3], Jiji Mathews[4], Fatoumata Cole[3], Swathi Rathore[4], Ousman Jarjou[3], Dylan Thompson[5], Alexander Jarde[3], Mustapha Bittaye[3], Stanley J Ulijaszek[6], Caroline H D Fall[2], Clive Osmond[2], Andrew M Prentice[3], Fredrik Karpe[1,7]

[1]Oxford Centre for Diabetes, Endocrinology and Metabolism (OCDEM), University of Oxford Churchill Hospital, Oxford OX3 7LE, UK

[2]MRC Lifecourse Epidemiology Unit, Southampton General Hospital, University of Southampton, Tremona Road, Southampton, UK

[3]MRC Unit The Gambia at London School of Hygiene & Tropical Medicine, Fajara, The Gambia

[4]Department of Obstetrics & Gynaecology, Christian Medical College & Hospital, Vellore. Tamil Nadu, India

[5]Department for Health, Centre for Therapeutic Innovation, University of Bath, UK

[6]Institute of Social and Cultural Anthropology, University of Oxford, Oxford, UK

[7]NIHR Oxford Biomedical Research Centre, OUH Foundation Trust, Oxford, UK

Key words: gestational diabetes, yoghurt, physical activity, Africa, India, randomised controlled trial

Corresponding author:
Prof Fredrik Karpe
Oxford Centre for Diabetes, Endocrinology and Metabolism (OCDEM),
University of Oxford
Churchill Hospital
Oxford OX3 7LE
UK
Email: Fredrik.Karpe@ocdem.ox.ac.uk
Tel: +44-1865-857222

For peer review only

Abstract

Introduction: Lifestyle modification is the mainstay of gestational diabetes mellitus (GDM) prevention. However, clinical trials evaluating the safety and efficacy of diet or physical activity (PA) in low-and middle-income settings such as Africa and India are lacking. This trial aims to evaluate the efficacy of yoghurt consumption and increased PA (daily walking) in reducing GDM incidence in high-risk pregnant women.

Methods and analysis: The study is a 2x2 factorial, open-labelled, multi-centre randomized controlled trial to be conducted in Vellore, South India and The Gambia, West Africa. "High-risk" pregnant women (n=1,856) aged ≥18 years and ≤16 weeks of gestational age (GA), with at least one risk factor for developing GDM, will be randomized to either (i) yoghurt (ii) PA (iii) yoghurt + PA or (iv) standard antenatal care. Participants will be followed until 32 weeks of gestation with total active intervention lasting for a minimum of 16 weeks. The primary endpoint is GDM incidence at 26-28 weeks diagnosed using International Association of the Diabetes and Pregnancy Study Groups (IADPSG) criteria or elevated fasting glucose (≥5.1 mmol/l) at 32 weeks. Secondary endpoints include absolute values of fasting plasma glucose concentration at 32 weeks gestation, maternal blood pressure, gestational weight gain, intrapartum and neonatal outcomes. Analysis will be both by intention to treat and per-protocol. Continuous outcome measurements will be analysed using multiple linear regression and binary variables by logistic regression.

Ethics and dissemination: The study is approved by Oxford Tropical Research Ethics Committee (OxTREC 44-18), ethics committees of the Christian Medical College, Vellore (IRB 11367) and MRCG Scientific Coordinating Committee (SCC 1645) and The Gambia Government/MRCG joint ethics committee (L2020.E15). Findings of the study will be published in peer-reviewed scientific journals and presented in conferences.

Trial registration number: ISRCTN: 18467720 and Clinical Trials Registry, India (CTRI/2018/07/014947)

Word count:
Abstract: 294
Manuscript: 4182

For peer review only - http://bmjopen.bmj.com/site/about/guidelines.xhtml

Strengths and limitations of the study

- This study will be the largest lifestyle intervention trial among high-risk GDM mothers in India and Africa to evaluate the efficacy of daily yoghurt consumption and physical activity in reducing GDM incidence.
- The selective screening and intervention in high-risk women using low-cost interventions is suitable for GDM prevention in low-resource settings such as India and Africa.
- The trial will help understand barriers to lifestyle interventions during pregnancy and will thus help tailor lifestyle interventions that are cost-effective as well as socially and culturally acceptable to pregnant women in these populations.

For peer review only

BACKGROUND

For peer review only - http://bmjopen.bmj.com/site/about/guidelines.xhtml

Gestational Diabetes Mellitus (GDM) accounts for 86% of hyperglycaemia during pregnancy[1] and is associated with significant intrapartum complications, perinatal morbidity, and long-term risk of developing type 2 diabetes (T2D) and cardiovascular disease in both the mother and the offspring.[2-4] Consequent to the global rise in obesity prevalence and the introduction of more stringent diagnostic criteria by the International Association of the Diabetes and Pregnancy Study Groups (IADPSG), the global prevalence of GDM has been estimated at 17.8% (range 9.3–25.5%).[5] In low- and middle-income countries (LMICs), especially in India and Africa, there are few nation-wide estimates of GDM burden. Cross-sectional studies using variable diagnostic criteria report GDM prevalence ranging between 10 to 16% in India[6 7] and 11 to 14% in Africa[8 9], which is higher than the reported prevalence from high income countries like the UK (2-3% using NICE guidelines) and USA (2-10% using various criteria including IADPSG).[10 11] Direct comparisons of these estimates may not be appropriate due to inconsistency in diagnostic criteria. Nevertheless, it seems clear that LMICs are facing a high burden of GDM.

In normal pregnancy, the physiological response to reduced insulin-mediated glucose removal is an increase in insulin secretion to maintain normoglycaemia.[12] In GDM, insufficient β-cell plasticity leads to an inability to secrete adequate quantities of insulin to counterbalance the insulin resistance (IR), and this process is accelerated in obesity. Women with chronic IR states such as pre-pregnancy excess body weight or obesity, impaired glucose tolerance prior to pregnancy, polycystic ovarian syndrome (PCOS), previous GDM, advanced maternal age and rapid gestational weight gain (GWG), have a higher risk for developing glucose intolerance during pregnancy.[13] The hyperglycaemia of GDM rapidly abates following delivery and normoglycaemia normally returns within 12 weeks. However, 50-70% of GDM mothers progress to T2D within 5-10 years postpartum[14] suggesting that GDM may be a prodrome of “common T2D” and may reflect the underlying T2D frequency in the community.[15]

Screening for GDM

Following the demonstration of a linear relationship between maternal glycaemia and obstetric and perinatal outcomes by the Hyperglycemia and Adverse Pregnancy Outcome (HAPO) study,[16 17] the IADPSG recommends universal screening of all women at first antenatal visit, without restriction to high-risk pregnant women, in order to identify all potential GDM cases.[18] However, some countries have adopted selective screening, despite the possibility of missing over 40% of GDM cases.[19] In low economic setting like the LMICs, this approach may be more cost effective and oral glucose tolerance test (OGTT) may be less beneficial to low risk women.[20]

GDM management

The main goal of lifestyle modification using diet and PA in pregnancy is to prevent excessive GWG, improve materno-foetal outcomes and prevent the onset/subsequent progression to GDM/T2D in both the mother and the new born.[21] In order to maximize the benefit of such interventions, the best strategy is likely to target high-risk populations by initiating early interventions during the “critical window”, i.e. before insufficient β-cell plasticity sets in[22] (**Figure 1**).

Dietary interventions in Pregnancy

For peer review only

Meta-analysis of dietary intervention trials in pregnant women, have shown that diet modification in any form resulted in a 61% risk reduction in GDM incidence (relative risk (RR) 0.39, 95%CI 0.23-0.69).[23-26] Besides macronutrient modification, dairy products, particularly yoghurt has been shown to lower the risk of T2D, [27] through mechanisms such as reduction in inflammatory signaling and gut permeability giving rise to a range of putative signals from the gut to the systemic circulation which ameliorate metabolic endotoxemia.[28-30] Differences in gut microbial composition between healthy pregnant and GDM women provide evidence of a potential role of altered gut microbiota in pregnancy-related metabolic dysfunction.[31 32]

Although exact mechanisms of the positive effects of the manipulating the gut microbiome during pregnancy is not studied, evidence from epidemiological data suggest that using fermented dairy products or probiotics prior to and during pregnancy has positive impact on maternal weight, blood pressure, insulin resistance and plasma lipid profile.[31 33-37] The "Probiotics and Pregnancy outcome" study showed that incident GDM was lower in women supplemented with probiotics compared to diet/placebo or control groups (16% vs 36% vs 34%).[38] The Nutrition, Allergy, Mucosal immunology and Intestinal microbiota (NAMI) study showed reductions in glucose and insulin concentration, GDM incidence and central adiposity, possibly mediated by anti-inflammatory cytokine production in probiotic supplemented pregnant women.[39 40] Multiple pieces of evidence also support a positive relationship between probiotic yoghurt supplementation during pregnancy and improved glycaemic profile in women with GDM or obesity.[41-43]

Physical activity in pregnancy

PA directly impacts non-insulin mediated glucose utilization through translocation of glucose transporter type 4 (GLUT 4) on skeletal muscle.[44 45] PA during pregnancy is safe and ACOG guidelines recommend ≥30 minutes of moderate PA performed at an intensity of 3-6 metabolic equivalents, which corresponds to brisk walking at 5-7km/h on most days of the week.[46]

Evidence of a beneficial effect from PA-intervention during pregnancy mostly come from high-income countries and include a wide range of exercise programme that vary in their intensity, duration, frequency and assessment techniques. Results show that regular PA in any form improves maternal (hyperglycaemia, hypertension, weight gain and cardiovascular function)[47], obstetric (pre-eclampsia, shoulder dystocia, preterm births), and neonatal outcomes (low birth weight or small-for-gestational age, decreased fat mass, improved stress tolerance and advanced neurobehavioural maturation).[48]

Meta-analysis of RCTs of PA intervention (8 studies, n=1,441) in obese/overweight women has shown a 24% reduction in GDM incidence in the intervention groups (RR 0.76, 95% CI 0.56-1.03; p=0.07) with moderate heterogeneity between studies (I^2=50%, p=0.05).[49] Some studies have shown negative or neutral effects, including a Cochrane review (5 studies, n=1,115) of PA on GDM incidence,[50-52] and this is likely to be related to study heterogeneity, bias, type of exercise, timing, duration and intensity of exercise and also subjective assessments. Nevertheless, the potential beneficial effects of effective PA intervention in pregnancy should not be underestimated.

For peer review only - http://bmjopen.bmj.com/site/about/guidelines.xhtml

The knowledge gap on effectiveness of PA interventions in LMIC settings is particularly challenging in women from Asian Indian and African backgrounds where the baseline PA level is generally considered low. The ability to engage pregnant women from diverse ethnic groups will also be determined by population-specific socioeconomic and cultural factors.

Combined approaches in Pregnancy

Targeting multiple risk factors simultaneously may have a synergistic effect to reduce GDM. Combined approaches have shown favourable improvements in maternal and foetal outcomes, particularly GWG, which is a potentially modifiable risk factor for GDM development.[53-57] Two RCTs using combined interventions in high-risk women (BMI ≥ 30kg/m^2), the UPBEAT[58] and RADIEL trial,[59] showed 0.55 kg and 0.58 kg reduction in GWG, respectively. The RADIEL trial additionally showed about 36% reduction in GDM incidence (crude RR 0.64; 95% CI 0.38–1.09) in the intervention group. A recent Cochrane review of combined interventions showed a tendency towards reduced GDM risk (RR 0.85, 95%CI 0.71-1.01; 6,633 women, 19 RCTs) and reduced weight gain (mean difference -0.89 kg, 95%CI -1.39 to -0.40; 5,052 women, 16RCTs).[60]

HYPOTHESIS

We hypothesize that daily yoghurt consumption and/or increased PA will reduce the risk of developing GDM in 'high-risk' pregnant women.

AIMS

To examine if daily yoghurt consumption and/or PA beginning from ≤16 weeks of gestation in 'high-risk' pregnant women can reduce the incidence of GDM in two LMIC settings.

OBJECTIVES

Primary objective

To establish the efficacy and safety of low-cost interventions (fermented yoghurt consumption/daily walking/both) to prevent GDM in two LMIC settings.

Secondary objectives

(i) To evaluate the effect of yoghurt/daily walking/combination of both on fasting glucose concentration at 32 weeks of gestation, GWG, blood pressure, intrapartum and neonatal outcomes.
(ii) To identify barriers that prevent participation/adherence to lifestyle changes during pregnancy and thus design culturally and socially acceptable, cost effective interventions that can be implemented successfully in LMICs.

CENTRES

Christian Medical College, Vellore, India
MRC Unit at LSHTM, Fajara, The Gambia

DESIGN

The study is a 2 x 2 factorial design and eligible women will be randomized to either one of the arms – (i) yoghurt (ii) PA (iii) yoghurt + PA (iv) standard care

For peer review only - http://bmjopen.bmj.com/site/about/guidelines.xhtml

ELIGIBILITY CRITERIA

The study will include pregnant women aged ≥18 years and GA ≤16 weeks and meeting at least one risk-factor for GDM (booking BMI ≥25kg/m^2, age ≥25 years; a first-degree relative with diabetes; a previous pregnancy complicated by GDM, pre-eclampsia/eclampsia and/or a large baby (≥3.5kg); a history of PCOS/impaired fasting glucose) and not currently on any medication that is known to interfere with glucose metabolism.

Exclusion criteria

- GDM diagnosed prior to screening visit based on IADPSG criteria or documented raised HbA1C, i.e., either fasting glucose ≥5.1 mmol/L or 1h glucose ≥10.0 mmol/L or 2h glucose ≥8.5 mmol/L, or a documented HbA1c of ≥6.5% at first booking
- History of pre-gestational diabetes, severe hyperemesis in the first trimester
- History of recurrent (≥2) first trimester spontaneous abortions, stillbirth, significant ante- or post-partum haemorrhage in previous pregnancies
- Multiple gestation in the current pregnancy
- Uncontrolled pre-gestational or gestational hypertension (BP>150/100 mmHg) on treatment
- Previous child born with congenital anomalies
- Pregnancy following in-vitro fertilization or any assisted reproductive technology
- Previous or current psychiatric illness on medication, epileptic seizures or on anti-epileptic medication
- Women meeting absolute contraindications for physical activity during pregnancy as recommended by the ACOG (heart disease, restrictive lung disease, incompetent cervix/cerclage, pregnancies at risk for premature labour, gestational hypertension, severe anaemia)

METHODS

Women attending obstetric outpatient clinics of the Christian Medical College, Vellore, India and MRC Unit in The Gambia operating in four centres in The Gambia (Brikama Major Health Centre, Bundung Maternal and Child Hospital, Barfrow Medical Centre and Edward Francis Small Teaching Hospital) will be informed about the study and assessed for eligibility. Following informed consent, women enrolled in the study will be asked to attend five scheduled visits (screening, run-in phase, randomisation, visits 1, 2 and 3) as shown in **Figure 2** and detailed in the protocol **(online supplementary file 1)**.

RANDOMISATION

Participants will be randomly allocated to one of the following arms (i) yoghurt (ii) PA (iii) yoghurt + PA or (iv) standard care following an allocation schedule pre-stratified for centre, age and BMI. A variable block randomisation, stratified by centre, and then by age, (<25 and ≥25 years) and BMI (<25 and ≥25 kg/m^2) will be generated, using a password-protected access database developed by an independent statistician not involved with trial participants. If a woman has more than one risk factor, she will be allocated to the strata depending on the first of the risk factors in the hierarchical block randomisation procedure (age or BMI). In India, the allocation will be performed using a computer interface (Access database), while separate computer-generated randomisation lists for each strata combination (age X BMI)

For peer review only - http://bmjopen.bmj.com/site/about/guidelines.xhtml

will be prepared for each health facility in the Gambia. Due to logistic limitations, this will be implemented using pre-issued opaque, sealed envelopes containing the allocation code.

INTERVENTIONS

Dietary intervention Yoghurt will be prepared locally at the dietary department in CMC, Vellore and at Kombo Dairy farm in The Gambia based on a common recipe and using the same starter culture containing *lactobacillus sp* and *Bifidobacterium sp* at 10^7 CFU. Yoghurt will be prepared in batches and an internal quality check will be performed to ensure that the bacterial species and colony count are maintained with each batch. Participants in the active yoghurt arm will consume 200 g/day of regular, unflavoured, fermented yoghurt throughout the active intervention period independent of the timing of their regular meal. Yoghurt will be dispensed either daily or weekly as feasible by the study centre and possibility of storage in refrigerator at participants' homes when not in use. Compliance with yoghurt will be assessed using pot count by field workers. All participants will receive dietary counselling, will not be advised to stop their regular dairy consumption, assuming that any effect on the outcome will be related to the trial yoghurt consumed. Information on background dairy consumption will be collected at each scheduled visit using a standardized DAIRY consumption questionnaire.

Physical activity intervention The PA activity intervention will include daily walking to increase the individual target step count, monitored using a wrist-mounted accelerometer device (Garmin Vivofit 4 fitness band).

The baseline step count will be recorded in all participants (blinding the step count on the device) for 7 days, with no emphasis on step count and/or activity during the run-in phase. At randomisation, baseline step count will be calculated as the average over 7 days including two weekend days. An individual target will be set, to a 40% or greater increase in daily step count compared with the baseline reading. The increment of 40% was based on a feasibility estimation. Systematic reviews have shown that simple pedometer interventions can lead to a sustained increase of around 2000-2500 steps a day in a great number of populations and settings.[61 62] The background step count in these populations was reasonably low (~5000), which will mean that 2000 additional step represent 40%.

The step count reading on the device will be unblinded after randomisation for women in the active PA arms, to provide visual feed back to the participants and to motivate them to achieve their daily target step count. Women in the non-PA arm will continue to use their fitness bands blinded throughout the study to measure their routine PA behaviour. Step count data will be periodically uploaded using Garmin application by the field worker to quantify compliance monitoring and counselling to participants in the PA arm. As the device is water resistant, the participants would be instructed to wear it day and night.

STUDY ASSESSMENT

Clinical measurements

Detailed study-related procedures, as outlined in **Table 1** and routine antenatal examination will be carried out at all study visits. All measurements will be standardized between the two study centres and will be carried out as detailed in study specific standard operating procedures (SOPs).

For peer review only - http://bmjopen.bmj.com/site/about/guidelines.xhtml

Table 1. Study-related procedures at each study visit

Procedures	Visits						
	Visit -2	Visit -1	Visit 0	Visit 1	Visit 2	Visit 3	Delivery
	Screening ≤16 weeks	Run-in-phase ≤17 weeks	Randomization Week ≤18	Week 18-20	Week 26-28	Week 32	
Informed consent	X						
Eligibility assessment	X						
Demographics	X						
History	X						
Height	X						
Weight	X	X	X	X	X	X	X
Blood pressure	X	X	X	X	X	X	X
Antenatal examination	X	X	X	X	X	X	
Dairy consumption questionnaire		X	X	X	X	X	
Barriers questionnaire	X					X	
Baseline PA monitoring with Vivofit 4		X					
PA step-count monitoring			X	X	X	X	
Randomisation			X				
OGTT	X				X		
Dating scan	X						
Morphology scan				X			
Ultrasound scan (growth)						X	
Fasting plasma glucose						X	
Intrapartum assessment (mother)							X
New born assessment							X

For peer review only

Laboratory tests

Study participants will undergo a 75g OGTT at screening and visit 2 (week 26-28 GA) following an 8 hour overnight fast. Additional fasting plasma glucose will be measured at the end-of-

For peer review only - http://bmjopen.bmj.com/site/about/guidelines.xhtml

study visit (week 32). Blood glucose measurements will be analysed using a Roche Cobas 800 autoanalyser at respective laboratories (Department of Clinical Biochemistry in Vellore, India and the MRCG at LSHTM, The Gambia). Other routine ante-natal laboratory tests, including haemoglobin will be done as a part of routine antenatal care according to local guidelines.

GDM ascertainment

IADPSG criteria will be used to diagnose GDM using an OGTT at week 26-28. GDM diagnosis will be established if either fasting glucose ≥5.1 mmol/L or 1h glucose ≥10.0 mmol/L or 2h glucose ≥8.5 mmol/L.

Questionnaire based assessments

Standardized questionnaires will be used to obtain information on social demographics, background dairy consumption and to identify barriers to lifestyle intervention during pregnancy in all participating women. The barriers questionnaire is specifically designed to address ethnic-specific social and cultural barriers to lifestyle intervention during pregnancy in both populations and would be administered at the start and the end of the study. The DAIRY consumption questionnaire used in this study is a modification the Dutch Dairy Food Frequency Questionnaire (Wageningen University, Department Human Nutrition, 2005)[63]. The questions relate to consumption of various dairy products divided into 6 different groups: total dairy, cheese, milk, nonfermented milk products, fermented milk, and other dairy products. Flavoured milk, puddings, and cream are all part of the nonfermented milk product group. The fermented milk products consist of yogurt, yogurt drinks, and butter milk. Additional modifications to include other dairy preparations that are locally consumed in both countries are included.

Ultrasound scans

Ultrasound scans will be performed by an ultrasound-trained obstetrician on Voluson GE S-8 (in India) and Aloka USI-145 (in Gambia) ultrasound machines on all participates at three visits, i) at screening (dating) ii) morphology scan at visit 1 (18-20 weeks GA) and iii) at visit 3 (week 32) for growth monitoring. Both centres will follow standard protocols as described in study standard operating procedures (SOPs). Evidence of foetal congenital anomaly, placental malformation or malposition or abnormal ultrasound features that can compromise pregnancy outcome will lead to termination of the participant's involvement in the study and referral for the normal care pathway at each participating centre.

OUTCOMES

Primary outcome

Incidence of GDM diagnosed using IADPSG criteria between 26-28 weeks of gestation or if the fasting plasma glucose concentration is ≥5.1 mmol/l at 32 weeks of gestation.

Secondary outcomes

Effect of the intervention on fasting blood glucose concentration at 32 weeks, gestational weight gain and blood pressure from randomisation to week 32, proportion of women undergoing instrumental/caesarean delivery, proportion of women developing pre-eclampsia and eclampsia, blood loss during delivery (subjective assessment by the obstetrician), post-

For peer review only - http://bmjopen.bmj.com/site/about/guidelines.xhtml

partum haemorrhage, pre-term births (<37 weeks GA), foetal macrosomia defined as birth weight >2 SDs above the population-specific mean in each setting, birth weight and length, APGAR score at 1 and 5 minutes and responses to questionnaire based assessments of barriers to lifestyle interventions in pregnancy.

ADVERSE (AE)/SERIOUS ADVERSE EVENTS (SAEs) AND REPORTING

All AE and SAEs will be captured in respective electronic CRFs. Details of all events including onset date, relationship to the study intervention, treatment given, and stop date will be captured in the respective AE report form. The PI will report all SAEs without filtration, whether or not related to the intervention, within 24 hours of becoming aware of the event, to the sponsor. All SAEs will be notified to the respective Ethics Committee within seven calendar days if fatal or life-threatening, and all others within 15 calendar days. All AEs and SAEs will also be notified to the Data Safety and Monitoring Committee (DSMC). Expected SAEs which are protocol-defined exceptions to SAE reporting will be captured and reported to respective ECs and DSMC as outlined in the protocol **(online supplementary file 1)**

STATISTICAL ANALYSIS PLAN INCLUDING SAMPLE SIZE AND POWER CALCULATION

Sample size

The sample size calculation was performed by simulation (in R version 3.5.2) using an algorithm as described in detail in the protocol (**online supplementary file 1**). After 10,000 repetitions, we estimated the sample size to be 1856 high-risk women (928 from each centre). The sample size is estimated to have 80% power to detect a 33% reduction in the incidence of GDM, for the main effects of diet and PA, with a family-wise significance level of 5% for a two-sided test (i.e., each effect was tested at a significance level of 2.5%). It was assumed that participants could be pooled across centres (no interaction between interventions and country at the 5% level). The sample size was inflated to allow for a 15% drop out rate in each of the four strata, with drop outs defined as non-compliant participants (i.e. no risk reduction in their incidence of GDM).

Data analysis

Data will be analysed using both (1) the intention-to-treat principle, including all enrolled participants regardless of their adherence to the proposed intervention, and (2) a per protocol approach, including only those participants adhering to at least 75% of the proposed intervention. In addition, we will run a sensitivity analysis to assess the effect of protocol deviation compared to 100% adherence.

Descriptive analyses at baseline

We will describe baseline characteristics of the four groups (Control, yoghurt, PA, and yoghurt + PA) using means and standard deviations (normally distributed quantitative variables), medians and interquartile ranges (non-normally distributed quantitative variables), or proportions (categorical variables).

Primary analysis

For our primary outcome (presence or absence of GDM), we will use multiple logistic regression models and results presented as OR and 95% CI. We will examine the assumption

For peer review only - http://bmjopen.bmj.com/site/about/guidelines.xhtml

that there are no interaction effects between study centre and each of the interventions by fitting a multiple logistic regression model.

We will build two covariate adjusted models. First, we will assess the relative difference between centres in the effects of each intervention on GDM by adding a term for study centre. Second, we will fit a covariate (age, pre-pregnancy BMI, previous history of GDM and GA) adjusted model adding further terms for each, as well as relevant interaction terms, to remove bias from the estimate of the treatment effect on the primary outcome. Each covariate will be added to the unadjusted model separately first and then added one at a time and kept in the model if their significance level is <10%.

Secondary analyses

Continuous variables will be analysed using multiple linear regression and binary variables by multiple logistic regression. For each secondary outcome, we will fit a simple model with only terms for each intervention, and a covariate adjusted model including terms for study centre and the remaining pre-specified covariates and relevant interactions.

DATA COLLECTION

Research data will be collected using electronic CRFs (eCRFs) on tablets by designated study staff after training. Where there is internet connectivity, collected data will be stored directly onto the central server housed at MRC, Fajara and where there is no/unreliable connectivity, data will be stored locally on the tablet and later synchronized to the central servers. The accelerometer data used for PA monitoring will be directly synced with the Garmin app on the tablets on a weekly basis and transferred to eCRFs. To ensure standardisation of processes, SOPs with respect to trial management, quality assurance, data management, IT & security, and statistics will be adhered to.

COMMITTEE OVERSIGHTS

The study will be conducted in accordance with the approved protocol, Good Clinical Practice regulations and SOPs. The implementation of the study will be overseen by the Trial Steering committee (TSC). An independent DSMC, comprised of experts in clinical trials/epidemiology, biostatistics and obstetrics will meet 6-monthly to review the progress of the trial (recruitment, compliance, loss to follow-up), clinical study safety data, data quality and assess and/or adjudicate all events of deaths, the GDM event rate and key secondary/safety outcomes and provide feedback to the TSC. The DSMC will also meet if required, more frequently depending on the enrolment rate, safety issues and AEs. A copy of the full study protocol and all amendments would be submitted to DSMC prior to study initiation and subsequently. Thereafter, periodic reports including summary of enrolment, demographics, protocol compliance and safety will be compiled by the study statistician and submitted at least one week prior to the scheduled meeting. The board will advise on continuation or stopping the trial based upon safety and efficacy.

ETHICS AND DISSEMINATION

Ethical issues

The proposed interventions are considered generally safe both for maternal and foetal health. Participation in this trial is voluntary and subject to informed consent. A sample informed consent statement is available in the study protocol (**online supplementary file 1).**

For peer review only - http://bmjopen.bmj.com/site/about/guidelines.xhtml

Participants who develop GDM at the visit 2 OGTT will be started on pharmacological therapy (as per local management guidelines) and results will be analysed as per intention to treat. Research participants can withdraw from the study at any time during the trial. The SPIRIT Checklist for the protocol is detailed in **online supplementary file 2.**

Dissemination

Results of the study will be presented at major national and international conferences, and publish in peer-reviewed journals, ensuring open access and the highest possible impact. The de-identified data will be openly available to the scientific community after publication of initial results. We will interact with national and international health agencies and governments to stimulate translation of our findings into policy and expect our research to influence policy for mothers and children within the next 5-10 years.

Patient and public involvement

The trial was informed by patients' need for effective preventive therapies for GDM since randomised trial data are lacking in these populations. Patients were not involved in the design, conduct or reporting of the study. A summary of the main trial results will be disseminated to the general public and study participants after the completion of the study.

DISCUSSION

GDM poses increased risks to maternal and child health. LMICs face numerous challenges to GDM care at various levels. These include (i) late second trimester screening for GDM, ii) lack of standardized nation-wide guidelines for diagnosis, iii) interventions targeting only women who are diagnosed with GDM around 24-28 weeks, iv) lack of knowledge and awareness of GDM and its health implications and v) sociocultural barriers to early antenatal care and life-style interventions during pregnancy. It seems very likely that early screening and establishment of positive lifestyle changes beginning from conception, or even prior to conception, particularly in selected high-risk women, and especially in low-resource settings, will reduce the burden of GDM as well as improve both immediate obstetric outcomes and long term health of the mother and the new born. Primary prevention of GDM, rather than treatment initiated after GDM onset is likely to lead to both economic and individual health benefits.

The PRIMORDIAL study is timely given the increasing prevalence of both diabetes and obesity in countries like Africa and India. Our study provides a unique opportunity to test simple life-style interventions during pregnancy in the LMIC settings, which have never been undertaken. The results will add to the existing evidence of beneficial effects of yoghurt on several pregnancy related outcomes.

The study is expected to bridge a gap in knowledge of the potential effectiveness of GDM prevention in two LMIC settings and create opportunities for capacity building in both India and The Gambia. We believe the trial can provide information that will aid in policy guidelines relating to GDM screening and management and also suggest areas that may require further research.

Contributors SKV and FK conceived and designed the study. SKV, DT, CHDF, CO, SJU, AMP and FK drafted the original grant proposal and trial protocol. CO, FC and AJ provided

For peer review only - http://bmjopen.bmj.com/site/about/guidelines.xhtml

methodological and data analysis plan. JM, SR, MJ, MB, and AMP provided expertise in the pregnancy clinical outcomes. DT, SJU, CHDF and AMP provided expertise in study interventions. FC and AJ will contribute significantly to the data management and central data monitoring. SKV drafted the original protocol. SKV, MJ, OJ, MB, JM, SR have responsibilities for day-to-day running of the trial including participant recruitment, data collection and liaising with other sites. SKV and FK drafted the first version of this manuscript. All authors critically reviewed and approved the final version of the manuscript. All authors agreed to be accountable for all aspects of the work.

Funding This study is supported by grants jointly from Medical Research Council, UK (MR/R020345/1), and Newton Fund- Director of Biotechnology, India (BT/IN/DBT-MRC/DIFD/JM/12/2018-19) The study is partly supported by the Global Challenges Research fund, University of Oxford, UK (0006138)

Disclaimer The views expressed in this publication are those of the author(s) and not necessarily those of the MRC, UK, GCRF, UK, DBT, India, healthcare systems or competent authorities.

Competing interests None declared.

Ethics approval This study will be conducted in accordance with the principles of Good Clinical Practice. The study is approved by Oxford Tropical Research Ethics Committee (OxTREC 44-18), ethics committees of Christian Medical College, Vellore (IRB 11367), Scientific co-ordinating committee, The Gambia (SCC 1645) and The Gambia Government/MRCG joint ethics committee (L2020.E15).

Provenance and peer review Not commissioned; externally peer reviewed.

REFERENCES

1. Cho NH, Shaw JE, Karuranga S, et al. IDF Diabetes Atlas: Global estimates of diabetes prevalence for 2017 and projections for 2045. *Diabetes Res Clin Pract* 2018;138:271-81. doi: 10.1016/j.diabres.2018.02.023
2. Buchanan TA, Xiang AH. Gestational diabetes mellitus. *J Clin Invest* 2005;115(3):485-91. doi: 10.1172/JCI24531 [published Online First: 2005/03/15]
3. Reece EA, Leguizamon G, Wiznitzer A. Gestational diabetes: the need for a common ground. *Lancet* 2009;373(9677):1789-97. doi: 10.1016/S0140-6736(09)60515-8
4. Yu Y, Arah OA, Liew Z, et al. Maternal diabetes during pregnancy and early onset of cardiovascular disease in offspring: population based cohort study with 40 years of follow-up. *BMJ* 2019;367:l6398. doi: 10.1136/bmj.l6398
5. Sacks DA, Hadden DR, Maresh M, et al. Frequency of gestational diabetes mellitus at collaborating centers based on IADPSG consensus panel-recommended criteria: the Hyperglycemia and Adverse Pregnancy Outcome (HAPO) Study. *Diabetes Care* 2012;35(3):526-8. doi: 10.2337/dc11-1641 [published Online First: 2012/02/23]

For peer review only - http://bmjopen.bmj.com/site/about/guidelines.xhtml

6. Wahi P, Dogra V, Jandial K, et al. Prevalence of gestational diabetes mellitus (GDM) and its outcomes in Jammu region. *J Assoc Physicians India* 2011;59:227-30.
7. Seshiah V, Balaji V, Balaji MS, et al. Pregnancy and diabetes scenario around the world: India. *Int J Gynaecol Obstet* 2009;104 Suppl 1:S35-8. doi: 10.1016/j.ijgo.2008.11.035
8. Macaulay S, Dunger DB, Norris SA. Gestational diabetes mellitus in Africa: a systematic review. *PLoS One* 2014;9(6):e97871. doi: 10.1371/journal.pone.0097871
9. Mwanri AW, Kinabo J, Ramaiya K, et al. Gestational diabetes mellitus in sub-Saharan Africa: systematic review and metaregression on prevalence and risk factors. *Trop Med Int Health* 2015;20(8):983-1002. doi: 10.1111/tmi.12521
10. Jiwani A, Marseille E, Lohse N, et al. Gestational diabetes mellitus: results from a survey of country prevalence and practices. *J Matern Fetal Neonatal Med* 2012;25(6):600-10. doi: 10.3109/14767058.2011.587921
11. Zhu Y, Zhang C. Prevalence of Gestational Diabetes and Risk of Progression to Type 2 Diabetes: a Global Perspective. *Curr Diab Rep* 2016;16(1):7. doi: 10.1007/s11892-015-0699-x
12. Catalano PM, Tyzbir ED, Roman NM, et al. Longitudinal changes in insulin release and insulin resistance in nonobese pregnant women. *Am J Obstet Gynecol* 1991;165(6 Pt 1):1667-72. doi: 10.1016/0002-9378(91)90012-g
13. Catalano PM. Obesity, insulin resistance, and pregnancy outcome. *Reproduction* 2010;140(3):365-71. doi: 10.1530/REP-10-0088
14. Benhalima K, Lens K, Bosteels J, et al. The Risk for Glucose Intolerance after Gestational Diabetes Mellitus since the Introduction of the IADPSG Criteria: A Systematic Review and Meta-Analysis. *J Clin Med* 2019;8(9) doi: 10.3390/jcm8091431
15. Ferrara A. Increasing prevalence of gestational diabetes mellitus: a public health perspective. *Diabetes Care* 2007;30 Suppl 2:S141-6. doi: 10.2337/dc07-s206
16. Group HSCR, Metzger BE, Lowe LP, et al. Hyperglycemia and adverse pregnancy outcomes. *N Engl J Med* 2008;358(19):1991-2002. doi: 10.1056/NEJMoa0707943
17. Lowe LP, Metzger BE, Dyer AR, et al. Hyperglycemia and Adverse Pregnancy Outcome (HAPO) Study: associations of maternal A1C and glucose with pregnancy outcomes. *Diabetes Care* 2012;35(3):574-80. doi: 10.2337/dc11-1687
18. International Association of D, Pregnancy Study Groups Consensus P, Metzger BE, et al. International association of diabetes and pregnancy study groups recommendations on the diagnosis and classification of hyperglycemia in pregnancy. *Diabetes Care* 2010;33(3):676-82. doi: 10.2337/dc09-1848
19. Berger H, Sermer M. Counterpoint: Selective screening for gestational diabetes mellitus. *Diabetes Care* 2009;32(7):1352-4. doi: 10.2337/dc09-0361
20. Ostlund I, Hanson U. Occurrence of gestational diabetes mellitus and the value of different screening indicators for the oral glucose tolerance test. *Acta Obstet Gynecol Scand* 2003;82(2):103-8. doi: 10.1034/j.1600-0412.2003.00001.x
21. Guo XY, Shu J, Fu XH, et al. Improving the effectiveness of lifestyle interventions for gestational diabetes prevention: a meta-analysis and meta-regression. *BJOG* 2019;126(3):311-20. doi: 10.1111/1471-0528.15467
22. Agha-Jaffar R, Oliver N, Johnston D, et al. Gestational diabetes mellitus: does an effective prevention strategy exist? *Nat Rev Endocrinol* 2016;12(9):533-46. doi: 10.1038/nrendo.2016.88

For peer review only - http://bmjopen.bmj.com/site/about/guidelines.xhtml

23. Thangaratinam S, Rogozinska E, Jolly K, et al. Effects of interventions in pregnancy on maternal weight and obstetric outcomes: meta-analysis of randomised evidence. *BMJ* 2012;344:e2088. doi: 10.1136/bmj.e2088
24. Aune D, Norat T, Romundstad P, et al. Dairy products and the risk of type 2 diabetes: a systematic review and dose-response meta-analysis of cohort studies. *Am J Clin Nutr* 2013;98(4):1066-83. doi: 10.3945/ajcn.113.059030
25. Elwood PC, Pickering JE, Givens DI, et al. The consumption of milk and dairy foods and the incidence of vascular disease and diabetes: an overview of the evidence. *Lipids* 2010;45(10):925-39. doi: 10.1007/s11745-010-3412-5
26. Grantham NM, Magliano DJ, Hodge A, et al. The association between dairy food intake and the incidence of diabetes in Australia: the Australian Diabetes Obesity and Lifestyle Study (AusDiab). *Public Health Nutr* 2013;16(2):339-45. doi: 10.1017/S1368980012001310
27. Tong X, Dong JY, Wu ZW, et al. Dairy consumption and risk of type 2 diabetes mellitus: a meta-analysis of cohort studies. *Eur J Clin Nutr* 2011;65(9):1027-31. doi: 10.1038/ejcn.2011.62 [published Online First: 2011/05/12]
28. Veiga P, Pons N, Agrawal A, et al. Changes of the human gut microbiome induced by a fermented milk product. *Sci Rep* 2014;4:6328. doi: 10.1038/srep06328
29. Hill C, Guarner F, Reid G, et al. Expert consensus document. The International Scientific Association for Probiotics and Prebiotics consensus statement on the scope and appropriate use of the term probiotic. *Nat Rev Gastroenterol Hepatol* 2014;11(8):506-14. doi: 10.1038/nrgastro.2014.66
30. Wen L, Duffy A. Factors Influencing the Gut Microbiota, Inflammation, and Type 2 Diabetes. *J Nutr* 2017;147(7):1468S-75S. doi: 10.3945/jn.116.240754
31. Gomez-Arango LF, Barrett HL, McIntyre HD, et al. Connections Between the Gut Microbiome and Metabolic Hormones in Early Pregnancy in Overweight and Obese Women. *Diabetes* 2016;65(8):2214-23. doi: 10.2337/db16-0278
32. Crusell MKW, Hansen TH, Nielsen T, et al. Gestational diabetes is associated with change in the gut microbiota composition in third trimester of pregnancy and postpartum. *Microbiome* 2018;6(1):89. doi: 10.1186/s40168-018-0472-x
33. Fox C, Eichelberger K. Maternal microbiome and pregnancy outcomes. *Fertil Steril* 2015;104(6):1358-63. doi: 10.1016/j.fertnstert.2015.09.037
34. Ruan Y, Sun J, He J, et al. Effect of Probiotics on Glycemic Control: A Systematic Review and Meta-Analysis of Randomized, Controlled Trials. *PLoS One* 2015;10(7):e0132121. doi: 10.1371/journal.pone.0132121
35. Gohir W, Ratcliffe EM, Sloboda DM. Of the bugs that shape us: maternal obesity, the gut microbiome, and long-term disease risk. *Pediatr Res* 2015;77(1-2):196-204. doi: 10.1038/pr.2014.169
36. Lindsay KL, Walsh CA, Brennan L, et al. Probiotics in pregnancy and maternal outcomes: a systematic review. *J Matern Fetal Neonatal Med* 2013;26(8):772-8. doi: 10.3109/14767058.2012.755166
37. Chen X, Jiang X, Huang X, et al. Association between Probiotic Yogurt Intake and Gestational Diabetes Mellitus: A Case-Control Study. *Iran J Public Health* 2019;48(7):1248-56.
38. Luoto R, Laitinen K, Nermes M, et al. Impact of maternal probiotic-supplemented dietary counselling on pregnancy outcome and prenatal and postnatal growth: a double-

For peer review only - http://bmjopen.bmj.com/site/about/guidelines.xhtml

blind, placebo-controlled study. *Br J Nutr* 2010;103(12):1792-9. doi: 10.1017/S0007114509993898
39. Laitinen K, Poussa T, Isolauri E, et al. Probiotics and dietary counselling contribute to glucose regulation during and after pregnancy: a randomised controlled trial. *Br J Nutr* 2009;101(11):1679-87. doi: 10.1017/S0007114508111461
40. Ilmonen J, Isolauri E, Poussa T, et al. Impact of dietary counselling and probiotic intervention on maternal anthropometric measurements during and after pregnancy: a randomized placebo-controlled trial. *Clin Nutr* 2011;30(2):156-64. doi: 10.1016/j.clnu.2010.09.009
41. Sahhaf Ebrahimi F, Homayouni Rad A, Mosen M, et al. Effect of L. acidophilus and B. lactis on blood glucose in women with gestational diabetes mellitus: a randomized placebo-controlled trial. *Diabetol Metab Syndr* 2019;11:75. doi: 10.1186/s13098-019-0471-5
42. Asgharian H, Homayouni-Rad A, Mirghafourvand M, et al. Effect of probiotic yoghurt on plasma glucose in overweight and obese pregnant women: a randomized controlled clinical trial. *Eur J Nutr* 2019 doi: 10.1007/s00394-019-01900-1
43. Asemi Z, Samimi M, Tabassi Z, et al. Effect of daily consumption of probiotic yoghurt on insulin resistance in pregnant women: a randomized controlled trial. *Eur J Clin Nutr* 2013;67(1):71-4. doi: 10.1038/ejcn.2012.189
44. Kennedy JW, Hirshman MF, Gervino EV, et al. Acute exercise induces GLUT4 translocation in skeletal muscle of normal human subjects and subjects with type 2 diabetes. *Diabetes* 1999;48(5):1192-7. doi: 10.2337/diabetes.48.5.1192
45. Perseghin G, Price TB, Petersen KF, et al. Increased glucose transport-phosphorylation and muscle glycogen synthesis after exercise training in insulin-resistant subjects. *N Engl J Med* 1996;335(18):1357-62. doi: 10.1056/NEJM199610313351804
46. Artal R, O'Toole M. Guidelines of the American College of Obstetricians and Gynecologists for exercise during pregnancy and the postpartum period. *Br J Sports Med* 2003;37(1):6-12; discussion 12. doi: 10.1136/bjsm.37.1.6
47. Aune D, Sen A, Henriksen T, et al. Physical activity and the risk of gestational diabetes mellitus: a systematic review and dose-response meta-analysis of epidemiological studies. *Eur J Epidemiol* 2016;31(10):967-97. doi: 10.1007/s10654-016-0176-0
48. Clapp JF, 3rd, Lopez B, Harcar-Sevcik R. Neonatal behavioral profile of the offspring of women who continued to exercise regularly throughout pregnancy. *Am J Obstet Gynecol* 1999;180(1 Pt 1):91-4. doi: 10.1016/s0002-9378(99)70155-9
49. Nasiri-Amiri F, Sepidarkish M, Shirvani MA, et al. The effect of exercise on the prevention of gestational diabetes in obese and overweight pregnant women: a systematic review and meta-analysis. *Diabetol Metab Syndr* 2019;11:72. doi: 10.1186/s13098-019-0470-6
50. Oostdam N, van Poppel MN, Wouters MG, et al. No effect of the FitFor2 exercise programme on blood glucose, insulin sensitivity, and birthweight in pregnant women who were overweight and at risk for gestational diabetes: results of a randomised controlled trial. *BJOG* 2012;119(9):1098-107. doi: 10.1111/j.1471-0528.2012.03366.x
51. Han S, Middleton P, Crowther CA. Exercise for pregnant women for preventing gestational diabetes mellitus. *Cochrane Database Syst Rev* 2012(7):CD009021. doi: 10.1002/14651858.CD009021.pub2
52. Stafne SN, Salvesen KA, Romundstad PR, et al. Regular exercise during pregnancy to prevent gestational diabetes: a randomized controlled trial. *Obstet Gynecol* 2012;119(1):29-36. doi: 10.1097/AOG.0b013e3182393f86

For peer review only - http://bmjopen.bmj.com/site/about/guidelines.xhtml

53. Vinter CA, Tanvig MH, Christensen MH, et al. Lifestyle Intervention in Danish Obese Pregnant Women With Early Gestational Diabetes Mellitus According to WHO 2013 Criteria Does Not Change Pregnancy Outcomes: Results From the LiP (Lifestyle in Pregnancy) Study. *Diabetes Care* 2018;41(10):2079-85. doi: 10.2337/dc18-0808
54. Simmons D, Jelsma JG, Galjaard S, et al. Results From a European Multicenter Randomized Trial of Physical Activity and/or Healthy Eating to Reduce the Risk of Gestational Diabetes Mellitus: The DALI Lifestyle Pilot. *Diabetes Care* 2015;38(9):1650-6. doi: 10.2337/dc15-0360
55. Renault KM, Norgaard K, Nilas L, et al. The Treatment of Obese Pregnant Women (TOP) study: a randomized controlled trial of the effect of physical activity intervention assessed by pedometer with or without dietary intervention in obese pregnant women. *Am J Obstet Gynecol* 2014;210(2):134 e1-9. doi: 10.1016/j.ajog.2013.09.029
56. Asbee SM, Jenkins TR, Butler JR, et al. Preventing excessive weight gain during pregnancy through dietary and lifestyle counseling: a randomized controlled trial. *Obstet Gynecol* 2009;113(2 Pt 1):305-12. doi: 10.1097/AOG.0b013e318195baef
57. International Weight Management in Pregnancy Collaborative G. Effect of diet and physical activity based interventions in pregnancy on gestational weight gain and pregnancy outcomes: meta-analysis of individual participant data from randomised trials. *BMJ* 2017;358:j3119. doi: 10.1136/bmj.j3119
58. Poston L, Bell R, Croker H, et al. Effect of a behavioural intervention in obese pregnant women (the UPBEAT study): a multicentre, randomised controlled trial. *Lancet Diabetes Endocrinol* 2015;3(10):767-77. doi: 10.1016/S2213-8587(15)00227-2
59. Koivusalo SB, Rono K, Klemetti MM, et al. Gestational Diabetes Mellitus Can Be Prevented by Lifestyle Intervention: The Finnish Gestational Diabetes Prevention Study (RADIEL): A Randomized Controlled Trial. *Diabetes Care* 2016;39(1):24-30. doi: 10.2337/dc15-0511
60. Shepherd E, Gomersall JC, Tieu J, et al. Combined diet and exercise interventions for preventing gestational diabetes mellitus. *Cochrane Database Syst Rev* 2017;11:CD010443. doi: 10.1002/14651858.CD010443.pub3
61. Bravata DM, Smith-Spangler C, Sundaram V, et al. Using pedometers to increase physical activity and improve health: a systematic review. *JAMA* 2007;298(19):2296-304. doi: 10.1001/jama.298.19.2296
62. Mansi S, Milosavljevic S, Baxter GD, et al. A systematic review of studies using pedometers as an intervention for musculoskeletal diseases. *BMC Musculoskelet Disord* 2014;15:231. doi: 10.1186/1471-2474-15-231
63. Vissers PA, Streppel MT, Feskens EJ, et al. The contribution of dairy products to micronutrient intake in the Netherlands. *J Am Coll Nutr* 2011;30(5 Suppl 1):415S-21S. doi: 10.1080/07315724.2011.10719985

For peer review only - http://bmjopen.bmj.com/site/about/guidelines.xhtml

Figure Legends

Figure 1

Relationship between pancreatic beta cell plasticity with advancement in gestational age

Figure 2

Flow chart of the study recruitment and scheduled visits

For peer review only

For peer review only - http://bmjopen.bmj.com/site/about/guidelines.xhtml

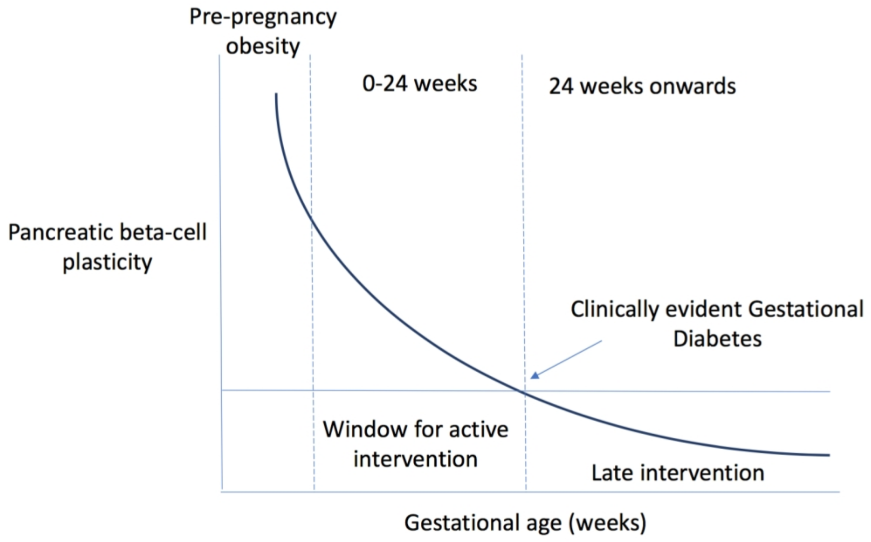

Figure 1. Relationship between pancreatic beta cell plasticity with advancement in gestational age

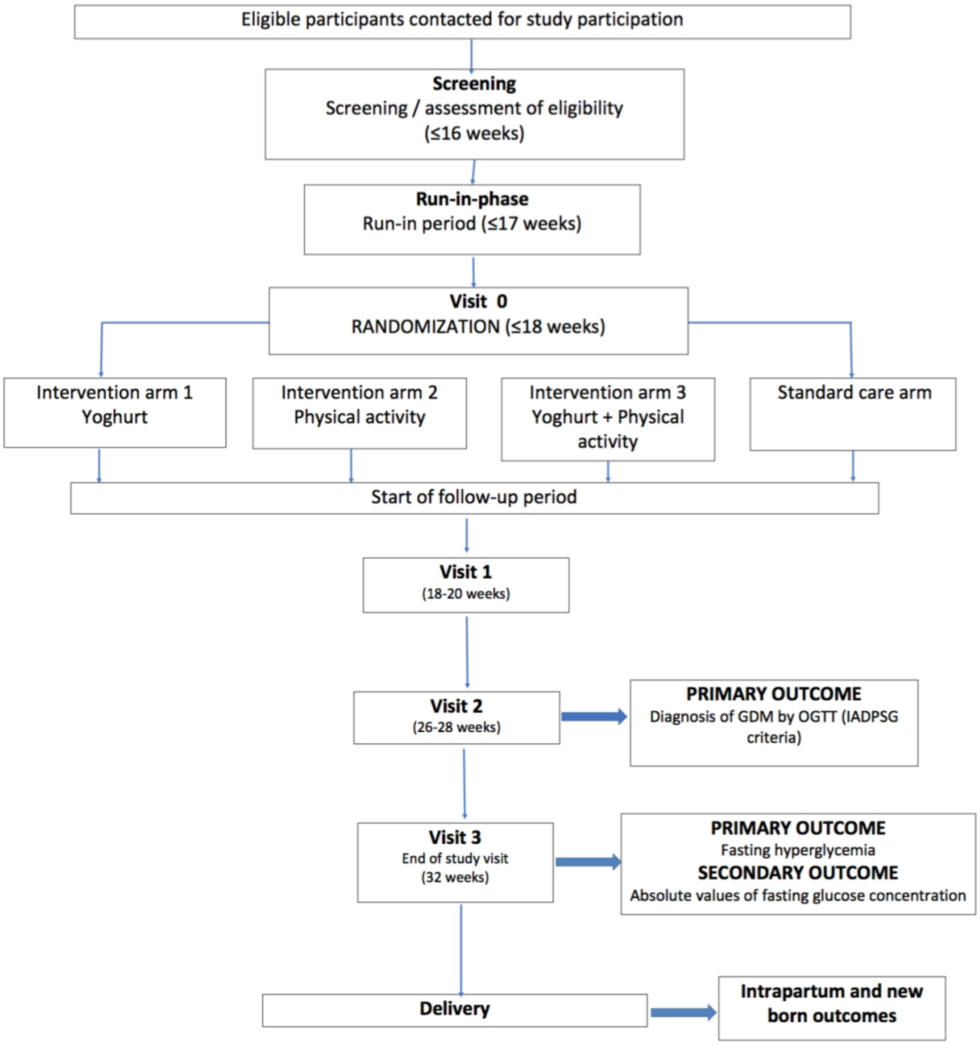

Figure 2. Flowchart of study recruitment and scheduled visits

Study Title: Pregnancy Related Interventions in Mothers at Risk for gestational Diabetes in Asian India and Low- and middle income countries

Internal Reference Number / Short title: PRIMORDIAL Study

OxTREC Ref: 44-18

Date and Version No: 18th July 2019. Version 4.2

Principal Investigator: Fredrik Karpe
Oxford Centre for Diabetes, Endocrinology and Metabolism (OCDEM)
University of Oxford
Churchill Hospital, Oxford OX3 7LE, UK
Phone: 0044 (0)1865 857222

Investigators: Andrew Prentice
MRC Unit The Gambia &
MRC International Nutrition Group,
London School of Hygiene & Tropical Medicine,
Keppel Street, London, WC1E 7HT, UK.

Jiji Elizabeth Matthews
Department of Obstetrics & Gynaecology – Unit 5
Christian Medical College & Hospital, Vellore -632004
Tamil Nadu, India

Sponsor: University of Oxford
Funder: Medical Research Council, UK

Principal Investigator Signature:

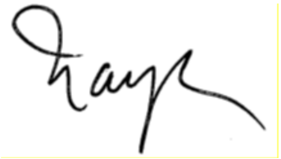

Trial Registration: ISRCTN: 18467720 and Clinical Trials Registry, India (CTRI/2018/07/014947)

Protocol written by: Senthil K Vasan and Fredrik Karpe, OCDEM, University of Oxford Churchill Hospital, Oxford OX3 7LE, UK

Conflicts of interest: None

Confidentiality Statement

This document contains confidential information that must not be disclosed to anyone other than the authorised individuals from the University of Oxford, the Investigator Team and members of the Oxford Tropical Research Ethics Committee (OxTREC), unless authorised to do so.

For peer review only

TABLE OF CONTENTS

1. SYNOPSIS 5
2. ABBREVIATIONS 7
3. BACKGROUND AND RATIONALE 9
4. OUTCOME MEASURES 17
5. STUDY DESIGN 18
6. PARTICIPANT IDENTIFICATION AND RECRUITMENT 20
   6.1. Study Participants 20
   6.2. Inclusion Criteria 21
   6.3. Exclusion Criteria 21
7. STUDY PROCEDURES 21
   7.1. Recruitment 22
   7.2. Informed Consent 23
   7.3. Screening and Eligibility Assessment: 23
   7.4. Randomisation 24
   7.5. Baseline Assessments 24
   7.6. Subsequent Visits 26
   7.6.1. Visit 1 (week 18-20) 26
   7.6.2. Visit 2 (week 26-28) 26
   7.6.3. Visit 3 (Week 32) - END OF STUDY VISIT 27
   7.6.4. Post-intervention surveillance visit 27
   7.7. Sample Handling 28
   7.8. Discontinuation/Withdrawal 28
   7.9. Lost to follow-up 30
   7.10. Protocol deviation 30
   7.11. Definition of End of Study 30
8. INTERVENTIONS 30
9. SAFETY REPORTING 32
   9.1. Adverse event reporting 32
   9.2. Definition of Serious Adverse Events 32
   9.3. Reporting Procedures for Serious Adverse Events 33
10. STATISTICS AND ANALYSIS 33
   10.1. Description of Statistical Methods 33
   10.2. The Number of Participants 34

For peer review only

10.3. Analysis of Outcome Measures 35
11. DATA MANAGEMENT 36
11.1. Types of data 36
11.2. Format and scale of data 36
11.3. Data collection/generation 37
11.4. Data quality and standards 37
11.5. Managing, storing and curating data 37
11.6. Data preservation strategy and standards 38
11.7. Main risks to data security 38
11.8. Suitability for sharing 38
11.9. Access to Data 38
11.10. Data Handling and Record Keeping 39
12. COMMITTEES STRUCTURES 39
12.1. DATA SAFETY MONITORING COMMITTEE (DSMC) 39
12.2. Trial Steering committee (TSC) 40
12.3. Executive committee 41
13. ETHICAL AND REGULATORY CONSIDERATIONS 41
13.1. ICH-GCP 41
13.2. Approvals 42
13.3. Participant Confidentiality 42
13.4. Expenses and Benefits 42
13.5. Annual Progress Report 42
13.6. Other Ethical Considerations 42
14. FINANCE AND INSURANCE 42
14.1. Funding 42
14.2. Insurance 43
15. PUBLICATION POLICY 43
16. REFERENCES 44
18. APPENDIX A: STUDY FLOW CHART 47
19. APPENDIX B: STUDY TIMELINES (approximate) 48
20. APPENDIX C: SCHEDULE OF STUDY PROCEDURES 49
21. APPENDIX D: PATIENT INFORMATION SHEET 50
22. APPENDIX E: INFORMED CONSENT FORM 53
23. APPENDIX F: AMENDMENT HISTORY 54

For peer review only

For peer review only

1. SYNOPSIS

**Long Study Title**	**Pr**egnancy **I**nterventions **I**n **M**others **R**elating to **D**iabetes In **A**sian India and **L**ow-income countries	
**Short Study Title (to be used on participant-facing documents, if applicable)**	PRIMORDIAL Study	
**Nature of Study Participants**	Pregnant women with high-risk for developing gestational diabetes	
**Intended number of participants**	1,875 high-risk women (~937 from each centre)	
**Planned Study Period**	36 months	
	**Objectives**	**Outcome Measures**
**Primary**	To determine the efficacy of life-style interventions (yoghurt consumption and physical actvity), given to pregnant women at high risk of GDM , in reducing the incidence of GDM.	Women will be diagnosed to have GDM if one or more plasma glucose concentrations following a 75g OGTT equals or exceeds the following: fasting glucose ≥5.1 mmol/l (92 mg/dl) and/or 1h glucose ≥10.0 mmol/l (180 mg/dl) and/or 2h glucose ≥8.5 mmol/l (153 mg/dl) at 26-28 weeks gestation or if the fasting plasma glucose concentration is ≥5.1 mmol/l at 32 weeks of gestation
**Secondary**	To determine the effect of life-style intervention (yoghurt and PA), given to women at high risk of GDM , on fasting blood glucose concentrations..	Absolute concentration of fasting blood glucose at 26-28 weeks and/or at 32 weeks of gestation.
Trial and post-trial surveillance	(i) To determine the safety and efficacy of life-style intervention (yoghurt and PA), given to women at high risk of GDM, on maternal weight gain and blood pressure during pregnancy<br><br>(ii) To determine the safety and efficacy of life-style	serial weight and blood pressure measurements at all scheduled visits<br><br>**Maternal outcomes**<br>• Proportion of women undergoing instrumental/caesarean

For peer review only - http://bmjopen.bmj.com/site/about/guidelines.xhtml

	intervention (yoghurt and PA) on adverse maternal and new-born outcomes.<br><br>(iii) To determine social and cultural barriers to life-style changes during pregnancy	delivery (elective and emergency) captured on delivery records by treating obstetrician<br>• Post-partum haemorrhage (Primary) as captured on delivery records by treating obstetrician<br>• Pre-eclampsia and eclampsia based on blood pressure recording at each visit<br>• Blood loss at delivery as captured on delivery records by treating obstetrician<br><br>**<u>New born outcomes</u>**<br>• Pre-term births (<37 weeks of gestational age) calculated from the ultrasound derived gestational age captured at screening visit dating scan.<br>• Foetal macrosomia (defined as birth weight >2 SDs above the population-specific mean in each setting)<br>• Birth weight and length<br>• APGAR score at 1 and 5min of after birth<br><br>Questionnaire based assessment of social and cultural barriers.

For peer review only

2. ABBREVIATIONS

AC	Abdominal circumference
ACOG	American College of Obstetrics and Gynaecology
AE	Adverse event
AFVI	Amniotic Fluid Volume Index
BMI	Body Mass Index
BPD	Biparietal diameter
BW	Birth weight
CRF	Case Report Form
CRL	Crown-rump length
CTRG	Clinical Trials & Research Governance, University of Oxford, UK
CI	Confidence Interval
CCM	Cultural Consensus Modelling
CFU	Colony forming units
CMC	Christian Medical College, Vellore, India
CTRI	Clinical Trials Registry, India
CUREC	Central University Research Ethics Committee
CVD	Cardiovascular disease
DMC	Data Monitoring Committee
FFQ	Food Frequency Questionnaire
GA	Gestational age
GCP	Good Clinical Practice
GP	General Practitioner
GDM	Gestational Diabetes Mellitus
GWG	Gestational weight gain
HC	Head circumference
IADPSG	International Association of the Diabetes and Pregnancy Study Groups
ICF	Informed Consent Form
IDEA	Impact of Diet and Exercise Activity on Pregnancy Outcomes study
IR	Insulin Resistance
ISRCTN	International Standard Randomised Controlled Trial Number
IUGR	Intrauterine growth restriction
LGA	Large for gestational age

For peer review only

LIMIT	Antenatal lifestyle advices to improve pregnancy outcomes study
LiP	Lifestyle interventions in Pregnancy study
LMICs	Low and Middle Income Countries
LSHTM	London School of Hygiene and Tropical Medicine, UK
MRCG	Medical Research Council, The Gambia at LSHTM
MRC, UK	Medical Research Council, United Kingdom
MS IDREC	Medical Sciences Interdivisional Research Ethics Committee, Oxford, UK
OGTT	Oral Glucose Tolerance Test
PA	Physical Activity
PAMELA	Physical activity during pregnancy and maternal-child health study
PCOD	Polycystic ovarian disease
PI	Principal Investigator
PIS	Participant Information Sheet
PID	Personally identifiable data
PI_x	Pulsatility Index
PPAQ	Pregnancy-related Physical Activity Questionnaire
PRIMORDIAL	Pregnancy Interventions In Mothers Relating to Diabetes In Asian India and Low-income countries
RCT	Randomized Controlled Trial
REC	Research Ethics Committee
RR	Risk ratio
SD	Standard deviation
SOP	Standard Operating Procedure
SAE	Serious Adverse Event
SGA	Small for Gestational Age
S/D ratio	Systolic/diastolic ratio
TOP	Treatment of Obese Pregnant Women study
T2D	Type 2 Diabetes Mellitus
UPBEAT	UK Pregnancies Better Eating and Activity Trial
WHO	World Health Organization

For peer review only

3. BACKGROUND AND RATIONALE

Gestational diabetes mellitus (GDM) is associated with significant intrapartum complications, perinatal morbidity, and long-term risk of developing T2D, obesity and CVD in both the mother and the offspring (1, 2). Normally, hyperglycaemia rapidly abates following delivery. However, about 50-70% of GDM mothers develop T2D within 5-10 years postpartum (3) suggesting that GDM may be a prodrome of "common T2D". The key underlying pathophysiology is the loss of β-cell plasticity to adapt to the progressive insulin resistance (IR). GDM risk can be reduced at two levels: i) by reducing IR secondary to obesity and ii) by improving glucose disposal. Recently, alterations in the gut microbiota have been linked to obesity, IR and T2D (4, 5). The gut microbiota composition changes over the course of gestation (6) and this is further exacerbated on the background of maternal IR and adiposity. Alteration of gut microbial composition through dietary changes and physical activity exert beneficial effects in terms of weight gain, blood glucose levels, reduced IR and T2D risk (7, 8). Thus, simple interventions that aim at altering the gut microbiota by life-style modification [diet and physical activity (PA)] and thus improving glucose disposal constitute potentially attractive options to reduce the risk of GDM.

Global burden of GDM GDM affects 1-14% of all pregnancies worldwide (9) and the risk of GDM reflects the underlying T2D frequency in the community (10). The prevalence is increased to 17.8% (range 9.3–25.5%) with the recent introduction of IADPSG criteria (11). The risk of GDM in specific ethnic groups particularly Asians and Africans is increased compared with Caucasians and thus contributes substantially to future T2D in these communities (12, 13). The incidence of GDM in LMICs is largely unknown and is mostly based on single-centre based statistics. In India, the reported prevalence varies between 17-19% (14). A recent systematic review of GDM prevalence in six African countries (mostly based on 2-step Carpenter Coustan's or WHO criteria), report a prevalence equating to 11% (15). This may not be a true representation of the African continent and there is a relative absence of reliable prevalence estimates of GDM in West Africa.

Window for intervention 'High-risk' GDM mothers include pregnant women with chronic IR states such as overweight or obesity, rapid gestational weight gain (GWG), history of a polycystic ovarian disease (PCOD), impaired glucose tolerance prior to pregnancy, previous GDM and advanced maternal age. Other recognised risk factors include having a first-degree relative with T2D, previous history of stillbirth/miscarriage, a large baby or pre-eclampsia. Intense glycaemic management is usually reserved for mothers diagnosed with GDM, typically late in pregnancy (24-26 weeks), when glucose intolerance is apparent and almost 60% of β-cell plasticity is lost (16) (Figure 1). The "critical window" for any intervention should therefore ideally start earlier than 24-26 weeks when the pancreatic β-cells are still able to compensate with the maternal IR. The RADIEL study showed a 39% risk reduction in GDM incidence among high-risk pregnant women with diet and

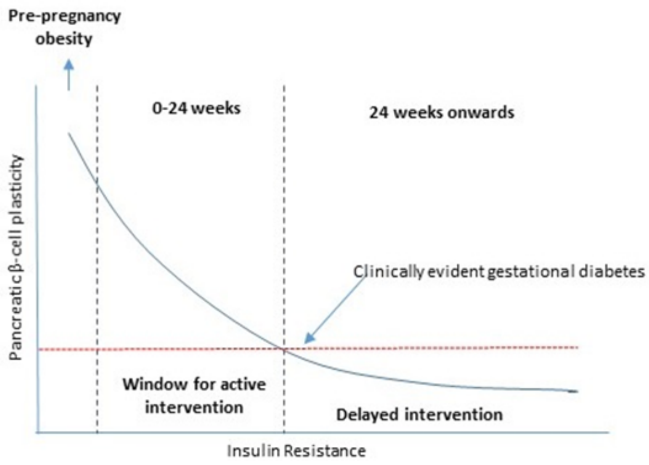

For peer review only - http://bmjopen.bmj.com/site/about/guidelines.xhtml

lifestyle intervention beginning from 20 weeks of gestation (17).

Dietary interventions Fermented dairy products (yoghurt and cultured sour milk) can alter the 'microbiotal-signature' and lower the gut-induced pro-inflammatory response (18). Recent meta-analyses have shown that daily yogurt consumption reduced risk of T2D by 14-18% compared to other dairy products (19, 20). Meta-analysis of probiotic use in late pregnancy has been shown to be safe (41). A summary of studies evaluating the beneficial effects of yoghurt in T2D prevention is shown in page 12.

Physical activity (PA) in pregnancy PA has well-documented health benefits and reduces peripheral insulin resistance in non-pregnant individuals. It is likely that the mechanisms responsible for alterations in insulin sensitivity and glucose uptake in response to exercise in non-pregnant populations may apply to pregnancy. PA is considered safe during pregnancy and substantially reduces the incidence of GDM by reducing IR (21, 22). RCTs and meta-analyses have shown benefits in terms of GWG, delivery type, preterm delivery rate and neonatal outcomes (23, 24). Interventional studies that used aerobic exercises or strength training initiated as early as 9-12 weeks and continued until 38 weeks have clearly shown benefits on glycaemic control, GWG and pregnancy outcomes (25, 26). The ACOG recommend 20-30 minutes of moderate-intensity aerobic activity or strength training at least 3-5 days/week during pregnancy (27). PA has also been shown to alter gut microbiota (28) and a differential microbiota pattern is observed between active vs. sedentary women, implying the role of PA in mediating glucose homeostasis by alteration of gut microbiota.

Hypothesis

Daily yoghurt and/or daily walking for at least 14 weeks will reduce the risk of developing gestational diabetes in 'high-risk' pregnant women

Benefits for the study population and the population whom the results of the project might be generalised to

Benefits to the participants: The current study will sensitise and expose women to specific healthy lifestyles during pregnancy. The opportunity to bring about behavioural changes in the early pregnant woman by changing maternal nutrition and increasing physical activity to prevent GDM can have immediate and lifelong health impact on the mother as well as the new born with possible positive consequences into adulthood. Lifestyle interventions during pregnancy are proven to have beneficial effects on GWG and improved neonatal outcomes. By demonstrating that maternal lifestyle changes beginning from early pregnancy using simple, realistic and culturally acceptable methods will have a beneficial impact in immediate and long term maternal and child health could potentially help reduce the overwhelming global burden of non-communicable diseases, particularly diabetes.

Benefits to the community: Our study aims at providing evidence to support political action and policy making at national and global level in low and middle-income countries (LMICs). This research aims at strengthening the existing antenatal services in The Gambia and the research facilities in India, where such resources are clearly lacking. In line with the research call, the

For peer review only

study will enable opportunities for capacity building in both India and the Gambia. The project will also create opportunities to specifically train young Gambian health providers (obstetricians, midwives, diabetes educators) to travel to India (which is a tertiary medical care facility and well known for its teaching excellence) to be trained in various aspects of antenatal care and high-risk management. In turn, the health researchers in India will clearly benefit from the support of MRC-Gambia in infrastructural set up of a data management facility.

For peer review only

Summary of findings from previous studies

Studies investigating the effect of consuming conventional or probiotic yoghurt during pregnancy

Study reference	Type of study	Target population	Intervention	Findings
Lindsay et al. 2013 (Ref 29)	Systematic review	7 studies including 3 clinical trials using probiotics in pregnancy	-	Impact of probiotic intervention on GDM is better among high-risk mothers
Laitinen et al. 2009 (Ref 30)	Double-blind RCT	Pregnant women <17 weeks (n=256)	Diet + Probiotic<br>Diet + Placebo<br>Control + placebo	Reduced risk of elevated glucose in probiotic group (OR 0.31, 95%CI 0.12-0.18; p=0.013)
Luoto et al. 2010 (Ref 31)	Double-blind RCT	Pregnant women <17 weeks (n=256)	Diet + Probiotic<br>Diet + Placebo<br>Control + placebo	Reduction in GDM risk by 13% in probiotic group
Zheng et al. 2018 (Ref 32)	Meta-analysis	Studies investigating probiotics in pregnancy		Probiotic supplementation during pregnancy is negatively associated with fasting insulin (OR -2.94, 95%CI [-5.69, -0.20], p = 0.04) and HOMA-IR (OR -0.65, 95%CI [-1.18, -0.11], p = 0.02)
Hajifaraji et al. 2018 (Ref 33)	Double-blind RCT	Pregnant women with GDM (n=64)	Probiotic vs placebo for 8 weeks	Improved inflammatory and oxidative stress biomarkers in probiotic group
Nilert et al. 2013 (SPRING Trial) (Ref 34)	Double-blind RCT	Pregnant women <16 weeks with BMI >25kg/m^2 (n=540)	Probiotic vs placebo capsules from 16 weeks to delivery	Ongoing

© Copyright: The University of Oxford 2019

For peer review only - http://bmjopen.bmj.com/site/about/guidelines.xhtml

Studies investigating the effect of PA during pregnancy

Study reference	Type of study	Target population	Intervention	Findings
Russo et al. 2015 (Ref 21)	Meta-analysis	10/469 studies (Total 3,401 GDM/high risk pregnant mothers, control healthy pregnant women)	-	The PA intervention arm showed a 28% risk reduction with GDM incidence compared with standard care arm.
Barakat et al. 2013 (Ref 35)	RCT	510 healthy gravida to either an exercise intervention or a usual care (control) group (n=255 each)	Moderate-intensity resistance and aerobic exercises (three times/week, 50–55 min/session) beginning from 2nd trimester	No reduction in GDM risk between groups. Other outcomes: 58% reduction macrosomia, 34% reducti in elective caesarean delivery and 12% reductio in maternal weight gain.
Barakat et al. 2011 (Ref 36)	RCT	Exercise programme with instructor (three sessions per week, n=40) vs non – exercise group (n=43)	25 min core session + 7-8 min gradual warm up and cool down beginning from 6-9 weeks to 38-39 weeks + one set of aerobic dance every 2 weeks for the intervention arm	Moderate PA during pregnancy improved maternal glucose toleranc
Poston et al. 2015 UK Pregnancies Better Eating and Activity (UPBEAT) trial. (Ref 37)	RCT	1,555 women with a BMI ≥ 30 kg/m2 between 15-18 weeks +	Behavioural intervention (n=772, healthy eating with low glycaemic load foods + 8 sessions with the health trainer on a weekly basis, each lasting 1 to 1.5 hours) vs standard care (n=783)	No difference in risk of GI or incidence of LGA babie

For peer review only

© Copyright: The University of Oxford 2019
For peer review only - http://bmjopen.bmj.com/site/about/guidelines.xhtml

Da Silva et al. 2017 (Ref 38) Physical activity during pregnancy and maternal-child health (PAMELA) Study.	RCT	Healthy pregnant women (n=639)	Exercise intervention thrice weekly for 16 weeks (from 16-20 to 32-36 GA)	No differences between t two groups in mean GWG GDM, BW, infant length, and head circumference.
Haakstad et al. 2011 (Ref 39)	RCT	Nulliparous, sedentary healthy pregnant women (n=105)	12-week supervised exercise programme (60 min supervised aerobic dance = strength training for 60min at least twice weekly)	Mean weight gain during pregnancy was lower in exercise compared to control group (11.0 +2.3 v 13.8 +3.8 kg, p 50.01)
Wang et al. 2017 (Ref 40)	RCT	Singleton pregnant women with BMI between 24 to 28 kg/m2 at 12+6 weeks of GA until 37 weeks	Exercise group vs controls Exercise 3 times per week (at least 30 min/session with a rating of perceived exertion between 12-14) via a cycling program at least 3 times/week	Lower GDM incidence in exercise vs control arm (22% vs 40.6%, p<0.001). Reduced GWG, IR, rates c caesarean delivery, fetal macrosomia and preterm births in exercise group

Studies investigating the effect of diet + PA during pregnancy

Study reference	Type of study	Target population	Intervention	Findings
Vinter et al. 2011 (Ref 41) Lifestyle in Pregnancy (LiP) study.	RCT	360 obese (pre-pregnant BMI 30-45 kg/m^2) pregnant women	Intervention program included dietary guidance + moderate PA for 30-60 min daily + closed training sessions with instructor for 1 h each week	Intervention group had a significantly lower median (range) GWG compared with the contro group of 7.0 (4.7–10.6) vs. 8.6 kg (5.7–11.5; P = 0.01)

For peer review only

Renault et al. 2014 (Ref 42) Treatment of Obese Pregnant Women (TOP) study.	RCT	425 obese pregnant women comparing 3 groups	PA + Diet, (n = 142)<br>PA intervention (daily step count of 11,000 steps/day every day for 4 weeks (n = 142)<br>Control group receiving standard care (n = 141)	PA intervention decreasec GWG by a mean of 1.38 kg (P =0 .040).
Koivusalo et al 2016. (Ref 43)<br>The Finnish Gestational Diabetes Prevention Study (RADIEL study).	RCT	293 pregnant women with a history of GDM and/or a pre-pregnancy BMI of ≥30 kg/m2 at <20 weeks gestation	Intervention group received individualized counselling on diet, physical activity, and weight control. control group received standard antenatal care	GDM incidence of was 13.9% in the intervention group and 21.6% in the control group ([95% CI 0.4 0.98%]; P = 0.044.<br>GWG was significantly lower in the intervention group (p=0.037)
Dodd et al. 2014 (Ref 44, 45)<br>Limiting weight gain in overweight and obese women during pregnancy (LIMIT) trial	RCT	2,152 singleton pregnant mothers between 10-20 weeks GA and BMI ≥25kg/m^2	Diet and lifestyle advice group (n=1,075) vs standard care (n=1067)	Risk of LGA babies were similar in both groups Babies of life-style advice group had higher mid-thig fat mass measured by US and rate of adipose tissue deposition was slow in thi group compared with control group.

© Copyright: The University of Oxford 2019
For peer review only - http://bmjopen.bmj.com/site/about/guidelines.xhtml

Bennet et al. 2018 (Ref 46)	Systematic review and meta-analysis	45 RCTs	-	Diet and PA interventions aimed to reduce GWG wa[illegible] associated with reduced GDM risk by 44% and 38[illegible] respectively. Among Asia[illegible] the risk reduction with die[illegible] and life-style 62% and 32[illegible] respectively
Shepherd et al. 2017 (Ref 47)	Cochrane Database Systematic Review	23 RCTs and cluster-RCTs, comparing combined diet and exercise interventions with no intervention (i.e. standard care), that reported on GDM diagnosis as an outcome	-	Reduced GDM risk in intervention group compared with the standard care group (average risk ratio (RR) 0.[illegible] 95% CI 0.71 to 1.01. Reduced risk of caesarean section (RR 0.95, 95% CI 0.88 to 1.02) in interventi[illegible] arm
Petrella et al. 2014 (Ref 48)	Case-control study	Singleton pregnant women with BMI >25 kg/m^2	Therapeutic lifestyle changes (diet 1800kcal/day + mild PA 30min/d thrice weekly) (n=33) vs Controls (n=28)	Lower GWG, lower incidence of GDM, hypertensive disorders an[illegible] preterm births in the intervention group compared to controls.

© Copyright: The University of Oxford 2019
For peer review only - http://bmjopen.bmj.com/site/about/guidelines.xhtml

4. OUTCOME MEASURES

**Outcomes**	**Measurements**	**Timepoint(s) of evaluation of this outcome measure (if applicable)**
**Primary objective**		
Incidence of GDM	Fasting plasma glucose<br>Glucose at 1 hour and 2 hours' post 75g OGTT (according to IADPSG criteria) or Fasting hyperglycemia measured by fasting glucose	Between weeks 26-28<br><br>At 32 weeks
**Secondary objective**		
Effect of intervention on fasting blood glucose concentration	Fasting plasma glucose	Between weeks 26-28 and at<br>32 weeks of gestation
**Trial and post-trial surveillance measures**		
Gestational weight gain	Maternal weight	At screening, run-in-phase, randomization, visits 1,2,3 and<br>at delivery (before the birth of the child)
Gestational hypertension	Maternal blood pressure	At screening, run-in-phase, randomization, visits 1,2,3 and<br>at delivery (before the birth of the child)
Proportion of women undergoing Instrumental/caesarean delivery	Instrumental/caesarean delivery (elective and emergency)	At delivery
Post-partum haemorrhage (primary)	Amount of blood loss within 24 hours of child birth	From delivery to 24 hours
Blood loss at delivery	Amount of blood lost during delivery	At delivery
Pre-eclampsia and eclampsia	Clinical diagnosis (hypertension + proteinuria +/- seizures)	Clinical diagnosis after 28 weeks of GA
Pre-term births (<37 weeks of gestational age)	Gestational age calculated from dating scan	At birth of the new born
Foetal macrosomia	Birth weight (defined as birth weight >2 SDs above the population-specific mean in each setting)	At birth of the new born
Birth weight	Birth weight	At birth of the new born
APGAR score at 1 and 5 min of birth	Clinically recorded APGAR score	At birth of the new born

Length of the new born	Length measured from head to the heel	Within 48 hours of birth
Barriers to interventions in pregnancy 1 & 2	Questionnaire based assessment	At screening<br>At 32 weeks

5. STUDY DESIGN

Design: 2 x 2 factorial design, multi-centric, open labelled, randomized controlled trial
Total study duration: 36 months
Number of scheduled visits: 5

(i) **Screening visit (visit -2, ≤16 weeks GA):** Singleton pregnant women willing to participate in the study will be assessed for eligibility based on inclusion/exclusion criteria and eligible participants will be asked to sign an informed consent if willing to participate. Eligible women will undergo an oral glucose tolerance test (OGTT) to rule out impaired glucose intolerance or diabetes at screening and will also undergo dating ultrasound scan. Routine antenatal examination will be done. Barriers to life-style interventions-1 during pregnancy will be assessed using a questionnaire.

(ii) **Run-in visit (visit -1, ≤17 weeks GA)**: Baseline assessments of dairy product consumption will be done using standardized questionnaire . Additionally, objective measurements of baseline PA activity over 7 days will be assessed in all study participants (with the result blinded to participants) using a wearable accelerometer device (Garmin Vivofit 4 fitness band).

(iii) **Randomization (≤18 weeks GA):** Women will be randomized to either (i) diet (consumption of 200g of yoghurt per day), (ii) PA (a minimum of 40% increase above the recorded baseline PA) (iii) diet and PA or (iv) standard care. Active intervention will be from randomization to 32 weeks of GA.

(iv) **Visit 1 (18-20 weeks GA)**: maternal weight, blood pressure, routine antenatal examination and foetal morphology scan.

(v) **Visit 2 (26-28 weeks GA)**: maternal weight, blood pressure, routine antenatal examination and OGTT. Primary endpoint (diagnosis of GDM based on IADPSG critria) will be assessed at this visit. Women with GDM will continue on allocated study intervention until end of study (week 32) in addition to the clinical managemnt provided as per the local obstetric practice.

(vi) **Visit 3 (32 weeks GA, End of Study visit)**: maternal weight, blood pressure, routine antenatal examination, ultrasound foetal scan and fasting blood glucose and barriers to pregnancy intervention-2 assessment. (A repeat ultrasound scan at this stage is based on the evidence that growth restriction although starts in early pregnancy, is more marked between 22-34 weeks and also that foetal measurements do not have

For peer review only

exact equivalents to new born measurements. Therefore, a last trimester scan is essential as it reflects foetal growth more precisely).

(vii) **Delivery visit:** This is a post-trial surveillance visit primarily intended to assess the safety and efficacy of interventions on pregnancy and new-born outcomes.

Aside from the scheduled study visits, participants will be contacted on a weekly basis either over telephone or visited at home by research personnel. This will be made to ensure general well-being, compliance (wearing device, adhering to individually advised increment in step count, reminders of study visits, yoghurt consumption etc) and motivation.

Interventions

Dietary intervention Each participant in the diet and diet+PA arm will be advised to consume yoghurt 200 g/day once daily prepared locally at the dietary department in CMC, Vellore and from a local production in The Gambia. The yoghurt consumption is independent of the timing of their regular meal time (can be consumed anytime). Record of daily yoghurt and background dairy consumption will be maintained by every participant. The yoghurt will be prepared on a common recipe with the same starter culture at both centres. The yoghurt will be dispensed daily/weekly as feasible by the study centre. Adherence to yoghurt consumption will be monitored on a weekly basis by home visiting field staff. Dietary counselling will be reinforced in all participants and the non-diet arm will not be advised to stop their regular dairy consumption, assuming that any effect on outcome is related to the trial yoghurt consumed.

Physical activity intervention Women randomized to the PA and diet+PA arm will be provided PA targets to be followed throughout pregnancy based on their baseline PA assessment which was obtained at run-in-phase. The recommendation will be set based on a minimum of a 40% increase of the baseline step count measured during run-in-phase (refer to section 8.1.2 for details). The daily PA activity will be monitored using an accelerometer device (Garmin Vivofit 4 fitness band) which will provide visual feed back to the participants in the active PA arms in order to motivate participants and help them achieve their daily target step count. All other (yoghurt and standard arm) will also be provided with fitness bands to objectively measure their routine PA behavior throughout the study. However, step counts will be blinded for these participants. Data from these devices will be periodically uploaded using vivohub 2 for compliance monitoring and downloaded to provide visual feedback and counselling to participants.

Potential risks and benefits

Risks with study-related procedures

Risks with venepuncture may include bruising in the area of venipuncture, dizziness, nausea, vasovagal syncope. Consumption of oral glucose solution on an empty stomach may cause nausea, vomiting, headache. Ultrasound scans during pregnancy are not considered harmful. Utmost care would be taken to minimize such risks. A trained phlebotomist or research nurse will draw blood samples for the study. We will use clean, sterile phlebotomy equipment. Blood will be drawn using vacutainers with women sitting or lying down, whichever is comfortable. Local pressure will be applied for 20-30 seconds to avoid post-puncture bleeding.

For peer review only

Risks with interventions

There are no major serious adverse effects directly linked to the proposed interventions in the study. Both yoghurt and PA are generally considered safe during pregnancy. However, some potential adverse effects of the proposed interventions can be anticipated and are not necessarily caused by or related to the intervention. In the yoghurt consumption arm, anticipated risks may include nausea, vomiting and intolerance to yoghurt. In women undertaking physical activity, common adverse effects may include dizziness, headache, exertional dyspnoea, easy fatigability and muscle aches. The proposed PA in our study meet the safety guidelines for physical activity recommended by the ACOG (27). Safety will be ensured by excluding women who have absolute contraindications to aerobic physical activity during screening. Potential risks of PA may include persistent vaginal spotting/bleeding, calf pain/swelling (diagnosed to be related to thrombophlebitis or deep vein thrombosis), dyspnoea at rest, painful premature contractions, decreased foetal movements, preterm labour and preterm/premature rupture of membranes. A participant will be withdrawn from the study, in the event of persistent adverse events reported by the participant, and the PI feels that the event may be related to the intervention.

Data collection process

Data type	Method of collection	
	Gambia	Vellore
Weight (of the mother)	Digital Tanita scale	Micro weight electronics. Model: DS415 series
Height (of the mother)	Leicester height measure, seca 214	Stadiometer
Blood pressure	Electronic BP apparatus (Omron Corporation, Tokyo, Japan)	Electronic BP apparatus (Omron Corporation, Tokyo, Japan)
Baseline diet consumption	Dairy consumption questionnaire	Dairy consumption questionnaire
Barriers assessment	Questionnaire	Questionnaire
PA activity monitoring device	Garmin Vivofit 4 fitness band	Garmin Vivofit 4 fitness band
Height (of the baby)	Infantometer	Infantometer
Ultrasound	Aloka USI-145	Voluson GE S-8
Biochemistry		
Blood glucose	Roche Cobas 800<br>Enzymic (autoanalyser)	Roche Cobas 800<br>Enzymic (autoanalyser)

6. PARTICIPANT IDENTIFICATION AND RECRUITMENT

6.1. Study Participants

The study will include healthy pregnant women who have at least one “risk-factor” for developing gestational diabetes (see section 6.2 for high-risk definition).

For peer review only

6.2. Inclusion Criteria

- Pregnant women, aged ≥18 years
- Pregnant women ≤16 weeks of gestational age
- In addition to the above, pregnant women should meet at least one of the following criteria for high-risk GDM:
  - Booking BMI ≥25kg/m^2
  - Age ≥25 years
  - First-degree relative with diabetes
  - Previous pregnancy with GDM
  - Previous gestation with large baby (≥3.5kg)
  - Previous pregnancy with pre-eclampsia/eclampsia
  - History of PCOD/impaired fasting glucose
- Not currently on any medications (except iron, calcium or folic acid supplements, thyroxine supplement for hypothyroidism, low dose aspirin for pre-eclampsia)*
  *women conceived by ovulation induction using clomiphene citrate or metformin for PCOD will not be excluded from the study.

6.3. Exclusion Criteria

The participant may not enter the study if ANY of the following apply:

- GDM diagnosed prior to screening visit based on IADPSG criteria or documented raised HbA1C, i.e., either fasting glucose ≥5.1 mmol/L or 1h glucose ≥10.0 mmol/L or 2h glucose ≥8.5 mmol/L, or a documented HbA1c of ≥6.5% at first booking
- History of pre-gestational diabetes
- Multiple gestation in current pregnancy
- History of severe hyperemesis in the first trimester
- Uncontrolled pre-gestational or gestational hypertension (BP>150/100mm Hg) or on treatment
- History of recurrent (more than two) first trimester spontaneous abortions or stillbirths
- History of significant ante- or post-partum hemorrhage in the previous pregnancy
- Previous child born with congenital anomalies
- Pregnancy following in-vitro fertilization or any assisted reproductive technology and those unwilling to adhere to the study protocol will be excluded from the study
- Previous or current psychiatric illness on medication
- Previous or current neurological condition (e.g. epilepsy) or on medication
- Women meeting absolute contraindications for physical activity during pregnancy as recommended by the ACOG (heart disease, restrictive lung disease, incompetent cervix/cerclage, pregnancies at risk for premature labour, gestational hypertension, severe anaemia)
- Physical disability to PA and/or known lactose intolerance

7. STUDY PROCEDURES

The schedule of procedures at each visit is provided below

For peer review only

Visit	Study procedures
**Screening visit (≤16 weeks) (Visit -2)**	Height<br>Weight<br>Blood pressure (mother)<br>Antenatal examination (fundal height)<br>OGTT<br>Dating Ultrasound scan<br>Barriers to pregnancy intervention assessment-1
**Run-in-phase (≤17 weeks) (visit -1)**	Weight<br>Blood pressure<br>Antenatal examination (fundal height)<br>Questionnaire based assessment of background dairy consumption<br>Diet and PA counselling<br>Dispensing wearable activity monitors device for assessment of baseline PA for 7-days and compliance testing.
**Randomization (≤18 weeks) (visit 0)**	Weight<br>Blood pressure<br>Routine antenatal examination (fundal height)<br>Randomization
**Visit 1 (week 18-20)**	Weight<br>Blood pressure<br>Routine antenatal examination (fundal height)<br>Morphology scan
**Visit 2 (26-28 weeks)**	Weight<br>Blood pressure<br>Routine antenatal examination (fundal height)<br>OGTT
**Visit 3 (32 weeks) End of Study visit**	Weight<br>Blood pressure<br>Routine antenatal examination (fundal height)<br>Fasting plasma glucose<br>Ultrasound scan (to monitor foetal growth)<br>Barriers to pregnancy interventions assessment-2 (for future studies)
**Delivery (post-trial surveillance)**	Weight (mother)<br>Blood pressure (mother)<br>Intrapartum outcomes<br>Neonatal outcomes (Birth length, Birth weight, APGAR score)
**Telephonic contacts / Home visits**	General well-being<br>Compliance with interventions (step count from accelerometer, yoghurt pot-count)

7.1. Recruitment

In both India and The Gambia, women will be identified through home-visits by field workers and in the out-patient clinics of recruiting hospitals operated through the MRC Unit. In The

For peer review only

Gambia, the investigators will use additional strategies such as (i) community sensitization through village meetings that includes the community leaders, ward heads and their development committees, traditional birth attendants (TBAs) and community health workers (ii) house-to-house campaigns led by field workers and TBAs to encourage newly pregnant women to attend antenatal clinics (iii) Frequent use of the local radio stations for general community sensitisation and (iv) referrals from maternal and child health staff and obstetric radiologists to enhance participant identification and recruitment. In India, CMC, Vellore, recruitment will also be made from referrals from several community hospitals and peripheral health centres such as Community Health and Development Hospital (CHAD), Low-cost Effective Care Unit (LCECU), Rural Unit for Health and Social Affairs (RUSHA) and Community Health Nursing (CONCH) by engaging the rural community in grass root health and social programmes. Participants will be recruited by word of mouth.

7.2. Informed Consent

Written and verbal versions of the Participant Information sheet (PIS) and Informed Consent form (ICF) will be presented to the participants detailing no less than: the exact nature of the study; what it will involve for the participant; the implications and constraints of the protocol; the known side effects and any risks involved in taking part. It will be clearly stated that the participant is free to withdraw from the study at any time for any reason, and with no obligation to give the reason for withdrawal. As a part of consenting women will be informed that withdrawing consent from the study will not prejudice their medical care in any way.
The participant would be allowed as much time as wished to consider the information, and the opportunity to question the personnel administering the ICF (physician, medical research officer/midwife/field worker who are authorised to do so by the PI). The participant would provide his willingness to take part in the study by signing the ICF. In the event that the participant in uneducated, her left thumb impression would be obtained along with the signature of the neutral person who will serve as an impartial witness. A copy of the signed ICF would be given to the participant. The original signed form will be retained at the study site.

7.3. Screening and Eligibility Assessment:

Pregnant women aged ≥18 years and ≤16 weeks of gestation who are willing to participate in the study will be screened and assessed for eligibility.

In addition to above, participants should meet at least one of the below mentioned criteria to be termed as “high-risk” for inclusion into the study

- Booking BMI ≥25kg/m^2
- Age ≥25 years
- First-degree relative with diabetes
- Previous pregnancy with gestational diabetes mellitus
- Previous pregnancy with large baby (≥3.5kg)
- Previous pregnancy with pre-eclampsia/eclampsia
- History of PCOD/impaired fasting glucose

Women will then undergo a dating scan to confirm the GA and an OGTT (as described in section 7. Study procedures). Women with blood glucose values at fasting, 1 hour and 2 hours within

For peer review only

normal limits will move forward to the run-in-phase (visit -1) and others will be treated as “screen failure”

7.4. Randomisation

Eligible women will be randomly allocated to one of the following arms (i) Yoghurt (ii) PA (iii) Yoghurt + PA or to the (iv) control group (neither intervention, standard care) following an allocation schedule prestratified for study centre, age and BMI generated by an independent statistician not involved in the study. A variable block randomisation, stratified by centre, and then by age, (<25 and ≥25 years) and BMI (<25 and ≥25 kg/m2) will be generated, using a password-protected Access database developed by an independent statistician who is not involved with trial participants or data analysis. A unique study ID will be assigned to each participant at screening by the research officer.

In India, the random allocation of each participant to one of the four intervention groups, stratified by age*BMI will be performed using a computer interface (Access database with forms and data separated into a frontend and password backend, respectively).

In Gambia, due to logistical limitations, separate lists will be generated for each strata combination (age x BMI) and randomisation assigned using opaque, sealed envelopes. As this is an open-labelled study, study participants, and investigators will not be blinded to the treatment arm. Laboratory personnel and the trial statistician will be blinded.

7.5. Baseline Assessments

Baseline assessments are divided over first three visits (screening, run-in-visit and randomization)

7.5.1. Baseline assessments at screening visit (Visit: -2; ≤16 weeks)

I. Eligibility check
II. **Height:** Height of the pregnant mother will be measured using a portable or fixed stadiometer.
III. **Weight:** Weight of the pregnant mother will be measured using a calibrated electronic weighing scale to the nearest 0.1kg.
IV. **Blood pressure**: Systolic and diastolic blood pressure will be measured after 5 minutes seated at rest. It will be measured three times using an automated validated device (Omron M3, Tokyo, Japan), with an appropriately sized cuff. Three measurements will be recorded in a seated position by a trained research staff. The average of three values will be taken for the final analysis.
V. **Antenatal examination (fundal height):** Routine antenatal examination will be carried out according to local obstetric practice.
VI. **Dating ultrasound scan:** The dating scan will be done at screening visit in all participants meeting the eligibility criteria. The following variables will be measured and recorded: Type of gestation –single/twins/triplets, gestational sac, crown-rump length, yolk sac, cardiac activity, gestational age by scan, scan expected date of confinement. Women diagnosed with multiple gestation on scan, gestational week corresponding to more than

For peer review only

1 week greater than the scheduled visit date and absent in-utero gestational sac will be screen failed.

VII. **Barriers to pregnancy intervention assessment-1:** This will be a questionnaire based assessment that includes questions relating to assessment of overall perceived barriers to life-style interventions in pregnancy, perceived difficulties to frequency of study visits, perceived willingness to interventions and study-related procedures, 9-point willingness to participate score, willingness and unwillingness to participate.

VIII. **OGTT:** A fasting glucose venous blood glucose sample taken will be following a minimum 8-hour fast. Participants will be then asked to drink a calibrated dose (75 grams) of anhydrous glucose solution within 10 minutes as soon as possible. Subsequent blood samples will be collected at 60 and 120 minutes following the drink. Based on IADPSG criteria, a diagnosis of GDM will be made if one or more values equals or exceeds the following:

	Values in mmol/l	Values in mg/dl
Fasting glucose	≥5.1 mmol/l	92 mg/dl
1-hour glucose	≥10.0 mmol/l	180 mg/dl
2-hour glucose	≥8.5 mmol/l	153 mg/dl

Women qualifying the criteria for diabetes (based on IADPSG cut-offs) at this stage will be screen failed.

7.5.2. Baseline assessment at Run-in visit (Visit: -1; week ≤17)

I. **Weight:** Weight of the mother will be recorded as described in 7.5.1 (II)

II. **Blood pressure:** Systolic and diastolic BP of the mother will be recorded as described in 7.5.1 (III)

III. **Antenatal examination (fundal height):** Routine antenatal examination including fundal height will be recorded as described in 7.5.1 (IV)

IV. **Dairy consumption assessment:** Background dairy product consumption of the study participant will be assessed using standardized questionnaires.

V. **Safety assessments** to interventions (e.g. adverse events and/or SAE assessment)

VI. **Concomitant medication** assessment

VII. **Baseline PA monitoring:** All participants will be advised to wear an accelerometer device to objectively measure baseline PA for next 7 days. The step counts will be blinded (not made visible on the device) in order to avoid influences on routine activity.

7.5.3. Baseline assessment at Randomization (Visit: 0; week ≤18)

I. **Weight:** Weight of the mother will be recorded as described in 7.5.1 (II)

II. **Blood pressure:** Systolic and diastolic BP of the mother will be recorded as described in 7.5.1 (III)

For peer review only

III. **Antenatal examination (fundal height):** Routine antenatal examination including fundal height will be recorded as described in 7.5.1 (IV)
IV. **Intervention allocation**
V. **Compliance assessment:** to interventions (step count, yoghurt diary)
VI. **Safety assessments** to interventions (e.g. adverse events and/or SAE assessment)
VII. **Concomitant medication** assessment.

7.6. Subsequent Visits

7.6.1. Visit 1 (week 18-20)

I. **Weight:** Weight of the mother will be recorded as described in 7.5.1 (II)
II. **Blood pressure:** Systolic and diastolic BP of the mother will be recorded as described in 7.5.1 (III)
III. **Antenatal examination (fundal height):** Routine antenatal examination including fundal height will be recorded as described in 7.5.1 (IV)
IV. **Safety assessments** to interventions (e.g. adverse events and/or SAE assessment).
V. **Concomitant medication** assessment
VI. **Compliance assessment:** to interventions (step count, yoghurt diary)
VII. **Morphology scan:** The following variables would be measured and recorded: Gestational age, bi-parietal diameter, head circumference, femoral length, Abdominal circumference, estimated foetal weight, liquor amount, placenta: anterior/posterior and upper segment/lower segment, amniotic Fluid Volume Index, Uterine artery Doppler: Pulsatility Index, Systolic/diastolic ratio, gross anomalies in region of head and neck (transcerebellar plane, transventricular plane, biparietal plane, nose/lips), thorax (lungs, 4 chamber view, outflow tract, diaphragm), Spine, Abdomen (stomach bubble, kidneys, cord insertion, number of vessels in cord, genitalia, limbs). Participants with any foetal congenital anomaly, placental malformation or malposition or abnormal ultrasound features that can compromise pregnancy outcome according to the investigator will be withdrawn from the study at this stage.

7.6.2. Visit 2 (week 26-28)

I. **Weight:** Weight of the mother will be recorded as described in 7.5.1 (II)
II. **Blood pressure:** Systolic and diastolic BP of the mother will be recorded as described in 7.5.1 (III)
III. **Antenatal examination (fundal height):** Routine antenatal examination including fundal height will be recorded as described in 7.5.1 (IV)
IV. **OGTT** (as described in in 7.5.1 (VIII))
V. **Safety assessments** to interventions (e.g. adverse events and/or SAE assessment) and review of concomitant medications.
VI. **Compliance assessment:** to interventions (step count, yoghurt diary)

For peer review only

7.6.3. Visit 3 (Week 32) - END OF STUDY VISIT

I. **Weight:** Weight of the mother will be recorded as described in 7.5.1 (II)
II. **Blood pressure:** Systolic and diastolic BP of the mother will be recorded as described in 7.5.1 (III)
III. **Antenatal examination (fundal height):** Routine antenatal examination including fundal height will be recorded as described in 7.5.1 (IV)
IV. **Fasting plasma glucose**
V. **Barriers** to pregnancy intervention-2 assessment
VI. **Ultrasound scan:** The ultrasound scan would be done during visit 3 (week 32) in all participants which would include measurement of bi-parietal diameter (BPD), head circumference, femoral length, abdominal circumference, estimated foetal weight, liquor amount, placenta: anterior/posterior and upper segment/lower segment, amniotic Fluid Volume Index, Uterine artery Doppler: Pulsatility Index, Systolic/diastolic ratio.
VII. **Safety assessments** to interventions (e.g. adverse events and/or SAE assessment) and review of concomitant medications.
VIII. **Compliance assessment:** to interventions (step count, yoghurt diary) & close of interventions.

In the event the participant withdraws from the study at any time prior to week 32 visit, then the date of voluntary withdrawal will be marked as "END OF STUDY VISIT". The participant will undergo all assessments as listed in 7.6.3 except morphology scan.

7.6.4. Post-intervention surveillance visit

All study-related intervention will be stopped at 32 weeks and mother and new born data will be collected during delivery as a part of post- trial surveillance visit. Women who consent will have weekly telephonic contacts/home visits by field staff to ensure general wellbeing. All women will be delivered in the hospital and inter-partum and neonatal measurements will be recorded as below

I. **Weight of the mother** before delivery of the baby
II. **Intrapartum maternal outcome assessment**
III. **Birth length (new born)** The measurement from the crown to the soles of the feet in centimetre scale would be recorded as birth length by an experienced mid-wife.
IV. **Birth weight (new born)** Weight at birth would be measured within 48 hours of delivery using a calibrated scale for measuring neonatal birth weight by an experienced mid-wife.
V. **APGAR score (new born)** The APGAR Score should be used as the accepted and convenient method for reporting the status of the new born immediately after birth and the response to resuscitation if needed. Local neonatal practices should be followed for APGAR score reporting in cases of maternal sedation or anaesthesia, congenital malformations and birth trauma. The APGAR assessment would be made as below by a trained medical neonatologist/paediatrician or midwife at 1 and 5 minutes.

For peer review only

Sign	0	1	2
Colour	Blue or Pale	Acrocyanotic	Pink
Heart rate	Absent	<100/minute	>100/minute
Reflex irritability	No response	Grimace	Cry or Active withdrawal
Muscle tone	Limp	Some flexion	Active motion
Respiration	Absent	Weak Cry	Good, Crying

All women recruited in the study will be regularly contacted over telephone or by home visit to monitor general well-being, diet and PA adherence and early detection of serious adverse events and safety to interventions. The additional weekly phone review is also expected to assist with adherence and retention.

7.7. Sample Handling

Blood sample

Following a minimum of 8 hour overnight fast, women will provide a fasting sample followed by blood samples at 1 hour and 2 hours of consuming oral glucose solution (see Section 7. Oral Glucose Tolerance Test). 2ml venous blood will be collected for each measurement in an EDTA fluoride vacutainer tube (Grey Capped) and placed on ice until centrifuge. Samples will be centrifuged (2,750 rpm for 10 minutes), aliquoted and transported to the analysis lab on ice. OGTT glucose samples and fasting venous glucose during visit 3 will be analysed on Roche Cobas 800 Autoanalyser either on the same day or will be stored in freezer if analysed on the subsequent date. A total of 12 ml blood will be collected during both OGTTs. General timelines for sample handling will be followed (venepuncture to ice no more than 10 minutes, ice to centrifuge no longer than 30 minutes, centrifuge to ice no more than 10 minutes and ice to freezer no more than 6 hours). No samples will be stored for future use. The current study does not involve any genetic or molecular analysis.

7.8. Discontinuation/Withdrawal

Discontinuation/withdrawal has to be distinguished from i) discontinuation from the study (interventions, follow-up visits, study-related procedures) and ii) discontinuation from interventions only and could occur under following circumstances

7.8.1. Discontinuation/Withdrawal from the study

Discontinuation from the study occurs

- When a participant voluntary withdraws consent to further participation at any time during the trial.

For peer review only

- Significant protocol deviation such as Ineligibility (either arising during the study or retrospectively having been overlooked at screening)
- Loss to follow-up

Details of withdrawing/discontinuation from the study prior to completion of all scheduled visits should entered in the "study termination form"

Participants who are diagnosed to have GDM (by laboratory evidence within the protocol or outside diagnosis confirmed by an OGTT at study centre) should continue interventions till week 32 (end of study visit), unless there is a safety concern.

Temporary or permanent discontinuation of interventions (as detailed in sections 7.8.1 and 7.8.2) for safety reasons will not lead to withdrawal of the participant from the trial. Withdrawal of the participants will not be replaced by recruitment of new participants. The primary reason for withdrawal will be specified in the Case Report Form.

7.8.2. Discontinuation/Withdrawal from interventions

7.8.2.1. Temporary discontinuation/withdrawal from interventions

Temporary discontinuation from interventions may occur when a participant does not tolerate the intervention or due to a ***suspected AE*** by the investigator. Best efforts should be made to resume the intervention as early as possible.

7.8.2.2. Permanent discontinuation/withdrawal from interventions

Permanent discontinuation from intervention is any intervention discontinuation associated with the definitive decision from the Investigator not to re-expose the participant to the either yoghurt or PA or both at any time during the study. This may occur during situations of ***unexpected SAE***, where, in the investigator's opinion, continuation with intervention would be detrimental to the participant's well-being

Examples of permanent discontinuation could be

- development of blood pressure 150/100 mmHg anytime during the trial
- Hospitalisation due to medical event (only if further continuation of interventions will affect maternal or new born health)
- Anaemia diagnosed based on haemoglobin <10g/dl
- Ultrasound evidence of major degree placenta previa

Participants will be followed-up according to the study procedures specified in this protocol up to the scheduled date of study completion. It is important to collect data on all participants, regardless of intervention, during the entire duration of the study as all data until the scheduled date of study completion will be used in statistical analyses.

In case the participant decides to withdraw participation or consent during the study, we will not work on participant's samples without permission, but any information already generated from the samples until the time of withdrawal will be used and samples already collected, for which they have given consent, will also be analysed and data used.

For peer review only

7.9. Lost to follow-up

A subject will be considered potentially lost to follow-up if she completed all protocol specific procedures up to the administration of the intervention, but was then lost during the follow-up period, with no safety information and no efficacy endpoint data ever became available. This could occur in situations where a participant repeatedly fails to return for scheduled visits and is unable to be contacted by the trial site.

- Before a subject is deemed lost to follow-up, the investigator should make every effort to regain contact with the subject.
- A subject will not be declared lost to follow-up before all the attempts have been repeated and the trial has come to an end.

The attempts must be documented in the CRF.

7.10. Protocol deviation

A protocol deviation is any noncompliance with the clinical trial protocol, good clinical practice (GCP), or other applicable regulatory requirements. The noncompliance may be either on the part of the participant or the investigator including the study team members, and may result in significant added risk to the study participant.

Deviations from the protocol should be avoided. If deviations do occur, they must be documented and explained in a protocol deviation CRF by stating the reason, date and the action(s) taken. As a result of a deviation, corrective action should be developed and implemented promptly. The investigator must inform the trial monitor and the implications of the deviation must be reviewed and discussed. All protocol deviations must be notified to the regulatory authorities according to local requirements

Deviation from the protocol can occur at following circumstances

- When the investigators miss to schedule a visit
- If an ineligible subject is randomised in error/randomisation stratification error

7.11. Definition of End of Study

End of study will be the last scheduled visit as per protocol i.e., visit 3 (week 32). However, if the participant chooses to discontinue form the study due to any reasons as stated above in 7.8, the "study termination form" should be completed.

8. INTERVENTIONS

The study will be a 2 x 2 factorial design and eligible women will be randomized to either one of the arms – (i) Yoghurt (ii) Physical activity (PA) (iii) Yoghurt + PA (iv) Standard care

8.1.1. Yoghurt intervention

Women randomized to 'Yoghurt' and 'Yoghurt + PA' arms will consume 1 pot (200g) of yoghurt daily from the day of randomization visit to 32 weeks of gestation (more details provided under section 5 Study design – interventions).

8.1.2. PA intervention

For peer review only

Women randomized to 'PA' and 'Yoghurt + PA' arms will follow PA intervention (daily walking and weekly group activity sessions) from randomization to Visit 3 (week 32).

8.1.2.1. Daily walking

"Absolute target" will be calculated as a minimum of a 40% increase from the baseline. This will be calculated as the average of the weekly step count at run-in-phase. Step counts will be monitored using Garmin vivofit 4 band from randomization. Participants will be advised to use the "absolute target" as the minimum reference step count, but try to increase the step count beyond this. Women will be encouraged to perform walking at as speed faster than usual but within the individual's comfort.

Compliance will be calculated based on average step count achieved over a 7-day period including two weekend days. Women achieving at least 80% of the absolute target on a 7-day average will be considered as compliant. Non-compliant participants will be continually motivated and encouraged to achieve their absolute targets either telephonically or by home visits by field workers.

**Baseline a**	**40% increase b**	**Minimum absolute target (a+b)**	**80% of absolute target (d=a+b) increase (expected average 1 week step count to be achieved) Used to calculate compliance**
2500	1000	3500	3300
3000	1200	4200	3960
3500	1400	4900	4620
4000	1600	5600	5280
4500	1800	6300	5940
5000	2000	7000	6600
5500	2200	7700	7260
6000	2400	8400	7920
6500	2600	9100	8580
7000	2800	9800	9240
7500	3000	10500	9900
8000	3200	11200	10560
8500	3400	11900	11220
9000	3600	12600	11880

For example, if baseline activity is 6,000 steps, then an increase of 40% would be an increase of 2,400 steps a day and absolute target to be achieved/day would be a minimum of 8,400 steps.

For peer review only

9. SAFETY REPORTING

Safety evaluations will be performed on all randomized participants at each follow-up visit (Visit 1-3). All AE and SAE, including expected SAEs will be captured in the AE report form. The participants will be instructed to contact the study team if there are any concerns regarding the interventions.

9.1. Adverse event reporting

Any adverse event reported by the participant will be captured in the 'adverse event report' form. Information on AE will be collected by the treating physician at each scheduled visit and by the field worker during home visits. Besides scheduled study visits, AEs will be collected through weekly phone reviews. Any participant reporting an AE will be seen by the research clinician at the earliest appointment (depending on the severity). Details of all events including onset date, causality to the study intervention will be reported, treatment given, stop date will be captured in the respective AE report form.

9.2. Definition of Serious Adverse Events

An untoward medical event leading to /resulting in any of the following will be considered as "serious adverse event"

- Death
- is life-threatening
- requires inpatient hospitalisation or prolongation of existing hospitalisation
- results in persistent or significant disability/incapacity
- loss of foetal movements or ultrasonographically confirmed foetal death
- 'Important medical events' if they jeopardise the participant or require an intervention to prevent one of the above consequences.

9.2.1. Expected SAE

Expected SAEs include potential medical events (as listed below) will be considered as they are likely to be related to the interventions. These events are therefore **protocol-defined exceptions to SAE reporting**. These events should be captured in the SAE form, but does not require reporting to DMC within scheduled timelines. However, local ethics committees should be informed of such events if the principal investigator believes the event is related to PRIMORDIAL interventions.

- persistent vaginal spotting/bleeding after randomisation
- calf pain/swelling (diagnosed to be related to thrombophlebitis or deep vein thrombosis)
- Dyspnoea at rest
- painful premature contractions
- decreased foetal movements
- preterm labour
- preterm/premature rupture of membranes
- Diagnosis of placenta praevia after 26 weeks of pregnancy
- Pre-eclampsia / Eclampsia

For peer review only

9.3. Reporting Procedures for Serious Adverse Events

All research staff will undergo formal GCP training as well as on reporting procedures for AEs and SAEs.

The PI shall report all SAEs without filtration, whether or not related to the intervention, within 24 hours of becoming aware of the event to the Sponsor. If the SAE is related to the intervention, the respective Ethics Committee should be notified within seven calendar days if fatal or life-threatening, and all others within 15 calendar days.

The minimum information required for this initial SAE report is:

- Trial number and (short) title
- Participant's ID
- Date and time of onset
- Description of the event (clinical history, associated signs and symptoms)
- Intervention administered
- Reporter's name

The PI/study coordinator will not wait for additional information to fully document the event before notifying. The report is then to be followed by submission of a completed SAE Report Form as soon as possible, detailing relevant aspects of the SAE in question. All actions taken by the PI and the outcome of the event must also be reported immediately. A decision whether to continue/discontinue the intervention (yoghurt+/- PA) will be made by the treating obstetrician depending on the causality of the event.

For documentation of the SAE, any actions taken, outcome and follow-up, a SAE Report Forms will be used. All follow-up activities have to be reported, if necessary on one or more consecutive SAE report forms in a timely manner. All fields with additional or changed information must be completed and the report form should be forwarded to the respective Ethics Committee within seven calendar days after receipt of the new information. Hospital case records and autopsy reports, including verbal autopsy, will be obtained where applicable.

All SAEs would be reported to the Data Monitoring Committee (DMC) and Oxford Tropical Research Ethics Committee (OxTREC) where in the opinion of the PI the event was 'related' (resulted from administration of any of the research procedures) and 'unexpected' (the type of event is not listed in the protocol as an expected SAE). Reports of related and unexpected SAEs should be submitted within 15 days of the PI becoming aware of the event to DMC and OxTREC.

10. STATISTICS AND ANALYSIS

10.1. Description of Statistical Methods

We will analyse the study using both (1) the intention-to-treat principle and (2) per protocol, including only those participants adhering to at least 75% of the proposed intervention. In addition, we will run a sensitivity analysis to assess the effect of protocol deviation compared to 100% adherence. Analyses will be performed blinded to the intervention group allocation.

For peer review only - http://bmjopen.bmj.com/site/about/guidelines.xhtml

Descriptive analysis of the first 40 participants who complete visit 2 (26-28 weeks) will be performed as advised by the funders (MRC-UK). The analysis is intended to assess feasibility, retention and adherence to all study-related procedures.

10.2. The Number of Participants

The sample size is 1856 high-risk women (928 to be recruited in The Gambia and India). The study was powered to have 80% to detect a 33% reduction in the incidence of GDM, for the main effects of diet and PA, with a family-wise significance level of 5% for a two-sided test (i.e. each effect was tested at a significance level of 2.5%). It was assumed that participants could be pooled across centres (no interaction between interventions and country at the 5% level). The sample size was inflated to allow for a 15% drop out rate in each of the four strata, with drop outs defined as non-compliant participants (i.e. no risk reduction in their incidence of GDM).

The effect sizes for the sample size calculation were:

Prevalence of GDM in high-risk pregnant women: The largest study of GDM women (The HAPO study, 25,505 pregnant women unselected for GDM risk at 15 centres in nine countries) reported a GDM prevalence rate, based on IADPSG criteria, of 17.8% among the general population. In our 'high-risk' population, we assume that the prevalence will be higher than in the HAPO study. Therefore, we have estimated the prevalence of 25% in our study.

Estimated risk reduction: A recent meta-analysis (Song et al. Obesity reviews 2016) has shown that either diet or PA results in 18% (95% CI 5-30%) risk reduction in GDM in general pregnant population and interventions as early as 15 weeks of gestational age was also associated with reduced GDM risk (Relative risk: 0.80, 95%CI 0.66-0.97). Putting all these together, we estimate the risk reduction would be higher (about 1/3, 33%) in this high-risk population. We modelled the GDM percentages in four equal-sized combinations of control (c) and treatment (t) arms as 25% (c-c), 16.75% (c-t and t-c) and 11.2 in t-t group. Therefore, in simple comparisons of one exposure, the percentages will be 20.9% vs 13.0%.

*Absence of intervention*country interaction:* In order to pool the participants of both countries, we will be assuming that there are no differences between the participants of both countries. This initial test for a non-significant interaction term (at the 5% level) between each intervention and country was also taken into account in the sample size simulation.

The sample size calculation was performed by simulation (in R version 3.5.2) according to the following algorithim:

1. For a given sample size, we generated 8 subgroups, two (one per country) for each intervention combination (control-control, treatment1-control, control-treatment2, treatment1-treatment2).

2. For each subgroup we generated the expected GDM incidence, except for a random 15% (drop-outs), whose expected GDM incidence was considered to be 25% (corresponding to the control group) regardless of their originally allocated subgroup.

For peer review only

3. We first tested if either the interaction term site*PA or site*Yoghurt was statistically significant (at the 5% level).

4. If the assumption of no intervention*country interaction was met, we recorded for each intervention if it was statistically significant (at the 2.5%, as we were doing two tests, one for each intervention), assuming a two-sided test

5. This was repeated 10,000 times.

6. The % of repetitions with statistically significant intervention effects represents the power associated with the sample size.

7. Steps 1-6 were repeated for increasing sample sizes.

8. A smoothed (lowess) line was fitted.

9. The point where this line crossed the 80% power mark gave us the final sample size required. (Figure 1)

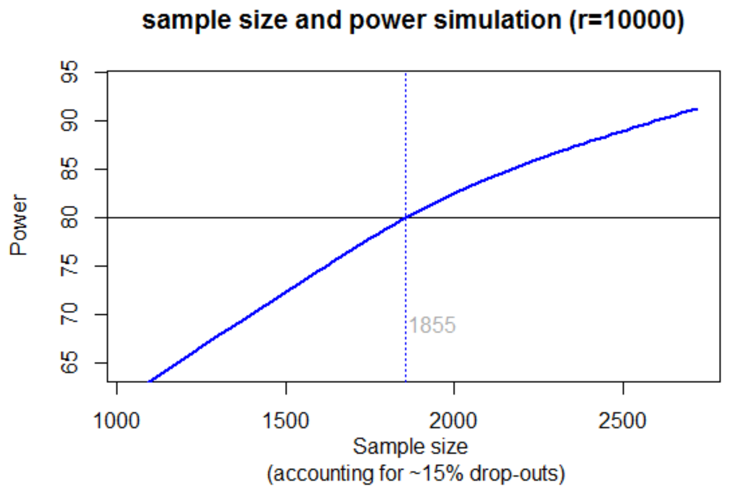

Figure 1: Estimated power for increasing sample size, with an estimated risk reduction of 33%, alpha = 0.05 and estimated drop-out rate of 15% (based on 10,000 iterations).

10.3. Analysis of Outcome Measures

All outcomes will be presented using descriptive statistics; normally distributed data by the mean and standard deviation and skewed distributions by the median and interquartile range. Binary variables will be presented using counts and percentages. We will summarise descriptive data using CONSORT reporting standards for RCTs. STATA will be used for statistical analysis.

10.3.1. Primary outcome

Gestational diabetes incidence will be analysed as risk ratios and risk differences will be estimated by binary regression and results presented as odds ratio and 95% confidence interval.

A covariate adjusted analysis of the effect of treatment on the primary outcome will be undertaken to remove bias from the estimate of the treatment effect on the primary outcome.

We will also test for the difference in effects on GDM incidence between the centres. If the odds ratio (OR) for GDM associated with the intervention in The Gambia is ORg, and in India is ORi, then the ratio of these two terms measures the difference in effect.

10.3.2. Secondary outcomes

The secondary outcomes will compare the difference in fasting plasma glucose at week 32, gestational weight gain, intrapartum and neonatal outcomes between the intervention and control groups. Continuous variables will be analysed using multiple linear regression and binary variables by logistic regression and adjusted for pre-specified covariates.

10.3.3. Safety

The incidence rates of AEs and SAEs and their relationship to trial interventions will be summarised by intervention group. The proportion of women discontinuing treatment will be summarised by reason and by intervention group.

11. DATA MANAGEMENT

All protocol required data will be captured in electronic Case Report forms (eCRF) that will be completed for each included participant by trial personnel. Data will be regularly checked by the Data Manager. On the eCRF, a reference to the source document will be provided. Instructions for completing all forms, including the CRF, used in the study will be developed. All data will be entered onto a secured, password-protected database that will only be accessed by the data manager and designated investigators.

11.1. Types of data

The study will include collection of both research (quantitative and qualitative) and personal data. Data minimisation has been considered and thus personal data will only include name, addresses and telephone number collected for the purpose of telephonic follow-ups/reminders and home visits by the field staff for the current study. These data will however be also used if the participant needs to be contacted for future approved studies (follow-up of babies born to high-risk GDM mothers). Personal information will be available only to the study PI and designated staff involved in contacting the participants either telephonically or by home visits. As this study has the potential to be extended in the future for other follow-up studies, personal data will pseudonymised and stored locally at their study centres. There will be no transfer/sharing of personal data outside the study centre. This personal data will be not be linked to research data and will be maintained in separate files at each study centre and will be password protected and accessible only to authorized staff of the study team.

11.2. Format and scale of data

REDCap software will be used in the trial. It is a secure web application for building and managing databases. The final data format will be downloaded to Excel and common statistical packages (SPSS, SAS, Stata, R). REDCap is specifically geared to support online or offline data

For peer review only

capture for research studies and operations. This data management system is a fully supported, externally validated (GCP compliant to 21 CFR parts 11) clinical database. The database will be hosted and managed by the MRCG and the final tested production database will be shared with the partner site in India who will enter/synchronize data into the central database.

11.3. Data collection/generation

Research data will be collected using electronic CRFs (eCRFs). Data collection will be undertaken by designated study staff involved in the study. Only those individuals who have been trained on the CRF/eCRFs and/or other document completion and have been authorized by the Site PI may complete eCRFs and other study specific documentation. The research data collected on the eCRFs will be captured using devices i.e. tablets and/or laptops used by study staff. Where there's internet connectivity, collected data will be stored directly onto the central server and where there is no/unreliable connectivity, data will be stored locally on the tablet and later synchronized to the central servers at MRCG Fajara. Field staff will be trained to use of the REDCap mobile application and clinicians will be trained to use the REDCap web portal. The data from the accelerometer used for monitoring PA will be directly downloaded on the tablet by the trained research staff on a weekly basis.

11.4. Data quality and standards

Review of data collection tools against the protocol will occur prior to database build by the Data Developer to ensure all data is being captured, at the correct time points, and in a format conducive to the proposed statistical analysis. Site based staff will be fully trained in conducting any study assessments and subsequent completion of the CRF and a Site delegation log will be maintained. Study staff will be trained in the use of Redcap. Principles of GCP will be adhered to and all training will be documented. To ensure standardisation of processes, standard operating procedures with respect to trial management, quality assurance, data management, IT & security, and statistics will be adhered to. The entry screens will be designed with range checks, skip patterns and validations to assist/guide data collectors and to ensure high level accuracy. Multiple choice options will be employed when and where applicable. Mandatory field checks and other computable data will be pre-populated to eliminate errors. The data manager will conduct periodical data cleaning routines to flag data queries that were not picked up in the earlier stages. All data queries raised by the data manager will be answered by the local PI or a delegated senior member of the study team involved in the generation of the data in question normally within one week. All changes to any CRFs or databases will be governed by a change control SOP, ensuring that all sites are using the same version of both CRFs and databases.

11.5. Managing, storing and curating data

Paper records will be stored securely in locked rooms or cabinets, according to standard filing and archiving procedures by both centres. Electronic data collected at both sites (India and The Gambia) will be centrally monitored and stored by the MRC Unit, The Gambia. The database will be housed on a secure network server with no direct physical access to it by project team members. Access to the system is via a web interface that will be hosted on the Fajara production server (https://redcap.mrc.gm/redcap/redcap) managed by the IT department. All data stored in REDCap will be formatted in XML when exported using Clinical Data. Interchange Standards Consortium Open Data Model (CDISC ODM) export format which is vendor neutral,

For peer review only - http://bmjopen.bmj.com/site/about/guidelines.xhtml

platform independent format for interchange and archive of clinical trials data. The database will be backed up as part of the IT department's backup routine and disaster recovery process. ICF will be maintained locally in the respective study centres in secure cabinets will limited access to authorized study staff only. The ICF will be retained for a period of 5 years after study completion and publication of primary results. Other PID (personally identifiable data) will be stored indefinitely for the purpose of future follow-up studies.

11.6. Data preservation strategy and standards

At the end of the study (when the last participant enrolled in the study completes the delivery visit) the database will be locked and all the electronic study data will be housed in the REDCap Database server until the first publication is made following which the data will be sent to the electronic data archives for long-term preservation. The locked database will have very highly controlled access as detailed in the Archives Research Records Management Policy (POL-ARM-001 - Available on request). All electronic records will be kept indefinitely and all paper records and lab books will be stored in the on-site temperature and pest controlled MRC as dictated by the MRC Unit The Gambia's SOPs on Data Retention and Destruction (SOP-ARM-005) and formal information/data security standards The study will be run in compliance with the MRC Corporate Information security Policy and the MRC Unit The Gambia's INFORMATION AND COMMUNICATION TECHNOLOGY SECURITY POL-INT-001 available on the MRC Gambia intranet (Available on request).

11.7. Main risks to data security

Main risk to confidentiality is the personal information on subjects kept by the local-PI for study follow up verification purposes. These will be kept separate from the actual research data and not available to the study staff so that no linkages of personal data to study records would be easily made. The research data will be stored in a limited access, password protected database so that only staff who have the required permissions can view the study records. Personal information will be available only to study PI and designated staff involved in contacting the participants either telephonically or by home visits.

11.8. Suitability for sharing

The data generated within the study will be suitable for sharing in an anonymised format. The data will be in a format that is suitable to share with other interested researchers. All data meant for sharing will be anonymised/de-identified by removing all individual-level participant data (IPD) and will be archived on the servers of the Archives department of MRCG. Data sharing will be in agreement with the MRCG as outlined and governed by the Data Sharing Policies i.e. Data Sharing Policy v2.2 and MRC Policy and Guidance on Sharing of Research Data from Population & Patient Studies. The regulations will also adhere to the University of Oxford data policy guidelines. PID will not be shared across organisations.

11.9. Access to Data

Direct access will be granted to authorised representatives from the University of Oxford and any host institution for monitoring and/or audit of the study to ensure compliance with regulations.

For peer review only

11.10. Data Handling and Record Keeping

All study data will be downloaded from RedCap into an Excel spreadsheet. The participants will be identified by a unique study specific number in any database. The name, addresses and any other identifying details will NOT be included in any study data electronic file. All data will be fully described in the Excel data dictionary which will include the variable name, variable description, label (if different from description), database table name, data grouping information, type of data (sting, integer, real etc.), data codes and decodes and any validation rules used. All data stored in REDCap will be formatted. Person data and research data will not be linked. After completion of the study, research data will be stored indefinitely at central servers in MRC, Fajara, The Gambia. PID including ICF will be stored for a minimal period of 3 years after study completion and destroyed subsequently as per local data management policies. All data management processes and procedures will be governed by the library of MRCG's data management standard operating procedures.

12. COMMITTEES STRUCTURES

The study will be conducted in accordance with the current approved protocol, Good Clinical Practice (GCP) regulations and standard operating procedures (SOPs).

12.1. DATA SAFETY MONITORING COMMITTEE (DSMC)

An independent Data Safety Monitoring Committee (DSMC) has been set up to review the progress of the trial and provide feedback to the Trial Steering Committee (TSC).

The DMC will be responsible for reviewing

(i) general study progress (recruitment, compliance, loss to follow-up)
(ii) clinical study safety data and assess and/or adjudicate all events of deaths
(iii) the GDM event rate and key secondary/safety outcomes
(iv) results of the interim analysis. The DSMC includes the following members (To address: chair of DSMC, Report preparation, confirm meeting timelines).

The DMC will review and analyze, on a regular basis, unblinded safety data throughout the study.

An independent DSMC with members who are independent from the Sponsor and the investigators for the PRIMORDIAL study comprises of experts in clinical trials/epidemiology, biostatistics and obstetrics and include the following members

Name (Designation)	Address	Email
Dr. Udak Okoma (Paediatrician & Postdoctoral Research Fellow)	MRC Unit The Gambia at the London School of Hygiene & Tropical Medicine, Fajara, Banjul, The Gambia	uokomo@mrc.gm
Dr. Matthew Johnson (Biostatistician)	MRC-LEU, University of Southampton, UK	mj2@mrc.soton.ac.uk

For peer review only

Dr. Lucy McKillop (Obstetrician)	Nuffield Department of Obstetrics and Gynaecology, Oxford, UK.	lucy.mackillop@ouh.nhs.uk

Meetings will be held by skype prior to start of the recruitment and then 6-monthly thereafter. The study protocol will be approved by the DSMC prior to enrolment. Any subsequent updates and implications will be reviewed periodically.

12.2. Trial Steering committee (TSC)

The TSC is responsible for supporting the Sponsor in designing a scientifically sound study. In this capacity, the members of the TSC shall address and resolve scientific issues encountered during the study. The TSC would take decisions on protocol amendments (along with DSMC, if required) and communicate with the research teams. All changes would be implemented following ethics approval from all participating bodies.

Name	Address	Email / Phone
Prof Lisa K Micklesfield (Independent chair)	University of Witwatersrand, Johannesburg, South Africa	Lisa.Micklesfield@wits.ac.za
Dr. G. Krishnaveni (Independent member)	CSI Holdsworth Memorial Hospital, Mysore, India	gv.krishnaveni@gmail.com
Prof Louise Bowman (Independent member)	CTSU, Nuffield Department of Population Health, Oxford	louise.bowman@ndph.ox.ac.uk
Prof Fredrik Karpe (Principal investigator)	OCDEM, Radcliffe department of Medicine, Oxford	fredrik.karpe@ocdem.ox.ac.uk
Prof Caroline Fall (Co-investigator)	MRC-LEU, University of Southampton, Southampton, UK	chdf@mrc.soton.ac.uk
Dr. Senthil Vasan (Trial Manager, Non-voting members)	OCDEM, Radcliffe department of Medicine, Oxford	senthil.vasan@ocdem.ox.ac.uk
Dr. Melissa Lennartz-Walker (Newton Fund Program Manager, MRC)	MRC	Melissa.Lennartz-Walker2@mrc.ukri.org

For peer review only

12.3. Executive committee

The executive committee includes active investigators who will be directly involved in the day-to-day running of the trial

Name	Address	Email / Phone
Prof Fredrik Karpe (Principal investigator)	OCDEM, Radcliffe department of Medicine, Oxford	fredrik.karpe@ocdem.ox.ac.uk
Dr. Senthil Vasan	OCDEM, Radcliffe department of Medicine, Oxford	senthil.vasan@ocdem.ox.ac.uk
Prof Andrew Prentice	MRC Unit The Gambia at LSHTM, Atlantic Boulevard, Fajara, P.O. Box 273, Banjul, The Gambia	Andrew.Prentice@lshtm.ac.uk
Dr. Modou Jobe	MRC Unit The Gambia at LSHTM, Atlantic Boulevard, Fajara, P.O. Box 273, Banjul, The Gambia	mojobe@mrc.gm
Dr. Mustapha Bittaye	MRC Unit The Gambia at LSHTM, Atlantic Boulevard, Fajara, P.O. Box 273, Banjul, The Gambia	mubittaye@mrc.gm
Prof Jiji Matthews	Department of Obstetrics and Gynaecology, Christian Medical College & Hospital, Vellore, India	coronistrial@yahoo.co.in
Dr Swathi Rathore	Department of Obstetrics and Gynaecology, Christian Medical College & Hospital, Vellore, India	swatixrathore@gmail.com

13. ETHICAL AND REGULATORY CONSIDERATIONS

13.1. ICH-GCP

The study will be conducted in accordance with the protocol and applicable International Conference on Harmonisation (ICH) GCP guidelines.

For peer review only

13.2. Approvals

The protocol, protocol amendments, ICF and the PIS will be submitted to the OxTREC, and host institution (MRCG and Ethics committee, CMC, Vellore) for written approval. Additionally, approvals will also be sought from Indian Council of Medical Research (ICMR) and the Medical control agency, The Gambia. The Investigator will submit and, where necessary, obtain approval from the above parties for all substantial amendments to the original approved documents.

13.3. Participant Confidentiality

The study staff will ensure that the participants' anonymity is maintained. The participants will be identified only by a participant ID number on all study documents and any electronic database. All documents will be stored securely and only accessible by study staff and authorised personnel. The study will comply with the Data Protection Act, which requires data to be anonymised as soon as it is practical to do so. Personal data will be not be linked to research data and maintained in separate files which are password protected and accessible only to authorized staff of the study team. More details are provided under Section 11.

13.4. Expenses and Benefits

Participants will be paid a sum equivalent to their day wage for their study visit as agreed by the respective ethics committees. Non-working mothers will be provided a compensation as agreeable by the respective ethics committees of Vellore and The Gambia. Reasonable travel expenses for any study-related visits will be reimbursed on production of receipts, or a mileage allowance provided as appropriate. The study participants will not have to pay for any study-related procedures (OGTT, ultrasound scans and study consultations).

13.5. Annual Progress Report

The PI would submit annually, a Progress Report to the OxTREC with a copy to CTRG. Annual report will also be submitted to the funding body – The MRC, UK.

13.6. Other Ethical Considerations

The study involves non-invasive measurements of the new born. Prior informed consent will be obtained from either the father or the mother to record new-born measurements. The parents of the new born will be informed that they can withdraw the consent from obtaining their child's measurements at any time point. No personal data of the new born will be collected. New-born's will be identified by a unique study identification number and linked to the mothers' research data of the mother and father's study identification number.

14. FINANCE AND INSURANCE

14.1. Funding

The study is funded by the Medical Research Council, UK and Global Challenges Research Fund, UK for the UK and the Gambian costs and by the Department of Biotechnology (DBT), India for the Indian costs. The Indian costs will be directly dealt between DBT and CMC, Vellore. The

For peer review only

sponsor of the study is Oxford University. The funders have no role in study design; collection, management, analysis, and interpretation of data; writing of the report; and the decision to submit the report for publication.

14.2. Insurance

The University of Oxford has a specialist insurance policy in place which would operate in the event of any participant suffering harm as a result of their involvement in the research (Newline Underwriting Management Ltd, at Lloyd's of London).

15. PUBLICATION POLICY

The Investigators will be involved in reviewing drafts of the manuscripts, abstracts, press releases and any other publications arising from the study. The study will be registered and the study protocol will be made available in publically accessible websites such as ISRCTN and some national registries (Clinical Trials Registry, India). The final results of the study will be published in peer-reviewed scientific journals. Authorship will be determined in accordance with the ICMJE guidelines and other contributors will be acknowledged. For those journals that request sharing of the analyzable data sets, requests should be made to the PI-Fredrik Karpe. All publications will acknowledge that the study was funded by MRC, UK and DBT, India.

In order to disseminate our research findings with general public and study participants, we will engage with our communities, the wider public, the media, the broad scientific community and national and international agencies. We will explain the relevance of our research to our research participants and provide feedback of the results to them. We will use websites, the written press, TV and radio, and presentations to lay and school groups in order to ensure that our research findings reach the widest possible public audience. We will hold annual retention meetings of the study participants where results of the interim analysis and final study will be conveyed.

For peer review only

16. REFERENCES

1. Buchanan TA, Xiang AH. Gestational diabetes mellitus. J Clin Invest 2005;115(3):485-91.
2. Reece EA, Leguizamón G, Wiznitzer A. Gestational diabetes: the need for a common ground. Lancet 2009;373(9677):1789-97.
3. Bellamy L, Casas JP, Hingorani AD, Williams D. Type 2 diabetes mellitus after gestational diabetes: a systematic review and meta-analysis. Lancet 2009;373(9677):1773-9.
4. Qin J, Li Y, Cai Z, Li S, Zhu J, Zhang F at al. A metagenome-wide association study of gut microbiota in type 2 diabetes. Nature 2012;490(7418):55-60.
5. Sanz Y, Santacruz A, Gauffin P. Gut microbiota in obesity and metabolic disorders. Proc Nutr Soc 2010;69(3):434-41.
6. Collado MC, Isolauri E, Laitinen K, Salminen S. Distinct composition of gut microbiota during pregnancy in overweight and normal-weight women. Am J Clin Nutr 2008;88(4):894-9.
7. Monda V, Villano I, Messina A, Valenzano A, Esposito T, Moscatelli F et al. Exercise Modifies the Gut Microbiota with Positive Health Effects. Oxid Med Cell Longev 2017;2017:3831972.
8. Le Chatelier E, Nielsen T, Qin J, Prifti E, Hildebrand F, Falony G et al. Richness of human gut microbiome correlates with metabolic markers. Nature 2013;500(7464):541-6.
9. Metzger BE, Buchanan TA, Coustan DR, de Leiva A, Dunger DB, Hadden DR et al. Summary and recommendations of the Fifth International Workshop-Conference on Gestational Diabetes Mellitus. Diabetes Care 2007;30 Suppl 2:S251-60.
10. Hunt KJ, Schuller KL. The increasing prevalence of diabetes in pregnancy. Obstet Gynecol Clin North Am 2007;34(2):173-99
11. Sacks DA, Hadden DR, Maresh M, Deerochanawong C, Dyer AR, Metzger BE at al. Frequency of gestational diabetes mellitus at collaborating centers based on IADPSG consensus panel recommended criteria: the Hyperglycemia and Adverse Pregnancy Outcome (HAPO) Study. Diabetes Care 2012;35(3):526-8.
12. Hunsberger M, Rosenberg KD, Donatelle RJ. Racial/ethnic disparities in gestational diabetes mellitus: findings from a population-based survey. Womens Health Issues 2010;20(5):323-8.
13. Savitz DA, Janevic TM, Engel SM, Kaufman JS, Herring AH. Ethnicity and gestational diabetes in New York City, 1995-2003. BJOG 2008;115(8):969-78.
14. Seshiah V, Balaji V, Balaji MS, Sanjeevi CB, Green A. Gestational diabetes mellitus in India. J Assoc Physicians India 2004;52:707-11.
15. Macaulay S, Dunger DB, Norris SA. Gestational diabetes mellitus in Africa: a systematic review. PLoS One 2014;9(6):e97871.
16. Harlev A, Wiznitzer A. New insights on glucose pathophysiology in gestational diabetes and insulin resistance. Curr Diab Rep 2010;10(3):242-7.
17. Koivusalo SB, Rönö K, Klemetti MM, Roine RP, Lindström J, Erkkola M et al. Gestational Diabetes Mellitus Can Be Prevented by Lifestyle Intervention: The Finnish Gestational Diabetes Prevention Study (RADIEL): A Randomized Controlled Trial. Diabetes Care 2016;39(1):24-30.
18. Cani PD, Delzenne NM. The role of the gut microbiota in energy metabolism and metabolic disease. Curr Pharm Des 2009;15(13):1546-58.
19. Gijsbers L, Ding EL, Malik VS, de Goede J, Geleijnse JM, Soedamah-Muthu SS. Consumption of dairy foods and diabetes incidence: a dose-response meta-analysis of observational studies. Am J Clin Nutr 2016;103(4):1111-24.
20. Chen M, Sun Q, Giovannucci E, Mozaffarian D, Manson JE, Willett WC et al. Dairy consumption and risk of type 2 diabetes: 3 cohorts of US adults and an updated meta-analysis. BMC Med 2014;12:215.

For peer review only

21. Dugoua JJ, Machado M, Zhu X, Chen X, Koren G, Einarson TR. Probiotic safety in pregnancy: a systematic review and meta-analysis of randomized controlled trials of Lactobacillus, Bifidobacterium, and Saccharomyces spp. J Obstet Gynaecol Can 2009;31(6):542-52.
21. Russo LM, Nobles C, Ertel KA, Chasan-Taber L, Whitcomb BW. Physical activity interventions in pregnancy and risk of gestational diabetes mellitus: a systematic review and meta-analysis. Obstet Gynecol 2015;125(3):576-82.
22. Sanabria-Martínez G, García-Hermoso A, Poyatos-León R, Álvarez-Bueno C, Sánchez-López M, Martínez-Vizcaíno V. Effectiveness of physical activity interventions on preventing gestational diabetes mellitus and excessive maternal weight gain: a meta-analysis. BJOG 2015;122(9):1167-74.
23. Barakat R, Pelaez M, Cordero Y, Perales M, Lopez C, Coteron J et al. Exercise during pregnancy protects against hypertension and macrosomia: randomized clinical trial. Am J Obstet Gynecol 2016;214(5):649 e1-8.
24. Wiebe HW, Boulé NG, Chari R, Davenport MH. The effect of supervised prenatal exercise on fetal growth: a meta-analysis. Obstet Gynecol 2015;125(5):1185-94.
25. Barakat R, Cordero Y, Coteron J, Luaces M, Montejo R. Exercise during pregnancy improves maternal glucose screen at 24-28 weeks: a randomised controlled trial. Br J Sports Med 2012;46(9):656-61.
26. de Barros MC, Lopes MA, Francisco RP, Sapienza AD, Zugaib M. Resistance exercise and glycemic control in women with gestational diabetes mellitus. Am J Obstet Gynecol 2010;203(6):556 e1-6.
27. ACOG Committee Opinion No. 650. Physical Activity and Exercise During Pregnancy and the Postpartum Period. Obstet Gynecol 2015;126(6):e135-42.
28. Campbell SC, Wisniewski PJ. Exercise is a Novel Promoter of Intestinal Health and Microbial Diversity. Exerc Sport Sci Rev 2017;45(1):41-47.
29. Lindsay KL, Walsh CA, Brennan L, McAuliffe FM at al. Probiotics in pregnancy and maternal outcomes: a systematic review. J Matern Fetal Neonatal Med 2013;26(8):772-8.
30. Laitinen K, Poussa T, Isolauri E; Nutrition, Allergy, Mucosal Immunology and Intestinal Microbiota Group. Probiotics and dietary counselling contribute to glucose regulation during and after pregnancy: a randomised controlled trial. Br J Nutr 2009;101(11):1679-87.
31. Luoto R, Laitinen K, Nermes M, Isolauri E. Impact of maternal probiotic-supplemented dietary counselling on pregnancy outcome and prenatal and postnatal growth: a double-blind, placebo-controlled study. Br J Nutr 2010;103(12):1792-9.
32. Zheng J, Feng Q, Zheng S, Xiao X. The effects of probiotics supplementation on metabolic health in pregnant women: An evidence based meta-analysis. PLoS One. 2018 May 21;13(5):e019777
33. Hajifaraji M, Jahanjou F, Abbasalizadeh F, Aghamohammadzadeh N, Abbasi MM, Dolatkhah N. Effect of probiotic supplements in women with gestational diabetes mellitus on inflammation and oxidative stress biomarkers: a randomized clinical trial. Asia Pac J Clin Nutr. 2018;27(3):581-591.
34. Nitert MD, Barrett HL, Foxcroft K, Tremellen A, Wilkinson S, Lingwood B et al. SPRING: an RCT study of probiotics in the prevention of gestational diabetes mellitus in overweight and obese women. BMC Pregnancy Childbirth. 2013 Feb 25;13:50.
35. Barakat R, Pelaez M, Cordero Y, Perales M, Lopez C, Coteron J et al. Exercise during pregnancy protects against hypertension and macrosomia: randomized clinical trial. Am J Obstet Gynecol 2016;214(5):649 e1-8.

For peer review only

36. Barakat R, Cordero Y, Coteron J, Luaces M, Montejo R. Exercise during pregnancy improves maternal glucose screen at 24-28 weeks: a randomised controlled trial. Br J Sports Med 2012;46(9):656-61.
37. Poston L, Bell R, Croker H, Flynn AC, Godfrey KM, Goff L at al. Effect of a behavioural intervention in obese pregnant women (the UPBEAT study): a multicentre, randomised controlled trial. Lancet Diabetes Endocrinol 2015;3(10):767-77
38. da Silva SG, Hallal PC, Domingues MR, Bertoldi AD, Silveira MFD, Bassani D et al. A randomized controlled trial of exercise during pregnancy on maternal and neonatal outcomes: results from the PAMELA study. Int J Behav Nutr Phys Act. 2017 Dec 22;14(1):175.
39. Haakstad LA, Bø K. Effect of regular exercise on prevention of excessive weight gain in pregnancy: a randomised controlled trial. Eur J Contracept Reprod Health Care. 2011 Apr;16(2):116-25.
40. Wang C, Wei Y, Zhang X, Zhang Y, Xu Q, Sun Y et al. A randomized clinical trial of exercise during pregnancy to prevent gestational diabetes mellitus and improve pregnancy outcome in overweight and obese pregnant women. Am J Obstet Gynecol. 2017 Apr;216(4):340-351.
41. Vinter CA, Jensen DM, Ovesen P, Beck-Nielsen H, Jørgensen JS. The LiP (Lifestyle in Pregnancy) study: a randomized controlled trial of lifestyle intervention in 360 obese pregnant women. Diabetes Care 2011;34(12):2502-7.
42. Renault KM, Nørgaard K, Nilas L, Carlsen EM, Cortes D, Pryds O at al. The Treatment of Obese Pregnant Women (TOP) study: a randomized controlled trial of the effect of physical activity intervention assessed by pedometer with or without dietary intervention in obese pregnant women. Am J Obstet Gynecol 2014;210(2):134 e1-9.
43. Koivusalo SB, Rönö K, Klemetti MM, Roine RP, Lindström J, Erkkola M et al. Gestational Diabetes Mellitus Can Be Prevented by Lifestyle Intervention: The Finnish Gestational Diabetes Prevention Study (RADIEL): A Randomized Controlled Trial. Diabetes Care 2016;39(1):24-30.
44. Dodd JM, Turnbull D, McPhee AJ, Deussen AR, Grivell RM, Yelland LN at al. Antenatal lifestyle advice for women who are overweight or obese: LIMIT randomised trial. BMJ 2014;348:g1285.
45. Grivell RM, Yelland LN, Deussen A, Crowther CA, Dodd JM. Antenatal dietary and lifestyle advice for women who are overweight or obese and the effect on fetal growth and adiposity: the LIMIT randomised trial. BJOG. 2016 Jan;123(2):233-43.
46. Bennett CJ, Walker RE, Blumfield ML, Gwini SM, Ma J, Wang F. Interventions designed to reduce excessive gestational weight gain can reduce the incidence of gestational diabetes mellitus: A systematic review and meta-analysis of randomised controlled trials. Diabetes Res Clin Pract. 2018 Jul;141:69-79.
47. Shepherd E, Gomersall JC, Tieu J, Han S, Crowther CA, Middleton P. Combined diet and exercise interventions for preventing gestational diabetes mellitus. Cochrane Database Syst Rev. 2017 Nov 13;11:CD010443
48. Petrella E, Malavolti M, Bertarini V, Pignatti L, Neri I, Battistini NC et al. Gestational weight gain in overweight and obese women enrolled in a healthy lifestyle and eating habits program. J Matern Fetal Neonatal Med. 2014 Sep;27(13):1348-52.

For peer review only

18. APPENDIX A: STUDY FLOW CHART

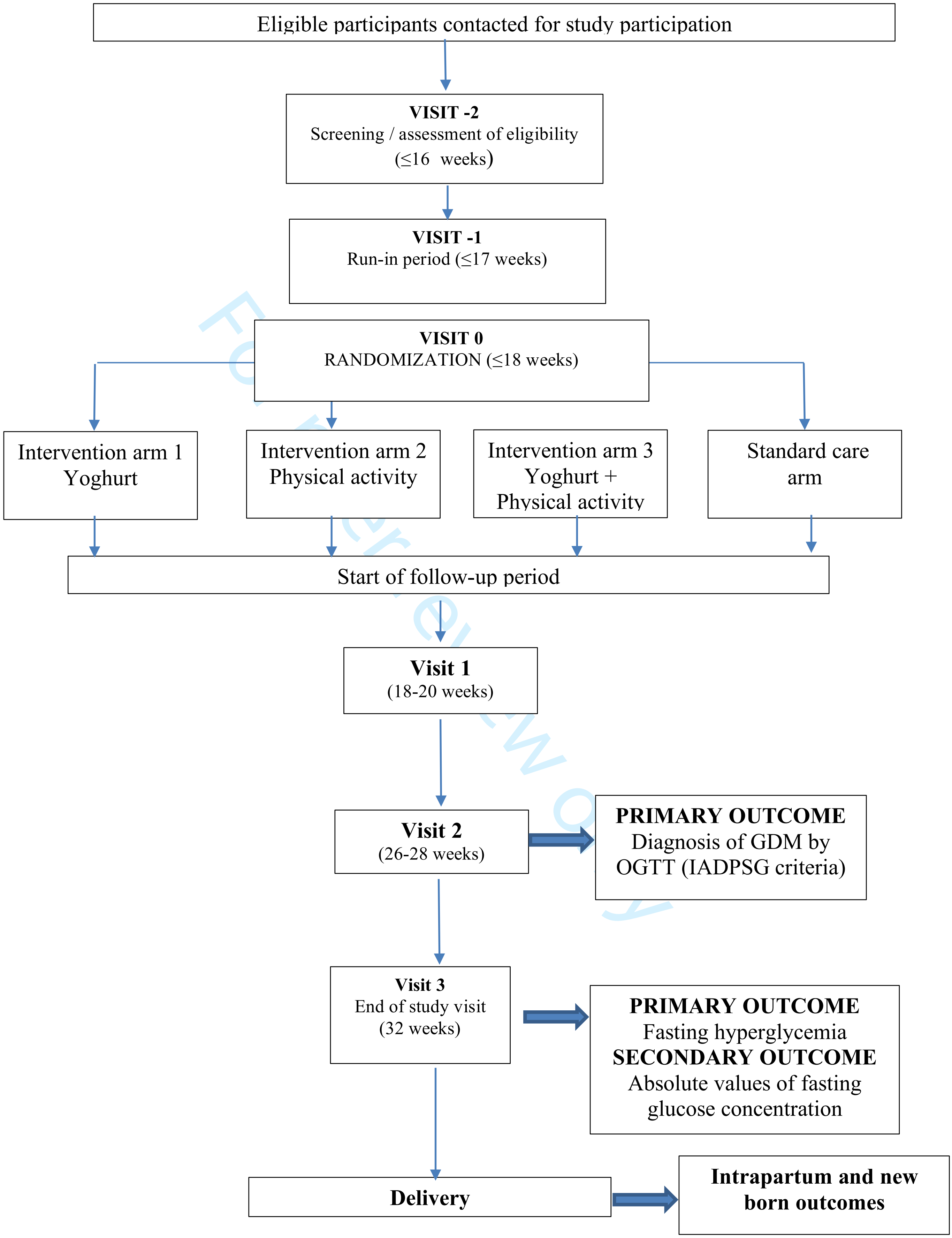

19. APPENDIX B: STUDY TIMELINES (approximate)

	2018-2019		2019		2020		2021	
	July - Nov	Dec 2018 - Jan 2019	Feb - June	Jul - Dec	Jan-Jun	Jul - Dec	Jan-Jun	Jul - Dec
Protocol preparation	■							
Regulatory approvals	■	■						
TSC meeting		■		■		■		
Investigators meeting			■		■			■
Staff training			■					
Study initiation			■					
Recruitment & follow up			■	■	■	■	■	
Study close out							■	
Interim analysis					■			
Monitoring visits				■		■		
Data quality checks				■		■		
DMC meeting				■		■		■
Annual report preparation				■		■		
Final data analysis								■
Preparation of manuscripts								■
Publication of manuscripts								■
Final report submission								■

For peer review only

20. APPENDIX C: SCHEDULE OF STUDY PROCEDURES

Procedures	Visits						
	Visit -2	Visit -1	Visit 0	Visit 1	Visit 2	Visit 3	Delivery
	Screening ≤16 weeks	Run-in-phase ≤17 weeks	Randomization Week ≤18	Week 18-20	Week 26-28	Week 32	
Informed consent	X						
Eligibility assessment	X						
Demographics	X						
History	X						
Height	X						
Weight	X	X	X	X	X	X	X
Blood pressure	X	X	X	X	X	X	X
Antenatal examination	X	X	X	X	X	X	
Dairy consumption questionnaire		X					
Barriers questionnaire	X					X	
Baseline PA monitoring with Vivofit 4		X					
PA step-count monitoring			X	X	X	X	
PA assessment (Baseline)		X					
Randomisation			X				
OGTT	X				X		
Dating scan	X						
Morphology scan				X			
Ultrasound scan						X	
Fasting plasma glucose						X	
Intrapartum assessment (mother)							X
New born assessment							X

For peer review only

21. APPENDIX D: PATIENT INFORMATION SHEET

PATIENT INFORMATION SHEET
Lifestyle interventions during pregnancy to prevent diabetes (PRIMORDIAL Study)

OxTREC reference number 44-18
Version 2.1 dated 19th September 2019

1. What's the purpose of this research?

Around 3 in 10 pregnant women are at risk for developing diabetes during pregnancy, and the risk is higher in African and Asian women. Diabetes during pregnancy imposes serious health effects to both the mother and the baby. For some women, this may be a sign of developing diabetes permanently in the future. In this study, we would like to find out, if simple lifestyle changes in diet and physical activity during pregnancy might help in reducing the risk of developing diabetes in pregnant women.

2. Why have I been invited to take part?

You are invited to take part in this study because you have been identified to have one (or more than one) condition that places you at an increased risk for developing diabetes during pregnancy. Risk factors can be being overweight or obese, previous pregnancy with diabetes, Age ≥25 years, first-degree relative with diabetes, previous pregnancy with large baby (≥3.5kg), previous pregnancy with hypertension/seizures, and history of impaired fasting glucose.

3. What's involved for me?

After study explained to you, if you agree to take part you will have to provide your consent. There are 6 visits in this study. Y our doctor may request you to attend the clinic for additional care if required. Information on what you will be asked to do at each visit are as below

Screening visit (Visit -2): At this first visit, you will be asked to do a timed blood test called the oral glucose tolerance test (OGTT), an ultrasound scan, answer a barriers questionnaire and routine clinical examination by your doctor.

A sample of your blood will be taken to measure fasting glucose (sugar). You should have not consumed any food or drink (except water) for a minimum 8 hours from previous night. You will then be given a sugary drink and subsequently blood samples will be taken at one hour and two hours from the time you have taken the drink. You cannot eat anything during the test but you can drink water. The test will take about 2 hours 15 minutes to be completed. The doctor will then perform an ultrasound scan to find out how many weeks of pregnancy are you currently in and also will be able to tell you the expected date of delivery. In addition, the doctor will take some basic measurements of the growing baby. The results of the scan will be informed to you immediately. The scans will take about 30 minutes. You will also to fill a questionnaire relating to difficulties you anticipate, that will prevent you from being physically active during pregnancy and eating healthy food including yoghurt during pregnancy. This will take about 30 minutes to complete.

You will be contacted within one week regarding your blood test and your eligibility to further continue in the study.

Run-in-phase (Visit -1): At the second visit, you will be asked questions relating to your regular milk-products and yoghurt consumption. This will take about 15 minutes for each of the questionnaire. You will also be asked to wear a watch-like device around your wrist for the next 7 days and do your routine day-to-day work. You will also be seen in the clinic by the doctor for general examination

Randomization (Visit 0): To be eligible to participate further in this study, you should have worn the wrist band on all 7 days as advised.

For peer review only

You will be allotted to one of the study arms – the yoghurt arm or the physical activity arm or the yoghurt and the physical activity arm or standard care arm. If you are allocated to the diet arm, you will have to consume a pot of yoghurt (200g) till 32 weeks of pregnancy. In the physical activity arm, you will be asked to increase your walking everyday till 32 weeks of pregnancy. In the yoghurt and physical activity arm, you will have to do both of the above. In the standard care arm, you will receive regular care from your physician.

Visit 1: In the fourth visit, the physician will perform an ultrasound scan to see the growth of the baby. Routine antenatal examination will be done by your doctor.

Visit 2: In the fifth visit, you will be asked to do blood tests similar to your first visit seen in the clinic by the doctor for routine antenatal examination.

Visit 3: A fasting blood sample (minimum 8-10 hours fasting from previous night) for blood glucose measurement will be taken and another scan to see if your baby is growing well. You will also be asked to complete a barriers questionnaire and provide feedback about your participation in the trial. All other routine antenatal care will be provided by your doctor.

This is the last visit and all interventions stop here. You will be contacted during delivery for measurements of yourself and new-born baby during delivery.

In the event that you develop diabetes at any point during the study, your physician will advise you on additional medications.

During the entire period you will be home visited by a field worker or telephonically contacted by a research staff to check if everything is ok.

4. What will happen to my data?

Research data You will be given a study number so that any information that you provide to us will be made anonymous. All data will be securely stored in the research office, kept in locked cupboards/secure servers in locked rooms with restricted access. All data will be owned by your local research investigator, kept in locked cupboards/secure servers in locked rooms with restricted access. Anonymised data will be shared between researchers in the Gambia, India, and Oxford, UK for research purposes. All research data collected as electronic records will be kept indefinitely in electronic data archives in The Gambia and may be used for future research studies with your consent. Responsible members of the research team may be given access to data for monitoring and/or audit of the study to ensure that the research is complying with applicable regulations.
Personal data Your name, date of birth, address and telephone number will be stored separately in order to invite you to follow-up visits and for the field staff to visit you at home. This information will not be shared with other researchers.

5. What will happen to my samples?

The blood samples will be used for study purpose only and analysed locally. Samples will not be shipped/stored overseas and will not be shared with other researchers. No genetic tests are carried out in this study.

6. Are there any risks in taking part?

There may be mild bruising/swelling in the area where blood is drawn. Consumption of oral glucose solution in empty stomach may cause nausea, vomiting, headache. Ultrasound scans during pregnancy are considered safe to the mother or the baby as the radiation exposure is minimal.

By consuming yoghurt, you may feel nauseated, experience vomiting, bowel upset or diarrhoea. Physical activity during pregnancy is considered generally safe. However, you may experience some common adverse effects like dizziness, headache, and breathlessness on exertion, easy fatigability and muscle aches. If either of the interventions is not tolerable, you can contact your doctor and he may stop the intervention if necessary.

For peer review only

7. What are the benefits of taking part?

The direct benefits of participating in this study is that your blood sugar levels, blood pressure and weight will be closely monitored. By achieving strict control of your sugar levels, blood pressure and weight, we hope to see immediate benefits and lifelong health impact on yourself and your baby.
You will be sensitised to healthy lifestyle during pregnancy.

8. Do I have to take part?

Participation in this study is voluntary. You can ask questions/concerns about the study to one of the members of the research team before deciding whether to take part. Your decision will not affect your routine clinical care. You can also withdraw from the study without penalty at any time.

10.Has the study been reviewed by an ethics committee?

The study has been reviewed and approved by the ethics committees of University of Oxford, UK, Christian Medical College & Hospital, Vellore, India and the MRC Unit, The Gambia.

11.What if I have any questions or want to raise a concern?

If you have any concerns about the study at any point or wish to complain about any aspect of the way in which you have been approached or treated in this study, you may contact one of the below mentioned persons

Research team:
Name:
Address:
Phone number:

Member independent of the study team
Name
Address:
Phone number:

12. Data protection
The University of Oxford is responsible for ensuring the safe and proper use of any personal information you provide, solely for research purposes.

For peer review only

22. APPENDIX E: INFORMED CONSENT FORM

INFORMED CONSENT FORM

Lifestyle interventions during pregnancy to prevent diabetes (PRIMORDIAL Study)

OxTREC reference number 44-18

1. I confirm that I have read the information sheet for the above study. I have had the opportunity to consider the information and ask questions, and have had these answered satisfactorily.	Yes / No
2. I give consent for my research data to be stored locally and shared with other investigators overseas	Yes / No
3. I agree to take part in this study.	Yes / No

Name of participant   Date   Signature

Name of person taking consent   Date   Signature

- For illiterate participants:

Name of participant   Thumbprint of participant

Name of witness   Relationship to participant   Date   Signature

Name of person taking consent   Date   Signature

23. APPENDIX F: AMENDMENT HISTORY

All protocol amendments whenever a new version of the protocol is produced will be updated here.

Amendment No.	Protocol Version No.	Date issued	Author(s) of changes	Details of Changes made
1	2.1	19th October 2018	Senthil K Vasan<br>Fredrik Karpe<br>Caroline Fall<br>Clive Osmond	1. Stratification for risk factors during randomization (Section 7.2)<br>2. Explanations to sample size calculation (Section 10.2)<br>3. Study analysis details included unde section 10.3.1 and 10.3.2<br>4. Details of DSMB included under section 12.<br>5. SAE reporting to DSMB and OxTREC is included under section 9.3.<br>6. Skin fold thickness measurement of the new born is removed from the protocol<br>7. Under Section 13,4, the following line is changed "Participants will be paid a sum equivalent to their day wage for their study visit as agreed by the respective ethics committees".
2	3.0	09th January 2019	Senthil K Vasan<br>Fredrik Karpe<br>Caroline Fall<br>Clive Osmond<br>Dylan Thompson	1. Additional change to primary outcome measure (eelevated fasting plasma glucose ≥5.1 mmol/l at 32 weeks GA) in Page 4<br>2. Secondary objective changed tc "To determine the effect of life style intervention (yoghurt and PA), given to high-risk women from 18 weeks of gestation, on fasting blood glucose at 32 weeks (Pages 4 and 16)<br>3. Low birth weight changed to 'birth weight' under section other outcome measures in Page 5

For peer review only

				4. Ponderal index and neonatal anthropometry deleted under neonatal outcomes in Pages 5, 16, 19, 27)<br>5. Run-in visit - Baseline PA will be assessed (blinded to participants) using a wearable accelerometer device (Garmin Vivofit 4 fitness band) for 7 days to objectively measure baseline step count in all participants (Page 17)<br>6. Visit 3 (32 weeks GA, End of Study visit): morphology scan changed to ultrasound scan (Pages 17, 26)<br>7. Physical actvity intervention The recommendation changed to 80% of the absolute target increase from the average target step count measured during run-in-phase (Pages 18 and 29)<br>8. Group session will use a patient participant initiative (PPI) approach where participants wi be involved in designing the type of group activity, which may include activities such as group walking, dancing, stretching exercises etc. (Page 18)<br>9. Reassessment of PA activity: All participants will be asked to objectively measure step count for next 7 days using Garmin Vivofit4 at visit 2 (26-28 weeks) Women not randomized to the diet and standard care arm will have blinded assessments, whil women on PA arm (PA arm or Diet+PA) will continue to achieve target step counts without blinded assessment (Page 18).

For peer review only

Date and version No: 18th July 2019, Version 4.2

					10. Data collection process – height and weight of father newly added (Pages 19, 21, 23-24)<br>11. Inclusion criteria changed to ≥1 weeks and ≤ 16 weeks of gestational age (Pages 20,23)<br>12. Inclusion criteria stamen revise as: In addition to the above, pregnant women should meet at least one of the following criteria for high-risk GDM (Page 20).<br>13. Exclusion criteria related to hypertension changed as "Currently on treatment for hypertension (pre-gestational o gestational)" (Page 20).<br>14. New exclusion criteria added: Physical disability to PA and/or known lactose intolerance (pag 21).<br>15. Details of stratification at randomisation provided: Random allocation to intervention will be prestratifie for age, BMI and risk factor for GDM within each study centre. We will create two age bands (<25 y and ≥ 25 years), two BMI categories (<25, ≥26) and presence of any risk factor (No 0/ Yes 1) (Page 23).<br>16. PA intervention details and 80% absolute target step count char included in Page 29.<br>17. PA compliance definition included in Page 29 as "compliance" will be defined as will be defined as at least 80% c absolute target increase achieved on an average over 7- days period.<br>18. Statistical methods changed as "The primary analysis will be intention-to-treat. We will also

For peer review only

				do a secondary analysis using the per protocol principle" in page 31.<br>19. Members of the executive committee newly included in Pages 36-37.<br>20. Appendix A – study flow chart Primary and secondary outcomes changed in Page 42.<br>21. Timelines of investigators meeting, study initiation, staff training in Gnat chart changed i Page 43
3	4.0	09th February 2019	Senthil K Vasan<br>Fredrik Karpe<br>(Changes as suggested from TSC)	1. Primary objective – outcome measure: 26-28 weeks included newly<br>2. 'Other outcome measures' rename Trial and post-trial surveillance' (Sections 1, 4).<br>3. maternal blood pressure added as objective and serial blood pressure measurement at all visits included a outcome measurement (Sections 1, 4).<br>4. For maternal outcome measurements under 'Trial and post-trial surveillance' data on intra and postpartum outcomes will be captured on eCRF from delivery records (Section 1).<br>5. New born outcomes measure preterm births will be based on ultrasound based gestational age recorded at screening (Section 1).<br>6. DSMB changed to DMC (data monitoring committee) in abbreviations. New abbreviations added – LSHTM, PID (Section 2).<br>7. Protocol change: Under Section 5 – Run-in-visit – consumption of yoghurt by all participants for 7 day removed.

For peer review only

				8. Protocol change: Section 5 – Run-in visit and in data collection process, Section 7: Baseline dietary assessment using FFQ changed to background dairy consumption questionnaire.<br>9. Under Section 5 and Section 7– Visits 1-3: ‘routine’ added to antenatal examination.<br>10. Under Section 5 – Visit 3: GDM women will continue on allocatec study intervention till week 32 in addition to the treatment provided as per local obstetric practice. This i changed from previous version that stated ‘terminating women who develop GDM from further intervention’.<br>11. Protocol change: Under Section 5 - Interventions: Newly added: All other (yoghurt and standard arm) will also be provided with fitness bands to objectively measure their routine PA behaviour throughout the study. However, step counts wil be blinded for these participants.<br>12. Under Section 6.2 – Medications revised to include women who are hypothyroid on thyroxine supplementation, low dose aspirin for pre-eclampsia) and women conceived by ovulation induction using clomiphene citrate or metformin for PCOD will not be excluded from the study.<br>13. Section 6.3 – Exclusion criteria – GDM diagnosed prior to screening visit based on IADPSG criteria or documented raised HbA1C.<br>14. Section 7 – Post-trial surveillance – Capillary blood glucose (mother) deleted.<br>15. Section 7.3 – Screening and Eligibility assessment: Newly added ‘In addition to above, participants

For peer review only

				should meet at least one of the below mentioned criteria to be termed as "high-risk" for inclusion into the study'<br>16. Section 7.4 – Randomisation: Newly added: After qualifying for eligibility and completion of the run-in-visit, details of age, BMI and GDM risk wi[illegible] be provided to the independent statistician, who will then assign the intervention arm. Randomisation allocation will be communicated to the study sites through emails.<br>17. Section 7.6.1 – Newly added under morphology scan - Participants with any foetal congenital anomaly, placental malformation or malposition or abnormal ultrasound features that can compromise pregnancy outcome according to th[illegible] investigator will be withdrawn from the study at this stage.<br>18. Section 7.6.1, 7.6.2, 7.6.3: Change made - Safety assessments to interventions (e.g. adverse events and/or SAE assessment).<br>19. Section 7.8.1 – more details provided for "Discontinuation/withdrawal"<br>20. Section 7.8.2 – Newly added "discontinuation from interventions".<br>21. Section 7.9 - more details provided for "lost to follow-up".<br>22. Section 7.11 – last scheduled visit changed from Visit 2 (26-28 weeks) to visit 3 (week 32).<br>23. Section 8.1.2 – Details of PA intervention provided under sections 8.1.2.1 – daily walking and 8.1.2.2 – Group activity sessions.<br>24. Section 9.1 – Details of AE reporting in eCRF added as 'Details of all events including onset date, causality to the study intervention

For peer review only

				will be reported, treatment given, stop date will be captured in the respective AE report form'.<br>25. Section 9.2.1 – Expected SAE – newly added.<br>26. Section 12 title changed as "Committee structures"<br>27. Section 12.1., 12.2., 12.3 – Roles an responsibilities of DMC, TSC and executive committees are included.<br>28. Section 13.1 renamed as ICH-GCP.<br>29. Section 15 – Publication Policy: Registration with ISRCTN and Clinica Trials Registry, India newly added.<br>30. Section 15 - Publication Policy: Fredrik Karpe will be the contact person for requests for sharing analysable data.<br>31. PPAQ removed at run-in-visit.
4	4.1	09th April 2019	Senthil K Vasan<br>Alexander Jarde<br>Fredrik Karpe	1. Gestational age at screening, run-in and randomisation visit are changed from 16, 17 and 18 weeks to ≤16, ≤17 and ≤18 weeks (Pages 17, 20-25)<br>2. Detailed randomisation procedure outlined in Page 23. Risk factors for GDM is removed from pre-stratification criteria.<br>3. Section 10 on Statistics and analysis (incl. 10.1 and 10.2) are expanded to provide more details (Pages 33-35)
5	4.2	18th July 2019	Senthil K Vasan<br>Fredrik Karpe	1. Scheduled visit 1 is changed form week 22 to week 18-2 weeks. This is in line with ACOG and NICE antenatal guidelines for morphology scan at week 18-20 (Pages 17,21,25,46)

For peer review only

STANDARD PROTOCOL ITEMS: RECOMMENDATIONS FOR INTERVENTIONAL TRIALS

SPIRIT 2013 Checklist: PRIMORDIAL Study

Section/item	PageNo	Description
**Administrative information**		
Title	1	Descriptive title identifying the study design, population, interventions, and, if applicable, trial acronym
Trial registration	1	Trial identifier and registry name. If not yet registered, name of intended registry
Protocol version	1	Date and version identifier
Funding	1	Sources and types of financial, material, and other support
Roles and responsibilities	1	Names, affiliations, and roles of protocol contributors
	1	Name and contact information for the trial sponsor
	42	Role of study sponsor and funders, if any, in study design; collection, management, analysis, and interpretation of data; writing of the report; and the decision to submit the report for publication, including whether they will have ultimate authority over any of these activities
	39-41	Composition, roles, and responsibilities of the coordinating centre, steering committee, endpoint adjudication committee, data management team, and other individuals or groups overseeing the trial, if applicable (see Item 21a for data monitoring committee)
**Introduction**		
Background and rationale	9-16	Description of research question and justification for undertaking the trial, including summary of relevant studies (published and unpublished) examining benefits and harms for each intervention
Objectives	10,17	Specific objectives or hypotheses
Trial design	18	Description of trial design including type of trial (eg, parallel group, crossover, factorial, single group), allocation ratio, and framework (eg, superiority, equivalence, noninferiority, exploratory)
**Methods: Participants, interventions, and outcomes**		
Study setting	22-23	Description of study settings (eg, community clinic, academic hospital) and list of countries where data will be collected. Reference to where list of study sites can be obtained

For peer review only

Eligibility criteria	21	Inclusion and exclusion criteria for participants. If applicable, eligibility criteria for study centres and individuals who will perform the interventions (eg, surgeons, psychotherapists)
Interventions	19, 31	Interventions for each group with sufficient detail to allow replication, including how and when they will be administered
	29	Criteria for discontinuing or modifying allocated interventions for a given trial participant (eg, drug dose change in response to harms, participant request, or improving/worsening disease)
Outcomes	17, 25, 35	Primary, secondary, and other outcomes, including the specific measurement variable (eg, blood glucose, OGTT), analysis metric (eg, change from baseline, final value, time to event), method of aggregation (eg, median, proportion), and time point for each outcome. Explanation of the clinical relevance of chosen efficacy and harm outcomes is strongly recommended
Participant timeline	22-26	Time schedule of enrolment, interventions (including any run-ins and washouts), assessments, and visits for participants. A schematic diagram is highly recommended (see Figure)
Sample size	34,35	Estimated number of participants needed to achieve study objectives and how it was determined, including clinical and statistical assumptions supporting any sample size calculations
Recruitment	22,23	Strategies for achieving adequate participant enrolment to reach target sample size

Methods: Assignment of interventions (for controlled trials)

Allocation:

Sequence generation	24	Method of generating the allocation sequence (eg, computer-generated random numbers), and list of any factors for stratification. To reduce predictability of a random sequence, details of any planned restriction (eg, blocking) should be provided in a separate document that is unavailable to those who enrol participants or assign interventions
Implementation	24	Who will generate the allocation sequence, who will enrol participants, and who will assign participants to interventions

Methods: Data collection, management, and analysis

Data collection methods	37-38	Plans for assessment and collection of outcome, baseline, and other trial data, including any related processes to promote data quality (eg, duplicate measurements, training of assessors) and a description of study instruments (eg, questionnaires, laboratory tests) along with their reliability and validity, if known. Reference to where data collection forms can be found, if not in the protocol

For peer review only - http://bmjopen.bmj.com/site/about/guidelines.xhtml

Data management	36-38	Plans for data entry, coding, security, and storage, including any related processes to promote data quality (eg, double data entry; range checks for data values). Reference to where details of data management procedures can be found, if not in the protocol
Statistical methods	33-36	Statistical methods for analysing primary and secondary outcomes. Reference to where other details of the statistical analysis plan can be found, if not in the protocol
	35,36	Methods for any additional analyses (eg, subgroup and adjusted analyses)
	33,36	Definition of analysis population relating to protocol non-adherence (eg, as randomised analysis)
**Methods: Monitoring**		
Data monitoring	39	Composition of data safety monitoring committee (DSMC); summary of its role and reporting structure; statement of whether it is independent from the sponsor and competing interests; and reference to where further details about its charter can be found, if not in the protocol. Alternatively, an explanation of why a DMC is not needed
Harms	32	Plans for collecting, assessing, reporting, and managing solicited and spontaneously reported adverse events and other unintended effects of trial interventions or trial conduct
**Ethics and dissemination**		
Research ethics approval	41	Plans for seeking research ethics committee/institutional review board (REC/IRB) approval
Protocol amendments	40,54-60	Plans for communicating important protocol modifications (eg, changes to eligibility criteria, outcomes, analyses) to relevant parties (eg, investigators, REC/IRBs, trial participants, trial registries, journals, regulators)
Consent or assent	23	Who will obtain informed consent or assent from potential trial participants or authorised surrogates, and how
Confidentiality	38	How personal information about potential and enrolled participants will be collected, shared, and maintained in order to protect confidentiality before, during, and after the trial
Access to data	38	Statement of who will have access to the final trial dataset, and disclosure of contractual agreements that limit such access for investigators

For peer review only

Dissemination policy	43	Plans for investigators and sponsor to communicate trial results to participants, healthcare professionals, the public, and other relevant groups (eg, via publication, reporting in results databases, or other data sharing arrangements), including any publication restrictions
	43	Authorship eligibility guidelines and any intended use of professional writers
Biological specimens	28	Plans for collection, laboratory evaluation, and storage of biological specimens for genetic or molecular analysis in the current trial and for future use in ancillary studies, if applicable

*It is strongly recommended that this checklist be read in conjunction with the SPIRIT 2013 Explanation & Elaboration for important clarification on the items. Amendments to the protocol should be tracked and dated. The SPIRIT checklist is copyrighted by the SPIRIT Group under the Creative Commons "Attribution-NonCommercial-NoDerivs 3.0 Unported" license.

For peer review only

BMJ Open

Protocol for a randomised controlled trial: Pregnancy Related Interventions in Mothers at risk for gestational Diabetes in Asian India and Low and middle income countries (PRIMORDIAL Study)

Journal:	*BMJ Open*
Manuscript ID	bmjopen-2020-042069.R2
Article Type:	Protocol
Date Submitted by the Author:	26-Jan-2021
Complete List of Authors:	Vasan, Senthil; University of Oxford, Oxford Centre for Diabetes, Endocrinology and Metabolism; University of Southampton, MRC Lifecourse Epidemiology Unit<br>Jobe, Modou ; Medical Research Council The Gambia<br>Mathews, Jiji; Christian Medical College and Hospital Vellore, Obstetrics and Gynaecology<br>Cole , Fatoumata; MRC Unit The Gambia at LSHTM<br>Rathore, Swathi; Christian Medical College and Hospital Vellore, Obstetrics and Gynaecology<br>Jarjou, Ousman; MRC Unit The Gambia at LSHTM, Nutrition<br>Thompson, Dylan; University of Bath, Department for Health<br>Jarde, Alexander; MRC Unit The Gambia at LSHTM, Statistics<br>Bittaye, Mustapha ; MRC Unit The Gambia at London School of Hygiene & Tropical Medicine<br>Ulijaszek, Stanley; University of Oxford, School of Anthropology<br>Fall, Caroline ; University of Southampton, MRC Lifecourse Epidemiology Unit<br>Osmond, Clive; University of Southampton, MRC Lifecourse Epidemiology Unit<br>Prentice, Andrew; London School of Hygiene & Tropical Medicine, MRC International Nutrition Group<br>Karpe, Fredrik; University of Oxford, Oxford center for Diabetes, Endocrinology and Metabolism
**Primary Subject Heading**:	Diabetes and endocrinology
Secondary Subject Heading:	Epidemiology, Obstetrics and gynaecology, Nutrition and metabolism, Diabetes and endocrinology
Keywords:	OBSTETRICS, PREVENTIVE MEDICINE, PUBLIC HEALTH, Diabetes in pregnancy < DIABETES & ENDOCRINOLOGY, Maternal medicine < OBSTETRICS

SCHOLARONE™
Manuscripts

I, the Submitting Author has the right to grant and does grant on behalf of all authors of the Work (as defined in the below author licence), an exclusive licence and/or a non-exclusive licence for contributions from authors who are: i) UK Crown employees; ii) where BMJ has agreed a CC-BY licence shall apply, and/or iii) in accordance with the terms applicable for US Federal Government officers or employees acting as part of their official duties; on a worldwide, perpetual, irrevocable, royalty-free basis to BMJ Publishing Group Ltd ("BMJ") its licensees and where the relevant Journal is co-owned by BMJ to the co-owners of the Journal, to publish the Work in this journal and any other BMJ products and to exploit all rights, as set out in our licence.

The Submitting Author accepts and understands that any supply made under these terms is made by BMJ to the Submitting Author unless you are acting as an employee on behalf of your employer or a postgraduate student of an affiliated institution which is paying any applicable article publishing charge ("APC") for Open Access articles. Where the Submitting Author wishes to make the Work available on an Open Access basis (and intends to pay the relevant APC), the terms of reuse of such Open Access shall be governed by a Creative Commons licence – details of these licences and which Creative Commons licence will apply to this Work are set out in our licence referred to above.

Other than as permitted in any relevant BMJ Author's Self Archiving Policies, I confirm this Work has not been accepted for publication elsewhere, is not being considered for publication elsewhere and does not duplicate material already published. I confirm all authors consent to publication of this Work and authorise the granting of this licence.

For peer review only

Protocol for a randomised controlled trial: Pregnancy Related Interventions in Mothers at risk for gestational Diabetes in Asian India and Low and middle income countries (PRIMORDIAL Study)

Senthil K Vasan[1,2], Modou Jobe[3], Jiji Mathews[4], Fatoumata Cole[3], Swathi Rathore[4], Ousman Jarjou[3], Dylan Thompson[5], Alexander Jarde[3], Mustapha Bittaye[3], Stanley J Ulijaszek[6], Caroline H D Fall[2], Clive Osmond[2], Andrew M Prentice[3], Fredrik Karpe[1,7]

[1]Oxford Centre for Diabetes, Endocrinology and Metabolism (OCDEM), University of Oxford Churchill Hospital, Oxford OX3 7LE, UK

[2]MRC Lifecourse Epidemiology Unit, Southampton General Hospital, University of Southampton, Tremona Road, Southampton, UK

[3]MRC Unit The Gambia at London School of Hygiene & Tropical Medicine, Fajara, The Gambia

[4]Department of Obstetrics & Gynaecology, Christian Medical College & Hospital, Vellore. Tamil Nadu, India

[5]Department for Health, Centre for Therapeutic Innovation, University of Bath, UK

[6]Institute of Social and Cultural Anthropology, University of Oxford, Oxford, UK

[7]NIHR Oxford Biomedical Research Centre, OUH Foundation Trust, Oxford, UK

Key words: gestational diabetes, yoghurt, physical activity, Africa, India, randomised controlled trial

Corresponding author:
Prof Fredrik Karpe
Oxford Centre for Diabetes, Endocrinology and Metabolism (OCDEM),
University of Oxford
Churchill Hospital
Oxford OX3 7LE
UK
Email: Fredrik.Karpe@ocdem.ox.ac.uk
Tel: +44-1865-857222

For peer review only

Abstract

Introduction: Lifestyle modification is the mainstay of gestational diabetes mellitus (GDM) prevention. However, clinical trials evaluating the safety and efficacy of diet or physical activity (PA) in low-and middle-income settings such as Africa and India are lacking. This trial aims to evaluate the efficacy of yoghurt consumption and increased PA (daily walking) in reducing GDM incidence in high-risk pregnant women.

Methods and analysis: The study is a 2x2 factorial, open-labelled, multi-centre randomized controlled trial to be conducted in Vellore, South India and The Gambia, West Africa. "High-risk" pregnant women (n=1,856) aged ≥18 years and ≤16 weeks of gestational age (GA), with at least one risk factor for developing GDM, will be randomized to either (i) yoghurt (ii) PA (iii) yoghurt + PA or (iv) standard antenatal care. Participants will be followed until 32 weeks of gestation with total active intervention lasting for a minimum of 16 weeks. The primary endpoint is GDM incidence at 26-28 weeks diagnosed using International Association of the Diabetes and Pregnancy Study Groups (IADPSG) criteria or elevated fasting glucose (≥5.1 mmol/l) at 32 weeks. Secondary endpoints include absolute values of fasting plasma glucose concentration at 32 weeks gestation, maternal blood pressure, gestational weight gain, intrapartum and neonatal outcomes. Analysis will be both by intention to treat and per-protocol. Continuous outcome measurements will be analysed using multiple linear regression and binary variables by logistic regression.

Ethics and dissemination: The study is approved by Oxford Tropical Research Ethics Committee (OxTREC 44-18), ethics committees of the Christian Medical College, Vellore (IRB 11367) and MRCG Scientific Coordinating Committee (SCC 1645) and The Gambia Government/MRCG joint ethics committee (L2020.E15). Findings of the study will be published in peer-reviewed scientific journals and presented in conferences.

Trial registration number: ISRCTN: 18467720 and Clinical Trials Registry, India (CTRI/2018/07/014947)

Word count:
Abstract: 294
Manuscript: 4194

For peer review only

For peer review only - http://bmjopen.bmj.com/site/about/guidelines.xhtml

Strengths and limitations of the study

- This study will be the largest lifestyle intervention trial among high-risk GDM mothers in India and Africa to evaluate the efficacy of daily yoghurt consumption and physical activity in reducing GDM incidence.
- The robust factorial design allows to evaluate the effectiveness of independent and combined effects of yoghurt and physical activity on GDM incidence as well as test for interactions simultaneously.
- The inclusion of participants from two different low- and middle-income settings with diverse cultural background, socioeconomic status, varied life-style behaviour and access to health care, will provide robust evidence of generalisability of these results across countries.
- Due to the nature of interventions, it is not possible to blind the study participants or research investigators, which may introduce bias and increase the risk of confounding.
- The selective screening and intervention in high-risk women using low-cost interventions is suitable for GDM prevention in low-resource settings such as India

For peer review only

BACKGROUND

Gestational Diabetes Mellitus (GDM) accounts for 86% of hyperglycaemia during pregnancy[1] and is associated with significant intrapartum complications, perinatal morbidity, and long-term risk of developing type 2 diabetes (T2D) and cardiovascular disease in both the mother and the offspring.[2-4] Consequent to the global rise in obesity prevalence and the introduction of more stringent diagnostic criteria by the International Association of the Diabetes and Pregnancy Study Groups (IADPSG), the global prevalence of GDM has been estimated at 17.8% (range 9.3–25.5%).[5] In low- and middle-income countries (LMICs), especially in India and Africa, there are few nation-wide estimates of GDM burden. Cross-sectional studies using variable diagnostic criteria report GDM prevalence ranging between 10 to 16% in India[6 7] and 11 to 14% in Africa[8 9], which is higher than the reported prevalence from high income countries like the UK (2-3% using NICE guidelines) and USA (2-10% using various criteria including IADPSG).[10 11] Direct comparisons of these estimates may not be appropriate due to inconsistency in diagnostic criteria. Nevertheless, it seems clear that LMICs are facing a high burden of GDM.

In normal pregnancy, the physiological response to reduced insulin-mediated glucose removal is an increase in insulin secretion to maintain normoglycaemia.[12] In GDM, insufficient β-cell plasticity leads to an inability to secrete adequate quantities of insulin to counterbalance the insulin resistance (IR), and this process is accelerated in obesity. Women with chronic IR states such as pre-pregnancy excess body weight or obesity, impaired glucose tolerance prior to pregnancy, polycystic ovarian syndrome (PCOS), previous GDM, advanced maternal age and rapid gestational weight gain (GWG), have a higher risk for developing glucose intolerance during pregnancy.[13] The hyperglycaemia of GDM rapidly abates following delivery and normoglycaemia normally returns within 12 weeks. However, 50-70% of GDM mothers progress to T2D within 5-10 years postpartum[14] suggesting that GDM may be a prodrome of "common T2D" and may reflect the underlying T2D frequency in the community.[15]

Screening for GDM

Following the demonstration of a linear relationship between maternal glycaemia and obstetric and perinatal outcomes by the Hyperglycemia and Adverse Pregnancy Outcome (HAPO) study,[16 17] the IADPSG recommends universal screening of all women at first antenatal visit, without restriction to high-risk pregnant women, in order to identify all potential GDM cases.[18] However, some countries have adopted selective screening, despite the possibility of missing over 40% of GDM cases.[19] In low economic setting like the LMICs, this approach may be more cost effective and oral glucose tolerance test (OGTT) may be less beneficial to low risk women.[20]

GDM management

The main goal of lifestyle modification using diet and PA in pregnancy is to prevent excessive GWG, improve materno-foetal outcomes and prevent the onset/subsequent progression to GDM/T2D in both the mother and the new born.[21] In order to maximize the benefit of such interventions, the best strategy is likely to target high-risk populations by initiating early interventions during the "critical window", i.e. before insufficient β-cell plasticity sets in[22] (**Figure 1**).

For peer review only

Dietary interventions in Pregnancy

Meta-analysis of dietary intervention trials in pregnant women, have shown that diet modification in any form resulted in a 61% risk reduction in GDM incidence (relative risk (RR) 0.39, 95%CI 0.23-0.69).[23-26] Besides macronutrient modification, dairy products, particularly yoghurt has been shown to lower the risk of T2D, [27] through mechanisms such as reduction in inflammatory signaling and gut permeability giving rise to a range of putative signals from the gut to the systemic circulation which ameliorate metabolic endotoxemia.[28-30] Differences in gut microbial composition between healthy pregnant and GDM women provide evidence of a potential role of altered gut microbiota in pregnancy-related metabolic dysfunction.[31 32]

Although exact mechanisms of the positive effects of the manipulating the gut microbiome during pregnancy is not studied, evidence from epidemiological data suggest that using fermented dairy products or probiotics prior to and during pregnancy has positive impact on maternal weight, blood pressure, insulin resistance and plasma lipid profile.[31 33-37] The "Probiotics and Pregnancy outcome" study showed that incident GDM was lower in women supplemented with probiotics compared to diet/placebo or control groups (16% vs 36% vs 34%).[38] The Nutrition, Allergy, Mucosal immunology and Intestinal microbiota (NAMI) study showed reductions in glucose and insulin concentration, GDM incidence and central adiposity, possibly mediated by anti-inflammatory cytokine production in probiotic supplemented pregnant women.[39 40] Multiple pieces of evidence also support a positive relationship between probiotic yoghurt supplementation during pregnancy and improved glycaemic profile in women with GDM or obesity.[41-43]

Physical activity in pregnancy

PA directly impacts non-insulin mediated glucose utilization through translocation of glucose transporter type 4 (GLUT 4) on skeletal muscle.[44 45] PA during pregnancy is safe and ACOG guidelines recommend ≥30 minutes of moderate PA performed at an intensity of 3-6 metabolic equivalents, which corresponds to brisk walking at 5-7km/h on most days of the week.[46]

Evidence of a beneficial effect from PA-intervention during pregnancy mostly come from high-income countries and include a wide range of exercise programme that vary in their intensity, duration, frequency and assessment techniques. Results show that regular PA in any form improves maternal (hyperglycaemia, hypertension, weight gain and cardiovascular function)[47], obstetric (pre-eclampsia, shoulder dystocia, preterm births), and neonatal outcomes (low birth weight or small-for-gestational age, decreased fat mass, improved stress tolerance and advanced neurobehavioural maturation).[48]

Meta-analysis of RCTs of PA intervention (8 studies, n=1,441) in obese/overweight women has shown a 24% reduction in GDM incidence in the intervention groups (RR 0.76, 95% CI 0.56-1.03; p=0.07) with moderate heterogeneity between studies (I^2=50%, p=0.05).[49] Some studies have shown negative or neutral effects, including a Cochrane review (5 studies, n=1,115) of PA on GDM incidence,[50-52] and this is likely to be related to study heterogeneity, bias, type of exercise, timing, duration and intensity of exercise and also subjective assessments. Nevertheless, the potential beneficial effects of effective PA intervention in pregnancy should not be underestimated.

For peer review only - http://bmjopen.bmj.com/site/about/guidelines.xhtml

The knowledge gap on effectiveness of PA interventions in LMIC settings is particularly challenging in women from Asian Indian and African backgrounds where the baseline PA level is generally considered low. The ability to engage pregnant women from diverse ethnic groups will also be determined by population-specific socioeconomic and cultural factors.

Combined approaches in Pregnancy

Targeting multiple risk factors simultaneously may have a synergistic effect to reduce GDM. Combined approaches have shown favourable improvements in maternal and foetal outcomes, particularly GWG, which is a potentially modifiable risk factor for GDM development.[53-57] Two RCTs using combined interventions in high-risk women (BMI ≥ 30kg/m^2), the UPBEAT[58] and RADIEL trial,[59] showed 0.55 kg and 0.58 kg reduction in GWG, respectively. The RADIEL trial additionally showed about 36% reduction in GDM incidence (crude RR 0.64; 95% CI 0.38–1.09) in the intervention group. A recent Cochrane review of combined interventions showed a tendency towards reduced GDM risk (RR 0.85, 95%CI 0.71-1.01; 6,633 women, 19 RCTs) and reduced weight gain (mean difference -0.89 kg, 95%CI -1.39 to -0.40; 5,052 women, 16RCTs).[60]

HYPOTHESIS

We hypothesize that daily yoghurt consumption and/or increased PA will reduce the risk of developing GDM in 'high-risk' pregnant women.

AIMS

To examine if daily yoghurt consumption and/or PA beginning from ≤16 weeks of gestation in 'high-risk' pregnant women can reduce the incidence of GDM in two LMIC settings.

OBJECTIVES

Primary objective

To establish the efficacy and safety of low-cost interventions (fermented yoghurt consumption/daily walking/both) to prevent GDM in two LMIC settings.

Secondary objectives

(i) To evaluate the effect of yoghurt/daily walking/combination of both on fasting glucose concentration at 32 weeks of gestation, GWG, blood pressure, intrapartum and neonatal outcomes.
(ii) To identify barriers that prevent participation/adherence to lifestyle changes during pregnancy and thus design culturally and socially acceptable, cost effective interventions that can be implemented successfully in LMICs.

CENTRES

Christian Medical College, Vellore, India
MRC Unit at LSHTM, Fajara, The Gambia

DESIGN

The study is a 2 x 2 factorial design and eligible women will be randomized to either one of the arms – (i) yoghurt (ii) PA (iii) yoghurt + PA (iv) standard care

For peer review only - http://bmjopen.bmj.com/site/about/guidelines.xhtml

ELIGIBILITY CRITERIA

The study will include pregnant women aged ≥18 years and GA ≤16 weeks and meeting at least one risk-factor for GDM (booking BMI ≥25kg/m^2, age ≥25 years; a first-degree relative with diabetes; a previous pregnancy complicated by GDM, pre-eclampsia/eclampsia and/or a large baby (≥3.5kg); a history of PCOS/impaired fasting glucose) and not currently on any medication that is known to interfere with glucose metabolism.

Exclusion criteria

- GDM diagnosed prior to screening visit based on IADPSG criteria or documented raised HbA1C, i.e., either fasting glucose ≥5.1 mmol/L or 1h glucose ≥10.0 mmol/L or 2h glucose ≥8.5 mmol/L, or a documented HbA1c of ≥6.5% at first booking
- History of pre-gestational diabetes, severe hyperemesis in the first trimester
- History of recurrent (≥2) first trimester spontaneous abortions, stillbirth, significant ante- or post-partum haemorrhage in previous pregnancies
- Multiple gestation in the current pregnancy
- Uncontrolled pre-gestational or gestational hypertension (BP>150/100 mmHg) on treatment
- Previous child born with congenital anomalies
- Pregnancy following in-vitro fertilization or any assisted reproductive technology
- Previous or current psychiatric illness on medication, epileptic seizures or on anti-epileptic medication
- Women meeting absolute contraindications for physical activity during pregnancy as recommended by the ACOG (heart disease, restrictive lung disease, incompetent cervix/cerclage, pregnancies at risk for premature labour, gestational hypertension, severe anaemia)

METHODS

Women attending obstetric outpatient clinics of the Christian Medical College, Vellore, India and MRC Unit in The Gambia operating in four centres in The Gambia (Brikama Major Health Centre, Bundung Maternal and Child Hospital, Barfrow Medical Centre and Edward Francis Small Teaching Hospital) will be informed about the study and assessed for eligibility. Following informed consent, women enrolled in the study will be asked to attend five scheduled visits (screening, run-in phase, randomisation, visits 1, 2 and 3) as shown in **Figure 2** and detailed in the protocol **(online supplementary file 1)**. The study is expected to run from September 2018 to August 2021.

RANDOMISATION

Participants will be randomly allocated to one of the following arms (i) yoghurt (ii) PA (iii) yoghurt + PA or (iv) standard care following an allocation schedule pre-stratified for centre, age and BMI. A variable block randomisation, stratified by centre, and then by age, (<25 and ≥25 years) and BMI (<25 and ≥25 kg/m^2) will be generated, using a password-protected access database developed by an independent statistician not involved with trial participants. If a woman has more than one risk factor, she will be allocated to the strata depending on the first of the risk factors in the hierarchical block randomisation procedure (age or BMI). In India, the allocation will be performed using a computer interface (Access database), while

For peer review only - http://bmjopen.bmj.com/site/about/guidelines.xhtml

separate computer-generated randomisation lists for each strata combination (age X BMI) will be prepared for each health facility in the Gambia. Due to logistic limitations, this will be implemented using pre-issued opaque, sealed envelopes containing the allocation code.

INTERVENTIONS

Dietary intervention Yoghurt will be prepared locally at the dietary department in CMC, Vellore and at Kombo Dairy farm in The Gambia based on a common recipe and using the same starter culture containing *lactobacillus sp* and *Bifidobacterium sp* at 10^7 CFU. Yoghurt will be prepared in batches and an internal quality check will be performed to ensure that the bacterial species and colony count are maintained with each batch. Participants in the active yoghurt arm will consume 200 g/day of regular, unflavoured, fermented yoghurt throughout the active intervention period independent of the timing of their regular meal. Yoghurt will be dispensed either daily or weekly as feasible by the study centre and possibility of storage in refrigerator at participants' homes when not in use. Compliance with yoghurt will be assessed using pot count by field workers. All participants will receive dietary counselling, will not be advised to stop their regular dairy consumption, assuming that any effect on the outcome will be related to the trial yoghurt consumed. Information on background dairy consumption will be collected at each scheduled visit using a standardized DAIRY consumption questionnaire.

Physical activity intervention The PA activity intervention will include daily walking to increase the individual target step count, monitored using a wrist-mounted accelerometer device (Garmin Vivofit 4 fitness band).

The baseline step count will be recorded in all participants (blinding the step count on the device) for 7 days, with no emphasis on step count and/or activity during the run-in phase. At randomisation, baseline step count will be calculated as the average over 7 days including two weekend days. An individual target will be set, to a 40% or greater increase in daily step count compared with the baseline reading. The increment of 40% was based on a feasibility estimation. Systematic reviews have shown that simple pedometer interventions can lead to a sustained increase of around 2000-2500 steps a day in a great number of populations and settings.[61 62] The background step count in these populations was reasonably low (~5000), which will mean that 2000 additional step represent 40%.

The step count reading on the device will be unblinded after randomisation for women in the active PA arms, to provide visual feed back to the participants and to motivate them to achieve their daily target step count. Women in the non-PA arm will continue to use their fitness bands blinded throughout the study to measure their routine PA behaviour. Step count data will be periodically uploaded using Garmin application by the field worker to quantify compliance monitoring and counselling to participants in the PA arm. As the device is water resistant, the participants would be instructed to wear it day and night.

STUDY ASSESSMENT

Clinical measurements

Detailed study-related procedures, as outlined in **Table 1** and routine antenatal examination will be carried out at all study visits. All measurements will be standardized between the two

For peer review only - http://bmjopen.bmj.com/site/about/guidelines.xhtml

study centres and will be carried out as detailed in study specific standard operating procedures (SOPs).

Table 1. Study-related procedures at each study visit

**Procedures**	**Visits**						
	**Visit -2**	**Visit -1**	**Visit 0**	**Visit 1**	**Visit 2**	**Visit 3**	**Delivery**
	**Screening ≤16 weeks**	**Run-in-phase ≤17 weeks**	**Randomization Week ≤18**	**Week 18-20**	**Week 26-28**	**Week 32**	
Informed consent	X						
Eligibility assessment	X						
Demographics	X						
History	X						
Height	X						
Weight	X	X	X	X	X	X	X
Blood pressure	X	X	X	X	X	X	X
Antenatal examination	X	X	X	X	X	X	
Dairy consumption questionnaire		X	X	X	X	X	
Barriers questionnaire	X					X	
Baseline PA monitoring with Vivofit 4		X					
PA step-count monitoring			X	X	X	X	
Randomisation			X				
OGTT	X				X		
Dating scan	X						
Morphology scan				X			
Ultrasound scan (growth)						X	
Fasting plasma glucose						X	
Intrapartum assessment (mother)							X
New born assessment							X

For peer review only

Laboratory tests
Study participants will undergo a 75g OGTT at screening and visit 2 (week 26-28 GA) following an 8 hour overnight fast. Additional fasting plasma glucose will be measured at the end-of-study visit (week 32). Blood glucose measurements will be analysed using a Roche Cobas 800 autoanalyser at respective laboratories (Department of Clinical Biochemistry in Vellore, India and the MRCG at LSHTM, The Gambia). Other routine ante-natal laboratory tests, including haemoglobin will be done as a part of routine antenatal care according to local guidelines.

GDM ascertainment
IADPSG criteria will be used to diagnose GDM using an OGTT at week 26-28. GDM diagnosis will be established if either fasting glucose ≥5.1 mmol/L or 1h glucose ≥10.0 mmol/L or 2h glucose ≥8.5 mmol/L.

Questionnaire based assessments
Standardized questionnaires will be used to obtain information on social demographics, background dairy consumption and to identify barriers to lifestyle intervention during pregnancy in all participating women. The barriers questionnaire is specifically designed to address ethnic-specific social and cultural barriers to lifestyle intervention during pregnancy in both populations and would be administered at the start and the end of the study. The DAIRY consumption questionnaire used in this study is a modification the Dutch Dairy Food Frequency Questionnaire (Wageningen University, Department Human Nutrition, 2005)[63]. The questions relate to consumption of various dairy products divided into 6 different groups: total dairy, cheese, milk, nonfermented milk products, fermented milk, and other dairy products. Flavoured milk, puddings, and cream are all part of the nonfermented milk product group. The fermented milk products consist of yogurt, yogurt drinks, and butter milk. Additional modifications to include other dairy preparations that are locally consumed in both countries are included.

Ultrasound scans
Ultrasound scans will be performed by an ultrasound-trained obstetrician on Voluson GE S-8 (in India) and Aloka USI-145 (in Gambia) ultrasound machines on all participates at three visits, i) at screening (dating) ii) morphology scan at visit 1 (18-20 weeks GA) and iii) at visit 3 (week 32) for growth monitoring. Both centres will follow standard protocols as described in study standard operating procedures (SOPs). Evidence of foetal congenital anomaly, placental malformation or malposition or abnormal ultrasound features that can compromise pregnancy outcome will lead to termination of the participant's involvement in the study and referral for the normal care pathway at each participating centre.

OUTCOMES

Primary outcome
Incidence of GDM diagnosed using IADPSG criteria between 26-28 weeks of gestation or if the fasting plasma glucose concentration is ≥5.1 mmol/l at 32 weeks of gestation.

Secondary outcomes
Effect of the intervention on fasting blood glucose concentration at 32 weeks, gestational weight gain and blood pressure from randomisation to week 32, proportion of women

For peer review only - http://bmjopen.bmj.com/site/about/guidelines.xhtml

undergoing instrumental/caesarean delivery, proportion of women developing pre-eclampsia and eclampsia, blood loss during delivery (subjective assessment by the obstetrician), post-partum haemorrhage, pre-term births (<37 weeks GA), foetal macrosomia defined as birth weight >2 SDs above the population-specific mean in each setting, birth weight and length, APGAR score at 1 and 5 minutes and responses to questionnaire based assessments of barriers to lifestyle interventions in pregnancy.

ADVERSE (AE)/SERIOUS ADVERSE EVENTS (SAEs) AND REPORTING

All AE and SAEs will be captured in respective electronic CRFs. Details of all events including onset date, relationship to the study intervention, treatment given, and stop date will be captured in the respective AE report form. The PI will report all SAEs without filtration, whether or not related to the intervention, within 24 hours of becoming aware of the event, to the sponsor. All SAEs will be notified to the respective Ethics Committee within seven calendar days if fatal or life-threatening, and all others within 15 calendar days. All AEs and SAEs will also be notified to the Data Safety and Monitoring Committee (DSMC). Expected SAEs which are protocol-defined exceptions to SAE reporting will be captured and reported to respective ECs and DSMC as outlined in the protocol **(online supplementary file 1)**

STATISTICAL ANALYSIS PLAN INCLUDING SAMPLE SIZE AND POWER CALCULATION

Sample size

The sample size calculation was performed by simulation (in R version 3.5.2) using an algorithm as described in detail in the protocol (**online supplementary file 1**). After 10,000 repetitions, we estimated the sample size to be 1856 high-risk women (928 from each centre). The sample size is estimated to have 80% power to detect a 33% reduction in the incidence of GDM, for the main effects of diet and PA, with a family-wise significance level of 5% for a two-sided test (i.e., each effect was tested at a significance level of 2.5%). It was assumed that participants could be pooled across centres (no interaction between interventions and country at the 5% level). The sample size was inflated to allow for a 15% drop out rate in each of the four strata, with drop outs defined as non-compliant participants (i.e. no risk reduction in their incidence of GDM).

Data analysis

Data will be analysed using both (1) the intention-to-treat principle, including all enrolled participants regardless of their adherence to the proposed intervention, and (2) a per protocol approach, including only those participants adhering to at least 75% of the proposed intervention. In addition, we will run a sensitivity analysis to assess the effect of protocol deviation compared to 100% adherence.

Descriptive analyses at baseline

We will describe baseline characteristics of the four groups (Control, yoghurt, PA, and yoghurt + PA) using means and standard deviations (normally distributed quantitative variables), medians and interquartile ranges (non-normally distributed quantitative variables), or proportions (categorical variables).

For peer review only - http://bmjopen.bmj.com/site/about/guidelines.xhtml

Primary analysis

For our primary outcome (presence or absence of GDM), we will use multiple logistic regression models and results presented as OR and 95% CI. We will examine the assumption that there are no interaction effects between study centre and each of the interventions by fitting a multiple logistic regression model.

We will build two covariate adjusted models. First, we will assess the relative difference between centres in the effects of each intervention on GDM by adding a term for study centre. Second, we will fit a covariate (age, pre-pregnancy BMI, previous history of GDM and GA) adjusted model adding further terms for each, as well as relevant interaction terms, to remove bias from the estimate of the treatment effect on the primary outcome. Each covariate will be added to the unadjusted model separately first and then added one at a time and kept in the model if their significance level is <10%.

Secondary analyses

Continuous variables will be analysed using multiple linear regression and binary variables by multiple logistic regression. For each secondary outcome, we will fit a simple model with only terms for each intervention, and a covariate adjusted model including terms for study centre and the remaining pre-specified covariates and relevant interactions.

DATA COLLECTION

Research data will be collected using electronic CRFs (eCRFs) on tablets by designated study staff after training. Where there is internet connectivity, collected data will be stored directly onto the central server housed at MRC, Fajara and where there is no/unreliable connectivity, data will be stored locally on the tablet and later synchronized to the central servers. The accelerometer data used for PA monitoring will be directly synced with the Garmin app on the tablets on a weekly basis and transferred to eCRFs. To ensure standardisation of processes, SOPs with respect to trial management, quality assurance, data management, IT & security, and statistics will be adhered to.

COMMITTEE OVERSIGHTS

The study will be conducted in accordance with the approved protocol, Good Clinical Practice regulations and SOPs. The implementation of the study will be overseen by the Trial Steering committee (TSC). An independent DSMC, comprised of experts in clinical trials/epidemiology, biostatistics and obstetrics will meet 6-monthly to review the progress of the trial (recruitment, compliance, loss to follow-up), clinical study safety data, data quality and assess and/or adjudicate all events of deaths, the GDM event rate and key secondary/safety outcomes and provide feedback to the TSC. The DSMC will also meet if required, more frequently depending on the enrolment rate, safety issues and AEs. A copy of the full study protocol and all amendments would be submitted to DSMC prior to study initiation and subsequently. Thereafter, periodic reports including summary of enrolment, demographics, protocol compliance and safety will be compiled by the study statistician and submitted at least one week prior to the scheduled meeting. The board will advise on continuation or stopping the trial based upon safety and efficacy.

For peer review only - http://bmjopen.bmj.com/site/about/guidelines.xhtml

ETHICS AND DISSEMINATION

Ethical issues

The proposed interventions are considered generally safe both for maternal and foetal health. Participation in this trial is voluntary and subject to informed consent. A sample informed consent statement is available in the study protocol (**online supplementary file 1).** Participants who develop GDM at the visit 2 OGTT will be started on pharmacological therapy (as per local management guidelines) and results will be analysed as per intention to treat. Research participants can withdraw from the study at any time during the trial. The SPIRIT Checklist for the protocol is detailed in **online supplementary file 2.**

Dissemination

Results of the study will be presented at major national and international conferences, and publish in peer-reviewed journals, ensuring open access and the highest possible impact. The de-identified data will be openly available to the scientific community after publication of initial results. We will interact with national and international health agencies and governments to stimulate translation of our findings into policy and expect our research to influence policy for mothers and children within the next 5-10 years.

Patient and public involvement

The trial was informed by patients' need for effective preventive therapies for GDM since randomised trial data are lacking in these populations. Patients were not involved in the design, conduct or reporting of the study. A summary of the main trial results will be disseminated to the general public and study participants after the completion of the study.

DISCUSSION

GDM poses increased risks to maternal and child health. LMICs face numerous challenges to GDM care at various levels. These include (i) late second trimester screening for GDM, ii) lack of standardized nation-wide guidelines for diagnosis, iii) interventions targeting only women who are diagnosed with GDM around 24-28 weeks, iv) lack of knowledge and awareness of GDM and its health implications and v) sociocultural barriers to early antenatal care and life-style interventions during pregnancy. It seems very likely that early screening and establishment of positive lifestyle changes beginning from conception, or even prior to conception, particularly in selected high-risk women, and especially in low-resource settings, will reduce the burden of GDM as well as improve both immediate obstetric outcomes and long term health of the mother and the new born. Primary prevention of GDM, rather than treatment initiated after GDM onset is likely to lead to both economic and individual health benefits.

The PRIMORDIAL study is timely given the increasing prevalence of both diabetes and obesity in countries like Africa and India. Our study provides a unique opportunity to test simple life-style interventions during pregnancy in the LMIC settings, which have never been undertaken. The results will add to the existing evidence of beneficial effects of yoghurt on several pregnancy related outcomes.

The study is expected to bridge a gap in knowledge of the potential effectiveness of GDM prevention in two LMIC settings and create opportunities for capacity building in both India and The Gambia. We believe the trial can provide information that will aid in policy guidelines

For peer review only - http://bmjopen.bmj.com/site/about/guidelines.xhtml

relating to GDM screening and management and also suggest areas that may require further research.

Contributors SKV and FK conceived and designed the study. SKV, DT, CHDF, CO, SJU, AMP and FK drafted the original grant proposal and trial protocol. CO, FC and AJ provided methodological and data analysis plan. JM, SR, MJ, MB, and AMP provided expertise in the pregnancy clinical outcomes. DT, SJU, CHDF and AMP provided expertise in study interventions. FC and AJ will contribute significantly to the data management and central data monitoring. SKV drafted the original protocol. SKV, MJ, OJ, MB, JM, SR have responsibilities for day-to-day running of the trial including participant recruitment, data collection and liaising with other sites. SKV and FK drafted the first version of this manuscript. All authors critically reviewed and approved the final version of the manuscript. All authors agreed to be accountable for all aspects of the work.

Funding This study is supported by grants jointly from Medical Research Council, UK (MR/R020345/1), and Newton Fund- Director of Biotechnology, India (BT/IN/DBT-MRC/DIFD/JM/12/2018-19) The study is partly supported by the Global Challenges Research fund, University of Oxford, UK (0006138)

Disclaimer The views expressed in this publication are those of the author(s) and not necessarily those of the MRC, UK, GCRF, UK, DBT, India, healthcare systems or competent authorities.

Competing interests None declared.

Ethics approval This study will be conducted in accordance with the principles of Good Clinical Practice. The study is approved by Oxford Tropical Research Ethics Committee (OxTREC 44-18), ethics committees of Christian Medical College, Vellore (IRB 11367), Scientific co-ordinating committee, The Gambia (SCC 1645) and The Gambia Government/MRCG joint ethics committee (L2020.E15).

Provenance and peer review Not commissioned; externally peer reviewed.

REFERENCES

1. Cho NH, Shaw JE, Karuranga S, et al. IDF Diabetes Atlas: Global estimates of diabetes prevalence for 2017 and projections for 2045. *Diabetes Res Clin Pract* 2018;138:271-81. doi: 10.1016/j.diabres.2018.02.023
2. Buchanan TA, Xiang AH. Gestational diabetes mellitus. *J Clin Invest* 2005;115(3):485-91. doi: 10.1172/JCI24531 [published Online First: 2005/03/15]
3. Reece EA, Leguizamon G, Wiznitzer A. Gestational diabetes: the need for a common ground. *Lancet* 2009;373(9677):1789-97. doi: 10.1016/S0140-6736(09)60515-8
4. Yu Y, Arah OA, Liew Z, et al. Maternal diabetes during pregnancy and early onset of cardiovascular disease in offspring: population based cohort study with 40 years of follow-up. *BMJ* 2019;367:l6398. doi: 10.1136/bmj.l6398

For peer review only - http://bmjopen.bmj.com/site/about/guidelines.xhtml

5. Sacks DA, Hadden DR, Maresh M, et al. Frequency of gestational diabetes mellitus at collaborating centers based on IADPSG consensus panel-recommended criteria: the Hyperglycemia and Adverse Pregnancy Outcome (HAPO) Study. *Diabetes Care* 2012;35(3):526-8. doi: 10.2337/dc11-1641 [published Online First: 2012/02/23]
6. Wahi P, Dogra V, Jandial K, et al. Prevalence of gestational diabetes mellitus (GDM) and its outcomes in Jammu region. *J Assoc Physicians India* 2011;59:227-30.
7. Seshiah V, Balaji V, Balaji MS, et al. Pregnancy and diabetes scenario around the world: India. *Int J Gynaecol Obstet* 2009;104 Suppl 1:S35-8. doi: 10.1016/j.ijgo.2008.11.035
8. Macaulay S, Dunger DB, Norris SA. Gestational diabetes mellitus in Africa: a systematic review. *PLoS One* 2014;9(6):e97871. doi: 10.1371/journal.pone.0097871
9. Mwanri AW, Kinabo J, Ramaiya K, et al. Gestational diabetes mellitus in sub-Saharan Africa: systematic review and metaregression on prevalence and risk factors. *Trop Med Int Health* 2015;20(8):983-1002. doi: 10.1111/tmi.12521
10. Jiwani A, Marseille E, Lohse N, et al. Gestational diabetes mellitus: results from a survey of country prevalence and practices. *J Matern Fetal Neonatal Med* 2012;25(6):600-10. doi: 10.3109/14767058.2011.587921
11. Zhu Y, Zhang C. Prevalence of Gestational Diabetes and Risk of Progression to Type 2 Diabetes: a Global Perspective. *Curr Diab Rep* 2016;16(1):7. doi: 10.1007/s11892-015-0699-x
12. Catalano PM, Tyzbir ED, Roman NM, et al. Longitudinal changes in insulin release and insulin resistance in nonobese pregnant women. *Am J Obstet Gynecol* 1991;165(6 Pt 1):1667-72. doi: 10.1016/0002-9378(91)90012-g
13. Catalano PM. Obesity, insulin resistance, and pregnancy outcome. *Reproduction* 2010;140(3):365-71. doi: 10.1530/REP-10-0088
14. Benhalima K, Lens K, Bosteels J, et al. The Risk for Glucose Intolerance after Gestational Diabetes Mellitus since the Introduction of the IADPSG Criteria: A Systematic Review and Meta-Analysis. *J Clin Med* 2019;8(9) doi: 10.3390/jcm8091431
15. Ferrara A. Increasing prevalence of gestational diabetes mellitus: a public health perspective. *Diabetes Care* 2007;30 Suppl 2:S141-6. doi: 10.2337/dc07-s206
16. Group HSCR, Metzger BE, Lowe LP, et al. Hyperglycemia and adverse pregnancy outcomes. *N Engl J Med* 2008;358(19):1991-2002. doi: 10.1056/NEJMoa0707943
17. Lowe LP, Metzger BE, Dyer AR, et al. Hyperglycemia and Adverse Pregnancy Outcome (HAPO) Study: associations of maternal A1C and glucose with pregnancy outcomes. *Diabetes Care* 2012;35(3):574-80. doi: 10.2337/dc11-1687
18. International Association of D, Pregnancy Study Groups Consensus P, Metzger BE, et al. International association of diabetes and pregnancy study groups recommendations on the diagnosis and classification of hyperglycemia in pregnancy. *Diabetes Care* 2010;33(3):676-82. doi: 10.2337/dc09-1848
19. Berger H, Sermer M. Counterpoint: Selective screening for gestational diabetes mellitus. *Diabetes Care* 2009;32(7):1352-4. doi: 10.2337/dc09-0361
20. Ostlund I, Hanson U. Occurrence of gestational diabetes mellitus and the value of different screening indicators for the oral glucose tolerance test. *Acta Obstet Gynecol Scand* 2003;82(2):103-8. doi: 10.1034/j.1600-0412.2003.00001.x
21. Guo XY, Shu J, Fu XH, et al. Improving the effectiveness of lifestyle interventions for gestational diabetes prevention: a meta-analysis and meta-regression. *BJOG* 2019;126(3):311-20. doi: 10.1111/1471-0528.15467

For peer review only

22. Agha-Jaffar R, Oliver N, Johnston D, et al. Gestational diabetes mellitus: does an effective prevention strategy exist? *Nat Rev Endocrinol* 2016;12(9):533-46. doi: 10.1038/nrendo.2016.88
23. Thangaratinam S, Rogozinska E, Jolly K, et al. Effects of interventions in pregnancy on maternal weight and obstetric outcomes: meta-analysis of randomised evidence. *BMJ* 2012;344:e2088. doi: 10.1136/bmj.e2088
24. Aune D, Norat T, Romundstad P, et al. Dairy products and the risk of type 2 diabetes: a systematic review and dose-response meta-analysis of cohort studies. *Am J Clin Nutr* 2013;98(4):1066-83. doi: 10.3945/ajcn.113.059030
25. Elwood PC, Pickering JE, Givens DI, et al. The consumption of milk and dairy foods and the incidence of vascular disease and diabetes: an overview of the evidence. *Lipids* 2010;45(10):925-39. doi: 10.1007/s11745-010-3412-5
26. Grantham NM, Magliano DJ, Hodge A, et al. The association between dairy food intake and the incidence of diabetes in Australia: the Australian Diabetes Obesity and Lifestyle Study (AusDiab). *Public Health Nutr* 2013;16(2):339-45. doi: 10.1017/S1368980012001310
27. Tong X, Dong JY, Wu ZW, et al. Dairy consumption and risk of type 2 diabetes mellitus: a meta-analysis of cohort studies. *Eur J Clin Nutr* 2011;65(9):1027-31. doi: 10.1038/ejcn.2011.62 [published Online First: 2011/05/12]
28. Veiga P, Pons N, Agrawal A, et al. Changes of the human gut microbiome induced by a fermented milk product. *Sci Rep* 2014;4:6328. doi: 10.1038/srep06328
29. Hill C, Guarner F, Reid G, et al. Expert consensus document. The International Scientific Association for Probiotics and Prebiotics consensus statement on the scope and appropriate use of the term probiotic. *Nat Rev Gastroenterol Hepatol* 2014;11(8):506-14. doi: 10.1038/nrgastro.2014.66
30. Wen L, Duffy A. Factors Influencing the Gut Microbiota, Inflammation, and Type 2 Diabetes. *J Nutr* 2017;147(7):1468S-75S. doi: 10.3945/jn.116.240754
31. Gomez-Arango LF, Barrett HL, McIntyre HD, et al. Connections Between the Gut Microbiome and Metabolic Hormones in Early Pregnancy in Overweight and Obese Women. *Diabetes* 2016;65(8):2214-23. doi: 10.2337/db16-0278
32. Crusell MKW, Hansen TH, Nielsen T, et al. Gestational diabetes is associated with change in the gut microbiota composition in third trimester of pregnancy and postpartum. *Microbiome* 2018;6(1):89. doi: 10.1186/s40168-018-0472-x
33. Fox C, Eichelberger K. Maternal microbiome and pregnancy outcomes. *Fertil Steril* 2015;104(6):1358-63. doi: 10.1016/j.fertnstert.2015.09.037
34. Ruan Y, Sun J, He J, et al. Effect of Probiotics on Glycemic Control: A Systematic Review and Meta-Analysis of Randomized, Controlled Trials. *PLoS One* 2015;10(7):e0132121. doi: 10.1371/journal.pone.0132121
35. Gohir W, Ratcliffe EM, Sloboda DM. Of the bugs that shape us: maternal obesity, the gut microbiome, and long-term disease risk. *Pediatr Res* 2015;77(1-2):196-204. doi: 10.1038/pr.2014.169
36. Lindsay KL, Walsh CA, Brennan L, et al. Probiotics in pregnancy and maternal outcomes: a systematic review. *J Matern Fetal Neonatal Med* 2013;26(8):772-8. doi: 10.3109/14767058.2012.755166
37. Chen X, Jiang X, Huang X, et al. Association between Probiotic Yogurt Intake and Gestational Diabetes Mellitus: A Case-Control Study. *Iran J Public Health* 2019;48(7):1248-56.

For peer review only - http://bmjopen.bmj.com/site/about/guidelines.xhtml

38. Luoto R, Laitinen K, Nermes M, et al. Impact of maternal probiotic-supplemented dietary counselling on pregnancy outcome and prenatal and postnatal growth: a double-blind, placebo-controlled study. *Br J Nutr* 2010;103(12):1792-9. doi: 10.1017/S0007114509993898
39. Laitinen K, Poussa T, Isolauri E, et al. Probiotics and dietary counselling contribute to glucose regulation during and after pregnancy: a randomised controlled trial. *Br J Nutr* 2009;101(11):1679-87. doi: 10.1017/S0007114508111461
40. Ilmonen J, Isolauri E, Poussa T, et al. Impact of dietary counselling and probiotic intervention on maternal anthropometric measurements during and after pregnancy: a randomized placebo-controlled trial. *Clin Nutr* 2011;30(2):156-64. doi: 10.1016/j.clnu.2010.09.009
41. Sahhaf Ebrahimi F, Homayouni Rad A, Mosen M, et al. Effect of L. acidophilus and B. lactis on blood glucose in women with gestational diabetes mellitus: a randomized placebo-controlled trial. *Diabetol Metab Syndr* 2019;11:75. doi: 10.1186/s13098-019-0471-5
42. Asgharian H, Homayouni-Rad A, Mirghafourvand M, et al. Effect of probiotic yoghurt on plasma glucose in overweight and obese pregnant women: a randomized controlled clinical trial. *Eur J Nutr* 2019 doi: 10.1007/s00394-019-01900-1
43. Asemi Z, Samimi M, Tabassi Z, et al. Effect of daily consumption of probiotic yoghurt on insulin resistance in pregnant women: a randomized controlled trial. *Eur J Clin Nutr* 2013;67(1):71-4. doi: 10.1038/ejcn.2012.189
44. Kennedy JW, Hirshman MF, Gervino EV, et al. Acute exercise induces GLUT4 translocation in skeletal muscle of normal human subjects and subjects with type 2 diabetes. *Diabetes* 1999;48(5):1192-7. doi: 10.2337/diabetes.48.5.1192
45. Perseghin G, Price TB, Petersen KF, et al. Increased glucose transport-phosphorylation and muscle glycogen synthesis after exercise training in insulin-resistant subjects. *N Engl J Med* 1996;335(18):1357-62. doi: 10.1056/NEJM199610313351804
46. Artal R, O'Toole M. Guidelines of the American College of Obstetricians and Gynecologists for exercise during pregnancy and the postpartum period. *Br J Sports Med* 2003;37(1):6-12; discussion 12. doi: 10.1136/bjsm.37.1.6
47. Aune D, Sen A, Henriksen T, et al. Physical activity and the risk of gestational diabetes mellitus: a systematic review and dose-response meta-analysis of epidemiological studies. *Eur J Epidemiol* 2016;31(10):967-97. doi: 10.1007/s10654-016-0176-0
48. Clapp JF, 3rd, Lopez B, Harcar-Sevcik R. Neonatal behavioral profile of the offspring of women who continued to exercise regularly throughout pregnancy. *Am J Obstet Gynecol* 1999;180(1 Pt 1):91-4. doi: 10.1016/s0002-9378(99)70155-9
49. Nasiri-Amiri F, Sepidarkish M, Shirvani MA, et al. The effect of exercise on the prevention of gestational diabetes in obese and overweight pregnant women: a systematic review and meta-analysis. *Diabetol Metab Syndr* 2019;11:72. doi: 10.1186/s13098-019-0470-6
50. Oostdam N, van Poppel MN, Wouters MG, et al. No effect of the FitFor2 exercise programme on blood glucose, insulin sensitivity, and birthweight in pregnant women who were overweight and at risk for gestational diabetes: results of a randomised controlled trial. *BJOG* 2012;119(9):1098-107. doi: 10.1111/j.1471-0528.2012.03366.x
51. Han S, Middleton P, Crowther CA. Exercise for pregnant women for preventing gestational diabetes mellitus. *Cochrane Database Syst Rev* 2012(7):CD009021. doi: 10.1002/14651858.CD009021.pub2

For peer review only - http://bmjopen.bmj.com/site/about/guidelines.xhtml

52. Stafne SN, Salvesen KA, Romundstad PR, et al. Regular exercise during pregnancy to prevent gestational diabetes: a randomized controlled trial. *Obstet Gynecol* 2012;119(1):29-36. doi: 10.1097/AOG.0b013e3182393f86
53. Vinter CA, Tanvig MH, Christensen MH, et al. Lifestyle Intervention in Danish Obese Pregnant Women With Early Gestational Diabetes Mellitus According to WHO 2013 Criteria Does Not Change Pregnancy Outcomes: Results From the LiP (Lifestyle in Pregnancy) Study. *Diabetes Care* 2018;41(10):2079-85. doi: 10.2337/dc18-0808
54. Simmons D, Jelsma JG, Galjaard S, et al. Results From a European Multicenter Randomized Trial of Physical Activity and/or Healthy Eating to Reduce the Risk of Gestational Diabetes Mellitus: The DALI Lifestyle Pilot. *Diabetes Care* 2015;38(9):1650-6. doi: 10.2337/dc15-0360
55. Renault KM, Norgaard K, Nilas L, et al. The Treatment of Obese Pregnant Women (TOP) study: a randomized controlled trial of the effect of physical activity intervention assessed by pedometer with or without dietary intervention in obese pregnant women. *Am J Obstet Gynecol* 2014;210(2):134 e1-9. doi: 10.1016/j.ajog.2013.09.029
56. Asbee SM, Jenkins TR, Butler JR, et al. Preventing excessive weight gain during pregnancy through dietary and lifestyle counseling: a randomized controlled trial. *Obstet Gynecol* 2009;113(2 Pt 1):305-12. doi: 10.1097/AOG.0b013e318195baef
57. International Weight Management in Pregnancy Collaborative G. Effect of diet and physical activity based interventions in pregnancy on gestational weight gain and pregnancy outcomes: meta-analysis of individual participant data from randomised trials. *BMJ* 2017;358:j3119. doi: 10.1136/bmj.j3119
58. Poston L, Bell R, Croker H, et al. Effect of a behavioural intervention in obese pregnant women (the UPBEAT study): a multicentre, randomised controlled trial. *Lancet Diabetes Endocrinol* 2015;3(10):767-77. doi: 10.1016/S2213-8587(15)00227-2
59. Koivusalo SB, Rono K, Klemetti MM, et al. Gestational Diabetes Mellitus Can Be Prevented by Lifestyle Intervention: The Finnish Gestational Diabetes Prevention Study (RADIEL): A Randomized Controlled Trial. *Diabetes Care* 2016;39(1):24-30. doi: 10.2337/dc15-0511
60. Shepherd E, Gomersall JC, Tieu J, et al. Combined diet and exercise interventions for preventing gestational diabetes mellitus. *Cochrane Database Syst Rev* 2017;11:CD010443. doi: 10.1002/14651858.CD010443.pub3
61. Bravata DM, Smith-Spangler C, Sundaram V, et al. Using pedometers to increase physical activity and improve health: a systematic review. *JAMA* 2007;298(19):2296-304. doi: 10.1001/jama.298.19.2296
62. Mansi S, Milosavljevic S, Baxter GD, et al. A systematic review of studies using pedometers as an intervention for musculoskeletal diseases. *BMC Musculoskelet Disord* 2014;15:231. doi: 10.1186/1471-2474-15-231
63. Vissers PA, Streppel MT, Feskens EJ, et al. The contribution of dairy products to micronutrient intake in the Netherlands. *J Am Coll Nutr* 2011;30(5 Suppl 1):415S-21S. doi: 10.1080/07315724.2011.10719985

For peer review only - http://bmjopen.bmj.com/site/about/guidelines.xhtml

Figure Legends

Figure 1

Relationship between pancreatic beta cell plasticity with advancement in gestational age

Figure 2

Flow chart of the study recruitment and scheduled visits

For peer review only

For peer review only - http://bmjopen.bmj.com/site/about/guidelines.xhtml

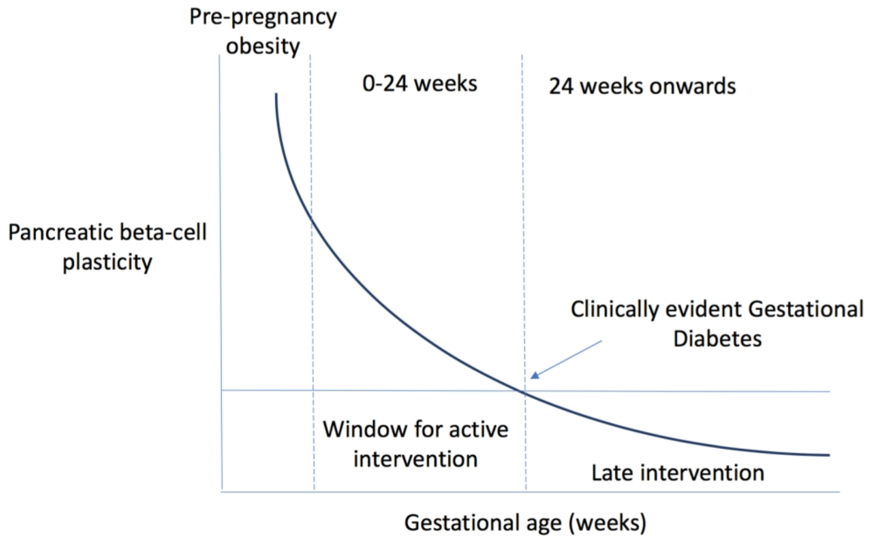

Figure 1. Relationship between pancreatic beta cell plasticity with advancement in gestational age

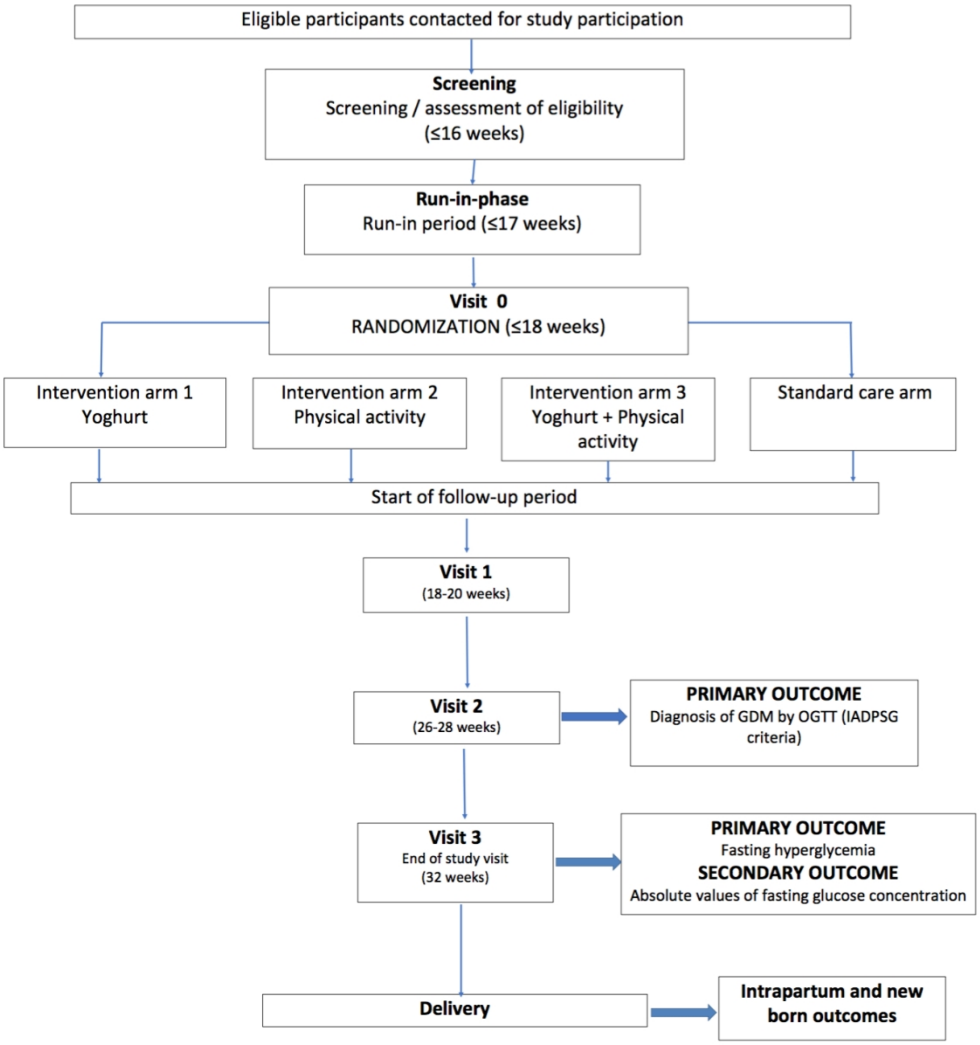

Figure 2. Flowchart of study recruitment and scheduled visits

Study Title: Pregnancy Related Interventions in Mothers at Risk for gestational Diabetes in Asian India and Low- and middle income countries

Internal Reference Number / Short title: PRIMORDIAL Study

OxTREC Ref: 44-18

Date and Version No: 18th July 2019. Version 4.2

Principal Investigator: Fredrik Karpe
Oxford Centre for Diabetes, Endocrinology and Metabolism (OCDEM)
University of Oxford
Churchill Hospital, Oxford OX3 7LE, UK
Phone: 0044 (0)1865 857222

Investigators: Andrew Prentice
MRC Unit The Gambia &
MRC International Nutrition Group,
London School of Hygiene & Tropical Medicine,
Keppel Street, London, WC1E 7HT, UK.

Jiji Elizabeth Matthews
Department of Obstetrics & Gynaecology – Unit 5
Christian Medical College & Hospital, Vellore -632004
Tamil Nadu, India

Sponsor: University of Oxford
Funder: Medical Research Council, UK

Principal Investigator Signature:

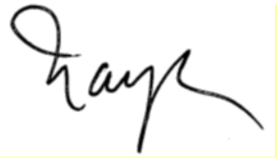

Trial Registration: ISRCTN: 18467720 and Clinical Trials Registry, India (CTRI/2018/07/014947)

Protocol written by: Senthil K Vasan and Fredrik Karpe, OCDEM, University of Oxford Churchill Hospital, Oxford OX3 7LE, UK

Conflicts of interest: None

Confidentiality Statement

This document contains confidential information that must not be disclosed to anyone other than the authorised individuals from the University of Oxford, the Investigator Team and members of the Oxford Tropical Research Ethics Committee (OxTREC), unless authorised to do so.

For peer review only

TABLE OF CONTENTS

1. SYNOPSIS 5
2. ABBREVIATIONS 7
3. BACKGROUND AND RATIONALE 9
4. OUTCOME MEASURES 17
5. STUDY DESIGN 18
6. PARTICIPANT IDENTIFICATION AND RECRUITMENT 20
6.1. Study Participants 20
6.2. Inclusion Criteria 21
6.3. Exclusion Criteria 21
7. STUDY PROCEDURES 21
7.1. Recruitment 22
7.2. Informed Consent 23
7.3. Screening and Eligibility Assessment: 23
7.4. Randomisation 24
7.5. Baseline Assessments 24
7.6. Subsequent Visits 26
7.6.1. Visit 1 (week 18-20) 26
7.6.2. Visit 2 (week 26-28) 26
7.6.3. Visit 3 (Week 32) - END OF STUDY VISIT 27
7.6.4. Post-intervention surveillance visit 27
7.7. Sample Handling 28
7.8. Discontinuation/Withdrawal 28
7.9. Lost to follow-up 30
7.10. Protocol deviation 30
7.11. Definition of End of Study 30
8. INTERVENTIONS 30
9. SAFETY REPORTING 32
9.1. Adverse event reporting 32
9.2. Definition of Serious Adverse Events 32
9.3. Reporting Procedures for Serious Adverse Events 33
10. STATISTICS AND ANALYSIS 33
10.1. Description of Statistical Methods 33
10.2. The Number of Participants 34

For peer review only

10.3. Analysis of Outcome Measures 35
11. DATA MANAGEMENT 36
11.1. Types of data 36
11.2. Format and scale of data 36
11.3. Data collection/generation 37
11.4. Data quality and standards 37
11.5. Managing, storing and curating data 37
11.6. Data preservation strategy and standards 38
11.7. Main risks to data security 38
11.8. Suitability for sharing 38
11.9. Access to Data 38
11.10. Data Handling and Record Keeping 39
12. COMMITTEES STRUCTURES 39
12.1. DATA SAFETY MONITORING COMMITTEE (DSMC) 39
12.2. Trial Steering committee (TSC) 40
12.3. Executive committee 41
13. ETHICAL AND REGULATORY CONSIDERATIONS 41
13.1. ICH-GCP 41
13.2. Approvals 42
13.3. Participant Confidentiality 42
13.4. Expenses and Benefits 42
13.5. Annual Progress Report 42
13.6. Other Ethical Considerations 42
14. FINANCE AND INSURANCE 42
14.1. Funding 42
14.2. Insurance 43
15. PUBLICATION POLICY 43
16. REFERENCES 44
18. APPENDIX A: STUDY FLOW CHART 47
19. APPENDIX B: STUDY TIMELINES (approximate) 48
20. APPENDIX C: SCHEDULE OF STUDY PROCEDURES 49
21. APPENDIX D: PATIENT INFORMATION SHEET 50
22. APPENDIX E: INFORMED CONSENT FORM 53
23. APPENDIX F: AMENDMENT HISTORY 54

For peer review only

For peer review only

1. SYNOPSIS

**Long Study Title**	**Pr**egnancy **I**nterventions **I**n **M**others **R**elating to **D**iabetes In **A**sian India and **L**ow-income countries	
**Short Study Title (to be used on participant-facing documents, if applicable)**	PRIMORDIAL Study	
**Nature of Study Participants**	Pregnant women with high-risk for developing gestational diabetes	
**Intended number of participants**	1,875 high-risk women (~937 from each centre)	
**Planned Study Period**	36 months	
	**Objectives**	**Outcome Measures**
**Primary**	To determine the efficacy of life-style interventions (yoghurt consumption and physical actvity), given to pregnant women at high risk of GDM , in reducing the incidence of GDM.	Women will be diagnosed to have GDM if one or more plasma glucose concentrations following a 75g OGTT equals or exceeds the following: fasting glucose ≥5.1 mmol/l (92 mg/dl) and/or 1h glucose ≥10.0 mmol/l (180 mg/dl) and/or 2h glucose ≥8.5 mmol/l (153 mg/dl) at 26-28 weeks gestation or if the fasting plasma glucose concentration is ≥5.1 mmol/l at 32 weeks of gestation
**Secondary**	To determine the effect of life-style intervention (yoghurt and PA), given to women at high risk of GDM , on fasting blood glucose concentrations..	Absolute concentration of fasting blood glucose at 26-28 weeks and/or at 32 weeks of gestation.
Trial and post-trial surveillance	(i) To determine the safety and efficacy of life-style intervention (yoghurt and PA), given to women at high risk of GDM, on maternal weight gain and blood pressure during pregnancy<br><br>(ii) To determine the safety and efficacy of life-style	serial weight and blood pressure measurements at all scheduled visits<br><br>**Maternal outcomes**<br>• Proportion of women undergoing instrumental/caesarean

For peer review only

	intervention (yoghurt and PA) on adverse maternal and new-born outcomes.	delivery (elective and emergency) captured on delivery records by treating obstetrician<br>• Post-partum haemorrhage (Primary) as captured on delivery records by treating obstetrician<br>• Pre-eclampsia and eclampsia based on blood pressure recording at each visit<br>• Blood loss at delivery as captured on delivery records by treating obstetrician<br><br>**New born outcomes**<br>• Pre-term births (<37 weeks of gestational age) calculated from the ultrasound derived gestational age captured at screening visit dating scan.<br>• Foetal macrosomia (defined as birth weight >2 SDs above the population-specific mean in each setting)<br>• Birth weight and length<br>• APGAR score at 1 and 5min of after birth
	(iii) To determine social and cultural barriers to life-style changes during pregnancy	Questionnaire based assessment of social and cultural barriers.

For peer review only

2. ABBREVIATIONS

AC	Abdominal circumference
ACOG	American College of Obstetrics and Gynaecology
AE	Adverse event
AFVI	Amniotic Fluid Volume Index
BMI	Body Mass Index
BPD	Biparietal diameter
BW	Birth weight
CRF	Case Report Form
CRL	Crown-rump length
CTRG	Clinical Trials & Research Governance, University of Oxford, UK
CI	Confidence Interval
CCM	Cultural Consensus Modelling
CFU	Colony forming units
CMC	Christian Medical College, Vellore, India
CTRI	Clinical Trials Registry, India
CUREC	Central University Research Ethics Committee
CVD	Cardiovascular disease
DMC	Data Monitoring Committee
FFQ	Food Frequency Questionnaire
GA	Gestational age
GCP	Good Clinical Practice
GP	General Practitioner
GDM	Gestational Diabetes Mellitus
GWG	Gestational weight gain
HC	Head circumference
IADPSG	International Association of the Diabetes and Pregnancy Study Groups
ICF	Informed Consent Form
IDEA	Impact of Diet and Exercise Activity on Pregnancy Outcomes study
IR	Insulin Resistance
ISRCTN	International Standard Randomised Controlled Trial Number
IUGR	Intrauterine growth restriction
LGA	Large for gestational age

For peer review only

LIMIT	Antenatal lifestyle advices to improve pregnancy outcomes study
LiP	Lifestyle interventions in Pregnancy study
LMICs	Low and Middle Income Countries
LSHTM	London School of Hygiene and Tropical Medicine, UK
MRCG	Medical Research Council, The Gambia at LSHTM
MRC, UK	Medical Research Council, United Kingdom
MS IDREC	Medical Sciences Interdivisional Research Ethics Committee, Oxford, UK
OGTT	Oral Glucose Tolerance Test
PA	Physical Activity
PAMELA	Physical activity during pregnancy and maternal-child health study
PCOD	Polycystic ovarian disease
PI	Principal Investigator
PIS	Participant Information Sheet
PID	Personally identifiable data
PI_x	Pulsatility Index
PPAQ	Pregnancy-related Physical Activity Questionnaire
PRIMORDIAL	Pregnancy Interventions In Mothers Relating to Diabetes In Asian India and Low-income countries
RCT	Randomized Controlled Trial
REC	Research Ethics Committee
RR	Risk ratio
SD	Standard deviation
SOP	Standard Operating Procedure
SAE	Serious Adverse Event
SGA	Small for Gestational Age
S/D ratio	Systolic/diastolic ratio
TOP	Treatment of Obese Pregnant Women study
T2D	Type 2 Diabetes Mellitus
UPBEAT	UK Pregnancies Better Eating and Activity Trial
WHO	World Health Organization

For peer review only

3. BACKGROUND AND RATIONALE

Gestational diabetes mellitus (GDM) is associated with significant intrapartum complications, perinatal morbidity, and long-term risk of developing T2D, obesity and CVD in both the mother and the offspring (1, 2). Normally, hyperglycaemia rapidly abates following delivery. However, about 50-70% of GDM mothers develop T2D within 5-10 years postpartum (3) suggesting that GDM may be a prodrome of “common T2D”. The key underlying pathophysiology is the loss of β-cell plasticity to adapt to the progressive insulin resistance (IR). GDM risk can be reduced at two levels: i) by reducing IR secondary to obesity and ii) by improving glucose disposal. Recently, alterations in the gut microbiota have been linked to obesity, IR and T2D (4, 5). The gut microbiota composition changes over the course of gestation (6) and this is further exacerbated on the background of maternal IR and adiposity. Alteration of gut microbial composition through dietary changes and physical activity exert beneficial effects in terms of weight gain, blood glucose levels, reduced IR and T2D risk (7, 8). Thus, simple interventions that aim at altering the gut microbiota by life-style modification [diet and physical activity (PA)] and thus improving glucose disposal constitute potentially attractive options to reduce the risk of GDM.

Global burden of GDM GDM affects 1-14% of all pregnancies worldwide (9) and the risk of GDM reflects the underlying T2D frequency in the community (10). The prevalence is increased to 17.8% (range 9.3**–**25.5%) with the recent introduction of IADPSG criteria (11). The risk of GDM in specific ethnic groups particularly Asians and Africans is increased compared with Caucasians and thus contributes substantially to future T2D in these communities (12, 13). The incidence of GDM in LMICs is largely unknown and is mostly based on single-centre based statistics. In India, the reported prevalence varies between 17-19% (14). A recent systematic review of GDM prevalence in six African countries (mostly based on 2-step Carpenter Coustan’s or WHO criteria), report a prevalence equating to 11% (15). This may not be a true representation of the African continent and there is a relative absence of reliable prevalence estimates of GDM in West Africa.

Window for intervention ‘High-risk’ GDM mothers include pregnant women with chronic IR states such as overweight or obesity, rapid gestational weight gain (GWG), history of a polycystic ovarian disease (PCOD), impaired glucose tolerance prior to pregnancy, previous GDM and advanced maternal age. Other recognised risk factors include having a first-degree relative with T2D, previous history of stillbirth/miscarriage, a large baby or pre-eclampsia. Intense glycaemic management is usually reserved for mothers diagnosed with GDM, typically late in pregnancy (24-26 weeks), when glucose intolerance is apparent and almost 60% of β-cell plasticity is lost (16) (Figure 1). The “critical window” for any intervention should therefore ideally start earlier than 24-26 weeks when the pancreatic β-cells are still able to compensate with the maternal IR. The RADIEL study showed a 39% risk reduction in GDM incidence among high-risk pregnant women with diet and

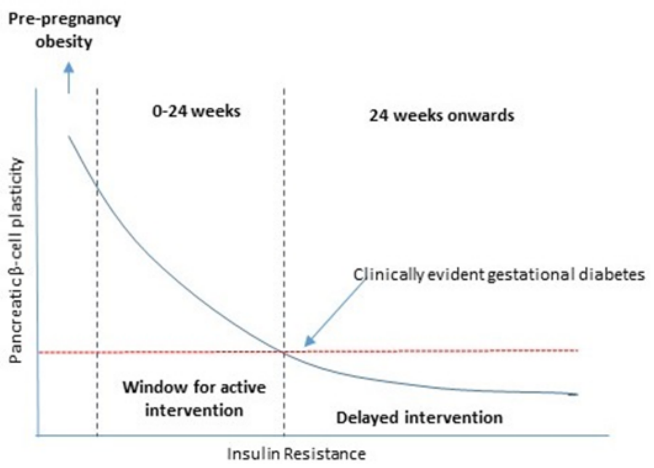

For peer review only - http://bmjopen.bmj.com/site/about/guidelines.xhtml

lifestyle intervention beginning from 20 weeks of gestation (17).

Dietary interventions Fermented dairy products (yoghurt and cultured sour milk) can alter the 'microbiotal-signature' and lower the gut-induced pro-inflammatory response (18). Recent meta-analyses have shown that daily yogurt consumption reduced risk of T2D by 14-18% compared to other dairy products (19, 20). Meta-analysis of probiotic use in late pregnancy has been shown to be safe (41). A summary of studies evaluating the beneficial effects of yoghurt in T2D prevention is shown in page 12.

Physical activity (PA) in pregnancy PA has well-documented health benefits and reduces peripheral insulin resistance in non-pregnant individuals. It is likely that the mechanisms responsible for alterations in insulin sensitivity and glucose uptake in response to exercise in non-pregnant populations may apply to pregnancy. PA is considered safe during pregnancy and substantially reduces the incidence of GDM by reducing IR (21, 22). RCTs and meta-analyses have shown benefits in terms of GWG, delivery type, preterm delivery rate and neonatal outcomes (23, 24). Interventional studies that used aerobic exercises or strength training initiated as early as 9-12 weeks and continued until 38 weeks have clearly shown benefits on glycaemic control, GWG and pregnancy outcomes (25, 26). The ACOG recommend 20-30 minutes of moderate-intensity aerobic activity or strength training at least 3-5 days/week during pregnancy (27). PA has also been shown to alter gut microbiota (28) and a differential microbiota pattern is observed between active vs. sedentary women, implying the role of PA in mediating glucose homeostasis by alteration of gut microbiota.

Hypothesis

Daily yoghurt and/or daily walking for at least 14 weeks will reduce the risk of developing gestational diabetes in 'high-risk' pregnant women

Benefits for the study population and the population whom the results of the project might be generalised to

Benefits to the participants: The current study will sensitise and expose women to specific healthy lifestyles during pregnancy. The opportunity to bring about behavioural changes in the early pregnant woman by changing maternal nutrition and increasing physical activity to prevent GDM can have immediate and lifelong health impact on the mother as well as the new born with possible positive consequences into adulthood. Lifestyle interventions during pregnancy are proven to have beneficial effects on GWG and improved neonatal outcomes. By demonstrating that maternal lifestyle changes beginning from early pregnancy using simple, realistic and culturally acceptable methods will have a beneficial impact in immediate and long term maternal and child health could potentially help reduce the overwhelming global burden of non-communicable diseases, particularly diabetes.

Benefits to the community: Our study aims at providing evidence to support political action and policy making at national and global level in low and middle-income countries (LMICs). This research aims at strengthening the existing antenatal services in The Gambia and the research facilities in India, where such resources are clearly lacking. In line with the research call, the

For peer review only

study will enable opportunities for capacity building in both India and the Gambia. The project will also create opportunities to specifically train young Gambian health providers (obstetricians, midwives, diabetes educators) to travel to India (which is a tertiary medical care facility and well known for its teaching excellence) to be trained in various aspects of antenatal care and high-risk management. In turn, the health researchers in India will clearly benefit from the support of MRC-Gambia in infrastructural set up of a data management facility.

For peer review only

Summary of findings from previous studies

Studies investigating the effect of consuming conventional or probiotic yoghurt during pregnancy

Study reference	Type of study	Target population	Intervention	Findings
Lindsay et al. 2013 (Ref 29)	Systematic review	7 studies including 3 clinical trials using probiotics in pregnancy	-	Impact of probiotic intervention on GDM is better among high-risk mothers
Laitinen et al. 2009 (Ref 30)	Double-blind RCT	Pregnant women <17 weeks (n=256)	Diet + Probiotic<br>Diet + Placebo<br>Control + placebo	Reduced risk of elevated glucose in probiotic group (OR 0.31, 95%CI 0.12-0.18; p=0.013)
Luoto et al. 2010 (Ref 31)	Double-blind RCT	Pregnant women <17 weeks (n=256)	Diet + Probiotic<br>Diet + Placebo<br>Control + placebo	Reduction in GDM risk by 13% in probiotic group
Zheng et al. 2018 (Ref 32)	Meta-analysis	Studies investigating probiotics in pregnancy		Probiotic supplementation during pregnancy is negatively associated with fasting insulin (OR -2.94, 95%CI [-5.69, -0.20], p = 0.04) and HOMA-IR (OR -0.65, 95%CI [-1.18, -0.11], p = 0.02)
Hajifaraji et al. 2018 (Ref 33)	Double-blind RCT	Pregnant women with GDM (n=64)	Probiotic vs placebo for 8 weeks	Improved inflammatory and oxidative stress biomarkers in probiotic group
Nilert et al. 2013 (SPRING Trial) (Ref 34)	Double-blind RCT	Pregnant women <16 weeks with BMI >25kg/m^2 (n=540)	Probiotic vs placebo capsules from 16 weeks to delivery	Ongoing

For peer review only - http://bmjopen.bmj.com/site/about/guidelines.xhtml
© Copyright: The University of Oxford 2019

Studies investigating the effect of PA during pregnancy

Study reference	Type of study	Target population	Intervention	Findings
Russo et al. 2015 (Ref 21)	Meta-analysis	10/469 studies (Total 3,401 GDM/high risk pregnant mothers, control healthy pregnant women)	-	The PA intervention arm showed a 28% risk reduction with GDM incidence compared with standard care arm.
Barakat et al. 2013 (Ref 35)	RCT	510 healthy gravida to either an exercise intervention or a usual care (control) group (n=255 each)	Moderate-intensity resistance and aerobic exercises (three times/week, 50–55 min/session) beginning from 2nd trimester	No reduction in GDM risk between groups. Other outcomes: 58% reduction macrosomia, 34% reducti in elective caesarean delivery and 12% reductic in maternal weight gain.
Barakat et al. 2011 (Ref 36)	RCT	Exercise programme with instructor (three sessions per week, n=40) vs non – exercise group (n=43)	25 min core session + 7-8 min gradual warm up and cool down beginning from 6-9 weeks to 38-39 weeks + one set of aerobic dance every 2 weeks for the intervention arm	Moderate PA during pregnancy improved maternal glucose toleranc
Poston et al. 2015 UK Pregnancies Better Eating and Activity (UPBEAT) trial. (Ref 37)	RCT	1,555 women with a BMI ≥ 30 kg/m2 between 15-18 weeks +	Behavioural intervention (n=772, healthy eating with low glycaemic load foods + 8 sessions with the health trainer on a weekly basis, each lasting 1 to 1.5 hours) vs standard care (n=783)	No difference in risk of GI or incidence of LGA babie

© Copyright: The University of Oxford 2019
For peer review only - http://bmjopen.bmj.com/site/about/guidelines.xhtml
For peer review only

Da Silva et al. 2017 (Ref 38) Physical activity during pregnancy and maternal-child health (PAMELA) Study.	RCT	Healthy pregnant women (n=639)	Exercise intervention thrice weekly for 16 weeks (from 16-20 to 32-36 GA)	No differences between t two groups in mean GWG GDM, BW, infant length, and head circumference.
Haakstad et al. 2011 (Ref 39)	RCT	Nulliparous, sedentary healthy pregnant women (n=105)	12-week supervised exercise programme (60 min supervised aerobic dance = strength training for 60min at least twice weekly)	Mean weight gain during pregnancy was lower in exercise compared to control group (11.0 +2.3 v 13.8 +3.8 kg, p 50.01)
Wang et al. 2017 (Ref 40)	RCT	Singleton pregnant women with BMI between 24 to 28 kg/m2 at 12+6 weeks of GA until 37 weeks	Exercise group vs controls Exercise 3 times per week (at least 30 min/session with a rating of perceived exertion between 12-14) via a cycling program at least 3 times/week	Lower GDM incidence in exercise vs control arm (22% vs 40.6%, p<0.001). Reduced GWG, IR, rates c caesarean delivery, fetal macrosomia and preterm births in exercise group

Studies investigating the effect of diet + PA during pregnancy

**Study reference**	**Type of study**	**Target population**	**Intervention**	**Findings**
Vinter et al. 2011 (Ref 41) Lifestyle in Pregnancy (LiP) study.	RCT	360 obese (pre-pregnant BMI 30-45 kg/m^2) pregnant women	Intervention program included dietary guidance + moderate PA for 30-60 min daily + closed training sessions with instructor for 1 h each week	Intervention group had a significantly lower median (range) GWG compared with the contro group of 7.0 (4.7–10.6) vs. 8.6 kg (5.7–11.5; P = 0.01)

For peer review only - http://bmjopen.bmj.com/site/about/guidelines.xhtml

Renault et al. 2014 (Ref 42) Treatment of Obese Pregnant Women (TOP) study.	RCT	425 obese pregnant women comparing 3 groups	PA + Diet, (n = 142)<br>PA intervention (daily step count of 11,000 steps/day every day for 4 weeks (n = 142)<br>Control group receiving standard care (n = 141)	PA intervention decreased GWG by a mean of 1.38 kg (P =0 .040).
Koivusalo et al 2016. (Ref 43)<br>The Finnish Gestational Diabetes Prevention Study (RADIEL study).	RCT	293 pregnant women with a history of GDM and/or a pre-pregnancy BMI of ≥30 kg/m2 at <20 weeks gestation	Intervention group received individualized counselling on diet, physical activity, and weight control. control group received standard antenatal care	GDM incidence of was 13.9% in the intervention group and 21.6% in the control group ([95% CI 0.4 0.98%]; P = 0.044.<br>GWG was significantly lower in the intervention group (p=0.037)
Dodd et al. 2014<br>(Ref 44, 45)<br>Limiting weight gain in overweight and obese women during pregnancy (LIMIT) trial	RCT	2,152 singleton pregnant mothers between 10-20 weeks GA and BMI ≥25kg/m^2	Diet and lifestyle advice group (n=1,075) vs standard care (n=1067)	Risk of LGA babies were similar in both groups<br>Babies of life-style advice group had higher mid-thig fat mass measured by US and rate of adipose tissue deposition was slow in thi group compared with control group.

For peer review only

Bennet et al. 2018 (Ref 46)	Systematic review and meta-analysis	45 RCTs	-	Diet and PA interventions aimed to reduce GWG wa[illegible] associated with reduced GDM risk by 44% and 38[illegible] respectively. Among Asia[illegible] the risk reduction with die[illegible] and life-style 62% and 32[illegible] respectively
Shepherd et al. 2017 (Ref 47)	Cochrane Database Systematic Review	23 RCTs and cluster-RCTs, comparing combined diet and exercise interventions with no intervention (i.e. standard care), that reported on GDM diagnosis as an outcome	-	Reduced GDM risk in intervention group compared with the standard care group (average risk ratio (RR) 0.[illegible] 95% CI 0.71 to 1.01. Reduced risk of caesarean section (RR 0.95, 95% CI 0.88 to 1.02) in interventi[illegible] arm
Petrella et al. 2014 (Ref 48)	Case-control study	Singleton pregnant women with BMI >25 kg/m^2	Therapeutic lifestyle changes (diet 1800kcal/day + mild PA 30min/d thrice weekly) (n=33) vs Controls (n=28)	Lower GWG, lower incidence of GDM, hypertensive disorders an[illegible] preterm births in the intervention group compared to controls.

For peer review only

© Copyright: The University of Oxford 2019
For peer review only - http://bmjopen.bmj.com/site/about/guidelines.xhtml

4. OUTCOME MEASURES

Outcomes	Measurements	Timepoint(s) of evaluation of this outcome measure (if applicable)
**Primary objective**		
Incidence of GDM	Fasting plasma glucose<br>Glucose at 1 hour and 2 hours' post 75g OGTT (according to IADPSG criteria) or Fasting hyperglycemia measured by fasting glucose	Between weeks 26-28<br><br>At 32 weeks
**Secondary objective**		
Effect of intervention on fasting blood glucose concentration	Fasting plasma glucose	Between weeks 26-28 and at<br>32 weeks of gestation
**Trial and post-trial surveillance measures**		
Gestational weight gain	Maternal weight	At screening, run-in-phase, randomization, visits 1,2,3 and<br>at delivery (before the birth of the child)
Gestational hypertension	Maternal blood pressure	At screening, run-in-phase, randomization, visits 1,2,3 and<br>at delivery (before the birth of the child)
Proportion of women undergoing Instrumental/caesarean delivery	Instrumental/caesarean delivery (elective and emergency)	At delivery
Post-partum haemorrhage (primary)	Amount of blood loss within 24 hours of child birth	From delivery to 24 hours
Blood loss at delivery	Amount of blood lost during delivery	At delivery
Pre-eclampsia and eclampsia	Clinical diagnosis (hypertension + proteinuria +/- seizures)	Clinical diagnosis after 28 weeks of GA
Pre-term births (<37 weeks of gestational age)	Gestational age calculated from dating scan	At birth of the new born
Foetal macrosomia	Birth weight (defined as birth weight >2 SDs above the population-specific mean in each setting)	At birth of the new born
Birth weight	Birth weight	At birth of the new born
APGAR score at 1 and 5 min of birth	Clinically recorded APGAR score	At birth of the new born

Length of the new born	Length measured from head to the heel	Within 48 hours of birth
Barriers to interventions in pregnancy 1 & 2	Questionnaire based assessment	At screening<br>At 32 weeks

5. STUDY DESIGN

Design: 2 x 2 factorial design, multi-centric, open labelled, randomized controlled trial
Total study duration: 36 months
Number of scheduled visits: 5

(i) **Screening visit (visit -2, ≤16 weeks GA):** Singleton pregnant women willing to participate in the study will be assessed for eligibility based on inclusion/exclusion criteria and eligible participants will be asked to sign an informed consent if willing to participate. Eligible women will undergo an oral glucose tolerance test (OGTT) to rule out impaired glucose intolerance or diabetes at screening and will also undergo dating ultrasound scan. Routine antenatal examination will be done. Barriers to life-style interventions-1 during pregnancy will be assessed using a questionnaire.

(ii) **Run-in visit (visit -1, ≤17 weeks GA)**: Baseline assessments of dairy product consumption will be done using standardized questionnaire . Additionally, objective measurements of baseline PA activity over 7 days will be assessed in all study participants (with the result blinded to participants) using a wearable accelerometer device (Garmin Vivofit 4 fitness band).

(iii) **Randomization (≤18 weeks GA):** Women will be randomized to either (i) diet (consumption of 200g of yoghurt per day), (ii) PA (a minimum of 40% increase above the recorded baseline PA) (iii) diet and PA or (iv) standard care. Active intervention will be from randomization to 32 weeks of GA.

(iv) **Visit 1 (18-20 weeks GA)**: maternal weight, blood pressure, routine antenatal examination and foetal morphology scan.

(v) **Visit 2 (26-28 weeks GA)**: maternal weight, blood pressure, routine antenatal examination and OGTT. Primary endpoint (diagnosis of GDM based on IADPSG critria) will be assessed at this visit. Women with GDM will continue on allocated study intervention until end of study (week 32) in addition to the clinical managemnt provided as per the local obstetric practice.

(vi) **Visit 3 (32 weeks GA, End of Study visit)**: maternal weight, blood pressure, routine antenatal examination, ultrasound foetal scan and fasting blood glucose and barriers to pregnancy intervention-2 assessment. (A repeat ultrasound scan at this stage is based on the evidence that growth restriction although starts in early pregnancy, is more marked between 22-34 weeks and also that foetal measurements do not have

For peer review only

exact equivalents to new born measurements. Therefore, a last trimester scan is essential as it reflects foetal growth more precisely).

(vii) **Delivery visit:** This is a post-trial surveillance visit primarily intended to assess the safety and efficacy of interventions on pregnancy and new-born outcomes.

Aside from the scheduled study visits, participants will be contacted on a weekly basis either over telephone or visited at home by research personnel. This will be made to ensure general well-being, compliance (wearing device, adhering to individually advised increment in step count, reminders of study visits, yoghurt consumption etc) and motivation.

Interventions

Dietary intervention Each participant in the diet and diet+PA arm will be advised to consume yoghurt 200 g/day once daily prepared locally at the dietary department in CMC, Vellore and from a local production in The Gambia. The yoghurt consumption is independent of the timing of their regular meal time (can be consumed anytime). Record of daily yoghurt and background dairy consumption will be maintained by every participant. The yoghurt will be prepared on a common recipe with the same starter culture at both centres. The yoghurt will be dispensed daily/weekly as feasible by the study centre. Adherence to yoghurt consumption will be monitored on a weekly basis by home visiting field staff. Dietary counselling will be reinforced in all participants and the non-diet arm will not be advised to stop their regular dairy consumption, assuming that any effect on outcome is related to the trial yoghurt consumed.

Physical activity intervention Women randomized to the PA and diet+PA arm will be provided PA targets to be followed throughout pregnancy based on their baseline PA assessment which was obtained at run-in-phase. The recommendation will be set based on a minimum of a 40% increase of the baseline step count measured during run-in-phase (refer to section 8.1.2 for details). The daily PA activity will be monitored using an accelerometer device (Garmin Vivofit 4 fitness band) which will provide visual feed back to the participants in the active PA arms in order to motivate participants and help them achieve their daily target step count. All other (yoghurt and standard arm) will also be provided with fitness bands to objectively measure their routine PA behavior throughout the study. However, step counts will be blinded for these participants. Data from these devices will be periodically uploaded using vivohub 2 for compliance monitoring and downloaded to provide visual feedback and counselling to participants.

Potential risks and benefits

Risks with study-related procedures

Risks with venepuncture may include bruising in the area of venipuncture, dizziness, nausea, vasovagal syncope. Consumption of oral glucose solution on an empty stomach may cause nausea, vomiting, headache. Ultrasound scans during pregnancy are not considered harmful. Utmost care would be taken to minimize such risks. A trained phlebotomist or research nurse will draw blood samples for the study. We will use clean, sterile phlebotomy equipment. Blood will be drawn using vacutainers with women sitting or lying down, whichever is comfortable. Local pressure will be applied for 20-30 seconds to avoid post-puncture bleeding.

For peer review only

Risks with interventions

There are no major serious adverse effects directly linked to the proposed interventions in the study. Both yoghurt and PA are generally considered safe during pregnancy. However, some potential adverse effects of the proposed interventions can be anticipated and are not necessarily caused by or related to the intervention. In the yoghurt consumption arm, anticipated risks may include nausea, vomiting and intolerance to yoghurt. In women undertaking physical activity, common adverse effects may include dizziness, headache, exertional dyspnoea, easy fatigability and muscle aches. The proposed PA in our study meet the safety guidelines for physical activity recommended by the ACOG (27). Safety will be ensured by excluding women who have absolute contraindications to aerobic physical activity during screening. Potential risks of PA may include persistent vaginal spotting/bleeding, calf pain/swelling (diagnosed to be related to thrombophlebitis or deep vein thrombosis), dyspnoea at rest, painful premature contractions, decreased foetal movements, preterm labour and preterm/premature rupture of membranes. A participant will be withdrawn from the study, in the event of persistent adverse events reported by the participant, and the PI feels that the event may be related to the intervention.

Data collection process

Data type	Method of collection	
	Gambia	Vellore
Weight (of the mother)	Digital Tanita scale	Micro weight electronics. Model: DS415 series
Height (of the mother)	Leicester height measure, seca 214	Stadiometer
Blood pressure	Electronic BP apparatus (Omron Corporation, Tokyo, Japan)	Electronic BP apparatus (Omron Corporation, Tokyo, Japan)
Baseline diet consumption	Dairy consumption questionnaire	Dairy consumption questionnaire
Barriers assessment	Questionnaire	Questionnaire
PA activity monitoring device	Garmin Vivofit 4 fitness band	Garmin Vivofit 4 fitness band
Height (of the baby)	Infantometer	Infantometer
Ultrasound	Aloka USI-145	Voluson GE S-8
Biochemistry		
Blood glucose	Roche Cobas 800<br>Enzymic (autoanalyser)	Roche Cobas 800<br>Enzymic (autoanalyser)

6. PARTICIPANT IDENTIFICATION AND RECRUITMENT

6.1. Study Participants

The study will include healthy pregnant women who have at least one “risk-factor” for developing gestational diabetes (see section 6.2 for high-risk definition).

For peer review only

6.2. Inclusion Criteria

- Pregnant women, aged ≥18 years
- Pregnant women ≤16 weeks of gestational age
- In addition to the above, pregnant women should meet at least one of the following criteria for high-risk GDM:
  - Booking BMI ≥25kg/m^2
  - Age ≥25 years
  - First-degree relative with diabetes
  - Previous pregnancy with GDM
  - Previous gestation with large baby (≥3.5kg)
  - Previous pregnancy with pre-eclampsia/eclampsia
  - History of PCOD/impaired fasting glucose
- Not currently on any medications (except iron, calcium or folic acid supplements, thyroxine supplement for hypothyroidism, low dose aspirin for pre-eclampsia)*
  *women conceived by ovulation induction using clomiphene citrate or metformin for PCOD will not be excluded from the study.

6.3. Exclusion Criteria

The participant may not enter the study if ANY of the following apply:

- GDM diagnosed prior to screening visit based on IADPSG criteria or documented raised HbA1C, i.e., either fasting glucose ≥5.1 mmol/L or 1h glucose ≥10.0 mmol/L or 2h glucose ≥8.5 mmol/L, or a documented HbA1c of ≥6.5% at first booking
- History of pre-gestational diabetes
- Multiple gestation in current pregnancy
- History of severe hyperemesis in the first trimester
- Uncontrolled pre-gestational or gestational hypertension (BP>150/100mm Hg) or on treatment
- History of recurrent (more than two) first trimester spontaneous abortions or stillbirths
- History of significant ante- or post-partum hemorrhage in the previous pregnancy
- Previous child born with congenital anomalies
- Pregnancy following in-vitro fertilization or any assisted reproductive technology and those unwilling to adhere to the study protocol will be excluded from the study
- Previous or current psychiatric illness on medication
- Previous or current neurological condition (e.g. epilepsy) or on medication
- Women meeting absolute contraindications for physical activity during pregnancy as recommended by the ACOG (heart disease, restrictive lung disease, incompetent cervix/cerclage, pregnancies at risk for premature labour, gestational hypertension, severe anaemia)
- Physical disability to PA and/or known lactose intolerance

7. STUDY PROCEDURES

The schedule of procedures at each visit is provided below

For peer review only

Visit	Study procedures
**Screening visit (≤16 weeks) (Visit -2)**	Height<br>Weight<br>Blood pressure (mother)<br>Antenatal examination (fundal height)<br>OGTT<br>Dating Ultrasound scan<br>Barriers to pregnancy intervention assessment-1
**Run-in-phase (≤17 weeks) (visit -1)**	Weight<br>Blood pressure<br>Antenatal examination (fundal height)<br>Questionnaire based assessment of background dairy consumption<br>Diet and PA counselling<br>Dispensing wearable activity monitors device for assessment of baseline PA for 7-days and compliance testing.
**Randomization (≤18 weeks) (visit 0)**	Weight<br>Blood pressure<br>Routine antenatal examination (fundal height)<br>Randomization
**Visit 1 (week 18-20)**	Weight<br>Blood pressure<br>Routine antenatal examination (fundal height)<br>Morphology scan
**Visit 2 (26-28 weeks)**	Weight<br>Blood pressure<br>Routine antenatal examination (fundal height)<br>OGTT
**Visit 3 (32 weeks) End of Study visit**	Weight<br>Blood pressure<br>Routine antenatal examination (fundal height)<br>Fasting plasma glucose<br>Ultrasound scan (to monitor foetal growth)<br>Barriers to pregnancy interventions assessment-2 (for future studies)
**Delivery (post-trial surveillance)**	Weight (mother)<br>Blood pressure (mother)<br>Intrapartum outcomes<br>Neonatal outcomes (Birth length, Birth weight, APGAR score)
**Telephonic contacts / Home visits**	General well-being<br>Compliance with interventions (step count from accelerometer, yoghurt pot-count)

7.1. Recruitment

In both India and The Gambia, women will be identified through home-visits by field workers and in the out-patient clinics of recruiting hospitals operated through the MRC Unit. In The

For peer review only

Gambia, the investigators will use additional strategies such as (i) community sensitization through village meetings that includes the community leaders, ward heads and their development committees, traditional birth attendants (TBAs) and community health workers (ii) house-to-house campaigns led by field workers and TBAs to encourage newly pregnant women to attend antenatal clinics (iii) Frequent use of the local radio stations for general community sensitisation and (iv) referrals from maternal and child health staff and obstetric radiologists to enhance participant identification and recruitment. In India, CMC, Vellore, recruitment will also be made from referrals from several community hospitals and peripheral health centres such as Community Health and Development Hospital (CHAD), Low-cost Effective Care Unit (LCECU), Rural Unit for Health and Social Affairs (RUSHA) and Community Health Nursing (CONCH) by engaging the rural community in grass root health and social programmes. Participants will be recruited by word of mouth.

7.2. Informed Consent

Written and verbal versions of the Participant Information sheet (PIS) and Informed Consent form (ICF) will be presented to the participants detailing no less than: the exact nature of the study; what it will involve for the participant; the implications and constraints of the protocol; the known side effects and any risks involved in taking part. It will be clearly stated that the participant is free to withdraw from the study at any time for any reason, and with no obligation to give the reason for withdrawal. As a part of consenting women will be informed that withdrawing consent from the study will not prejudice their medical care in any way.
The participant would be allowed as much time as wished to consider the information, and the opportunity to question the personnel administering the ICF (physician, medical research officer/midwife/field worker who are authorised to do so by the PI). The participant would provide his willingness to take part in the study by signing the ICF. In the event that the participant in uneducated, her left thumb impression would be obtained along with the signature of the neutral person who will serve as an impartial witness. A copy of the signed ICF would be given to the participant. The original signed form will be retained at the study site.

7.3. Screening and Eligibility Assessment:

Pregnant women aged ≥18 years and ≤16 weeks of gestation who are willing to participate in the study will be screened and assessed for eligibility.

In addition to above, participants should meet at least one of the below mentioned criteria to be termed as “high-risk” for inclusion into the study

- Booking BMI ≥25kg/m^2
- Age ≥25 years
- First-degree relative with diabetes
- Previous pregnancy with gestational diabetes mellitus
- Previous pregnancy with large baby (≥3.5kg)
- Previous pregnancy with pre-eclampsia/eclampsia
- History of PCOD/impaired fasting glucose

Women will then undergo a dating scan to confirm the GA and an OGTT (as described in section 7. Study procedures). Women with blood glucose values at fasting, 1 hour and 2 hours within

For peer review only

normal limits will move forward to the run-in-phase (visit -1) and others will be treated as "screen failure"

7.4. Randomisation

Eligible women will be randomly allocated to one of the following arms (i) Yoghurt (ii) PA (iii) Yoghurt + PA or to the (iv) control group (neither intervention, standard care) following an allocation schedule prestratified for study centre, age and BMI generated by an independent statistician not involved in the study. A variable block randomisation, stratified by centre, and then by age, (<25 and ≥25 years) and BMI (<25 and ≥25 kg/m2) will be generated, using a password-protected Access database developed by an independent statistician who is not involved with trial participants or data analysis. A unique study ID will be assigned to each participant at screening by the research officer.

In India, the random allocation of each participant to one of the four intervention groups, stratified by age*BMI will be performed using a computer interface (Access database with forms and data separated into a frontend and password backend, respectively).

In Gambia, due to logistical limitations, separate lists will be generated for each strata combination (age x BMI) and randomisation assigned using opaque, sealed envelopes. As this is an open-labelled study, study participants, and investigators will not be blinded to the treatment arm. Laboratory personnel and the trial statistician will be blinded.

7.5. Baseline Assessments

Baseline assessments are divided over first three visits (screening, run-in-visit and randomization)

7.5.1. Baseline assessments at screening visit (Visit: -2; ≤16 weeks)

I. Eligibility check
II. **Height:** Height of the pregnant mother will be measured using a portable or fixed stadiometer.
III. **Weight:** Weight of the pregnant mother will be measured using a calibrated electronic weighing scale to the nearest 0.1kg.
IV. **Blood pressure**: Systolic and diastolic blood pressure will be measured after 5 minutes seated at rest. It will be measured three times using an automated validated device (Omron M3, Tokyo, Japan), with an appropriately sized cuff. Three measurements will be recorded in a seated position by a trained research staff. The average of three values will be taken for the final analysis.
V. **Antenatal examination (fundal height):** Routine antenatal examination will be carried out according to local obstetric practice.
VI. **Dating ultrasound scan:** The dating scan will be done at screening visit in all participants meeting the eligibility criteria. The following variables will be measured and recorded: Type of gestation –single/twins/triplets, gestational sac, crown-rump length, yolk sac, cardiac activity, gestational age by scan, scan expected date of confinement. Women diagnosed with multiple gestation on scan, gestational week corresponding to more than

For peer review only

1 week greater than the scheduled visit date and absent in-utero gestational sac will be screen failed.

VII. **Barriers to pregnancy intervention assessment-1:** This will be a questionnaire based assessment that includes questions relating to assessment of overall perceived barriers to life-style interventions in pregnancy, perceived difficulties to frequency of study visits, perceived willingness to interventions and study-related procedures, 9-point willingness to participate score, willingness and unwillingness to participate.

VIII. **OGTT:** A fasting glucose venous blood glucose sample taken will be following a minimum 8-hour fast. Participants will be then asked to drink a calibrated dose (75 grams) of anhydrous glucose solution within 10 minutes as soon as possible. Subsequent blood samples will be collected at 60 and 120 minutes following the drink. Based on IADPSG criteria, a diagnosis of GDM will be made if one or more values equals or exceeds the following:

	Values in mmol/l	Values in mg/dl
Fasting glucose	≥5.1 mmol/l	92 mg/dl
1-hour glucose	≥10.0 mmol/l	180 mg/dl
2-hour glucose	≥8.5 mmol/l	153 mg/dl

Women qualifying the criteria for diabetes (based on IADPSG cut-offs) at this stage will be screen failed.

7.5.2. Baseline assessment at Run-in visit (Visit: -1; week ≤17)

I. **Weight:** Weight of the mother will be recorded as described in 7.5.1 (II)

II. **Blood pressure:** Systolic and diastolic BP of the mother will be recorded as described in 7.5.1 (III)

III. **Antenatal examination (fundal height):** Routine antenatal examination including fundal height will be recorded as described in 7.5.1 (IV)

IV. **Dairy consumption assessment:** Background dairy product consumption of the study participant will be assessed using standardized questionnaires.

V. **Safety assessments** to interventions (e.g. adverse events and/or SAE assessment)

VI. **Concomitant medication** assessment

VII. **Baseline PA monitoring:** All participants will be advised to wear an accelerometer device to objectively measure baseline PA for next 7 days. The step counts will be blinded (not made visible on the device) in order to avoid influences on routine activity.

7.5.3. Baseline assessment at Randomization (Visit: 0; week ≤18)

I. **Weight:** Weight of the mother will be recorded as described in 7.5.1 (II)

II. **Blood pressure:** Systolic and diastolic BP of the mother will be recorded as described in 7.5.1 (III)

For peer review only

III. **Antenatal examination (fundal height):** Routine antenatal examination including fundal height will be recorded as described in 7.5.1 (IV)
IV. **Intervention allocation**
V. **Compliance assessment:** to interventions (step count, yoghurt diary)
VI. **Safety assessments** to interventions (e.g. adverse events and/or SAE assessment)
VII. **Concomitant medication** assessment.

7.6. Subsequent Visits

7.6.1. Visit 1 (week 18-20)

I. **Weight:** Weight of the mother will be recorded as described in 7.5.1 (II)
II. **Blood pressure:** Systolic and diastolic BP of the mother will be recorded as described in 7.5.1 (III)
III. **Antenatal examination (fundal height):** Routine antenatal examination including fundal height will be recorded as described in 7.5.1 (IV)
IV. **Safety assessments** to interventions (e.g. adverse events and/or SAE assessment).
V. **Concomitant medication** assessment
VI. **Compliance assessment:** to interventions (step count, yoghurt diary)
VII. **Morphology scan:** The following variables would be measured and recorded: Gestational age, bi-parietal diameter, head circumference, femoral length, Abdominal circumference, estimated foetal weight, liquor amount, placenta: anterior/posterior and upper segment/lower segment, amniotic Fluid Volume Index, Uterine artery Doppler: Pulsatility Index, Systolic/diastolic ratio, gross anomalies in region of head and neck (transcerebellar plane, transventricular plane, biparietal plane, nose/lips), thorax (lungs, 4 chamber view, outflow tract, diaphragm), Spine, Abdomen (stomach bubble, kidneys, cord insertion, number of vessels in cord, genitalia, limbs). Participants with any foetal congenital anomaly, placental malformation or malposition or abnormal ultrasound features that can compromise pregnancy outcome according to the investigator will be withdrawn from the study at this stage.

7.6.2. Visit 2 (week 26-28)

I. **Weight:** Weight of the mother will be recorded as described in 7.5.1 (II)
II. **Blood pressure:** Systolic and diastolic BP of the mother will be recorded as described in 7.5.1 (III)
III. **Antenatal examination (fundal height):** Routine antenatal examination including fundal height will be recorded as described in 7.5.1 (IV)
IV. **OGTT** (as described in in 7.5.1 (VIII))
V. **Safety assessments** to interventions (e.g. adverse events and/or SAE assessment) and review of concomitant medications.
VI. **Compliance assessment:** to interventions (step count, yoghurt diary)

For peer review only

7.6.3. Visit 3 (Week 32) - END OF STUDY VISIT

I. **Weight:** Weight of the mother will be recorded as described in 7.5.1 (II)
II. **Blood pressure:** Systolic and diastolic BP of the mother will be recorded as described in 7.5.1 (III)
III. **Antenatal examination (fundal height):** Routine antenatal examination including fundal height will be recorded as described in 7.5.1 (IV)
IV. **Fasting plasma glucose**
V. **Barriers** to pregnancy intervention-2 assessment
VI. **Ultrasound scan:** The ultrasound scan would be done during visit 3 (week 32) in all participants which would include measurement of bi-parietal diameter (BPD), head circumference, femoral length, abdominal circumference, estimated foetal weight, liquor amount, placenta: anterior/posterior and upper segment/lower segment, amniotic Fluid Volume Index, Uterine artery Doppler: Pulsatility Index, Systolic/diastolic ratio.
VII. **Safety assessments** to interventions (e.g. adverse events and/or SAE assessment) and review of concomitant medications.
VIII. **Compliance assessment:** to interventions (step count, yoghurt diary) & close of interventions.

In the event the participant withdraws from the study at any time prior to week 32 visit, then the date of voluntary withdrawal will be marked as "END OF STUDY VISIT". The participant will undergo all assessments as listed in 7.6.3 except morphology scan.

7.6.4. Post-intervention surveillance visit

All study-related intervention will be stopped at 32 weeks and mother and new born data will be collected during delivery as a part of post- trial surveillance visit. Women who consent will have weekly telephonic contacts/home visits by field staff to ensure general wellbeing. All women will be delivered in the hospital and inter-partum and neonatal measurements will be recorded as below

I. **Weight of the mother** before delivery of the baby
II. **Intrapartum maternal outcome assessment**
III. **Birth length (new born)** The measurement from the crown to the soles of the feet in centimetre scale would be recorded as birth length by an experienced mid-wife.
IV. **Birth weight (new born)** Weight at birth would be measured within 48 hours of delivery using a calibrated scale for measuring neonatal birth weight by an experienced mid-wife.
V. **APGAR score (new born)** The APGAR Score should be used as the accepted and convenient method for reporting the status of the new born immediately after birth and the response to resuscitation if needed. Local neonatal practices should be followed for APGAR score reporting in cases of maternal sedation or anaesthesia, congenital malformations and birth trauma. The APGAR assessment would be made as below by a trained medical neonatologist/paediatrician or midwife at 1 and 5 minutes.

For peer review only

Sign	0	1	2
Colour	Blue or Pale	Acrocyanotic	Pink
Heart rate	Absent	<100/minute	>100/minute
Reflex irritability	No response	Grimace	Cry or Active withdrawal
Muscle tone	Limp	Some flexion	Active motion
Respiration	Absent	Weak Cry	Good, Crying

All women recruited in the study will be regularly contacted over telephone or by home visit to monitor general well-being, diet and PA adherence and early detection of serious adverse events and safety to interventions. The additional weekly phone review is also expected to assist with adherence and retention.

7.7. Sample Handling

Blood sample

Following a minimum of 8 hour overnight fast, women will provide a fasting sample followed by blood samples at 1 hour and 2 hours of consuming oral glucose solution (see Section 7. Oral Glucose Tolerance Test). 2ml venous blood will be collected for each measurement in an EDTA fluoride vacutainer tube (Grey Capped) and placed on ice until centrifuge. Samples will be centrifuged (2,750 rpm for 10 minutes), aliquoted and transported to the analysis lab on ice. OGTT glucose samples and fasting venous glucose during visit 3 will be analysed on Roche Cobas 800 Autoanalyser either on the same day or will be stored in freezer if analysed on the subsequent date. A total of 12 ml blood will be collected during both OGTTs. General timelines for sample handling will be followed (venepuncture to ice no more than 10 minutes, ice to centrifuge no longer than 30 minutes, centrifuge to ice no more than 10 minutes and ice to freezer no more than 6 hours). No samples will be stored for future use. The current study does not involve any genetic or molecular analysis.

7.8. Discontinuation/Withdrawal

Discontinuation/withdrawal has to be distinguished from i) discontinuation from the study (interventions, follow-up visits, study-related procedures) and ii) discontinuation from interventions only and could occur under following circumstances

7.8.1. Discontinuation/Withdrawal from the study

Discontinuation from the study occurs

- When a participant voluntary withdraws consent to further participation at any time during the trial.

For peer review only

- Significant protocol deviation such as Ineligibility (either arising during the study or retrospectively having been overlooked at screening)
- Loss to follow-up

Details of withdrawing/discontinuation from the study prior to completion of all scheduled visits should entered in the "study termination form"

Participants who are diagnosed to have GDM (by laboratory evidence within the protocol or outside diagnosis confirmed by an OGTT at study centre) should continue interventions till week 32 (end of study visit), unless there is a safety concern.

Temporary or permanent discontinuation of interventions (as detailed in sections 7.8.1 and 7.8.2) for safety reasons will not lead to withdrawal of the participant from the trial. Withdrawal of the participants will not be replaced by recruitment of new participants. The primary reason for withdrawal will be specified in the Case Report Form.

7.8.2. Discontinuation/Withdrawal from interventions

7.8.2.1. Temporary discontinuation/withdrawal from interventions

Temporary discontinuation from interventions may occur when a participant does not tolerate the intervention or due to a ***suspected AE*** by the investigator. Best efforts should be made to resume the intervention as early as possible.

7.8.2.2. Permanent discontinuation/withdrawal from interventions

Permanent discontinuation from intervention is any intervention discontinuation associated with the definitive decision from the Investigator not to re-expose the participant to the either yoghurt or PA or both at any time during the study. This may occur during situations of ***unexpected SAE***, where, in the investigator's opinion, continuation with intervention would be detrimental to the participant's well-being

Examples of permanent discontinuation could be

- development of blood pressure 150/100 mmHg anytime during the trial
- Hospitalisation due to medical event (only if further continuation of interventions will affect maternal or new born health)
- Anaemia diagnosed based on haemoglobin <10g/dl
- Ultrasound evidence of major degree placenta previa

Participants will be followed-up according to the study procedures specified in this protocol up to the scheduled date of study completion. It is important to collect data on all participants, regardless of intervention, during the entire duration of the study as all data until the scheduled date of study completion will be used in statistical analyses.

In case the participant decides to withdraw participation or consent during the study, we will not work on participant's samples without permission, but any information already generated from the samples until the time of withdrawal will be used and samples already collected, for which they have given consent, will also be analysed and data used.

For peer review only

7.9. Lost to follow-up

A subject will be considered potentially lost to follow-up if she completed all protocol specific procedures up to the administration of the intervention, but was then lost during the follow-up period, with no safety information and no efficacy endpoint data ever became available. This could occur in situations where a participant repeatedly fails to return for scheduled visits and is unable to be contacted by the trial site.

- Before a subject is deemed lost to follow-up, the investigator should make every effort to regain contact with the subject.
- A subject will not be declared lost to follow-up before all the attempts have been repeated and the trial has come to an end.

The attempts must be documented in the CRF.

7.10. Protocol deviation

A protocol deviation is any noncompliance with the clinical trial protocol, good clinical practice (GCP), or other applicable regulatory requirements. The noncompliance may be either on the part of the participant or the investigator including the study team members, and may result in significant added risk to the study participant.

Deviations from the protocol should be avoided. If deviations do occur, they must be documented and explained in a protocol deviation CRF by stating the reason, date and the action(s) taken. As a result of a deviation, corrective action should be developed and implemented promptly. The investigator must inform the trial monitor and the implications of the deviation must be reviewed and discussed. All protocol deviations must be notified to the regulatory authorities according to local requirements

Deviation from the protocol can occur at following circumstances

- When the investigators miss to schedule a visit
- If an ineligible subject is randomised in error/randomisation stratification error

7.11. Definition of End of Study

End of study will be the last scheduled visit as per protocol i.e., visit 3 (week 32). However, if the participant chooses to discontinue form the study due to any reasons as stated above in 7.8, the "study termination form" should be completed.

8. INTERVENTIONS

The study will be a 2 x 2 factorial design and eligible women will be randomized to either one of the arms – (i) Yoghurt (ii) Physical activity (PA) (iii) Yoghurt + PA (iv) Standard care

8.1.1. Yoghurt intervention

Women randomized to 'Yoghurt' and 'Yoghurt + PA' arms will consume 1 pot (200g) of yoghurt daily from the day of randomization visit to 32 weeks of gestation (more details provided under section 5 Study design – interventions).

8.1.2. PA intervention

For peer review only

Women randomized to 'PA' and 'Yoghurt + PA' arms will follow PA intervention (daily walking and weekly group activity sessions) from randomization to Visit 3 (week 32).

8.1.2.1. Daily walking

"Absolute target" will be calculated as a minimum of a 40% increase from the baseline. This will be calculated as the average of the weekly step count at run-in-phase. Step counts will be monitored using Garmin vivofit 4 band from randomization. Participants will be advised to use the "absolute target" as the minimum reference step count, but try to increase the step count beyond this. Women will be encouraged to perform walking at as speed faster than usual but within the individual's comfort.

Compliance will be calculated based on average step count achieved over a 7-day period including two weekend days. Women achieving at least 80% of the absolute target on a 7-day average will be considered as compliant. Non-compliant participants will be continually motivated and encouraged to achieve their absolute targets either telephonically or by home visits by field workers.

Baseline a	40% increase b	Minimum absolute target (a+b)	80% of absolute target (d=a+b) increase (expected average 1 week step count to be achieved) Used to calculate compliance
2500	1000	3500	3300
3000	1200	4200	3960
3500	1400	4900	4620
4000	1600	5600	5280
4500	1800	6300	5940
5000	2000	7000	6600
5500	2200	7700	7260
6000	2400	8400	7920
6500	2600	9100	8580
7000	2800	9800	9240
7500	3000	10500	9900
8000	3200	11200	10560
8500	3400	11900	11220
9000	3600	12600	11880

For example, if baseline activity is 6,000 steps, then an increase of 40% would be an increase of 2,400 steps a day and absolute target to be achieved/day would be a minimum of 8,400 steps.

For peer review only

9. SAFETY REPORTING

Safety evaluations will be performed on all randomized participants at each follow-up visit (Visit 1-3). All AE and SAE, including expected SAEs will be captured in the AE report form. The participants will be instructed to contact the study team if there are any concerns regarding the interventions.

9.1. Adverse event reporting

Any adverse event reported by the participant will be captured in the 'adverse event report' form. Information on AE will be collected by the treating physician at each scheduled visit and by the field worker during home visits. Besides scheduled study visits, AEs will be collected through weekly phone reviews. Any participant reporting an AE will be seen by the research clinician at the earliest appointment (depending on the severity). Details of all events including onset date, causality to the study intervention will be reported, treatment given, stop date will be captured in the respective AE report form.

9.2. Definition of Serious Adverse Events

An untoward medical event leading to /resulting in any of the following will be considered as "serious adverse event"

- Death
- is life-threatening
- requires inpatient hospitalisation or prolongation of existing hospitalisation
- results in persistent or significant disability/incapacity
- loss of foetal movements or ultrasonographically confirmed foetal death
- 'Important medical events' if they jeopardise the participant or require an intervention to prevent one of the above consequences.

9.2.1. Expected SAE

Expected SAEs include potential medical events (as listed below) will be considered as they are likely to be related to the interventions. These events are therefore **protocol-defined exceptions to SAE reporting**. These events should be captured in the SAE form, but does not require reporting to DMC within scheduled timelines. However, local ethics committees should be informed of such events if the principal investigator believes the event is related to PRIMORDIAL interventions.

- persistent vaginal spotting/bleeding after randomisation
- calf pain/swelling (diagnosed to be related to thrombophlebitis or deep vein thrombosis)
- Dyspnoea at rest
- painful premature contractions
- decreased foetal movements
- preterm labour
- preterm/premature rupture of membranes
- Diagnosis of placenta praevia after 26 weeks of pregnancy
- Pre-eclampsia / Eclampsia

For peer review only

9.3. Reporting Procedures for Serious Adverse Events

All research staff will undergo formal GCP training as well as on reporting procedures for AEs and SAEs.

The PI shall report all SAEs without filtration, whether or not related to the intervention, within 24 hours of becoming aware of the event to the Sponsor. If the SAE is related to the intervention, the respective Ethics Committee should be notified within seven calendar days if fatal or life-threatening, and all others within 15 calendar days.

The minimum information required for this initial SAE report is:

- Trial number and (short) title
- Participant's ID
- Date and time of onset
- Description of the event (clinical history, associated signs and symptoms)
- Intervention administered
- Reporter's name

The PI/study coordinator will not wait for additional information to fully document the event before notifying. The report is then to be followed by submission of a completed SAE Report Form as soon as possible, detailing relevant aspects of the SAE in question. All actions taken by the PI and the outcome of the event must also be reported immediately. A decision whether to continue/discontinue the intervention (yoghurt+/- PA) will be made by the treating obstetrician depending on the causality of the event.

For documentation of the SAE, any actions taken, outcome and follow-up, a SAE Report Forms will be used. All follow-up activities have to be reported, if necessary on one or more consecutive SAE report forms in a timely manner. All fields with additional or changed information must be completed and the report form should be forwarded to the respective Ethics Committee within seven calendar days after receipt of the new information. Hospital case records and autopsy reports, including verbal autopsy, will be obtained where applicable.

All SAEs would be reported to the Data Monitoring Committee (DMC) and Oxford Tropical Research Ethics Committee (OxTREC) where in the opinion of the PI the event was 'related' (resulted from administration of any of the research procedures) and 'unexpected' (the type of event is not listed in the protocol as an expected SAE). Reports of related and unexpected SAEs should be submitted within 15 days of the PI becoming aware of the event to DMC and OxTREC.

10. STATISTICS AND ANALYSIS

10.1. Description of Statistical Methods

We will analyse the study using both (1) the intention-to-treat principle and (2) per protocol, including only those participants adhering to at least 75% of the proposed intervention. In addition, we will run a sensitivity analysis to assess the effect of protocol deviation compared to 100% adherence. Analyses will be performed blinded to the intervention group allocation.

For peer review only

Descriptive analysis of the first 40 participants who complete visit 2 (26-28 weeks) will be performed as advised by the funders (MRC-UK). The analysis is intended to assess feasibility, retention and adherence to all study-related procedures.

10.2. The Number of Participants

The sample size is 1856 high-risk women (928 to be recruited in The Gambia and India). The study was powered to have 80% to detect a 33% reduction in the incidence of GDM, for the main effects of diet and PA, with a family-wise significance level of 5% for a two-sided test (i.e. each effect was tested at a significance level of 2.5%). It was assumed that participants could be pooled across centres (no interaction between interventions and country at the 5% level). The sample size was inflated to allow for a 15% drop out rate in each of the four strata, with drop outs defined as non-compliant participants (i.e. no risk reduction in their incidence of GDM).

The effect sizes for the sample size calculation were:

Prevalence of GDM in high-risk pregnant women: The largest study of GDM women (The HAPO study, 25,505 pregnant women unselected for GDM risk at 15 centres in nine countries) reported a GDM prevalence rate, based on IADPSG criteria, of 17.8% among the general population. In our 'high-risk' population, we assume that the prevalence will be higher than in the HAPO study. Therefore, we have estimated the prevalence of 25% in our study.

Estimated risk reduction: A recent meta-analysis (Song et al. Obesity reviews 2016) has shown that either diet or PA results in 18% (95% CI 5-30%) risk reduction in GDM in general pregnant population and interventions as early as 15 weeks of gestational age was also associated with reduced GDM risk (Relative risk: 0.80, 95%CI 0.66-0.97). Putting all these together, we estimate the risk reduction would be higher (about 1/3, 33%) in this high-risk population. We modelled the GDM percentages in four equal-sized combinations of control (c) and treatment (t) arms as 25% (c-c), 16.75% (c-t and t-c) and 11.2 in t-t group. Therefore, in simple comparisons of one exposure, the percentages will be 20.9% vs 13.0%.

*Absence of intervention*country interaction:* In order to pool the participants of both countries, we will be assuming that there are no differences between the participants of both countries. This initial test for a non-significant interaction term (at the 5% level) between each intervention and country was also taken into account in the sample size simulation.

The sample size calculation was performed by simulation (in R version 3.5.2) according to the following algorithim:

1. For a given sample size, we generated 8 subgroups, two (one per country) for each intervention combination (control-control, treatment1-control, control-treatment2, treatment1-treatment2).

2. For each subgroup we generated the expected GDM incidence, except for a random 15% (drop-outs), whose expected GDM incidence was considered to be 25% (corresponding to the control group) regardless of their originally allocated subgroup.

For peer review only

3. We first tested if either the interaction term site*PA or site*Yoghurt was statistically significant (at the 5% level).

4. If the assumption of no intervention*country interaction was met, we recorded for each intervention if it was statistically significant (at the 2.5%, as we were doing two tests, one for each intervention), assuming a two-sided test

5. This was repeated 10,000 times.

6. The % of repetitions with statistically significant intervention effects represents the power associated with the sample size.

7. Steps 1-6 were repeated for increasing sample sizes.

8. A smoothed (lowess) line was fitted.

9. The point where this line crossed the 80% power mark gave us the final sample size required. (Figure 1)

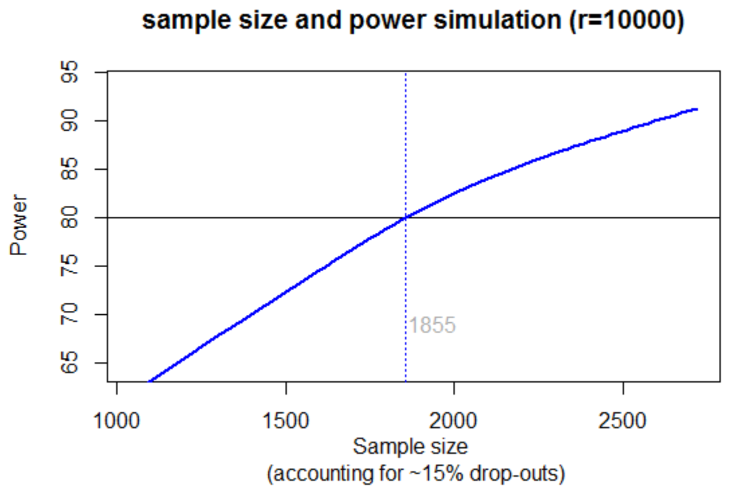

Figure 1: Estimated power for increasing sample size, with an estimated risk reduction of 33%, alpha = 0.05 and estimated drop-out rate of 15% (based on 10,000 iterations).

10.3. Analysis of Outcome Measures

All outcomes will be presented using descriptive statistics; normally distributed data by the mean and standard deviation and skewed distributions by the median and interquartile range. Binary variables will be presented using counts and percentages. We will summarise descriptive data using CONSORT reporting standards for RCTs. STATA will be used for statistical analysis.

10.3.1. Primary outcome

Gestational diabetes incidence will be analysed as risk ratios and risk differences will be estimated by binary regression and results presented as odds ratio and 95% confidence interval.

A covariate adjusted analysis of the effect of treatment on the primary outcome will be undertaken to remove bias from the estimate of the treatment effect on the primary outcome.

We will also test for the difference in effects on GDM incidence between the centres. If the odds ratio (OR) for GDM associated with the intervention in The Gambia is ORg, and in India is ORi, then the ratio of these two terms measures the difference in effect.

10.3.2. Secondary outcomes

The secondary outcomes will compare the difference in fasting plasma glucose at week 32, gestational weight gain, intrapartum and neonatal outcomes between the intervention and control groups. Continuous variables will be analysed using multiple linear regression and binary variables by logistic regression and adjusted for pre-specified covariates.

10.3.3. Safety

The incidence rates of AEs and SAEs and their relationship to trial interventions will be summarised by intervention group. The proportion of women discontinuing treatment will be summarised by reason and by intervention group.

11. DATA MANAGEMENT

All protocol required data will be captured in electronic Case Report forms (eCRF) that will be completed for each included participant by trial personnel. Data will be regularly checked by the Data Manager. On the eCRF, a reference to the source document will be provided. Instructions for completing all forms, including the CRF, used in the study will be developed. All data will be entered onto a secured, password-protected database that will only be accessed by the data manager and designated investigators.

11.1. Types of data

The study will include collection of both research (quantitative and qualitative) and personal data. Data minimisation has been considered and thus personal data will only include name, addresses and telephone number collected for the purpose of telephonic follow-ups/reminders and home visits by the field staff for the current study. These data will however be also used if the participant needs to be contacted for future approved studies (follow-up of babies born to high-risk GDM mothers). Personal information will be available only to the study PI and designated staff involved in contacting the participants either telephonically or by home visits. As this study has the potential to be extended in the future for other follow-up studies, personal data will pseudonymised and stored locally at their study centres. There will be no transfer/sharing of personal data outside the study centre. This personal data will be not be linked to research data and will be maintained in separate files at each study centre and will be password protected and accessible only to authorized staff of the study team.

11.2. Format and scale of data

REDCap software will be used in the trial. It is a secure web application for building and managing databases. The final data format will be downloaded to Excel and common statistical packages (SPSS, SAS, Stata, R). REDCap is specifically geared to support online or offline data

For peer review only

capture for research studies and operations. This data management system is a fully supported, externally validated (GCP compliant to 21 CFR parts 11) clinical database. The database will be hosted and managed by the MRCG and the final tested production database will be shared with the partner site in India who will enter/synchronize data into the central database.

11.3. Data collection/generation

Research data will be collected using electronic CRFs (eCRFs). Data collection will be undertaken by designated study staff involved in the study. Only those individuals who have been trained on the CRF/eCRFs and/or other document completion and have been authorized by the Site PI may complete eCRFs and other study specific documentation. The research data collected on the eCRFs will be captured using devices i.e. tablets and/or laptops used by study staff. Where there's internet connectivity, collected data will be stored directly onto the central server and where there is no/unreliable connectivity, data will be stored locally on the tablet and later synchronized to the central servers at MRCG Fajara. Field staff will be trained to use of the REDCap mobile application and clinicians will be trained to use the REDCap web portal. The data from the accelerometer used for monitoring PA will be directly downloaded on the tablet by the trained research staff on a weekly basis.

11.4. Data quality and standards

Review of data collection tools against the protocol will occur prior to database build by the Data Developer to ensure all data is being captured, at the correct time points, and in a format conducive to the proposed statistical analysis. Site based staff will be fully trained in conducting any study assessments and subsequent completion of the CRF and a Site delegation log will be maintained. Study staff will be trained in the use of Redcap. Principles of GCP will be adhered to and all training will be documented. To ensure standardisation of processes, standard operating procedures with respect to trial management, quality assurance, data management, IT & security, and statistics will be adhered to. The entry screens will be designed with range checks, skip patterns and validations to assist/guide data collectors and to ensure high level accuracy. Multiple choice options will be employed when and where applicable. Mandatory field checks and other computable data will be pre-populated to eliminate errors. The data manager will conduct periodical data cleaning routines to flag data queries that were not picked up in the earlier stages. All data queries raised by the data manager will be answered by the local PI or a delegated senior member of the study team involved in the generation of the data in question normally within one week. All changes to any CRFs or databases will be governed by a change control SOP, ensuring that all sites are using the same version of both CRFs and databases.

11.5. Managing, storing and curating data

Paper records will be stored securely in locked rooms or cabinets, according to standard filing and archiving procedures by both centres. Electronic data collected at both sites (India and The Gambia) will be centrally monitored and stored by the MRC Unit, The Gambia. The database will be housed on a secure network server with no direct physical access to it by project team members. Access to the system is via a web interface that will be hosted on the Fajara production server (https://redcap.mrc.gm/redcap/redcap) managed by the IT department. All data stored in REDCap will be formatted in XML when exported using Clinical Data. Interchange Standards Consortium Open Data Model (CDISC ODM) export format which is vendor neutral,

For peer review only

platform independent format for interchange and archive of clinical trials data. The database will be backed up as part of the IT department's backup routine and disaster recovery process. ICF will be maintained locally in the respective study centres in secure cabinets will limited access to authorized study staff only. The ICF will be retained for a period of 5 years after study completion and publication of primary results. Other PID (personally identifiable data) will be stored indefinitely for the purpose of future follow-up studies.

11.6. Data preservation strategy and standards

At the end of the study (when the last participant enrolled in the study completes the delivery visit) the database will be locked and all the electronic study data will be housed in the REDCap Database server until the first publication is made following which the data will be sent to the electronic data archives for long-term preservation. The locked database will have very highly controlled access as detailed in the Archives Research Records Management Policy (POL-ARM-001 - Available on request). All electronic records will be kept indefinitely and all paper records and lab books will be stored in the on-site temperature and pest controlled MRC as dictated by the MRC Unit The Gambia's SOPs on Data Retention and Destruction (SOP-ARM-005) and formal information/data security standards The study will be run in compliance with the MRC Corporate Information security Policy and the MRC Unit The Gambia's INFORMATION AND COMMUNICATION TECHNOLOGY SECURITY POL-INT-001 available on the MRC Gambia intranet (Available on request).

11.7. Main risks to data security

Main risk to confidentiality is the personal information on subjects kept by the local-PI for study follow up verification purposes. These will be kept separate from the actual research data and not available to the study staff so that no linkages of personal data to study records would be easily made. The research data will be stored in a limited access, password protected database so that only staff who have the required permissions can view the study records. Personal information will be available only to study PI and designated staff involved in contacting the participants either telephonically or by home visits.

11.8. Suitability for sharing

The data generated within the study will be suitable for sharing in an anonymised format. The data will be in a format that is suitable to share with other interested researchers. All data meant for sharing will be anonymised/de-identified by removing all individual-level participant data (IPD) and will be archived on the servers of the Archives department of MRCG. Data sharing will be in agreement with the MRCG as outlined and governed by the Data Sharing Policies i.e. Data Sharing Policy v2.2 and MRC Policy and Guidance on Sharing of Research Data from Population & Patient Studies. The regulations will also adhere to the University of Oxford data policy guidelines. PID will not be shared across organisations.

11.9. Access to Data

Direct access will be granted to authorised representatives from the University of Oxford and any host institution for monitoring and/or audit of the study to ensure compliance with regulations.

For peer review only

11.10. Data Handling and Record Keeping

All study data will be downloaded from RedCap into an Excel spreadsheet. The participants will be identified by a unique study specific number in any database. The name, addresses and any other identifying details will NOT be included in any study data electronic file. All data will be fully described in the Excel data dictionary which will include the variable name, variable description, label (if different from description), database table name, data grouping information, type of data (sting, integer, real etc.), data codes and decodes and any validation rules used. All data stored in REDCap will be formatted. Person data and research data will not be linked. After completion of the study, research data will be stored indefinitely at central servers in MRC, Fajara, The Gambia. PID including ICF will be stored for a minimal period of 3 years after study completion and destroyed subsequently as per local data management policies. All data management processes and procedures will be governed by the library of MRCG's data management standard operating procedures.

12. COMMITTEES STRUCTURES

The study will be conducted in accordance with the current approved protocol, Good Clinical Practice (GCP) regulations and standard operating procedures (SOPs).

12.1. DATA SAFETY MONITORING COMMITTEE (DSMC)

An independent Data Safety Monitoring Committee (DSMC) has been set up to review the progress of the trial and provide feedback to the Trial Steering Committee (TSC).

The DMC will be responsible for reviewing

(i) general study progress (recruitment, compliance, loss to follow-up)
(ii) clinical study safety data and assess and/or adjudicate all events of deaths
(iii) the GDM event rate and key secondary/safety outcomes
(iv) results of the interim analysis. The DSMC includes the following members (To address: chair of DSMC, Report preparation, confirm meeting timelines).

The DMC will review and analyze, on a regular basis, unblinded safety data throughout the study.

An independent DSMC with members who are independent from the Sponsor and the investigators for the PRIMORDIAL study comprises of experts in clinical trials/epidemiology, biostatistics and obstetrics and include the following members

Name (Designation)	Address	Email
Dr. Udak Okoma (Paediatrician & Postdoctoral Research Fellow)	MRC Unit The Gambia at the London School of Hygiene & Tropical Medicine, Fajara, Banjul, The Gambia	uokomo@mrc.gm
Dr. Matthew Johnson (Biostatistician)	MRC-LEU, University of Southampton, UK	mj2@mrc.soton.ac.uk

Dr. Lucy McKillop (Obstetrician)	Nuffield Department of Obstetrics and Gynaecology, Oxford, UK.	lucy.mackillop@ouh.nhs.uk

Meetings will be held by skype prior to start of the recruitment and then 6-monthly thereafter. The study protocol will be approved by the DSMC prior to enrolment. Any subsequent updates and implications will be reviewed periodically.

12.2. Trial Steering committee (TSC)

The TSC is responsible for supporting the Sponsor in designing a scientifically sound study. In this capacity, the members of the TSC shall address and resolve scientific issues encountered during the study. The TSC would take decisions on protocol amendments (along with DSMC, if required) and communicate with the research teams. All changes would be implemented following ethics approval from all participating bodies.

Name	Address	Email / Phone
Prof Lisa K Micklesfield (Independent chair)	University of Witwatersrand, Johannesburg, South Africa	Lisa.Micklesfield@wits.ac.za
Dr. G. Krishnaveni (Independent member)	CSI Holdsworth Memorial Hospital, Mysore, India	gv.krishnaveni@gmail.com
Prof Louise Bowman (Independent member)	CTSU, Nuffield Department of Population Health, Oxford	louise.bowman@ndph.ox.ac.uk
Prof Fredrik Karpe (Principal investigator)	OCDEM, Radcliffe department of Medicine, Oxford	fredrik.karpe@ocdem.ox.ac.uk
Prof Caroline Fall (Co-investigator)	MRC-LEU, University of Southampton, Southampton, UK	chdf@mrc.soton.ac.uk
Dr. Senthil Vasan (Trial Manager, Non-voting members)	OCDEM, Radcliffe department of Medicine, Oxford	senthil.vasan@ocdem.ox.ac.uk
Dr. Melissa Lennartz-Walker (Newton Fund Program Manager, MRC)	MRC	Melissa.Lennartz-Walker2@mrc.ukri.org

For peer review only

12.3. Executive committee

The executive committee includes active investigators who will be directly involved in the day-to-day running of the trial

Name	Address	Email / Phone
Prof Fredrik Karpe (Principal investigator)	OCDEM, Radcliffe department of Medicine, Oxford	fredrik.karpe@ocdem.ox.ac.uk
Dr. Senthil Vasan	OCDEM, Radcliffe department of Medicine, Oxford	senthil.vasan@ocdem.ox.ac.uk
Prof Andrew Prentice	MRC Unit The Gambia at LSHTM, Atlantic Boulevard, Fajara, P.O. Box 273, Banjul, The Gambia	Andrew.Prentice@lshtm.ac.uk
Dr. Modou Jobe	MRC Unit The Gambia at LSHTM, Atlantic Boulevard, Fajara, P.O. Box 273, Banjul, The Gambia	mojobe@mrc.gm
Dr. Mustapha Bittaye	MRC Unit The Gambia at LSHTM, Atlantic Boulevard, Fajara, P.O. Box 273, Banjul, The Gambia	mubittaye@mrc.gm
Prof Jiji Matthews	Department of Obstetrics and Gynaecology, Christian Medical College & Hospital, Vellore, India	coronistrial@yahoo.co.in
Dr Swathi Rathore	Department of Obstetrics and Gynaecology, Christian Medical College & Hospital, Vellore, India	swatixrathore@gmail.com

13. ETHICAL AND REGULATORY CONSIDERATIONS

13.1. ICH-GCP

The study will be conducted in accordance with the protocol and applicable International Conference on Harmonisation (ICH) GCP guidelines.

For peer review only

13.2. Approvals

The protocol, protocol amendments, ICF and the PIS will be submitted to the OxTREC, and host institution (MRCG and Ethics committee, CMC, Vellore) for written approval. Additionally, approvals will also be sought from Indian Council of Medical Research (ICMR) and the Medical control agency, The Gambia. The Investigator will submit and, where necessary, obtain approval from the above parties for all substantial amendments to the original approved documents.

13.3. Participant Confidentiality

The study staff will ensure that the participants' anonymity is maintained. The participants will be identified only by a participant ID number on all study documents and any electronic database. All documents will be stored securely and only accessible by study staff and authorised personnel. The study will comply with the Data Protection Act, which requires data to be anonymised as soon as it is practical to do so. Personal data will be not be linked to research data and maintained in separate files which are password protected and accessible only to authorized staff of the study team. More details are provided under Section 11.

13.4. Expenses and Benefits

Participants will be paid a sum equivalent to their day wage for their study visit as agreed by the respective ethics committees. Non-working mothers will be provided a compensation as agreeable by the respective ethics committees of Vellore and The Gambia. Reasonable travel expenses for any study-related visits will be reimbursed on production of receipts, or a mileage allowance provided as appropriate. The study participants will not have to pay for any study-related procedures (OGTT, ultrasound scans and study consultations).

13.5. Annual Progress Report

The PI would submit annually, a Progress Report to the OxTREC with a copy to CTRG. Annual report will also be submitted to the funding body – The MRC, UK.

13.6. Other Ethical Considerations

The study involves non-invasive measurements of the new born. Prior informed consent will be obtained from either the father or the mother to record new-born measurements. The parents of the new born will be informed that they can withdraw the consent from obtaining their child's measurements at any time point. No personal data of the new born will be collected. New-born's will be identified by a unique study identification number and linked to the mothers' research data of the mother and father's study identification number.

14. FINANCE AND INSURANCE

14.1. Funding

The study is funded by the Medical Research Council, UK and Global Challenges Research Fund, UK for the UK and the Gambian costs and by the Department of Biotechnology (DBT), India for the Indian costs. The Indian costs will be directly dealt between DBT and CMC, Vellore. The

For peer review only

sponsor of the study is Oxford University. The funders have no role in study design; collection, management, analysis, and interpretation of data; writing of the report; and the decision to submit the report for publication.

14.2. Insurance

The University of Oxford has a specialist insurance policy in place which would operate in the event of any participant suffering harm as a result of their involvement in the research (Newline Underwriting Management Ltd, at Lloyd's of London).

15. PUBLICATION POLICY

The Investigators will be involved in reviewing drafts of the manuscripts, abstracts, press releases and any other publications arising from the study. The study will be registered and the study protocol will be made available in publically accessible websites such as ISRCTN and some national registries (Clinical Trials Registry, India). The final results of the study will be published in peer-reviewed scientific journals. Authorship will be determined in accordance with the ICMJE guidelines and other contributors will be acknowledged. For those journals that request sharing of the analyzable data sets, requests should be made to the PI-Fredrik Karpe. All publications will acknowledge that the study was funded by MRC, UK and DBT, India.

In order to disseminate our research findings with general public and study participants, we will engage with our communities, the wider public, the media, the broad scientific community and national and international agencies. We will explain the relevance of our research to our research participants and provide feedback of the results to them. We will use websites, the written press, TV and radio, and presentations to lay and school groups in order to ensure that our research findings reach the widest possible public audience. We will hold annual retention meetings of the study participants where results of the interim analysis and final study will be conveyed.

For peer review only

16. REFERENCES

1. Buchanan TA, Xiang AH. Gestational diabetes mellitus. J Clin Invest 2005;115(3):485-91.
2. Reece EA, Leguizamón G, Wiznitzer A. Gestational diabetes: the need for a common ground. Lancet 2009;373(9677):1789-97.
3. Bellamy L, Casas JP, Hingorani AD, Williams D. Type 2 diabetes mellitus after gestational diabetes: a systematic review and meta-analysis. Lancet 2009;373(9677):1773-9.
4. Qin J, Li Y, Cai Z, Li S, Zhu J, Zhang F at al. A metagenome-wide association study of gut microbiota in type 2 diabetes. Nature 2012;490(7418):55-60.
5. Sanz Y, Santacruz A, Gauffin P. Gut microbiota in obesity and metabolic disorders. Proc Nutr Soc 2010;69(3):434-41.
6. Collado MC, Isolauri E, Laitinen K, Salminen S. Distinct composition of gut microbiota during pregnancy in overweight and normal-weight women. Am J Clin Nutr 2008;88(4):894-9.
7. Monda V, Villano I, Messina A, Valenzano A, Esposito T, Moscatelli F et al. Exercise Modifies the Gut Microbiota with Positive Health Effects. Oxid Med Cell Longev 2017;2017:3831972.
8. Le Chatelier E, Nielsen T, Qin J, Prifti E, Hildebrand F, Falony G et al. Richness of human gut microbiome correlates with metabolic markers. Nature 2013;500(7464):541-6.
9. Metzger BE, Buchanan TA, Coustan DR, de Leiva A, Dunger DB, Hadden DR et al. Summary and recommendations of the Fifth International Workshop-Conference on Gestational Diabetes Mellitus. Diabetes Care 2007;30 Suppl 2:S251-60.
10. Hunt KJ, Schuller KL. The increasing prevalence of diabetes in pregnancy. Obstet Gynecol Clin North Am 2007;34(2):173-99
11. Sacks DA, Hadden DR, Maresh M, Deerochanawong C, Dyer AR, Metzger BE at al. Frequency of gestational diabetes mellitus at collaborating centers based on IADPSG consensus panel recommended criteria: the Hyperglycemia and Adverse Pregnancy Outcome (HAPO) Study. Diabetes Care 2012;35(3):526-8.
12. Hunsberger M, Rosenberg KD, Donatelle RJ. Racial/ethnic disparities in gestational diabetes mellitus: findings from a population-based survey. Womens Health Issues 2010;20(5):323-8.
13. Savitz DA, Janevic TM, Engel SM, Kaufman JS, Herring AH. Ethnicity and gestational diabetes in New York City, 1995-2003. BJOG 2008;115(8):969-78.
14. Seshiah V, Balaji V, Balaji MS, Sanjeevi CB, Green A. Gestational diabetes mellitus in India. J Assoc Physicians India 2004;52:707-11.
15. Macaulay S, Dunger DB, Norris SA. Gestational diabetes mellitus in Africa: a systematic review. PLoS One 2014;9(6):e97871.
16. Harlev A, Wiznitzer A. New insights on glucose pathophysiology in gestational diabetes and insulin resistance. Curr Diab Rep 2010;10(3):242-7.
17. Koivusalo SB, Rönö K, Klemetti MM, Roine RP, Lindström J, Erkkola M et al. Gestational Diabetes Mellitus Can Be Prevented by Lifestyle Intervention: The Finnish Gestational Diabetes Prevention Study (RADIEL): A Randomized Controlled Trial. Diabetes Care 2016;39(1):24-30.
18. Cani PD, Delzenne NM. The role of the gut microbiota in energy metabolism and metabolic disease. Curr Pharm Des 2009;15(13):1546-58.
19. Gijsbers L, Ding EL, Malik VS, de Goede J, Geleijnse JM, Soedamah-Muthu SS. Consumption of dairy foods and diabetes incidence: a dose-response meta-analysis of observational studies. Am J Clin Nutr 2016;103(4):1111-24.
20. Chen M, Sun Q, Giovannucci E, Mozaffarian D, Manson JE, Willett WC et al. Dairy consumption and risk of type 2 diabetes: 3 cohorts of US adults and an updated meta-analysis. BMC Med 2014;12:215.

For peer review only

21. Dugoua JJ, Machado M, Zhu X, Chen X, Koren G, Einarson TR. Probiotic safety in pregnancy: a systematic review and meta-analysis of randomized controlled trials of Lactobacillus, Bifidobacterium, and Saccharomyces spp. J Obstet Gynaecol Can 2009;31(6):542-52.
21. Russo LM, Nobles C, Ertel KA, Chasan-Taber L, Whitcomb BW. Physical activity interventions in pregnancy and risk of gestational diabetes mellitus: a systematic review and meta-analysis. Obstet Gynecol 2015;125(3):576-82.
22. Sanabria-Martínez G, García-Hermoso A, Poyatos-León R, Álvarez-Bueno C, Sánchez-López M, Martínez-Vizcaíno V. Effectiveness of physical activity interventions on preventing gestational diabetes mellitus and excessive maternal weight gain: a meta-analysis. BJOG 2015;122(9):1167-74.
23. Barakat R, Pelaez M, Cordero Y, Perales M, Lopez C, Coteron J et al. Exercise during pregnancy protects against hypertension and macrosomia: randomized clinical trial. Am J Obstet Gynecol 2016;214(5):649 e1-8.
24. Wiebe HW, Boulé NG, Chari R, Davenport MH. The effect of supervised prenatal exercise on fetal growth: a meta-analysis. Obstet Gynecol 2015;125(5):1185-94.
25. Barakat R, Cordero Y, Coteron J, Luaces M, Montejo R. Exercise during pregnancy improves maternal glucose screen at 24-28 weeks: a randomised controlled trial. Br J Sports Med 2012;46(9):656-61.
26. de Barros MC, Lopes MA, Francisco RP, Sapienza AD, Zugaib M. Resistance exercise and glycemic control in women with gestational diabetes mellitus. Am J Obstet Gynecol 2010;203(6):556 e1-6.
27. ACOG Committee Opinion No. 650. Physical Activity and Exercise During Pregnancy and the Postpartum Period. Obstet Gynecol 2015;126(6):e135-42.
28. Campbell SC, Wisniewski PJ. Exercise is a Novel Promoter of Intestinal Health and Microbial Diversity. Exerc Sport Sci Rev 2017;45(1):41-47.
29. Lindsay KL, Walsh CA, Brennan L, McAuliffe FM at al. Probiotics in pregnancy and maternal outcomes: a systematic review. J Matern Fetal Neonatal Med 2013;26(8):772-8.
30. Laitinen K, Poussa T, Isolauri E; Nutrition, Allergy, Mucosal Immunology and Intestinal Microbiota Group. Probiotics and dietary counselling contribute to glucose regulation during and after pregnancy: a randomised controlled trial. Br J Nutr 2009;101(11):1679-87.
31. Luoto R, Laitinen K, Nermes M, Isolauri E. Impact of maternal probiotic-supplemented dietary counselling on pregnancy outcome and prenatal and postnatal growth: a double-blind, placebo-controlled study. Br J Nutr 2010;103(12):1792-9.
32. Zheng J, Feng Q, Zheng S, Xiao X. The effects of probiotics supplementation on metabolic health in pregnant women: An evidence based meta-analysis. PLoS One. 2018 May 21;13(5):e019777
33. Hajifaraji M, Jahanjou F, Abbasalizadeh F, Aghamohammadzadeh N, Abbasi MM, Dolatkhah N. Effect of probiotic supplements in women with gestational diabetes mellitus on inflammation and oxidative stress biomarkers: a randomized clinical trial. Asia Pac J Clin Nutr. 2018;27(3):581-591.
34. Nitert MD, Barrett HL, Foxcroft K, Tremellen A, Wilkinson S, Lingwood B et al. SPRING: an RCT study of probiotics in the prevention of gestational diabetes mellitus in overweight and obese women. BMC Pregnancy Childbirth. 2013 Feb 25;13:50.
35. Barakat R, Pelaez M, Cordero Y, Perales M, Lopez C, Coteron J et al. Exercise during pregnancy protects against hypertension and macrosomia: randomized clinical trial. Am J Obstet Gynecol 2016;214(5):649 e1-8.

For peer review only

36. Barakat R, Cordero Y, Coteron J, Luaces M, Montejo R. Exercise during pregnancy improves maternal glucose screen at 24-28 weeks: a randomised controlled trial. Br J Sports Med 2012;46(9):656-61.
37. Poston L, Bell R, Croker H, Flynn AC, Godfrey KM, Goff L at al. Effect of a behavioural intervention in obese pregnant women (the UPBEAT study): a multicentre, randomised controlled trial. Lancet Diabetes Endocrinol 2015;3(10):767-77
38. da Silva SG, Hallal PC, Domingues MR, Bertoldi AD, Silveira MFD, Bassani D et al. A randomized controlled trial of exercise during pregnancy on maternal and neonatal outcomes: results from the PAMELA study. Int J Behav Nutr Phys Act. 2017 Dec 22;14(1):175.
39. Haakstad LA, Bø K. Effect of regular exercise on prevention of excessive weight gain in pregnancy: a randomised controlled trial. Eur J Contracept Reprod Health Care. 2011 Apr;16(2):116-25.
40. Wang C, Wei Y, Zhang X, Zhang Y, Xu Q, Sun Y et al. A randomized clinical trial of exercise during pregnancy to prevent gestational diabetes mellitus and improve pregnancy outcome in overweight and obese pregnant women. Am J Obstet Gynecol. 2017 Apr;216(4):340-351.
41. Vinter CA, Jensen DM, Ovesen P, Beck-Nielsen H, Jørgensen JS. The LiP (Lifestyle in Pregnancy) study: a randomized controlled trial of lifestyle intervention in 360 obese pregnant women. Diabetes Care 2011;34(12):2502-7.
42. Renault KM, Nørgaard K, Nilas L, Carlsen EM, Cortes D, Pryds O at al. The Treatment of Obese Pregnant Women (TOP) study: a randomized controlled trial of the effect of physical activity intervention assessed by pedometer with or without dietary intervention in obese pregnant women. Am J Obstet Gynecol 2014;210(2):134 e1-9.
43. Koivusalo SB, Rönö K, Klemetti MM, Roine RP, Lindström J, Erkkola M et al. Gestational Diabetes Mellitus Can Be Prevented by Lifestyle Intervention: The Finnish Gestational Diabetes Prevention Study (RADIEL): A Randomized Controlled Trial. Diabetes Care 2016;39(1):24-30.
44. Dodd JM, Turnbull D, McPhee AJ, Deussen AR, Grivell RM, Yelland LN at al. Antenatal lifestyle advice for women who are overweight or obese: LIMIT randomised trial. BMJ 2014;348:g1285.
45. Grivell RM, Yelland LN, Deussen A, Crowther CA, Dodd JM. Antenatal dietary and lifestyle advice for women who are overweight or obese and the effect on fetal growth and adiposity: the LIMIT randomised trial. BJOG. 2016 Jan;123(2):233-43.
46. Bennett CJ, Walker RE, Blumfield ML, Gwini SM, Ma J, Wang F. Interventions designed to reduce excessive gestational weight gain can reduce the incidence of gestational diabetes mellitus: A systematic review and meta-analysis of randomised controlled trials. Diabetes Res Clin Pract. 2018 Jul;141:69-79.
47. Shepherd E, Gomersall JC, Tieu J, Han S, Crowther CA, Middleton P. Combined diet and exercise interventions for preventing gestational diabetes mellitus. Cochrane Database Syst Rev. 2017 Nov 13;11:CD010443
48. Petrella E, Malavolti M, Bertarini V, Pignatti L, Neri I, Battistini NC et al. Gestational weight gain in overweight and obese women enrolled in a healthy lifestyle and eating habits program. J Matern Fetal Neonatal Med. 2014 Sep;27(13):1348-52.

For peer review only

18. APPENDIX A: STUDY FLOW CHART

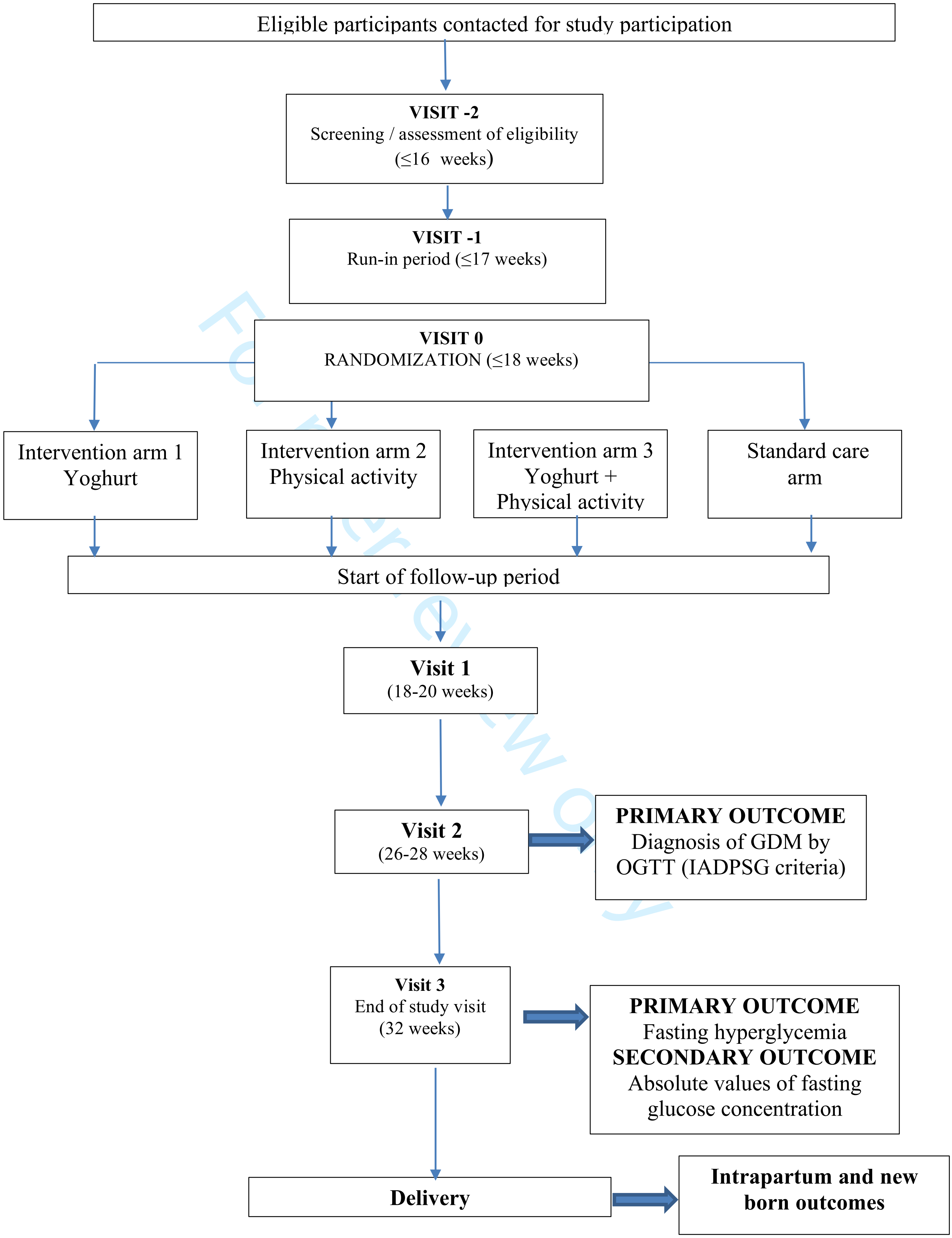

For peer review only - http://bmjopen.bmj.com/site/about/guidelines.xhtml

19. APPENDIX B: STUDY TIMELINES (approximate)

	2018-2019		2019		2020		2021	
	July - Nov	Dec 2018 - Jan 2019	Feb - June	Jul - Dec	Jan-Jun	Jul - Dec	Jan-Jun	Jul - Dec
Protocol preparation								
Regulatory approvals								
TSC meeting								
Investigators meeting								
Staff training								
Study initiation								
Recruitment & follow up								
Study close out								
Interim analysis								
Monitoring visits								
Data quality checks								
DMC meeting								
Annual report preparation								
Final data analysis								
Preparation of manuscripts								
Publication of manuscripts								
Final report submission								

For peer review only

20. APPENDIX C: SCHEDULE OF STUDY PROCEDURES

Procedures	Visits						
	Visit -2	Visit -1	Visit 0	Visit 1	Visit 2	Visit 3	Delivery
	Screening ≤16 weeks	Run-in-phase ≤17 weeks	Randomization Week ≤18	Week 18-20	Week 26-28	Week 32	
Informed consent	X						
Eligibility assessment	X						
Demographics	X						
History	X						
Height	X						
Weight	X	X	X	X	X	X	X
Blood pressure	X	X	X	X	X	X	X
Antenatal examination	X	X	X	X	X	X	
Dairy consumption questionnaire		X					
Barriers questionnaire	X					X	
Baseline PA monitoring with Vivofit 4		X					
PA step-count monitoring			X	X	X	X	
PA assessment (Baseline)		X					
Randomisation			X				
OGTT	X				X		
Dating scan	X						
Morphology scan				X			
Ultrasound scan						X	
Fasting plasma glucose						X	
Intrapartum assessment (mother)							X
New born assessment							X

For peer review only

21. APPENDIX D: PATIENT INFORMATION SHEET

PATIENT INFORMATION SHEET
Lifestyle interventions during pregnancy to prevent diabetes (PRIMORDIAL Study)

OxTREC reference number 44-18
Version 2.1 dated 19th September 2019

1. What's the purpose of this research?

Around 3 in 10 pregnant women are at risk for developing diabetes during pregnancy, and the risk is higher in African and Asian women. Diabetes during pregnancy imposes serious health effects to both the mother and the baby. For some women, this may be a sign of developing diabetes permanently in the future. In this study, we would like to find out, if simple lifestyle changes in diet and physical activity during pregnancy might help in reducing the risk of developing diabetes in pregnant women.

2. Why have I been invited to take part?

You are invited to take part in this study because you have been identified to have one (or more than one) condition that places you at an increased risk for developing diabetes during pregnancy. Risk factors can be being overweight or obese, previous pregnancy with diabetes, Age ≥25 years, first-degree relative with diabetes, previous pregnancy with large baby (≥3.5kg), previous pregnancy with hypertension/seizures, and history of impaired fasting glucose.

3. What's involved for me?

After study explained to you, if you agree to take part you will have to provide your consent. There are 6 visits in this study. Y our doctor may request you to attend the clinic for additional care if required. Information on what you will be asked to do at each visit are as below

Screening visit (Visit -2): At this first visit, you will be asked to do a timed blood test called the oral glucose tolerance test (OGTT), an ultrasound scan, answer a barriers questionnaire and routine clinical examination by your doctor.

A sample of your blood will be taken to measure fasting glucose (sugar). You should have not consumed any food or drink (except water) for a minimum 8 hours from previous night. You will then be given a sugary drink and subsequently blood samples will be taken at one hour and two hours from the time you have taken the drink. You cannot eat anything during the test but you can drink water. The test will take about 2 hours 15 minutes to be completed. The doctor will then perform an ultrasound scan to find out how many weeks of pregnancy are you currently in and also will be able to tell you the expected date of delivery. In addition, the doctor will take some basic measurements of the growing baby. The results of the scan will be informed to you immediately. The scans will take about 30 minutes. You will also to fill a questionnaire relating to difficulties you anticipate, that will prevent you from being physically active during pregnancy and eating healthy food including yoghurt during pregnancy. This will take about 30 minutes to complete.

You will be contacted within one week regarding your blood test and your eligibility to further continue in the study.

Run-in-phase (Visit -1): At the second visit, you will be asked questions relating to your regular milk-products and yoghurt consumption. This will take about 15 minutes for each of the questionnaire. You will also be asked to wear a watch-like device around your wrist for the next 7 days and do your routine day-to-day work. You will also be seen in the clinic by the doctor for general examination

Randomization (Visit 0): To be eligible to participate further in this study, you should have worn the wrist band on all 7 days as advised.

For peer review only

You will be allotted to one of the study arms – the yoghurt arm or the physical activity arm or the yoghurt and the physical activity arm or standard care arm. If you are allocated to the diet arm, you will have to consume a pot of yoghurt (200g) till 32 weeks of pregnancy. In the physical activity arm, you will be asked to increase your walking everyday till 32 weeks of pregnancy. In the yoghurt and physical activity arm, you will have to do both of the above. In the standard care arm, you will receive regular care from your physician.

Visit 1: In the fourth visit, the physician will perform an ultrasound scan to see the growth of the baby. Routine antenatal examination will be done by your doctor.

Visit 2: In the fifth visit, you will be asked to do blood tests similar to your first visit seen in the clinic by the doctor for routine antenatal examination.

Visit 3: A fasting blood sample (minimum 8-10 hours fasting from previous night) for blood glucose measurement will be taken and another scan to see if your baby is growing well. You will also be asked to complete a barriers questionnaire and provide feedback about your participation in the trial. All other routine antenatal care will be provided by your doctor.

This is the last visit and all interventions stop here. You will be contacted during delivery for measurements of yourself and new-born baby during delivery.

In the event that you develop diabetes at any point during the study, your physician will advise you on additional medications.

During the entire period you will be home visited by a field worker or telephonically contacted by a research staff to check if everything is ok.

4. What will happen to my data?

Research data You will be given a study number so that any information that you provide to us will be made anonymous. All data will be securely stored in the research office, kept in locked cupboards/secure servers in locked rooms with restricted access. All data will be owned by your local research investigator, kept in locked cupboards/secure servers in locked rooms with restricted access. Anonymised data will be shared between researchers in the Gambia, India, and Oxford, UK for research purposes. All research data collected as electronic records will be kept indefinitely in electronic data archives in The Gambia and may be used for future research studies with your consent. Responsible members of the research team may be given access to data for monitoring and/or audit of the study to ensure that the research is complying with applicable regulations.
Personal data Your name, date of birth, address and telephone number will be stored separately in order to invite you to follow-up visits and for the field staff to visit you at home. This information will not be shared with other researchers.

5. What will happen to my samples?

The blood samples will be used for study purpose only and analysed locally. Samples will not be shipped/stored overseas and will not be shared with other researchers. No genetic tests are carried out in this study.

6. Are there any risks in taking part?

There may be mild bruising/swelling in the area where blood is drawn. Consumption of oral glucose solution in empty stomach may cause nausea, vomiting, headache. Ultrasound scans during pregnancy are considered safe to the mother or the baby as the radiation exposure is minimal.

By consuming yoghurt, you may feel nauseated, experience vomiting, bowel upset or diarrhoea. Physical activity during pregnancy is considered generally safe. However, you may experience some common adverse effects like dizziness, headache, and breathlessness on exertion, easy fatigability and muscle aches. If either of the interventions is not tolerable, you can contact your doctor and he may stop the intervention if necessary.

For peer review only

7. What are the benefits of taking part?

The direct benefits of participating in this study is that your blood sugar levels, blood pressure and weight will be closely monitored. By achieving strict control of your sugar levels, blood pressure and weight, we hope to see immediate benefits and lifelong health impact on yourself and your baby.
You will be sensitised to healthy lifestyle during pregnancy.

8. Do I have to take part?

Participation in this study is voluntary. You can ask questions/concerns about the study to one of the members of the research team before deciding whether to take part. Your decision will not affect your routine clinical care. You can also withdraw from the study without penalty at any time.

10.Has the study been reviewed by an ethics committee?

The study has been reviewed and approved by the ethics committees of University of Oxford, UK, Christian Medical College & Hospital, Vellore, India and the MRC Unit, The Gambia.

11.What if I have any questions or want to raise a concern?

If you have any concerns about the study at any point or wish to complain about any aspect of the way in which you have been approached or treated in this study, you may contact one of the below mentioned persons

Research team:
Name:
Address:
Phone number:

Member independent of the study team
Name
Address:
Phone number:

12. Data protection
The University of Oxford is responsible for ensuring the safe and proper use of any personal information you provide, solely for research purposes.

For peer review only

22. APPENDIX E: INFORMED CONSENT FORM

INFORMED CONSENT FORM

Lifestyle interventions during pregnancy to prevent diabetes (PRIMORDIAL Study)

OxTREC reference number 44-18

1. I confirm that I have read the information sheet for the above study. I have had the opportunity to consider the information and ask questions, and have had these answered satisfactorily.	Yes / No
2. I give consent for my research data to be stored locally and shared with other investigators overseas	Yes / No
3. I agree to take part in this study.	Yes / No

______________________ ______________________ ______________________

Name of participant | Date | Signature

______________________ ______________________ ______________________

Name of person taking consent | Date | Signature

- For illiterate participants:

____

Name of participant | Thumbprint of participant

______________________ ______________________ ______________________ ______________________

Name of witness | Relationship to participant | Date | Signature

______________________ ______________________ ______________________

Name of person taking consent | Date | Signature

For peer review

23. APPENDIX F: AMENDMENT HISTORY

All protocol amendments whenever a new version of the protocol is produced will be updated here.

Amendment No.	Protocol Version No.	Date issued	Author(s) of changes	Details of Changes made
1	2.1	19th October 2018	Senthil K Vasan<br>Fredrik Karpe<br>Caroline Fall<br>Clive Osmond	1. Stratification for risk factors during randomization (Section 7.2)<br>2. Explanations to sample size calculation (Section 10.2)<br>3. Study analysis details included unde section 10.3.1 and 10.3.2<br>4. Details of DSMB included under section 12.<br>5. SAE reporting to DSMB and OxTREC is included under section 9.3.<br>6. Skin fold thickness measurement of the new born is removed from the protocol<br>7. Under Section 13,4, the following line is changed "Participants will be paid a sum equivalent to their day wage for their study visit as agreed by the respective ethics committees".
2	3.0	09th January 2019	Senthil K Vasan<br>Fredrik Karpe<br>Caroline Fall<br>Clive Osmond<br>Dylan Thompson	1. Additional change to primary outcome measure (eelevated fasting plasma glucose ≥5.1 mmol/l at 32 weeks GA) in Page 4<br>2. Secondary objective changed tc "To determine the effect of life style intervention (yoghurt and PA), given to high-risk women from 18 weeks of gestation, on fasting blood glucose at 32 weeks (Pages 4 and 16)<br>3. Low birth weight changed to 'birth weight' under section other outcome measures in Page 5

For peer review only

				4. Ponderal index and neonatal anthropometry deleted under neonatal outcomes in Pages 5, 16, 19, 27)<br>5. Run-in visit - Baseline PA will be assessed (blinded to participants) using a wearable accelerometer device (Garmin Vivofit 4 fitness band) for 7 day[illegible] to objectively measure baselin[illegible] step count in all participants (Page 17)<br>6. Visit 3 (32 weeks GA, End of Study visit): morphology scan changed to ultrasound scan (Pages 17, 26)<br>7. Physical actvity intervention Th[illegible] recommendation changed to 80% of the absolute target increase from the average targe[illegible] step count measured during run-in-phase (Pages 18 and 29)<br>8. Group session will use a patient participant initiative (PPI) approach where participants wi[illegible] be involved in designing the type of group activity, which may include activities such as group walking, dancing, stretching exercises etc. (Page 18)<br>9. Reassessment of PA activity: All participants will be asked to objectively measure step count for next 7 days using Garmin Vivofit4 at visit 2 (26-28 weeks) Women not randomized to the diet and standard care arm will have blinded assessments, whil[illegible] women on PA arm (PA arm or Diet+PA) will continue to achieve target step counts without blinded assessment (Page 18).

For peer review only

					10. Data collection process – height and weight of father newly added (Pages 19, 21, 23-24)<br>11. Inclusion criteria changed to ≥1[illegible] weeks and ≤ 16 weeks of gestational age (Pages 20,23)<br>12. Inclusion criteria stamen revise[illegible] as: In addition to the above, pregnant women should meet at least one of the following criteria for high-risk GDM (Page 20).<br>13. Exclusion criteria related to hypertension changed as “Currently on treatment for hypertension (pre-gestational o[illegible] gestational)” (Page 20).<br>14. New exclusion criteria added: Physical disability to PA and/or known lactose intolerance (pag[illegible] 21).<br>15. Details of stratification at randomisation provided: Random allocation to intervention will be prestratifie[illegible] for age, BMI and risk factor for GDM within each study centre. We will create two age bands (<25 y and ≥ 25 years), two BM[illegible] categories (<25, ≥26) and presence of any risk factor (No 0/ Yes 1) (Page 23).<br>16. PA intervention details and 80[illegible] absolute target step count char[illegible] included in Page 29.<br>17. PA compliance definition included in Page 29 as “compliance” will be defined as will be defined as at least 80% [illegible] absolute target increase achieved on an average over 7-days period.<br>18. Statistical methods changed as “The primary analysis will be intention-to-treat. We will also

For peer review only

				do a secondary analysis using the per protocol principle" in page 31.<br>19. Members of the executive committee newly included in Pages 36-37.<br>20. Appendix A – study flow chart Primary and secondary outcomes changed in Page 42.<br>21. Timelines of investigators meeting, study initiation, staff training in Gnat chart changed i Page 43
3	4.0	09th February 2019	Senthil K Vasan<br>Fredrik Karpe<br>(Changes as suggested from TSC)	1. Primary objective – outcome measure: 26-28 weeks included newly<br>2. 'Other outcome measures' rename Trial and post-trial surveillance' (Sections 1, 4).<br>3. maternal blood pressure added as objective and serial blood pressure measurement at all visits included a outcome measurement (Sections 1, 4).<br>4. For maternal outcome measurements under 'Trial and post-trial surveillance' data on intra and postpartum outcomes will be captured on eCRF from delivery records (Section 1).<br>5. New born outcomes measure preterm births will be based on ultrasound based gestational age recorded at screening (Section 1).<br>6. DSMB changed to DMC (data monitoring committee) in abbreviations. New abbreviations added – LSHTM, PID (Section 2).<br>7. Protocol change: Under Section 5 – Run-in-visit – consumption of yoghurt by all participants for 7 day removed.

For peer review only

				8. Protocol change: Section 5 – Run-in visit and in data collection process, Section 7: Baseline dietary assessment using FFQ changed to background dairy consumption questionnaire.<br>9. Under Section 5 and Section 7– Visits 1-3: 'routine' added to antenatal examination.<br>10. Under Section 5 – Visit 3: GDM women will continue on allocatec study intervention till week 32 in addition to the treatment provided as per local obstetric practice. This i changed from previous version that stated 'terminating women who develop GDM from further intervention'.<br>11. Protocol change: Under Section 5 - Interventions: Newly added: All other (yoghurt and standard arm) will also be provided with fitness bands to objectively measure their routine PA behaviour throughout the study. However, step counts wil be blinded for these participants.<br>12. Under Section 6.2 – Medications revised to include women who are hypothyroid on thyroxine supplementation, low dose aspirin for pre-eclampsia) and women conceived by ovulation induction using clomiphene citrate or metformin for PCOD will not be excluded from the study.<br>13. Section 6.3 – Exclusion criteria – GDM diagnosed prior to screening visit based on IADPSG criteria or documented raised HbA1C.<br>14. Section 7 – Post-trial surveillance – Capillary blood glucose (mother) deleted.<br>15. Section 7.3 – Screening and Eligibility assessment: Newly added 'In addition to above, participants

For peer review only

				should meet at least one of the below mentioned criteria to be termed as "high-risk" for inclusion into the study'<br>16. Section 7.4 – Randomisation: Newly added: After qualifying for eligibility and completion of the run-in-visit, details of age, BMI and GDM risk wi[illegible] be provided to the independent statistician, who will then assign the intervention arm. Randomisation allocation will be communicated to the study sites through emails.<br>17. Section 7.6.1 – Newly added under morphology scan - Participants with any foetal congenital anomaly, placental malformation or malposition or abnormal ultrasounc[illegible] features that can compromise pregnancy outcome according to th[illegible] investigator will be withdrawn from the study at this stage.<br>18. Section 7.6.1, 7.6.2, 7.6.3: Change made - Safety assessments to interventions (e.g. adverse events and/or SAE assessment).<br>19. Section 7.8.1 – more details provided for "Discontinuation/withdrawal"<br>20. Section 7.8.2 – Newly added "discontinuation from interventions".<br>21. Section 7.9 - more details provided for "lost to follow-up".<br>22. Section 7.11 – last scheduled visit changed from Visit 2 (26-28 weeks) to visit 3 (week 32).<br>23. Section 8.1.2 – Details of PA intervention provided under sections 8.1.2.1 – daily walking and 8.1.2.2 – Group activity sessions.<br>24. Section 9.1 – Details of AE reporting in eCRF added as 'Details of all events including onset date, causality to the study intervention

For peer review only

				will be reported, treatment given, stop date will be captured in the respective AE report form'.<br>25. Section 9.2.1 – Expected SAE – newly added.<br>26. Section 12 title changed as "Committee structures"<br>27. Section 12.1., 12.2., 12.3 – Roles an responsibilities of DMC, TSC and executive committees are included.<br>28. Section 13.1 renamed as ICH-GCP.<br>29. Section 15 – Publication Policy: Registration with ISRCTN and Clinica Trials Registry, India newly added.<br>30. Section 15 - Publication Policy: Fredrik Karpe will be the contact person for requests for sharing analysable data.<br>31. PPAQ removed at run-in-visit.
4	4.1	09th April 2019	Senthil K Vasan<br>Alexander Jarde<br>Fredrik Karpe	1. Gestational age at screening, run-in and randomisation visit are changed from 16, 17 and 18 weeks to ≤16, ≤17 and ≤18 weeks (Pages 17, 20-25)<br>2. Detailed randomisation procedure outlined in Page 23. Risk factors for GDM is remove from pre-stratification criteria.<br>3. Section 10 on Statistics and analysis (incl. 10.1 and 10.2) are expanded to provide more details (Pages 33-35)
5	4.2	18th July 2019	Senthil K Vasan<br>Fredrik Karpe	1. Scheduled visit 1 is change form week 22 to week 18-2 weeks. This is in line with ACOG and NICE antenatal guidelines for morphology scan at week 18-20 (Pages 17,21,25,46)

For peer review only

STANDARD PROTOCOL ITEMS: RECOMMENDATIONS FOR INTERVENTIONAL TRIALS

SPIRIT 2013 Checklist: PRIMORDIAL Study

Section/item	PageNo	Description
**Administrative information**		
Title	1	Descriptive title identifying the study design, population, interventions, and, if applicable, trial acronym
Trial registration	1	Trial identifier and registry name. If not yet registered, name of intended registry
Protocol version	1	Date and version identifier
Funding	1	Sources and types of financial, material, and other support
Roles and responsibilities	1	Names, affiliations, and roles of protocol contributors
	1	Name and contact information for the trial sponsor
	42	Role of study sponsor and funders, if any, in study design; collection, management, analysis, and interpretation of data; writing of the report; and the decision to submit the report for publication, including whether they will have ultimate authority over any of these activities
	39-41	Composition, roles, and responsibilities of the coordinating centre, steering committee, endpoint adjudication committee, data management team, and other individuals or groups overseeing the trial, if applicable (see Item 21a for data monitoring committee)
**Introduction**		
Background and rationale	9-16	Description of research question and justification for undertaking the trial, including summary of relevant studies (published and unpublished) examining benefits and harms for each intervention
Objectives	10,17	Specific objectives or hypotheses
Trial design	18	Description of trial design including type of trial (eg, parallel group, crossover, factorial, single group), allocation ratio, and framework (eg, superiority, equivalence, noninferiority, exploratory)
**Methods: Participants, interventions, and outcomes**		
Study setting	22-23	Description of study settings (eg, community clinic, academic hospital) and list of countries where data will be collected. Reference to where list of study sites can be obtained

For peer review only

Eligibility criteria	21	Inclusion and exclusion criteria for participants. If applicable, eligibility criteria for study centres and individuals who will perform the interventions (eg, surgeons, psychotherapists)
Interventions	19, 31	Interventions for each group with sufficient detail to allow replication, including how and when they will be administered
	29	Criteria for discontinuing or modifying allocated interventions for a given trial participant (eg, drug dose change in response to harms, participant request, or improving/worsening disease)
Outcomes	17, 25, 35	Primary, secondary, and other outcomes, including the specific measurement variable (eg, blood glucose, OGTT), analysis metric (eg, change from baseline, final value, time to event), method of aggregation (eg, median, proportion), and time point for each outcome. Explanation of the clinical relevance of chosen efficacy and harm outcomes is strongly recommended
Participant timeline	22-26	Time schedule of enrolment, interventions (including any run-ins and washouts), assessments, and visits for participants. A schematic diagram is highly recommended (see Figure)
Sample size	34,35	Estimated number of participants needed to achieve study objectives and how it was determined, including clinical and statistical assumptions supporting any sample size calculations
Recruitment	22,23	Strategies for achieving adequate participant enrolment to reach target sample size

Methods: Assignment of interventions (for controlled trials)

Allocation:

Sequence generation	24	Method of generating the allocation sequence (eg, computer-generated random numbers), and list of any factors for stratification. To reduce predictability of a random sequence, details of any planned restriction (eg, blocking) should be provided in a separate document that is unavailable to those who enrol participants or assign interventions
Implementation	24	Who will generate the allocation sequence, who will enrol participants, and who will assign participants to interventions

Methods: Data collection, management, and analysis

Data collection methods	37-38	Plans for assessment and collection of outcome, baseline, and other trial data, including any related processes to promote data quality (eg, duplicate measurements, training of assessors) and a description of study instruments (eg, questionnaires, laboratory tests) along with their reliability and validity, if known. Reference to where data collection forms can be found, if not in the protocol

For peer review only - http://bmjopen.bmj.com/site/about/guidelines.xhtml

Data management	36-38	Plans for data entry, coding, security, and storage, including any related processes to promote data quality (eg, double data entry; range checks for data values). Reference to where details of data management procedures can be found, if not in the protocol
Statistical methods	33-36	Statistical methods for analysing primary and secondary outcomes. Reference to where other details of the statistical analysis plan can be found, if not in the protocol
	35,36	Methods for any additional analyses (eg, subgroup and adjusted analyses)
	33,36	Definition of analysis population relating to protocol non-adherence (eg, as randomised analysis)
**Methods: Monitoring**		
Data monitoring	39	Composition of data safety monitoring committee (DSMC); summary of its role and reporting structure; statement of whether it is independent from the sponsor and competing interests; and reference to where further details about its charter can be found, if not in the protocol. Alternatively, an explanation of why a DMC is not needed
Harms	32	Plans for collecting, assessing, reporting, and managing solicited and spontaneously reported adverse events and other unintended effects of trial interventions or trial conduct
**Ethics and dissemination**		
Research ethics approval	41	Plans for seeking research ethics committee/institutional review board (REC/IRB) approval
Protocol amendments	40,54-60	Plans for communicating important protocol modifications (eg, changes to eligibility criteria, outcomes, analyses) to relevant parties (eg, investigators, REC/IRBs, trial participants, trial registries, journals, regulators)
Consent or assent	23	Who will obtain informed consent or assent from potential trial participants or authorised surrogates, and how
Confidentiality	38	How personal information about potential and enrolled participants will be collected, shared, and maintained in order to protect confidentiality before, during, and after the trial
Access to data	38	Statement of who will have access to the final trial dataset, and disclosure of contractual agreements that limit such access for investigators

For peer review only - http://bmjopen.bmj.com/site/about/guidelines.xhtml

Dissemination policy	43	Plans for investigators and sponsor to communicate trial results to participants, healthcare professionals, the public, and other relevant groups (eg, via publication, reporting in results databases, or other data sharing arrangements), including any publication restrictions
	43	Authorship eligibility guidelines and any intended use of professional writers
Biological specimens	28	Plans for collection, laboratory evaluation, and storage of biological specimens for genetic or molecular analysis in the current trial and for future use in ancillary studies, if applicable

*It is strongly recommended that this checklist be read in conjunction with the SPIRIT 2013 Explanation & Elaboration for important clarification on the items. Amendments to the protocol should be tracked and dated. The SPIRIT checklist is copyrighted by the SPIRIT Group under the Creative Commons "Attribution-NonCommercial-NoDerivs 3.0 Unported" license.

For peer review only
